# Design, Synthesis, and Phenotypic Profiling of Pyrano‐Furo‐Pyridone Pseudo Natural Products

**DOI:** 10.1002/anie.201907853

**Published:** 2019-08-28

**Authors:** Andreas Christoforow, Julian Wilke, Aylin Binici, Axel Pahl, Claude Ostermann, Sonja Sievers, Herbert Waldmann

**Affiliations:** ^1^ Department of Chemical Biology Max-Planck-Institute of Molecular Physiology Otto-Hahn-Straße 11 44227 Dortmund Germany; ^2^ Faculty of Chemistry and Chemical Biology Technical University Dortmund Otto-Hahn-Straße 6 44227 Dortmund Germany; ^3^ Compound Management and Screening Center, Dortmund Otto-Hahn-Str. 11 44227 Dortmund Germany

**Keywords:** cell painting assays, cell-based screening, high-throughput screening, inhibitors, pseudo natural products

## Abstract

Natural products (NPs) inspire the design and synthesis of novel biologically relevant chemical matter, for instance through biology‐oriented synthesis (BIOS). However, BIOS is limited by the partial coverage of NP‐like chemical space by the guiding NPs. The design and synthesis of “pseudo NPs” overcomes these limitations by combining NP‐inspired strategies with fragment‐based compound design through de novo combination of NP‐derived fragments to unprecedented compound classes not accessible through biosynthesis. We describe the development and biological evaluation of pyrano‐furo‐pyridone (PFP) pseudo NPs, which combine pyridone‐ and dihydropyran NP fragments in three isomeric arrangements. Cheminformatic analysis indicates that the PFPs reside in an area of NP‐like chemical space not covered by existing NPs but rather by drugs and related compounds. Phenotypic profiling in a target‐agnostic “cell painting” assay revealed that PFPs induce formation of reactive oxygen species and are structurally novel inhibitors of mitochondrial complex I.

## Graphical abstract

**Not quite natural**: Phenotypic profiling of pseudo natural products (NPs) obtained by de novo combination of pyridone‐ and dihydropyran fragments reveals a new class of inhibitors of mitochondrial complex I.

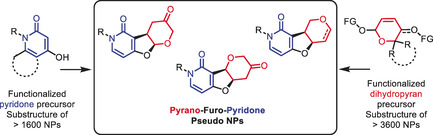

## Introduction

The discovery of bioactive small molecules with a view to illuminate biology is at the heart of chemical biology research. For the design of such compound classes, biological relevance is a key property to consider. One way to guarantee this relevance is to draw inspiration from the structural properties of the biologically pre‐validated molecular repository generated in evolution.^[1]^ This strategy underlies recently developed approaches like biology‐oriented synthesis (BIOS)^[2]^ and the complexity to diversity ring‐distortion/modification approach introduced by Hergenrother et al.^[3]^

In BIOS, complex natural products (NPs) are simplified to their fundamental core scaffolds for compound collection design, while characteristic NP properties are retained.^[4]^ However, the BIOS approach is biased in the exploration of chemical and biological space since it focuses primarily on the structures of the guiding NPs and their molecular target(s). This focus is limiting since the currently described NP scaffolds represent only a relatively small fraction of natural product‐like chemical space in a wider sense.^[5]^

**Figure 1:**
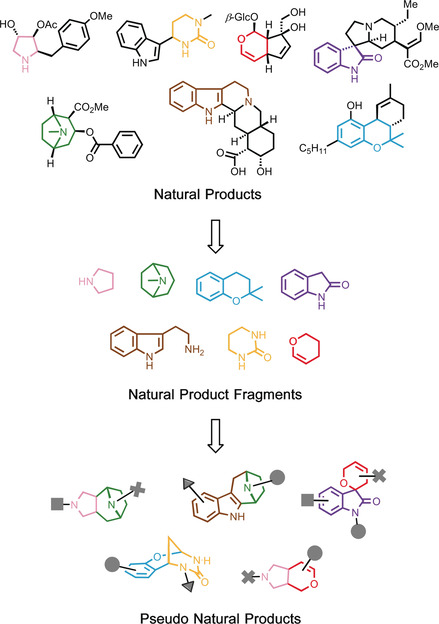
Design of pseudo NPs. NPs are deconstructed to their underlying fragments, which are then recombined in complexity‐generating transformations to yield unprecedented structures not available from current biosynthetic routes.

We recently proposed and provided proof for the concept of uniting the principles of BIOS with the logic of fragment‐based compound discovery^[6]^ to overcome these limitations. In this design strategy, biosynthetically unrelated NP‐derived fragments that represent NP structure and properties^[7]^ are combined de novo to populate NP‐like biologically relevant chemical space not covered by nature. To this end, guiding natural products are deconstructed by cheminformatic methods^[7]^ to their underlying fragments, which then are employed to inspire complexity‐generating synthesis routes for their recombination in unprecedented arrangements (Figure 1 illustrates the design approach). The molecular frameworks of such pseudo natural products (pseudo NPs) are not available from currently utilized biosynthetic machinery and bear the potential for unexpected and novel bioactivity.^[8]^ In light of their unpredictable bioactivity, pseudo NPs will best be evaluated in unbiased target‐agnostic cell‐based assays, monitoring for instance entire signaling cascades or covering a wide range of biological processes as it is the case for phenotypic profiling approaches.^[9]^

**Figure 2:**
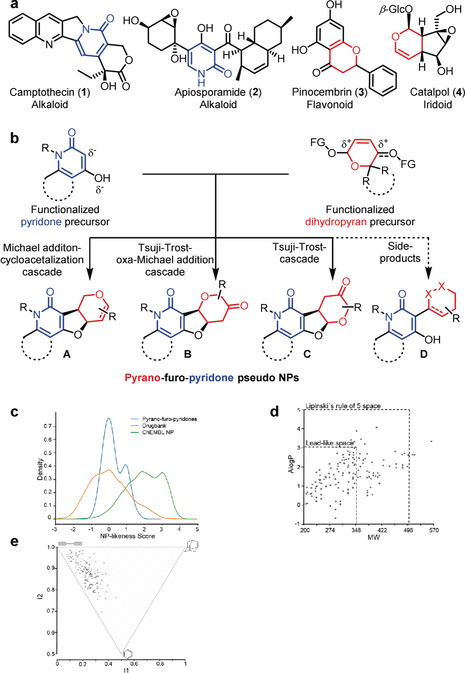
Design and cheminformatic analysis of PFPs. a) Selected NPs incorporating a 2‐pyridone fragment or dihydropyran fragment. b) Design of a pseudo NP collection. The fusion of functionalized pyridone precursors with functionalized dihydropyran fragments leads to three PFP pseudo NP classes **A**–**C** with bipodal connection between the pyridone‐ and dihydropyran fragments, as well as one compound class with mono‐podal connection (**D**). c) Comparison of NP‐likeness scores derived from PFPs (blue curve), DrugBank (orange curve) and NPs represented in ChEMBL (green curve). d) A Log P vs. MW scatter plot of PFPs. 98 % of molecules (158 out of 162 PFPs) fall into Lipinski's rule‐of‐five space with 61 % (99 out of 162 PFPs) exhibiting lead‐like properties. e) PMI plot for PFPs.

Herein, we describe the design and synthesis of a pseudo NP collection obtained by synthetic recombination of NP‐derived pyridone‐ and dihydropyran fragments to unprecedented pyrano‐furo‐pyridone (PFP) pseudo NPs (Figure 2 a,b). Analysis of their bioactivity in several cell‐based assays monitoring different biological processes and, in particular, in the multiparametric “cell painting assay”, which identifies the establishment of complex phenotypes, revealed that a distinct class of PFP pseudo NPs induce the formation of reactive oxygen species (ROS) by targeting mitochondrial complex I.

## Results and Discussion

For the design of a novel pseudo NP class,^[6]^ we intended to combine fragments derived from biosynthetically unrelated NPs with diverse biological activity to enable reconfiguration of protein binding patterns. It was planned to combine the fragments in three‐dimensional arrangements including formation of stereogenic centers since stereogenicity correlates favorably with bioactivity.[^6^, ^10^] In addition, it was planned to combine NP fragments with complementary heteroatoms, in particular nitrogen and oxygen, to create structural differences from the guiding NPs. With these design criteria in mind, we envisioned combining 2‐pyridone‐ and dihydropyran fragments, which define the structures of numerous NPs with diverse bioactivities. For instance, the pyridone alkaloid camptothecin (**1**) is a topoisomerase I‐targeting anti‐cancer drug,^[11]^ and apiosporamide (**2**) is an antifungal NP,^[12]^ whereas for instance the dihydropyran‐containing flavonoid pinocembrin (**3**) and the iridoid catalpol (**4**) are endowed with antioxidant^[13]^ and neuritogenic activity,^[14]^ respectively (Figure 2 a). In addition, an extensive substructure search in the Dictionary of Natural Products revealed that 2‐pyridones and dihydropyrans are rarely combined in natural products, and if so, only in particular arrangements (see the Supporting Information, Figure S1).

For the fusion of the pyridone and dihydropyran fragments, we chose regioisomerically different bipodal^[6]^ edge‐fused connectivity patterns **A**–**C** (Figure 2 b), which do not occur in any natural product. In **A**–**C**, a linking dihydrofuran fragment with two stereocenters is established, which may be considered a NP fragment on its own^[15]^ (Figure 2 b). In addition, the syntheses yielded compound class **D**, representing a monopodal connection, which occurs only in a few NPs (see the Supporting Information, Figure S1).

**Scheme 1:**
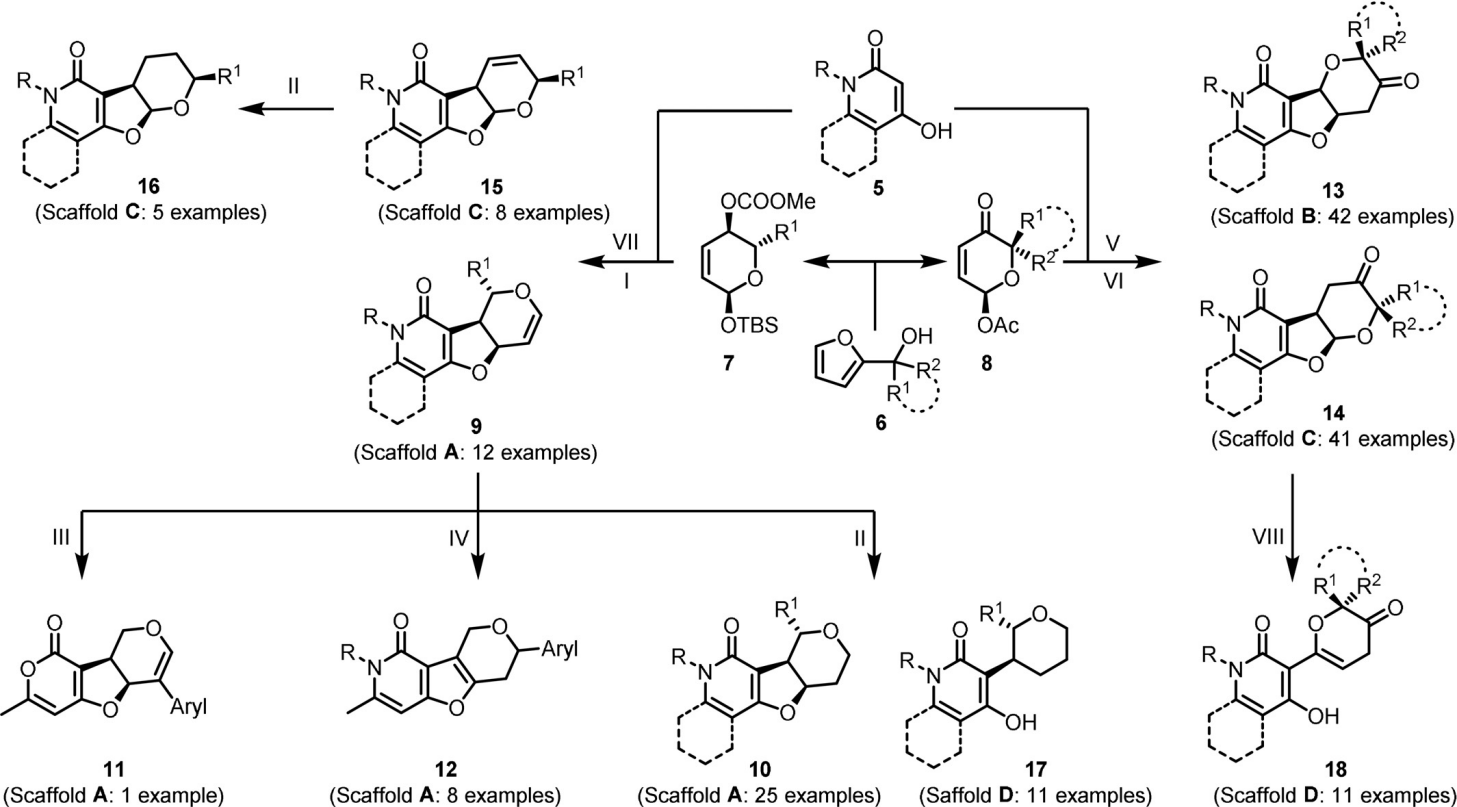
Synthesis of the PFP pseudo NP library; General reaction conditions: I) R=Me: Pd(PPh_3_)_4_, THF/DMF, rt, overnight, R=H: Pd[P[3,5‐(CF_3_)_2_C_6_H_3_]_3_]_3_, THF/DMF, 100–110 °C MW, 1–2 h; II) R=H, R=Me: Pd/C, H_2_, toluene, rt, 1 h–1 d; III) 1. NBS, AgNO_3_, acetonitrile, 80 °C, 2 h; 2. Aryl‐B(OH)_2_, NaO*t*Bu, Pd(OAc)_2_, Xphos, toluene, 130 °C MW, 40 min; IV) Aryl‐B(OH)_2_, Pd(OAc)_2_, DMF, rt, overnight; V) R^1^=R^2^=H, R^1^=R^2^=Me, R^1^=R^2^=*N*‐Boc piperidine, R^1^=H R^2^=Me: Pd(PPh_3_)_4_, NEt_3_, THF/DMF, rt, overnight; VI) R^1^=R^2^=H, R^1^=R^2^=Me, R^1^=R^2^=*N*‐Boc piperidine: quinine, DCM, 60 °C, 18 h; VII) R=Me: Pd[P[3,5‐(CF_3_)_2_C_6_H_3_]_3_]_3_, THF/DMF, 100–110 °C MW, 1–2 h; VIII) R^1^=R^2^=*N*‐Boc piperidine: HCl in dioxane, 0 °C, rt, 90 min.

The synthesis of the compound collection commenced with readily available bicyclic 4‐hydroxy‐2‐pyridones and 4‐hydroxy‐6‐methyl‐2‐pyridones **5** (Scheme 1), which were obtained from 4‐hydroxy‐6‐methyl‐2‐pyrone by treatment with various primary amines^[16]^ (see the Supporting Information). For the synthesis of functionalized dihydropyrans **7** and **8** (Scheme 1), furyl alcohols **6** were subjected to an Achmatowicz rearrangement^[17]^ to establish the six‐membered 3,6‐dihydro‐2*H*‐pyran core scaffold. Subsequent protection of anomeric alcohols with TBSCl, diastereoselective substrate‐controlled Luche‐reduction of the keto group and protection of the resulting secondary alcohols as carbonates yielded bis‐functionalized dihydropyran substrates **7** (see the Supporting Information, Scheme S1). Alternatively, Achmatowicz rearrangement products were only O‐acetylated at the acetal oxygen to afford mono‐functionalized dihydropyranones **8** (see the Supporting Information, Scheme S1).

Difunctionalized 3,6‐dihydro‐2*H*‐pyrans **7** were used as bis‐electrophiles in a palladium‐catalyzed allylic alkylation cascade^[18]^ with bis‐nucleophilic pyridones **5** to obtain PFPs **9** (general scaffold **A**, Scheme 1, I). We observed instability for some examples of **9** owing to a Ferrier‐like rearrangement^[19]^ of the glycal moiety and decided therefore to perform heterogenous reduction of the endocyclic double bond to afford isomers **10** (Scheme 1, II) or exploit the glycal reactivity for further derivatization. For instance, regioselective bromination of the glycal double‐bond^[20]^ and subsequent Suzuki–Miyaura coupling with an electron withdrawing 4‐(trifluoromethyl)phenylboronic acid gave compound **11** (Scheme 1, III). Furthermore, a direct palladium(II)‐catalyzed *C*‐glycosidation^[21]^ of **9** with aryl boronic acids and subsequent isomerization of the pyran double bond into the dihydrofuran ring yielded compounds **12** (Scheme 1, IV). A Tsuji–Trost alkylation oxa‐Michael addition cascade^[22]^ employing bis‐nucleophilic 4‐hydroxy‐2‐pyridones **5** and acetylated dihydropyranone bis‐electrophiles **8** gave PFPs **13**, which resemble the general scaffold **B** (Scheme 1, V). The corresponding regio‐isomers **14** (general scaffold **C**, Scheme 1, VI) with inverted attachment points between the pyran fragment and the furopyridone fragment were synthesized by a quinine‐mediated Michael addition‐transacetalization cascade reaction.^[22]^ Analogues **15** (Scheme 1, VII) of cyclic acetals **14** in which the methylene‐carbonyl substructure is replaced by a double bond, and their reduced successors **16** (Scheme 1, II), were prepared by an unprecedented Tsuji–Trost alkylation transacetalization reaction sequence. In addition, monopodal connection isomers **17** and **18** (Scheme 1, II and VIII) were synthesized and employed in the biological analyses. In total, a collection of 162 (+/−)‐PFPs were synthesized in sufficient amounts for biological testing. In general, the compounds were purified chromatographically (purity >95 %) before they were subjected to biological investigations.

To analyze the chemical space occupied by the synthesized 162 PFPs, first the NP‐likeness of the bipodal‐fused combinations was evaluated employing the NP‐likeness score introduced by Ertl et al. as a metric tool^[23]^ and compared to NP‐scores calculated for molecules listed in DrugBank^[24]^ and the NP set in the ChEMBL database. The range of PFP NP scores is in an area of the graph that is only sparsely covered by NPs (Figure 2 c), which reflects that the novel fragment combinations realized in these bipodal‐fused pseudo NPs do not exist in NP scaffolds. In contrast, the NP‐scores calculated for drugs and closely related molecules show substantial overlap with the majority of the PFP NP‐scores, indicating that PFPs may have favorable physicochemical properties for potential drug discovery programs. Structural relatedness was further evaluated by mapping estimated hydrophobicity (ALogP) against the respective molecular weight (MW) using the open‐source software LLAMA (Figure 2 d).^[25]^ The vast majority of pseudo NPs (98 %, 158 out of 162 PFPs) fall into the Lipinski's rule‐of‐five space^[26]^ exhibiting an A Log *P*<5 and MW<500 Da. In addition, 61 % of the compounds (99 out of 162 PFPs) fall even into the lead‐like space, which defines a preferable fraction of chemical space where A Log *P*<3 and MW<350 Da.^[27]^ Analysis of the three‐dimensional character of the collection and visualization of the shape distribution in a principal moments of inertia (PMI) plot (Figure 2 e)^[28]^ revealed a shift from the linear/disc‐like axes towards a spherical shape for the pseudo NPs indicating a three‐dimensional character compared to commercially available compound collections.^[25]^ This observation further shows that the molecular shape diversity of NPs is conserved in the fragment recombination process.

Collectively, these data suggest that the combination of biosynthetically rarely related pyridones and dihydropyrans generated novel PFP scaffolds with advantageous physicochemical properties, increased molecular shape diversity, and NP‐score distributions not represented by NPs. This observation mirrors that PFPs are not accessible by biosynthesis such that these pseudo NPs define a distinct chemotype that is more than merely the sum of its fragments eventually representing a distinct area of chemical space. Other NP‐fragment combinations to distinct classes of pseudo NPs were previously reported to follow a similar trend in the distribution of NP‐scores.[^6^, ^29^]

Given the unprecedented structure of the pseudo NPs, their bioactivities may be unexpected and may differ widely from the activities displayed by the guiding NPs. Therefore, similar to the biological evaluation of newly discovered NPs, they should be investigated in multiple individual bioassays covering a wide spectrum of biology. Alternatively, pseudo NPs may be more readily and conclusively characterized by target‐agnostic phenotyping approaches based on high‐content technologies^[9]^ because such phenotypic profiling may efficiently cover larger areas of biological space in one experimental approach. In agreement with this notion, we assessed the bioactivity of the PFP pseudo NPs in a cell painting assay.[^9b^, ^30^] In this multiplex assay, small molecule dyes are employed to selectively stain different cellular compartments in the absence and presence of compounds. Subsequently, high‐content imaging is performed, and automated image analysis extracts and quantifies hundreds of morphological features, which are arrayed in a fingerprint pattern that characterizes the bioactivity of a compound. A comparison of the obtained morphological fingerprints with the patterns for reference compounds with annotated bioactivity may then be employed to generate hypotheses for compound targets or mode of action and guide subsequent experimental target identification and validation efforts.^[9]^

Morphological fingerprints for a total of 579 parameters (see the Supporting Information for the delineation of the parameters) were determined for all PFP pseudo NPs^[30d]^ at 10, 30, and 50 μm and for 3000 reference compounds (mostly at 10 μm) including bioactive small molecules generated in house (see the Supporting Information for details). For assessment of bioactivity similarity of fingerprint profiles (“biological similarity”, BioSim; see the Supporting Information for determination of similarity) was employed and in addition an “induction” value (the fraction of parameters (in %) that underwent significant changes (median absolute deviation (MAD) value upon compound treatment of at least +/− three‐fold of the median determined for the DMSO controls; see the Supporting Information) was determined as measure for compound bioactivity. This decision was based on the observation that for the pseudo NP class analyzed in this study, induction by bioactive members increased with concentration (see below).

For the identification of compounds with probably most interesting bioactivity, all PFPs that, at 10 μm, showed an induction of at least 10 % (bioactivity of compounds with lower induction is most likely only low) and not more than 80 % (compounds with higher induction might have multiple targets or pleiotropic activity) were considered. This analysis afforded five initial hits (see the Supporting Information, Table S1). All these hits exhibited a fingerprint profile similarity of more than 70 % to at least one of the reference compound profiles, thus enabling delineation of biological mode of action or targets.

**Figure 3:**
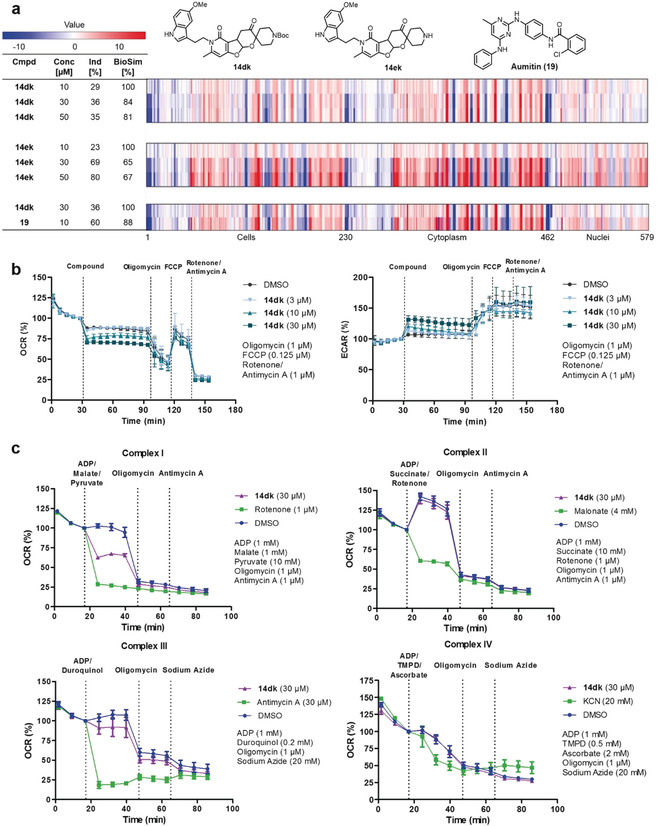
a) Concentration‐dependent fingerprint comparison of **14 dk** and **14 ek** (see Table S4, entries 3 and 13) as well as fingerprint comparison between **14 dk** and Aumitin (**19**). The top line in each bar graph is set as a reference fingerprint (100 % BioSim) to which subjacent fingerprints are compared. Blue indicates a decrease of a specific parameter compared to DMSO control; red indicates an increase of a specific parameter compared to DMSO control. b) Influence of compound **14 dk** on the mitochondrial respiration. HeLa cells were treated with compound and the oxygen consumption rate (OCR) and extracellular acidification rate (ECAR) were measured with a Seahorse XFp analyzer. Control inhibitors were added successively to the samples. Data is mean ± SD, *n*=3. c) Mitochondrial complex inhibition assay. HeLa cells were permeabilized by Seahorse XF plasma membrane permeabilizer and treated with **14 dk** and the respective substrates of the individual complexes. Control inhibitors were added successively to the samples. Data is mean ± SD, *n*=3.

The obtained hits were clustered according to biological similarity within the hit group itself to identify compounds with highest potency (i.e., induction) at high biological similarity of at least 80 % between two entries (see the Supporting Information for the clustering procedure and Table S1). The cluster with the pseudo NPs that displayed the highest induction value at 10 μm contained two members, that is, Boc‐protected PFP **14 dk** (Figure 3 a) and the deprotected analogue **14 ek** (Figure 3 a). **14 dk** and **14 ek** showed biological similarity of 84 % and were structurally similar (Tanimoto coefficient: 0.72; Table S1). The relatively high biological and chemical similarity suggest similar modes of action, and these compounds were characterized further.

To validate and justify the choice of induction as a measure for compound bioactivity, the concentration dependencies of the phenotypes for **14 dk** and **14 ek** (Figure 3 a) as representative members of the PFP pseudo NPs were determined by comparing the fingerprints at 10, 30, and 50 μm. For PFP **14 dk**, induction increased from 10 to 30 μm and remained constant at 50 μm, with biological similarity remaining high (>80 %) at all concentrations. For **14 ek**, induction increased with concentration while biological similarity remained at an average of 66 %. Thus, for **14 dk** and **14 ek** induction increased with concentration, while the fingerprint shape remained comparable with concentration‐dependent change of induction, such that induction can be employed as a measure for bioactivity. The lower profile similarity observed for **14 ek** at 10 versus 30 or 50 μm probably reflects the lower bioactivity of **14 ek** as compared to **14 dk**.

For identification of potential mode of action, a cross‐correlation analysis for profiles determined for **14 dk** and **14 ek** at 10, 30, and 50 μm, and the set of commonly found reference compounds at all measured concentrations was performed to generate a cross‐correlation matrix (see the Supporting Information, Table S3).

For both compounds at 30 and 50 μm, high biosimilarities (>83 %) were found to reference compounds that inhibit mitochondrial respiration, autophagy, glucose uptake as well as Wnt‐ and Hedgehog pathway signaling.[^6^, ^31^] Subsequent investigation in cell‐based assays excluded autophagy and Hedgehog signaling as well as glucose uptake inhibition as potential modes of action. However, we noted that these developmental and metabolism networks are involved in the regulation of reactive oxygen species (ROS) formation.^[32]^ In addition, the fingerprint determined for PFP **14 dk** at 30 μm showed 88 % similarity to the fingerprint determined for aumitin (**19**, Figure 3 a), a known inhibitor of mitochondrial respiration by targeting mitochondrial complex I.^[31d]^

Since these results suggested that pseudo NP **14 dk** may modulate mitochondrial function as well, a Mito Stress Test assay was carried out employing the Seahorse XF analyzer as previously reported.^[33]^ This assay enabled the evaluation of the effect of **14 dk** on oxygen consumption rate (OCR) and extracellular acidification rate (ECAR) reflecting the rates of mitochondrial respiration and glycolysis, respectively (Figure 3 b). At 10 and 30 μm, compound **14 dk** rapidly induced partial inhibition of mitochondrial respiration, which the cells counterbalanced by increasing their glycolysis rate. Given the fact that inhibition of mitochondrial complexes I and III induces formation of superoxide,^[34]^ we assayed formation of mitochondrial superoxide employing the fluorogenic indicator MitoSOX Red.^[31d]^ Pseudo NPs **14 dk** and **14 ek** induced concentration‐dependent mitochondrial superoxide formation in HeLa cells after 1 h incubation with EC_50_ values of 3.7±0.9 μm and 10.7±3.6 μm, respectively (see the Supporting Information, Table S4 entries 3 and 13).

In the context of formation of reactive oxygen species (ROS), it has been reported that ROS is involved in aging and pathogenesis of several diseases.^[35]^ Furthermore, induction of ROS formation is considered a potential opportunity for the development of new cancer therapies through the selective enhancement of cancer‐cell cytotoxicity.^[36]^

To further validate the hypothesis of complex I or III inhibition by pseudo NP **14 dk**, the previously reported semi‐intact assay for mitochondrial respiration was performed.^[37]^ This assay enables the individual investigation of either complex I‐, II‐, III‐, or IV‐mediated respiratory activity by permeabilizing the cells with a Seahorse XF Plasma Membrane Permeabilizer (PMP) and adding distinct substrates required for activity of the respective complexes (Figure 3 c).^[37]^ At 30 μm, compound **14 dk** partially inhibited complex I as compared to a full inhibition by the known complex I inhibitor rotenone^[38]^ at 1 μm, while complex II and IV were not affected by **14 dk**. For complex III, a potentially weak inhibition could not be excluded as a slight decrease in OCR compared to the DMSO control was observed upon addition of **14 dk**. While molecules exhibiting dual inhibitory activity on complex I and III were already described in the literature,^[39]^ the structural elements of **14 dk** and its fragments do not resemble any of the three major classes of complex I inhibitors or reported complex III inhibitors.^[40]^

These results demonstrate that target agnostic multiparametric phenotypic profiling, as implemented in the cell painting assay, may enable determination of mode of action of pseudo NPs and, by analogy, other bioactive small molecules.^[41]^

Beyond the identification of mode of action, we analyzed the cell painting data to determine whether this multiparametric phenotypic assay may guide identification of qualitative trends in structure–phenotype relationship (SPR). Subsequently, we assessed if the derived trends in SPR correlate with trends in structure–activity relationship (SAR) determined by means of the MitoSOX Red assay. These analyses could potentially inform hit expansion through synthesis of additional compounds. To this end, all pseudo NPs with induction >10 % and <80 % at all concentrations (10, 30, and 50 μm) were clustered, yielding a 16‐membered cluster of structurally related compounds with biosimilarities >82 % (see the Supporting Information, Table S2). Since **14 dk** displayed the highest induction at 10 μm and is a member of this cluster, its fingerprint profile determined at 10 μm was set as a reference phenotype to which all other cluster profiles and fingerprints of structurally related compounds were compared. To assure that the comparison is not negatively influenced by larger differences in fingerprint richness, comparison was made in an induction range of 20–40 % (see the Supporting Information, Table S4). Compounds representing the general trends in SPR were then subjected to EC_50_ determination with the MitoSOX Red assay (see the Supporting Information, Table S4).

Comparison of the induction values as measure for bioactivity (see above) revealed that variation of the pyridone *N*‐substituent at retained bioactivity is possible, but that the substituent should not be too small (see the Supporting Information, Table S4, compare entries 1–7) or too polar (Table S4, entries 8–10) to establish higher induction values. Thus, replacement of the methoxy‐indole (Table S4, entry 3) by different substituted phenyl groups or a thiophene led to high induction at comparable profile similarities (Table S4, compare entry 3 with entries 4–7). An isopentyl substituent also resulted in a high induction value, but if a methyl group was introduced, bioactivity was lost (Table S4, entries 1 and 2). Replacement of the lipophilic phenyl rings by a basic pyridine (Table S4, entries 8 and 9) abrogated induction, but if a chlorine was placed next to the basic nitrogen atom (thereby reducing basicity), bioactivity and biosimilarity were reestablished (Table S4, entry 10). These observations indicate that for R^1^ a lipophilic residue at a distance to the fused ring system is advantageous.

For R^2^, a bipodal‐fused and disubstituted cyclic acetal was beneficial for induction at high biological similarity to **14 dk** (Table S4, compare entries 11, 12, and 16 with entries 13 and 14). In addition, an α,α‐disubstituted ketone substructure with the quaternary carbon next to the acetal was required for activity (Table S4, compare entries 13 and 14 with entries 15 and 16).

Fragments of **14 dk** (Table S5, entries 1–3) were either not active or had low biological similarity to **14 dk**. In addition, the central cyclic acetal structure **C** seemed to be important for activity as compared to scaffold types **A**, **B**, and **D**. This trend was also apparent in a statistical activity analysis of all substructure classes (see the Supporting Information, Figure S2). However, the central cyclic acetal scaffold alone did not suffice to establish the observed kind of bioactivity. Compound **14 aa** (Table S4, entry 17), representing the core scaffold, exhibited almost no induction even at the highest measured concentration.

The EC_50_ values determined in the MitoSOX Red assay qualitatively parallel the SPR conclusions detailed above. Thus, compound **14 dk** (Table S4, entry 3) was the most potent PFP pseudo NP with an EC_50_ value of 3.7±0.9 μm, and most of the compounds with induction >20 % and profile similarity >80 % to **14 dk** induced ROS formation (Table S4, entries 4–7, 10, and 13). However, there were some notable exceptions (Table S4 entries 2, 11, and 14), indicating that, not surprisingly, the SPR‐SAR correlation is not fully parallel. While compound **14 dc** (Table S4, entry 2) must be regarded as a true outlier exhibiting activity in the cell painting assay with high bio similarity to **14 dk** but not in the MitoSOX Red assay, **14 cf** (Table S4, entry 14) elicited induction but with a profile similarity of 62 % to **14 dk** indicating that the morphological changes might be induced through a different mode of action. Compound **13 ah** (Table S4, entry 11) may be considered a borderline case since induction is 15 %, i.e. close to the 20 % cut‐off.

Consistent with the SPR, the fragments of **14 dk** were not active in the MitoSOX Red assay. Notably, fragment **8 d** (Table S5, entry 2) deviated from this trend. However, this compound induced decreased cell count and cell toxicity at all measured concentrations in the cell painting assay (see the Supporting Information, Figures S6–8).

Noteworthy, the fingerprints for PFP pseudo NPs could not be simulated by the mathematical addition of cell painting derived fingerprints of their respective fragments (see the Supporting Information, Figure S9). This shows that the synthetic combination of NP‐fragments generates pseudo NPs with properties that are more than merely the sum of topologic characteristics of the individual fragments.

## Conclusions

We have designed and developed a synthesis for a new pseudo natural product (pseudo‐NP) class, the pyrano‐furo‐pyridones (PFPs), that combines two biosynthetically rarely related natural product fragments in three different regioisomeric arrangements to arrive at structurally unprecedented, and therefore biosynthetically not accessed chemical matter. These pseudo NPs exhibit favorable drug‐like features and occupy an area of NP‐like chemical space different from NPs. Unbiased morphological profiling of PFPs in the cell painting assay and subsequent analysis, based on comparison of phenotypic fingerprints as compared to the fingerprints determined for annotated reference compounds, guided the discovery that the PFPs define a structurally novel class of mitochondrial superoxide formation inducers. Analysis of the morphological profiling data, employing the induction parameter as a qualitative measure for bioactivity, allowed us to establish general trends in SPR. These could be validated by determining EC_50_ values for structurally related compounds in a MitoSOX Red assay. The observed bioactivity was traced to inhibition of mitochondrial complex I as at least one responsible molecular target for the activity of the most potent compound.

Our results provide a proof of principle for the validity of the pseudo NP concept for the de novo design and synthesis of novel biologically relevant compound classes. Furthermore, they demonstrate the validity of phenotypic profiling, as exemplified by the cell painting assay, as a method to determine and characterize potential bioactivity of novel compound classes in a general sense. Beyond phenotypic profiling, the PFP pseudo‐natural product collection was investigated in individual assays monitoring signaling through the Wnt‐ and hedgehog pathways, inhibition of autophagy, indoleamine‐2,3‐dioxygenase and histone deacetylase SIRT‐1, as well as glucose transport by the GLUT1‐4 transporters. However, in none of these assays was substantial activity recorded at 10 μm, which could justify deeper investigation. These findings further highlight the value of pseudo NP profiling in unbiased morphological assays like cell painting, monitoring a wide range of biological processes simultaneously.

We note that qualitative structure–activity trends, as established herein, may not be fully representative of quantitative SAR analyses. In particular, compounds at an early hit stage as is certainly the case for the PFPs discussed in this study, may have multiple targets, and these may differ among the library members, such that caution is indicated not to over‐interpret the data obtained by the phenotypic analysis. However, if the phenotypic data follow a trend, as is the case for the PFPs as well, they may guide hit expansion into more potent compounds. They may also inform structural analysis, for instance, with respect to identification of a subsite in the compounds suitable for attachment of reporter groups and linkers, which will enable subsequent true target identification efforts. The different kinds of information that can be obtained from phenotypic profiling, as shown above and beyond our analysis, will be particularly valuable in cases in which target identification efforts following established methods frequently fail, for instance if the target protein is expressed only in a very low level, if it is a membrane protein, or if both conditions coincide.

## Conflict of interest

The authors declare no conflict of interest.

## Supporting information

As a service to our authors and readers, this journal provides supporting information supplied by the authors. Such materials are peer reviewed and may be re‐organized for online delivery, but are not copy‐edited or typeset. Technical support issues arising from supporting information (other than missing files) should be addressed to the authors.

Supplementary
