# Supplementary material for: Design, Synthesis, and Phenotypic Profiling of Pyrano‐Furo‐Pyridone Pseudo Natural Products
Source: Angew Chem Int Ed Engl. 2019 Aug 28;58(41):14715–23. doi: 10.1002/anie.201907853 (PMC7687248; doi:10.1002/anie.201907853)
Supplement: Supplementary file 1 — Supplementary [file ANIE-58-14715-s001.pdf]

Angewandte Chemie
Eine Zeitschrift der Gesellschaft Deutscher Chemiker

Supporting Information

# Design, Synthesis, and Phenotypic Profiling of Pyrano-Furo-Pyridone Pseudo Natural Products

*Andreas Christoforow, Julian Wilke, Aylin Binici, Axel Pahl, Claude Ostermann, Sonja Sievers, and Herbert Waldmann**

anie_201907853_sm_miscellaneous_information.pdf

**Supporting Information – Pyrano-Furo-Pyridones**


1. Chemistry ..... 2
   1.1. Synthesis of Pyridone Fragments ..... 3
   1.2. Synthesis of Functionalized Dihydropyrane Fragments ..... 8
   1.3. Synthesis of Pyrano-Furo-Pyridones ..... 16
   1.3.1. General Procedures ..... 25
   1.3.2. Synthesis of General Scaffold A Derivatives ..... 27
   1.3.3. Synthesis of General Scaffold B Derivatives ..... 54
   1.3.4. Synthesis of General Scaffold C Derivatives ..... 78
2. Substructure Search in DNP ..... 102
3. Biology ..... 103
   3.1. Cell-Painting Assay ..... 103
   3.1.1. Clustering ..... 105
   3.1.2. Cross-Correlation Matrix ..... 107
   3.1.3. Statistical Analysis of Structure-Induction-Relationship ..... 108
   3.1.4. Representative Cell-Painting Microscopy Images ..... 108
   3.1.5. Additivity of Profiles ..... 111
   3.2. Cell Culture ..... 111
   3.3. Cell Mito Stress Test ..... 112
   3.4. Semi-Intact Assay for Mitochondrial Respiration ..... 112
   3.5. MitoSOX Red Assay ..... 113
   3.6. Structure-Phenotype and Structure-Activity Relationship Studies ..... 114
4. Representative NMR Spectra ..... 117
5. References ..... 135

## 1. Chemistry

### *General*

All reactions were performed in oven dried glassware and under inert Argon atmosphere if not indicated differently. Dry solvents were purchased from Fischer Scientific and/or Acros and used without further treatment. Oxygen and/or moisture sensitive solutions were transferred using syringes and cannulas.

Thin layer chromatography (TLC) was performed on silica coated aluminium plates (Merck 60 $F_{254}$) and visualization was achieved under UV irradiation (254 nm), potassium permanganate stain (1.5 g KMnO4, 10 g $K_2CO_3$, 1.25 mL of 10% aqueous NaOH solution and 200 mL of water) or *p*-anisaldehyde stain (0.7 mL *p*-anisaldehyde, 9.5 mL conc. $H_2SO_4$, 2.7 mL of acetic acid and 250 mL of EtOH).

Analytical UHPLC-MS and LC-MS was performed on an Agilent 1290 Infinity system equipped with a mass detector (column: Zorbax Eclipse C18 Rapid Resolution 2.1x50 mm 1.8µm) and on a Thermo Scientific fleet station (column: Nuleodur C18 gravity EC 50/3, 1.8 µm). Appropriate gradient systems were applied by mixing Water (+ 0.1% TFA) and Acetonitrile (+ 0.1%).

Purification of crude products was achieved through flash column chromatography (FC, silica gel 60, 0.035-0.070 mm) or automated medium pressure liquid chromatography (MPLC, Grace Reveleris X2) using the indicated solvents. Challenging separations were carried out on an Agilent 1100 prep.arative HPLC system equipped with a mass detector (columns: Nuleodur C18 gravity VP 125/10 5 µm, Nucleodur C18 gravity VP 125/21 5 µm, Nucleodur C4 gravity VP 125/10 5 µm). Appropriate gradient systems were applied by mixing Water (+ 0.1% TFA) and Acetonitrile (+ 0.1%).

NMR spectra were recorded on Bruker AV 400 Avance III HD (NanoBay), Agilent Technologies DD2, Bruker AV 500 Avance III HD (Prodigy), Bruker AV 600 Avance III HD (CryoProbe) or Bruker AV 700 Avance III HD (CryoProbe) spectrometers. Data is reported in ppm with reference to the used deuterated solvent ($CDCl_3$: 7.26 ppm, 77.16 ppm; DMSO-$d_6$: 2.50 ppm, 39.52 ppm; $CD_2Cl_2$: 5.32 ppm, 53.84 ppm; MeOH-$d_4$: 3.31 ppm, 49.00 ppm; Acetone-$d_6$: 2.05 ppm, 29.84 ppm, 206.26 ppm).[1] Signals were assigned to their corresponding Hydrogens or Carbons based on 2D NMR correlations ($^1$H/$^1$H COSY, $^1$H/$^1$H NOESY, $^1$H/$^{13}$C HSQC, $^1$H/$^{13}$C HMBC).

High-resolution mass spectrometry (HRMS) was performed on an LTQ Orbitrap mass spectrometer coupled to an Accela HPLC-System (HPLC column: Hypersyl GOLD, 50 mm x 1 mm, particle size 1.9 µm, ionization method: electron spray ionization (ESI)).

Microwave reactions were carried out in a CEM Discover SP Activent machine.

Batch photoreactor consisted of a 250 mL or 500 mL schlenck flask, magnetic stirrer and two 34W blue LEDs (Kessil H150-Blue LED Lamp). The schlenck flask was placed in a dewar vessel of appropriate size. The dewar vessel was filled with iso-propanol and a constant flow of compressed air over the iso-propanol surface was adjusted. This cooling system maintained a bath temperature between 23-26 °C while irradiating the schlenck flask with two blue LEDs in a 45 °C angle.[2]

## 1.1. Synthesis of Pyridone Fragments

General procedure 1:[3]

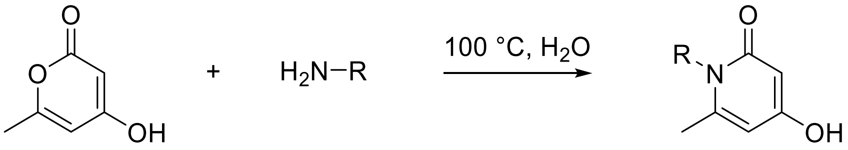


4-Hydroxy-6-mehtyl-pyrone (1.00 g, 7.93 mmol) was suspended in $H_2O$ (0.5 M) and amine (1 equiv) was added at room temperature. The mixture was stirred in a sealed vial at 100 °C overnight. Upon cooling to 0 °C a white precipitate formed which was filtered off and washed with cold $H_2O$. The remaining filter residue was triturated with EtOH and drying in vacuo afforded the corresponding pyridones as white to off-white solids.

**4-hydroxy-6-methylpyridin-2(1H)-one (5a)**[4]

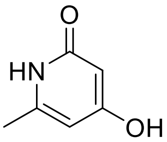


0.54 g, 54%. **$^1$H-NMR** (400 MHz, DMSO-d$_6$): δ 10.89 (s, 1H), 10.29 (s, 1H), 5.58 (dd, *J* = 2.2 Hz, 0.8 Hz, 1H), 5.32 (d, 2.2 Hz 1H), 2.07 (s, 3H). **LCMS**-ESI (m/z): 125.97 [M + H]$^+$.

**4-hydroxy-1,6-dimethylpyridin-2(1H)-one (5b)**[5]

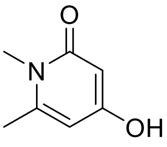


0.67 g, 61%. **$^1$H-NMR** (400 MHz, DMSO-d$_6$): δ 10.28 (s, 1H), 5.75 (dd, *J* = 2.2 Hz, 0.6 Hz, 1H), 5.48 (d, *J* = 2.2 Hz 1H), 3.29 (s, 3H), 2.26 (s, 3H). **LCMS**-ESI (m/z): 140.03 [M + H]$^+$.

**1-benzyl-4-hydroxy-6-methylpyridin-2(1H)-one (5c)**[4]

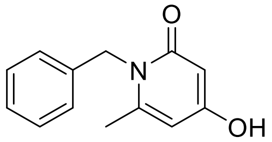

0.35 g, 62%. **$^1$H-NMR** (500 MHz, DMSO-d$_6$): δ 10.47 (s, 1H), 7.32 (t, *J* = 7.4 Hz, 2H), 7.24 (t, *J* = 7.4 Hz, 1H), 7.08 (d, *J* = 7.4 Hz, 2H), 5.79 (dd, 2.2 Hz, 1H), 5.59 (d, 2.2 Hz 1H), 5.18 (bs, 2H), 2.16 (s, 3H). **HRMS**-ESI (m/z): [M + H]$^+$ calculated for $C_{13}H_{14}O_2N^+$, 216.1019; found, 216.1017.

**4-hydroxy-1-(4-methoxybenzyl)-6-methylpyridin-2(1H)-one (5d)**[4]

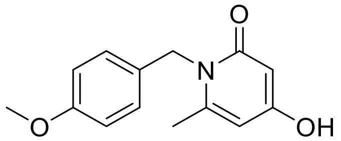

Using 500.0 mg (3.89 mmol) 4-Hydroxy-6-mehtyl-pyrone. Purification by MPLC (DCM/MeOH 1:0 to 4:1) afforded the desired product as a white solid (0.38 g, 40%). **$^1$H-NMR** (700 MHz, DMSO-d$_6$): δ 10.50 (s, 1H), 7.05 (d, *J* = 8.6 Hz, 2H), 6.87 (d, *J* = 8.6 Hz, 2H), 5.77 (d, 2.2 Hz, 1H), 5.59 (d, 2.2 Hz 1H), 5.11 (bs, 2H, CH$_2$), 3.71 (s, 1H), 2.17 (s, 3H). **$^{13}$C-NMR** (176 MHz, DMSO-d$_6$): δ 165.8, 164.1, 158.3, 147.6, 129.7, 127.6, 114.0, 100.4, 96.0, 55.1, 44.7, 20.0. **HRMS**-ESI (m/z): [M + H]$^+$ calculated for $C_{14}H_{16}O_3N^+$, 246.1125; found, 246.1123.

**cyclobutyl-4-hydroxy-6-methylpyridin-2(1H)-one (5e)**

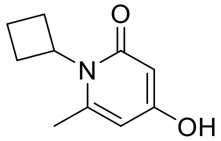

0.62 g, 56%. **$^1$H-NMR** (700 MHz, DMSO-d$_6$): 10.26 (s, 1H), 5.66 (d, *J* = 2.5 Hz, 1H), 5.40 (d, *J* = 2.5 Hz, 1H), 4.68 (p, *J* = 8.8 Hz, 1H), 3.12 (m, 2H), 2.26 (s, 3H), 2.13 – 2.07 (m, 2H), 1.77 (q, *J* = 10.3 Hz, 1H), 1.64 (dq, *J* = 18.4, 9.4 Hz, 1H). **$^{13}$C-NMR** (176 MHz, DMSO-d$_6$) δ 165.4, 165.1, 147.05, 100.5, 97.5, 51.2, 26.91, 20.9, 14.1. **HRMS**-ESI (m/z): [M + H]$^+$ calculated for $C_{10}H_{14}O_2N^+$, 180.1019; found, 180.1018.

**4-hydroxy-6-methyl-1-(4-morpholinophenyl)pyridin-2(1H)-one (5f)**

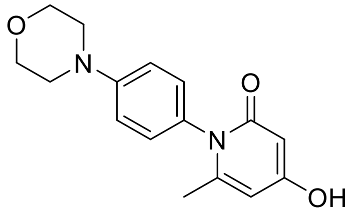

Using 500.0 mg (3.89 mmol) 4-Hydroxy-6-mehtyl-pyrone. 0.50 g, 45%. **$^1$H-NMR** (700 MHz, DMSO-d$_6$): δ 10.48 (s, 1H), 7.00 (s, 4H), 5.84 (d, *J* = 2.1 Hz, 1H), 5.52 (d, *J* = 2.1 Hz, 1H), 3.76 – 3.73 (m, 4H), 3.16 – 3.14 (m, 4H), 1.84 (s, 3H). **$^{13}$C-NMR** (176 MHz, DMSO-d$_6$): δ 166.6, 164.79, 150.9, 148.0, 130.4, 129.4, 115.6, 100.2, 96.5, 66.6, 48.6, 21.7. **HRMS**-ESI (m/z): [M + H]$^+$ calculated for $C_{16}H_{19}O_3N_2^+$, 287.1390; found, 287.1393.

**4-hydroxy-6-methyl-1-(tetrahydro-2H-pyran-4-yl)pyridin-2(1H)-one (5g)**

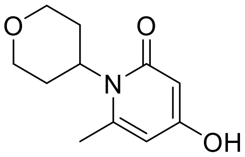


0.35 g, 22%. **$^{1}$H-NMR** (700 MHz, DMSO-d$_6$): δ 10.26 (s, 1H), 5.70 (bd, *J* = 1.6 Hz, 1H), 5.40 (s, 1H), 3.89 (dd, *J* = 11.5, 4.2 Hz, 2H), 3.35 (d, *J* = 11.5 Hz, 2H), 2.95 – 2.85 (bm, 2H), 2.32 (s, 3H), 1.44 (d, *J* = 11.5 Hz, 1H). **$^{13}$C-NMR** (176 MHz, DMSO-d$_6$): δ 165.2, 164.6, 147.2, 100.8, 97.9, 67.0, 52.0, 28.2, 21.0. **HRMS**-ESI (m/z): $[M + H]^+$ calculated for $C_{11}H_{16}O_3N^+$, 210.1125; found, 210.1126.

**4-hydroxy-1-(4-methoxyphenyl)-6-methylpyridin-2(1H)-one (5h)**[6]

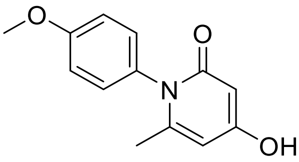


0.62 g, 35%. **$^{1}$H-NMR** (700 MHz, DMSO-d$_6$): δ 10.51 (s, 1H), 7.08 (d, *J* = 8.6 Hz, 2H), 7.01 (d, *J* = 8.6 Hz, 2H), 5.85 (s, 1H), 5.52 (s, 1H), 3.79 (s, 3H), 1.83 (s, 3H). **$^{13}$C-NMR** (176 MHz, DMSO-d$_6$): δ 166.3, 164.2, 158.7, 147.4, 131.5, 129.6, 114.3, 99.8, 96.0, 55.3, 20.8. **HRMS**-ESI (m/z): $[M + H]^+$ calculated for $C_{13}H_{14}O_3N^+$, 232.0895; found, 232.0969.

**4-hydroxy-6-methyl-1-(pyridin-4-ylmethyl)pyridin-2(1H)-one (5i)**[7]

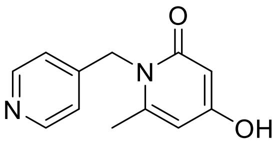


1.34 g, 80%. **$^{1}$H-NMR** (500 MHz, DMSO-d$_6$): δ 10.49 (bs, 1H), 8.49 (d, *J* = 6.0 Hz, 2H), 7.05 (d, *J* = 6.0 Hz, 2H), 5.84 (d, *J* = 2.3 Hz, 1H), 5.59 (d, *J* = 2.3 Hz, 1H), 5.20 (s, 2H), 2.14 (s, 3H). **$^{13}$C-NMR** (126 MHz, DMSO-d$_6$): δ 166.2, 163.9, 149.9, 147.4, 146.9, 121.2, 100.7, 95.8, 44.7, 19.9. **HRMS**-ESI (m/z): $[M + H]^+$ calculated for $C_{12}H_{13}O_2N_2^+$, 217.0972; found, 217.0970.

**4-hydroxy-6-methyl-1-(thiophen-2-ylmethyl)pyridin-2(1H)-one (5j)**

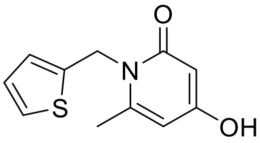


1.00 g, 60%. **$^{1}$H-NMR** (500 MHz, DMSO-d$_6$): δ 10.52 (s, 1H), 7.39 (dd, *J* = 5.1, 1.0 Hz, 1H), 7.03 (d, *J* = 3.1 Hz, 1H), 6.94 (dd, *J* = 5.1, 3.1 Hz, 1H), 5.76 (s, 1H), 5.56 (s, 1H), 5.26 (s, 2H), 2.32 (s, 3H). **$^{13}$C-NMR** (126 MHz, DMSO-d$_6$): δ 166.0, 163.6, 148.0, 140.0, 126.5, 126.5, 126.0, 100.5, 95.9, 41.2, 19.9. **HRMS**-ESI (m/z): $[M + H]^+$ calculated for $C_{11}H_{12}O_2NS^+$, 222.0583; found, 222.0582.

**1-(4-fluorobenzyl)-4-hydroxy-6-methylpyridin-2(1H)-one (5k)**[8]

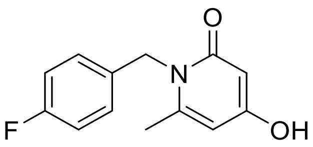


0.50 g**,** 28%. **$^{1}$H-NMR** (600 MHz, DMSO-d$_6$): δ 10.51 (s, 1H), 7.15 (d, *J* = 7.3 Hz, 4H), 5.79 (d, *J* = 2.3Hz, 1H), 5.59 (d, *J* = 2.3 Hz, 1H), 5.16 Hz (s, 2H), 2.17 (s, 3H). **$^{13}$C-NMR** (151 MHz, DMSO-d$_6$): δ 165.9, 164.0, 162.0, 160.3, 147.5, 133.9, 128.3, 128.2, 115.4, 115.3, 100.5, 95.9, 44.7, 19.9. **HRMS**-ESI (m/z): $[M + H]^+$ calculated for $C_{13}H_{13}O_2NF^+$, 234.0925; found, 234.0920.

**1-(2-chlorobenzyl)-4-hydroxy-6-methylpyridin-2(1H)-one (5l)**[9]

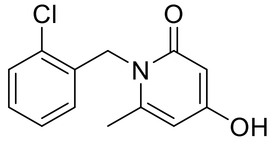


0.47 g, 24%. $^{1}$**H-NMR** (500 MHz, DMSO-d$_6$): δ 10.60 (s, 1H), 7.50 (dd, *J* = 7.2, 2.0 Hz, 1H), 7.32-7.26 (m, 2H), 6.59 (dd, *J* = 7.1, 2.2 Hz, 1H), 5.88 (d, *J* = 2.6 Hz, 1H), 5.60 (d, *J* = 2.6 Hz, 1H), 5.17 (s, 2H), 2.13 (s, 3H). $^{13}$**C-NMR** (126MHz, DMSO-d$_6$): δ 166.2, 163.7, 147.5, 134.7, 131.3, 129.4, 128.7, 127.7, 125.9, 100.8, 95.8, 43.8, 19.8. **HRMS**-ESI (m/z): $[M + H]^+$ calculated for $C_{13}H_{13}O_2NCl^+$, 250.0629; found, 250.0631.

**4-hydroxy-6-methyl-1-(pyridin-2-ylmethyl)pyridin-2(1H)-one) (5m)**[7]

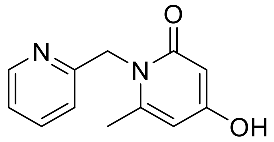


1.09 g, 64%. $^{1}$**H-NMR** (600 MHz, DMSO-d$_6$): δ 10.46 (s, 1H), 8.48 (ddd, *J* = 4.9, 1.9, 0.9 Hz, 1H), 7.74 (td, *J* = 7.8, 7.6, 1.9 Hz, 1H), 7.26 (ddd, *J* = 7.6, 4.9, 1.2 Hz, 1H), 7.10 (d, *J* = 7.8 Hz, 1H), 5.81 (dd, *J* = 2.6, 1.0 Hz, 1H), 5.53 (d, *J* = 2.6 Hz, 1H), 3.34 (s, 2H), 2.23 (s, 3H). $^{13}$**C-NMR** (151 MHz, DMSO-d$_6$): δ 165.9, 163.8, 156.9, 149.0, 147.9, 136.9, 122.3, 120.9, 100.3, 95.8, 47.4, 39.9, 39.8, 39.7, 39.5, 39.4, 39.2, 39.1, 20.2. **HRMS**-ESI (m/z): $[M + H]^+$ calculated for $C_{12}H_{13}O_2N_2^+$, 217.0972; found, 217.0972.

**1-(3,5-dimethylbenzyl)-4-hydroxy-6-methylpyridin-2(1H)-one (5n)**

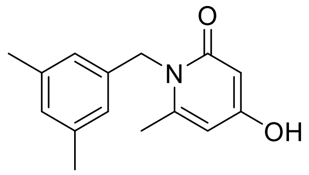


0.18 g, 9%. $^{1}$**H-NMR** (500 MHz, MeOH-d$_4$-d$_4$): δ 6.90 (s, 1H), 6.70 (s, 2H), 5.97 (d, *J* = 6.3 Hz, 1H), 5.82 (d, *J* = 2.6 Hz, 1H), 5.25 (s, 2H), 2.25 (s, 6H), 2.24 (s, 3H). **HRMS**-ESI (m/z): $[M + H]^+$ calculated for $C_{15}H_{18}O_2N^+$, 244.1332; found, 244.1331.

**1-(2-(1H-imidazol-4-yl)ethyl)-4-hydroxy-6-methylpyridin-2(1H)-one (5o)**[8]

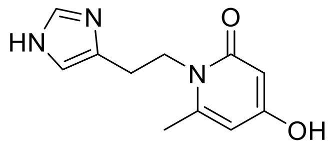


Using 500.0 mg (3.89 mmol) 4-Hydroxy-6-mehtyl-pyrone. 0.56 g, 66%. $^{1}$**H-NMR** (400 MHz, DMSO-d$_6$): δ 11.85 (s, 1H), 10.39 (s, 1H), 7.55 (s, 1H), 6.78 (s, 1H), 5.69 (d, *J* = 2.6 Hz, 1H), 5.50 (d , *J* = 2.6 Hz, 1H), 4.01 (dd, *J* = 8.25, 7.0 Hz, 2H), 2.74 (dd, *J* = 8.25, 7.0 Hz, 2H), 2.17 (s, 3H). $^{13}$**C-NMR** (100 MHz, DMSO-d$_6$): δ 165.6, 163.6, 147.3, 135.1, 134.9, 134.2, 100.0, 96.0, 43.3, 26.0, 19.8. **HRMS**-ESI (m/z): $[M + H]^+$ calculated for $C_{11}H_{14}O_2N_3^+$, 220.1081; found, 220.1076.

**4-hydroxy-1-(2-(5-methoxy-1H-indol-3-yl)ethyl)-6-methylpyridin-2(1H)-one (5p)**

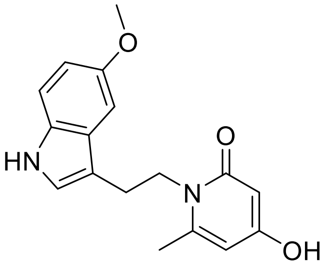


Using 500.0 mg (3.89 mmol) 4-Hydroxy-6-mehtyl-pyrone. 0.41 g, 36%. **$^1$H-NMR** (400 MHz, DMSO-d$_6$): δ 10.69 (s, 1H), 10.33 (s, 1H), 7.22 (d, *J* = 8.7 Hz, 1H), 7.10 (dd, *J* = 5.8, 2.5 Hz, 2H), 6.70 (dd, *J* = 8.7, 2.5 Hz, 1H), 5.68 (d, *J* = 2.6 Hz, 1H), 5.56 (d, *J* = 2.6 Hz, 1H), 4.10 – 4.02 (m, 2H), 3.74 (s, 3H), 2.94 – 2.89 (m, 2H), 2.16 (s, 3H). **$^{13}$C-NMR** (100 MHz, DMSO-d$_6$): δ 165.5, 163.8, 153.0, 147.3, 131.3, 127.6, 123.6, 112.0, 111.2, 111.0, 100.1. 99.9, 96.1, 55.2, 44.0, 24.1, 19.8. **HRMS**-ESI (m/z): [M + H]$^+$ calculated for $C_{17}H_{19}O_3N_2^+$, 299.1390; found, 299.1389.

**4-hydroxy-6-methyl-1-((3-methylpyridin-4-yl)methyl)pyridin-2(1H)-one (5q)**

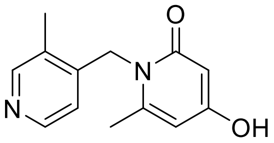


0.73 g, 41%. **$^1$H-NMR** (700 MHz, DMSO-d$_6$): δ 10.6 (s, 1H), 8.37 (s, 1H), 8.29 (d, *J* = 5.0 Hz, 1H), 6.42 (d, *J* = 5.0 Hz, 1H), 5.88 (d, *J* = 2.6 Hz, 1H), 5.60 (d, *J* = 2.6 Hz, 1H), 5.13 (s, 2H), 2.33 (s, 3H), 2.11 (s, 3H). **$^{13}$C-NMR** (176 MHz, DMSO-d$_6$): δ 166.1, 163.7, 150.1, 147.7, 147.4, 144.8, 130.4, 118.4, 100.7, 95.8, 43.0, 19.6, 15.3. **HRMS**-ESI (m/z): [M + H]$^+$ calculated for $C_{13}H_{15}O_2N_2^+$, 231.1128; found, 231.1124.

**1-((2-chloropyridin-4-yl)methyl)-4-hydroxy-6-methylpyridin-2(1H)-one (5r)**

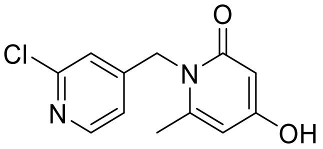


0.90 g, 51%. **$^1$H-NMR** (500 MHz, DMSO-d$_6$): δ 10.65 (s, 1H), 8.34 (d, *J* = 5.1 Hz, 1H), 7.17 (s, 1H), 7.07 (d, *J* = 5.1 Hz, 1H), 5.86 (d, *J* = 2.2 Hz, 1H), 5.59 (d, *J* = 2.2 Hz, 1H), 5.20 (s, 2H), 2.16 (s, 3H). **$^{13}$C-NMR** (126 MHz, DMSO-d$_6$): δ 166.3, 163.8, 151.2, 150.6, 150.3, 147.4, 121.5, 120.7, 100.9, 95.8, 44.6, 20.0. **HRMS**-ESI (m/z): [M + H]$^+$ calculated for $C_{112}H_{12}O_2N_2Cl^+$, 251.0582; found, 251.0581.

**4-hydroxy-6-methyl-1-((tetrahydrofuran-2-yl)methyl)pyridin-2(1H)-one (5s)[9]**

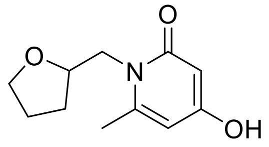


1.29 g, 80%. **$^1$H-NMR** (700 MHz, DMSO-d$_6$): δ 10.36 (s, 1H), 5.72 (s, 1H), 5.48 (s, 1H), 4.08 (dd, *J* = 13.9, 3.2 Hz, 1H ), 4.05-4.01 (m, 1H), 3.75 (ddd, *J* = 8.3, 7.2, 6.2 Hz, 1H), 3.67 (dd, *J* = 13.9, 8.3 Hz, 1H), 3.59 (td, *J* = 7.8, 6.1 Hz, 1H), 2.31 (s, 3H), 1.93 (dddd, *J* = 12.2, 8.5, 6.9, 5.2 Hz, 1H), 1.85 (dddd, *J* = 18.6, 8.7, 7.1, 5.7 Hz, 1H), 1.78 (ddtd, *J* = 12.0, 8.5, 7.3, 6.2 Hz, 1H), 1.55 (ddt, *J* = 12.2, 8.7, 7.1 Hz, 1H). **$^{13}$C-NMR** (176 MHz, DMSO-d$_6$): δ 166.1, 164.3, 148.5, 100.5, 96.3, 77.2, 67.6,

47.6, 29.2, 25.6, 21.0. **HRMS**-ESI (m/z): $[M + H]^+$ calculated for C11H16NO3$^+$, 210.1125; found, 210.1121.

**4-hydroxy-1-isopentyl-6-methylpyridin-2(1H)-one (5t)**[10]

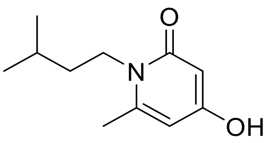


0.76 g, 50%. **$^1$H-NMR** (700 MHz, DMSO-$d_6$): δ 5.73 (dd, *J* = 2.7, 1.0 Hz, 1H), 5.46 (d, *J* = 2.7 Hz, 1H), 3.84 – 3.81 (m, 2H), 2.29 (s, 3H), 1.60 (dt, *J* = 13.3, 6.7 Hz, 1H), 1.37 (dt, *J* = 9.6, 6.8 Hz, 2H), 0.91 (d, *J* = 6.7 Hz, 6H). **$^{13}$C-NMR** (176 MHz, DMSO-$d_6$): δ 165.6, 163.7, 146.9, 100.4, 96.0, 41.4, 37.1, 26.0, 22.3, 19.7. **HRMS**-ESI (m/z): $[M + H]^+$ calculated for $C_{11}H_{18}NO_2^+$, 196.1332; found, 196.1330.

**7-hydroxy-5-oxo-1,2,3,5-tetrahydroindolizine-8-carboxylic acid (5u)**[11]

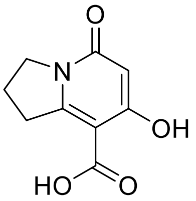


To a solution of methyl 7-hydroxy-5-oxo-1,2,3,5-tetrahydroindolizine-8-carboxylate (0.16 g, 0.76 mmol) in MeOH/THF/H2O (3:2:2) was added LiOH (91.6 mg, 3.82 mmol) and the mixture was stirred at room temperature overnight. The mixture was then concentrated, and the residual mixture was diluted with water and acidified to pH 5. The precipitated solid was filtered off, washed with cold H2O and dried in vacuo to afford the product as a white solid (0.12 g, 82%). **$^1$H-NMR** (500 MHz, DMSO-$d_6$): δ 5.55 (s, 1H), 3.96 (t, *J* = 7.5 Hz, 1H), 3.43 (t, *J* = 7.8 Hz, 1H), 2.08 (p, *J* = 7.8 Hz, 1H). **$^{13}$C-NMR** (126 MHz, DMSO-$d_6$): δ 171.4, 169.0, 161.5, 160.7, 96.4, 96.1, 49.3, 35.3, 20.2. **HRMS**-ESI (m/z): $[M + H]^+$ calculated for $C_9H_{10}O_4N^+$, 196.0604; found, 196.0601.

## 1.2. Synthesis of Functionalized Dihydropyrane Fragments

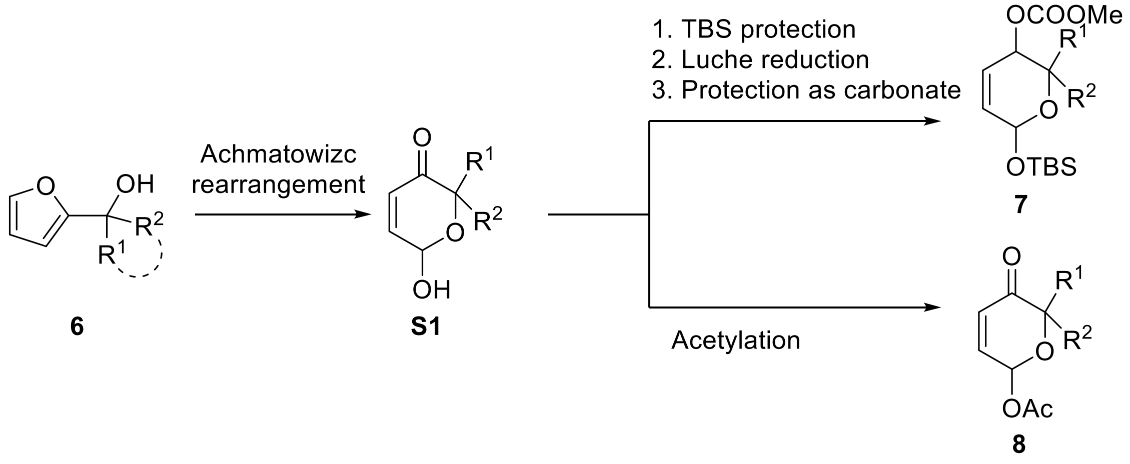


**Scheme S1**. General reaction sequence for the synthesis of functionalized dihydropyrane fragments.

**(±) 6-hydroxy-2H-pyran-3(6H)-one (S1a)**[12]

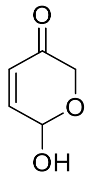

To a solution of furfuryl alcohol (5.00 g, 50.97 mmol) in dichloromethane (100 mL) at 0 °C was added *m*CPBA (13.19 g, 76.45 mmol, 1.5 equiv) in 3 portions over 45 minutes. The reaction mixture was allowed to slowly warm to ambient temperature and stirring was continued for 3 h. The reaction was then cooled to -20 °C and stirred for 15 minutes before removal of insoluble m-chlorobenzoic acid (white precipitate) by filtration. The filtrate was concentrated *in vacuo* and purified by flash column chromatography (EtOAc/Pet. Ether 1:3 to 2:3 to 1:1). The compound was isolated as a white crystalline solid (2.82 g, 49%). **$^1$H-NMR** (400 MHz, $CDCl_3$): δ 6.95 (dd, *J* = 10.4 Hz, 2.9 Hz, 1H), 6.17 (d, *J* = 10.4 Hz, 1H), 5.64 (d, *J* = 2.9 Hz, 1H), 4.58 (d, *J* = 16.9 Hz, 1H), 4.14 (d, *J* = 16.9 Hz, 1H), 3.00 (s, 1H).

**(±) 5-oxo-5,6-dihydro-2H-pyran-2-yl acetate (8a)**[13]

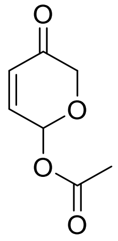

To **S22** (1.75 g, 15.31 mmol) in DCM (150 mL) was added pyridine (1.85 mL, 22.97 mmol, 1.5 equiv) and acetic anhydride (2.17 mL, 22.97 mmol, 1.5 equiv) at 0 °C. The mixture was allowed to warm to room temperature and kept stirring for 2 days. The reaction was quenched by addition of saturated $NaHCO_3$ solution (50 mL) and the layers were separated. The aqueous layer was extracted with EtOAc (3 x 50 mL). The combined organic layers were washed with brine (150 mL), dried over $MgSO_4$ and concentrated in vacuo. Flash chromatography (Pet. Ether/EtOAc 9:1 + 1% $NEt_3$) afforded the product as a colorless oil (1.77 g, 74%). **$^1$H-NMR** (400 MHz, $CDCl_3$): δ 6.92 (dd, *J* = 10.4, 3.6 Hz, 1H), 6.49 (dd, *J* = 3.6, 0.8 Hz, 1H), 6.27 (d, *J* = 10.4 Hz, 1H), 4.51 (d, *J* = 17.0 Hz, 1H), 4.23 (dd, *J* = 17.0, 0.5 Hz, 1H), 2.14 (s, 3H). **$^{13}$C-NMR** (100 MHz, $CDCl_3$): δ 193.5, 169.7, 142.4, 128.9, 86.8, 67.5, 21.1.

**(±) 6-((tert-butyldimethylsilyl)oxy)-2H-pyran-3(6H)-one (S2a)**[12]

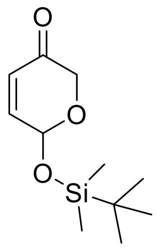

To a solution of **S1a** (2.80 g, 24.50 mmol) in THF (25 mL) was added $AgNO_3$ (5.00 g, 29.41 mmol, 1.2 equiv) and pyridine (8.78 mL, 0.11 mol, 4.44 equiv). The suspension was stirred for 20 minutes to allow the dissolution of any large lumps of solid. TBSCl (4.80 g, 31.86 mmol, 1.3 equiv) was added at 0 °C and precipitation of a white solid resulted. The reaction was stirred overnight at room temperature, after which the reaction mixture was filtered through celite and concentrated in vacuo. The resulting crude product was subjected to flash column chromatography (5% EtOAc / Pet. Ether) to afford the desired product as a white crystalline solid (5.33 g, 95%). **$^1$H-NMR**

(400 MHz, $CDCl_3$): δ 6.86 (dd, *J* = 10.3 Hz, 3.1 Hz, 1H), 6.08 (d, *J* = 10.3 Hz, 1H), 5.53 (d, *J* = 3.1 Hz, 1H), 4.50 (d, *J* = 16.8 Hz, 1H), 4.07 (d, *J* = 16.8 Hz, 1H), 0.92 (s, 9H), 0.17 (s, 6H).

**(±) *Cis* 6-((tert-butyldimethylsilyl)oxy)-3,6-dihydro-2H-pyran-3-ol (S3a)**[12]

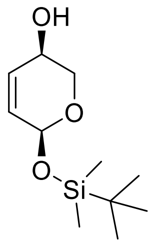

To a cooled (- 20 °C) solution of **S2a** (8.20 g, 35.91 mmol) in methanol (34 mL) was added $CeCl_3$ x $7H_2O$ (16.05 g, 43.09 mmol, 1.2 eq). The reaction mixture was kept at -20 °C and sodium borohydride (1.63 g, 43.09 mmol, 1.2 eq) was added portionwise over 45 minutes. The reaction was stirred at -20 °C for 3 h before quenching with 35 mL of acetone. The reaction mixture was warmed to room temperature, filtered through celite and concentrated in vacuo. The crude was diluted with water (150 mL) and dichloromethane (150 mL) and filtered through celite. The filtrate was then extracted with dichloromethane (3 x 100 mL) and the combined organic phases were washed with brine (200 mL), dried over $MgSO_4$, filtered and concentrated in vacuo. The crude product was purified by flash column chromatography (Pet. Ether/EtOAc 95:5 to 9:1 to 4:1). The product was isolated as a racemic mixture of the *cis*-isomer as a clear colourless oil (5.074 g, 61%). A mixed fraction of racemic *cis*- and *trans* isomer was isolated as well (*cis:trans* = 0.5:1, 36.7 mg, 0.5%). Overall dr *cis:trans* = 1:0.005. **$^1$H-NMR** *cis*-isomer (400 MHz, $CDCl_3$): δ 5.94 (dd, *J* = 10.3 Hz, 2.7 Hz, 1H), 5.75 (ddd, *J* = 10.3 Hz, 2.3 Hz, 1.7 Hz, 1H), 5.25 (m, 1H), 4.13 (m, 1H), 3.77 (m, 2H), 1.57 (bs, 1H), 0.91 (s, 9H), 0.13 (s, 6H).

**(±) *Cis* 6-((tert-butyldimethylsilyl)oxy)-3,6-dihydro-2H-pyran-3-yl methyl carbonate (7a)**[12]

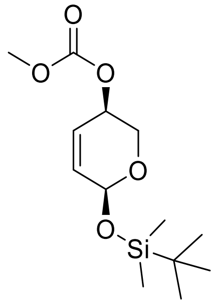

To a solution of **S3a** (4.179 g, 18.14 mmol) in degassed DCM (40 mL) was added DMAP (2.770 g, 22.67 mmol, 1.25 equiv), followed by methyl chloroformate (1.750 mL, 22.67 mmol, 1.25 equiv). The reaction was stirred for 1 d at room temperature before concentrating in vacuo and purification by flash column chromatography (5% EtOAc / Pet. Ether). The desired product was isolated as a clear colourless oil (4.191 g, 82% yield). **$^1$H-NMR** (600 MHz, $CDCl_3$): δ 5.91 (dd, *J* = 10.3 Hz, 2.2 Hz, 1H), 5.84 (dt, *J* = 10.3 Hz, 2.0 Hz, 1H), 5.27 (bs, 1H), 5.14 – 5.09 (m, 1H), 3.92 – 3.85 (m, 2H), 3.79 (s, 3H), 0.90 (s, 9H), 0.13 (s, 3H), 0.12 (s, 3H). **$^{13}$C-NMR** (151 MHz, $CDCl_3$): δ 155.4, 132.3, 126.5, 89.3, 68.5, 60.2, 55.0, 25.8, -3.5, -4.3, -5.1.

**(±) 6-hydroxy-2-methyl-2H-pyran-3(6H)-one (S1b)**[14]

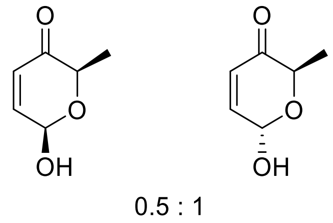


A 250 mL schlenck-flask was evacuated and backfilled with argon. $Na_2S_2O_8$ (11.1 g, 46.82 mmol) and $Ru(bpy)_3Cl_2$ x 6 $H_2O$ (66.8 mg, 0.2 mol%) were added and dissolved in water (85 mL), followed by 1-(2-furyl)-ethanol (5.00 g, 44.59 mmol, 1.0 equiv) in acetonitrile/DMSO (85 mL, 1:1). The reaction mixture was kept in the dark while argon was bubbled through it for 15 minutes. Then the reaction mixture was irradiated in the batch photoreactor with rapid stirring for 4 hours at 25 °C. After completion of reaction the mixture was diluted with brine (60 mL) and extracted with EtOAc (3 x 200 mL). The combined organic phases were dried over $MgSO_4$, filtered and concentrated in vacuo. The crude product was plugged through a short pad of silica and dried in vacuo to afford the product as a mixture of *trans/cis* isomere (1:0.5, 3.85 g, 67%). **$^1$H-NMR** *trans*-isomer (600 MHz, $CDCl_3$): δ 6.89 (dd, *J* = 10.2 Hz, 3.3 Hz, 1H), 6.11 (d, *J* = 10.2 Hz, 1H), 5.63 (d, *J* = 3.3 Hz, 1H), 4.71 (q, *J* = 6.8 Hz, 6.8 Hz, 6.8 Hz, 1H), 3.00 (bs, 1H), 1.39 (d, *J* = 6.8 Hz, 3H). **$^{13}$C-NMR** *trans*-isomer (151 MHz, $CDCl_3$): δ 196.9, 144.4, 127.3, 87.7, 70.4, 15.3. **$^1$H-NMR** *cis*-isomer (600 MHz, $CDCl_3$): δ 6.94 (dd, *J* = 10.3 Hz, 1.3 Hz, 1H), 6.15 (dd, *J* = 10.3 Hz, 1.5 Hz, 1H), 5.68 (bd, *J* = 1.3 Hz, 1H), 4.23 (qd, *J* = 6.8 Hz, 6.8 Hz, 6.8 Hz, 1.1 Hz, 1H), 1.65 (bs, 1H), 1.46 (d, *J* = 6.8 Hz, 3H). **$^{13}$C-NMR** *cis*-isomer (151 MHz, $CDCl_3$): δ 196.4, 148.0, 128.6, 91.0, 75.3, 16.3.

**(±) 6-((tert-butyldimethylsilyl)oxy)-2-methyl-3,6-dihydro-2H-pyran-3-yl methyl carbonate (7b)**

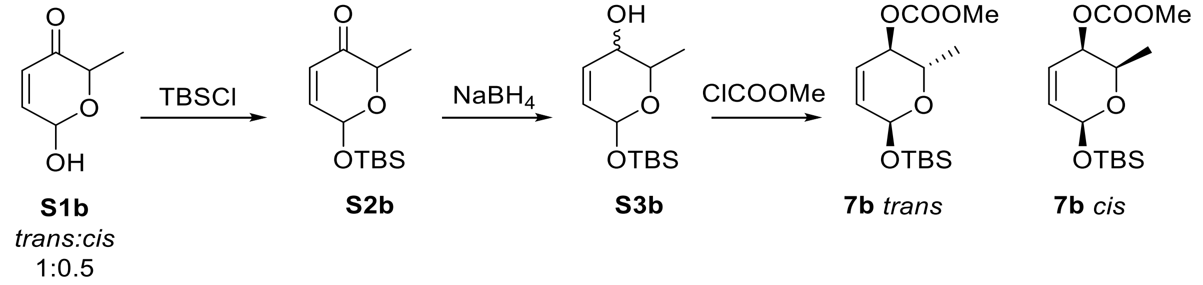


To a solution of **S1b** (3.85 g, 30.0 mmol) in THF (140 mL) was added $AgNO_3$ (7.65 g, 45.0 mmol, 1.5 equiv) and pyridine (10.8 mL, 133.2 mmol, 4.44 equiv). The suspension was stirred for 20 minutes to allow the dissolution of any large lumps of solid. TBSCl (6.79 g, 45.0 mmol, 1.5 equiv) was added at 0 °C and precipitation of a white solid resulted. The reaction was stirred overnight and filtered through celite. After dilution with with EtOAc (150 mL) the mixture was washed with saturated $NaHCO_3$ solution solution (200 mL) and the aqueous phase was

extracted with EtOAc (3 x 100 mL). The combined organic phases were washed with brine (200 mL), dried over $MgSO_4$ and concentrated in vacuo. The resulting crude product was plugged through a short pad of silica and the pad was flushed with DCM. Solvents were removed under reduced pressure and the crude was dried under high vacuum overnight to afford the desired crude product. The crude was dissolved in anhydrous DCM (200 mL) and $CeCl_3$ x 7 $H_2O$ (2.45 g, 6.58 mmol, 0.22 equiv) in methanol (12 mL) was added at -78 °C, followed by addition of $NaBH_4$ (1.36 g, 35.89 mmol, 1.3 equiv). The reaction was stirred for 4 hours at – 78 °C after which it was quenched by addition of acetone (2.1 mL) and saturated $NaHCO_3$ solution (200 mL). The layers were separated and the aqueous phase was extracted with DCM (3 x 100 mL). The combined organic phases were washed with brine (200 mL), dried over $MgSO_4$ and concentrated in vacuo. The crude was dissolved in anhydrous DCM (200 mL) and cooled to 0 °C. DMAP (0.36 g, 2.99 mmol, 0.1 equiv), pyridine (14.5 mL, 179.21 mmol, 6 equiv) and methyl chloroformate (13.9 mL, 179.21 mmol, 6 equiv) was added and the reaction was allowed to stir overnight at room temperature. The reaction mixture was quenched with saturated $NaHCO_3$ solution (200 mL), extracted with DCM (5 x 50 mL), dried over $MgSO_4$ and concentrated in vacuo. The crude was purified by flash chromatography (Pet. Ether/Tol 9:1 + 1% $NEt_3$) to afford the desired *trans*-product as a colorless oil (5.86 g, 65%). The *cis*-isomere was isolated in a separated fraction (2.93 g, 32%). **$^1$H-NMR** *trans*-isomere (400 MHz, $CDCl_3$): δ 5.83 (bd, *J* = 10.3 Hz, 1H), 5.78 (ddd, *J* = 10.3 Hz, 2.7 Hz, 1.9 Hz 1H), 5.31 (d, *J* = 1.9 Hz, 1H), 4.85 (ddd, *J* = 9.2 Hz, 2.7 Hz, 1.6 Hz, 1H), 4.04 (dd, *J* = 9.2 Hz, 6.3 Hz, 1H), 3.81 (s, 3H), 1.25 (d, *J* = 6.3 Hz, 1H), 0.90 (s, 9H), 0.12 (s, 6H). **$^{13}$C-NMR** *trans*-isomere (100 MHz, $CDCl_3$): δ 155.2, 130.8, 127.3, 89.2, 74.9, 64.4, 55.1, 25.8, 18.2, 18.1, -5.2. **$^1$H-NMR** *cis*-isomere (400 MHz, $CDCl_3$): δ 6.01-5.94 (m, 2H), 5.31 (bs, 1H), 4.80-4.78 (m, 1H), 3.85 (ddd, *J* = 6.5 Hz, 6.5 Hz, 2.8 Hz, 1H), 3.78 (s, 3H), 1.27 (d, *J* = 6.5 Hz, 1H), 0.90 (s, 9H), 0.12 (s, 6H). **$^{13}$C-NMR** *cis*-isomere (100 MHz, $CDCl_3$): δ 155.9, 136.3, 124.2, 92.8, 69.8, 69.6, 55.0, 25.9, 18.3, 16.5, -3.7, -4.5.

**(±) *Trans*-6-methyl-5-oxo-5,6-dihydro-2H-pyran-2-yl acetate (8b)[15]**

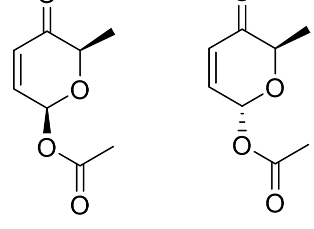

0.3 : 1

To **S1b** (500 mg, 3.90 mmol) in DCM (40 mL) was added pyridine (0.38 mL, 4.68 mmol, 1.2 equiv) and acetic anhydride (0.44 mL, 4.68 mmol, 1.2 equiv) at 0 °C. The mixture was allowed to warm to room temperature and kept stirring for 2 days. The reaction was quenched by addition of saturated $NaHCO_3$ solution (50 mL) and the layers were separated. The aqueous layer was extracted with EtOAc (3 x 50

mL). The combined organic layers were washed with brine (150 mL), dried over $MgSO_4$ and concentrated in vacuo. Flash chromatography (Pet. Ether/EtOAc 1:0 to 4:1) afforded two separated fractions of the racemic *cis*- and desired *trans* isomer (0.3: 1, 595 mg, 90%). **$^1$H-NMR** *trans-isomer* (700 MHz, $CDCl_3$): δ 6.87 (dd, *J* = 10.2 Hz, 3.6 Hz, 1H), 6.48 (d, *J* = 3.6 Hz, 1H), 6.21 (d, *J* = 10.2 Hz, 1H), 4.60 (q, *J* = 6.7 Hz, 6.7 Hz, 6.7 Hz, 1H), 2.14 (s, 3H), 1.41 (d, *J* = 6.7 Hz, 3H). **$^{13}$C-NMR** *trans*-isomer (176 MHz, $CDCl_3$): δ 196.0, 169.6, 141.8, 128.4, 87.1, 72.5, 21.0, 15.4. **$^1$H-NMR** *cis*-isomer (700 MHz, $CDCl_3$): δ 6.87 (dd, *J* = 10.3 Hz, 3.6 Hz, 1H), 6.55 (bs, 1H), 6.22 (d, *J* = 10.3 Hz, 1H), 4.37 (q, *J* = 7.0 Hz, 7.0 Hz, 7.0 Hz, 1H), 2.15 (s, 3H), 1.49 (d, *J* = 7.0 Hz, 3H). **$^{13}$C-NMR** *cis*-isomer (176 MHz, $CDCl_3$): δ 195.7, 169.2, 143.7, 128.5, 88.0, 76.0, 21.2, 18.3.

**(±) 6-hydroxy-2,2-dimethyl-2H-pyran-3(6H)-one (S1c)[16]**

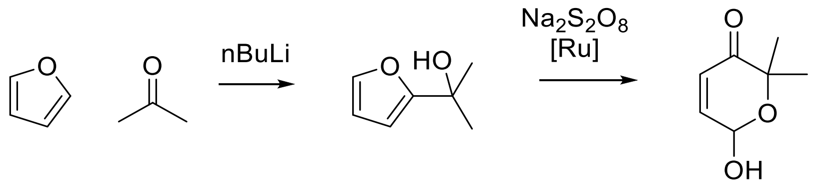

**S1c**

An oven dried flask was evacuated and filled with argon three times. To this flask was added dry THF (160 mL), followed by freshly distilled furan (8.70 mL, 120 mmol, 1.5 equiv). The solution was stirred and cooled to 0° C, and then n-BuLi (38.4 mL, 1.2 equiv, 2.5 M solution in hexanes) was added slowly. The reaction mixture was allowed to stir at 0° C for 1 hour at which point the reaction mixture was cooled to -78° C. Freshly distilled acetone (5.88 mml, 80.0 mmol) was added slowly and the reaction mixture was allowed to stir and warm to room temperature over 18 hours. The resulting reaction mixture was quenched under inert atmosphere via slow addition of saturated saturated $NH_4Cl$ (50 mL) followed by brine (50 mL). The aqueous phase was extracted with EtOAc (3 x 100 mL). The combined organic phases were dried over $MgSO_4$ and concentrated via rotary evaporator. The resulting crude product was dissolved in acetonitrile/DMSO (160 mL, 1:1) and loaded into a 500 mL oven dried schlenck- flask under argon. $Na_2S_2O_8$ (20.0 g, 84.00 mmol, 1.05 equiv) and $Ru(bpy)_3Cl_2$ x 6 $H_2O$ (149.7 mg, 0.2 mol%) dissolved in water (160 mL) were added to the mixture and argon was bubbled through it for 15 minutes in the dark. Then the reaction mixture was irradiated in the batch photoreactor with rapid stirring for 5 hours at 25 °C. After completion of reaction the mixture was diluted with brine (100 mL) and extracted with EtOAc (3 x 200 mL). The combined organic phases were dried over $MgSO_4$, filtered and concentrated in vacuo. The crude product was plugged through a short pad of silica and dried in vacuo to afford the product as colorless oil (5.69 g,

50%). **$^1$H-NMR** (400 MHz, $CDCl_3$): δ 6.87 (dd, *J* = 10.3, 2.1 Hz, 1H), 6.03 (dd, *J* = 10.3, 1.4 Hz, 1H), 5.70 (dd, *J* = 2.1, 1.4 Hz, 1H), 4.63 (d, *J* = 5.9 Hz, 1H), 1.47 (s, 3H), 1.38 (s, 3H).

**(±) 6,6-dimethyl-5-oxo-5,6-dihydro-2H-pyran-2-yl acetate (8c)**[17]

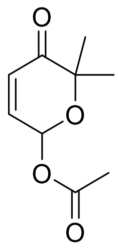

To **S1c** (2.84 g, 20.0 mmol) in DCM (200 mL) was added pyridine (4.03 mL, 50.0 mmol, 2.5 equiv) and acetic anhydride (3.77 mL, 40.0 mmol, 2 equiv) at 0 °C. The mixture was allowed to warm to room temperature and kept stirring overnight. The reaction was quenched by addition of saturated $NaHCO_3$ solution (100 mL) and the layers were separated. The aqueous layer was extracted with DCM (3 x 100 mL). The combined organic layers were washed with brine (250 mL), dried over $MgSO_4$ and concentrated in vacuo. Flash chromatography (Pet. Ether/EtOAc 1:0 to 4:1) provided the desired product as a yellow oil (2.06 g, 56%). **$^1$H-NMR** (700 MHz, $CDCl_3$): δ 6.82 (dd, *J* = 10.3, 3.2 Hz, 1H), 6.56 (dd, *J* = 3.2, 1.1 Hz, 1H), 6.16 (dd, *J* = 10.3, 1.1 Hz, 1H), 2.12 (s, 3H), 1.49 (s, 3H), 1.42 (s, 3H). **$^{13}$C-NMR** (176 MHz, $CDCl_3$): δ 198.4, 169.7, 141.7, 127.1, 86.9, 80.2, 27.2, 21.4.

**(±) Tert-butyl 2-acetoxy-5-oxo-1-oxa-9-azaspiro[5.5]undec-3-ene-9-carboxylate (8d)**

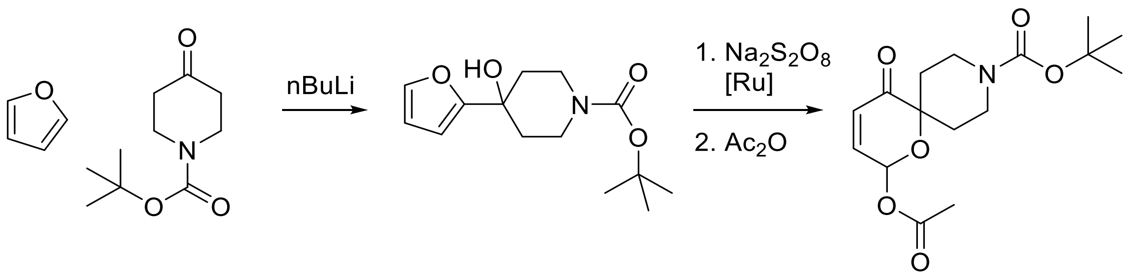


An oven dried flask was evacuated and filled with argon three times. To this flask was added dry THF (125 mL), followed by freshly distilled furan (2.91 mL, 40.15 mmol, 1.6 equiv). The solution was stirred and cooled to 0° C, and then n-BuLi (16.5 mL, 1.05 equiv, 1.6 M solution in hexanes) was added slowly. The reaction mixture was allowed to stir at 0° C for 1 hour at which point the reaction mixture was cooled to -78° C. Tert-butyl 4-oxopiperidine-1-carboxylate (5.00 g, 25.09 mmol) was added slowly and the reaction mixture was allowed to stir and warm to room temperature over 18 hours. The resulting reaction mixture was quenched under inert atmosphere via slow addition of saturated $NH_4Cl$ (50 mL) followed by brine (50 mL). The aqueous phase was extracted with EtOAc (3 x 100 mL). The combined organic phases were dried over $MgSO_4$ and concentrated via rotary evaporator. The resulting crude product was dissolved in acetonitrile/DMSO (50 mL, 1:1) and loaded into a 250 mL oven dried

schlenck- flask under argon. $Na_2S_2O_8$ (6.27 g, 26.3 mmol, 1.05 equiv) and $Ru(bpy)_3Cl_2$ x 6 $H_2O$ (37.6 mg, 0.2 mol%) dissolved in water (50 mL) were added to the mixture and argon was bubbled through it for 15 minutes in the dark. Then the reaction mixture was irradiated in the batch photoreactor with rapid stirring for 5 hours at 25 °C. After completion of reaction the mixture was diluted with brine (100 mL) and extracted with EtOAc (3 x 200 mL). The combined organic phases were dried over $MgSO_4$, filtered and concentrated in vacuo. The crude product was plugged through a short pad of silica and dried in vacuo to afford the product as colorless oil (5.90 g, 83%). This was dissolved in DCM (200 mL). Pyridine (4.19 mL, 52.1 mmol, 2.5 equiv) and acetic anhydride (2.36 mL, 25.0 mmol, 1.2 equiv) were then added at 0 °C. The mixture was allowed to warm to room temperature and kept stirring overnight. The reaction was quenched by addition of saturated $NaHCO_3$ solution (100 mL) and the layers were separated. The aqueous layer was extracted with DCM (3 x 100 mL). The combined organic layers were washed with brine (250 mL), dried over $MgSO_4$ and concentrated in vacuo. Flash chromatography (Pet. Ether/EtOAc 1:0 to 9:1) provided the desired product as a yellow solid (6.00 g, 89%). **$^1$H-NMR** (500 MHz, $CDCl_3$): δ 6.83 (dd, *J* = 10.3, 3.2 Hz, 1H), 6.61 (dd, *J* = 3.2, 1.1 Hz, 1H), 6.19 (dd, *J* = 10.3, 1.1 Hz, 1H), 4.07-3.86 (m, 2H), 3.20-2.98 (m, 2H), 2.12 (s, 3H), 2.05 – 1.99 (m, 2H), 1.76 (td, *J* = 13.1, 4.7 Hz, 1H)1.68-1.61 (m, 1H), 1.46 (s, 9H). **$^{13}$C-NMR** (126 MHz, $CDCl_3$): δ 196.9, 169.6, 147.0, 141.3, 127.3, 86.7, 79.9, 79.2, 38.1, 31.6, 28,6, 21.2. **HRMS**-ESI (m/z): $[M + H]^+$ calculated for $C_{16}H_{24}NO_6^+$, 326.1598; found, 326.1600.

**(±) 2-hydroxy-1-oxa-9-azaspiro[5.5]undec-3-en-5-one (8e)**

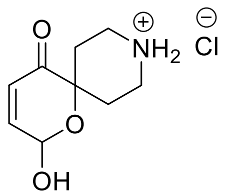


**8d** (22.0 mg, 0.07 mmol) was treated with HCl in dioaxen (4 M, 1 mL) at room temperature overnight. Diethyl ether (10 mL) and 1 M aqueous HCl (10 mL) were added and the phases were separated. The aqueous phase was concentrated and triturated with diethyl ether. The white precipitate was filtered off and dried in vacuo to afford the desired product (14.7 mg, 99%). **$^1$H-NMR** (500 MHz, DMSO-$d_6$): δ 8.24 (d, *J* = 5.6 Hz, 1H), 6.47 (d, *J* = 5.6 Hz, 1H), 3.21-3.11 (m, 5H), 3.07-2.97 (m, 1H), 2.73 (dt, *J* = 11.8, 6.0 Hz, 4H). **$^{13}$C-NMR** (126 MHz, DMSO-$d_6$): δ 169.6, 145.1, 141.7, 120.9, 119.4, 43.5, 42.9, 24.4, 24.3.

## 1.3. Synthesis of Pyrano-Furo-Pyridones

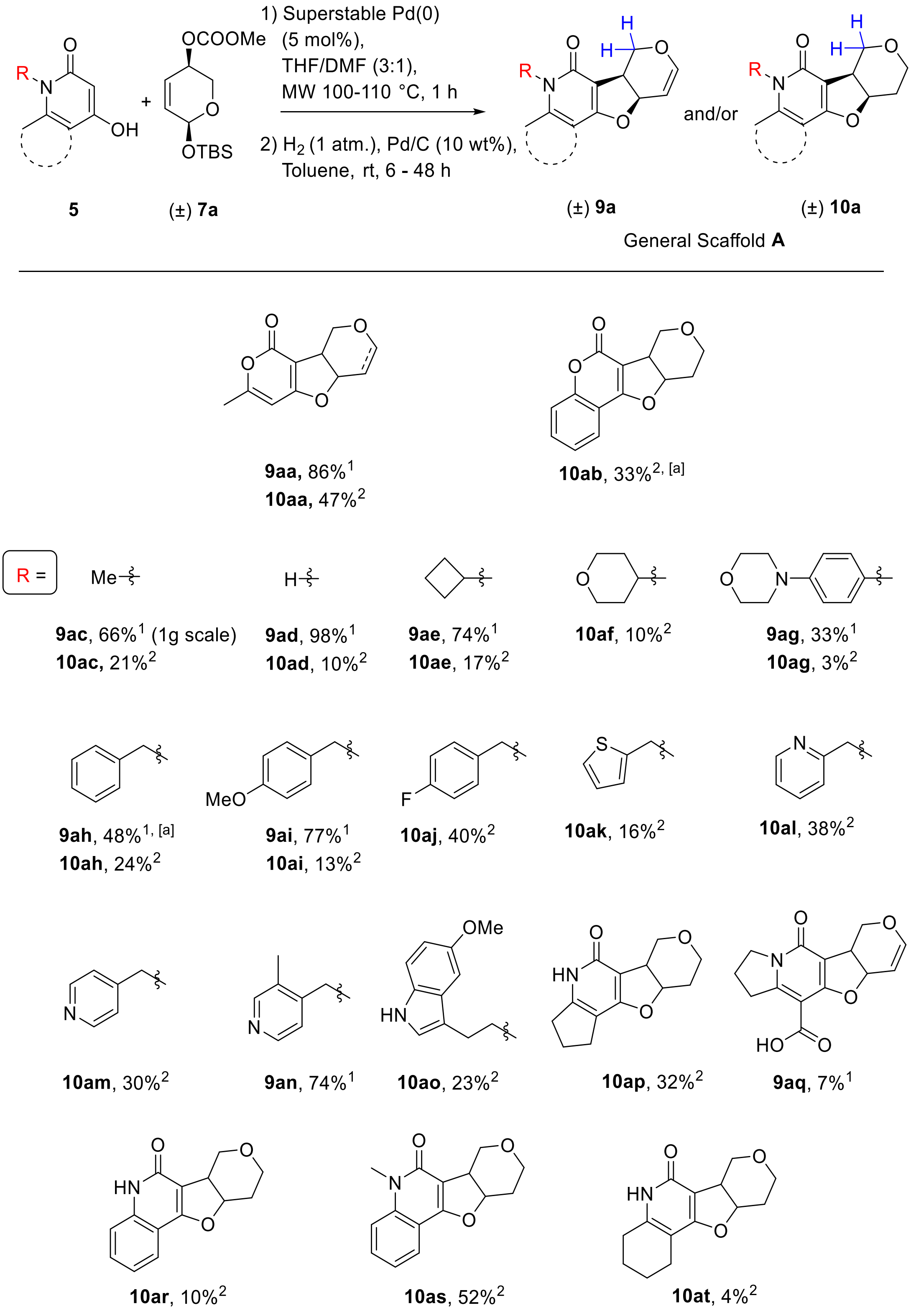


**Scheme S2.** Synthesis of general scaffold **A** derivatives by Pd-AAC employing various pyridones **5** and dihydropyran **7a**. 1) Yield for the Pd-AAC step. 2) Yield after the heterogenous reduction over two steps. [a] Pd-tetrakis was used as a catalyst. For compound numbering the first digit indicates the general scaffold according to Scheme 1 in the main text, the first letter indicates a specific sub-scaffold (blue) and the second letter indicates consecutive derivatives (red).

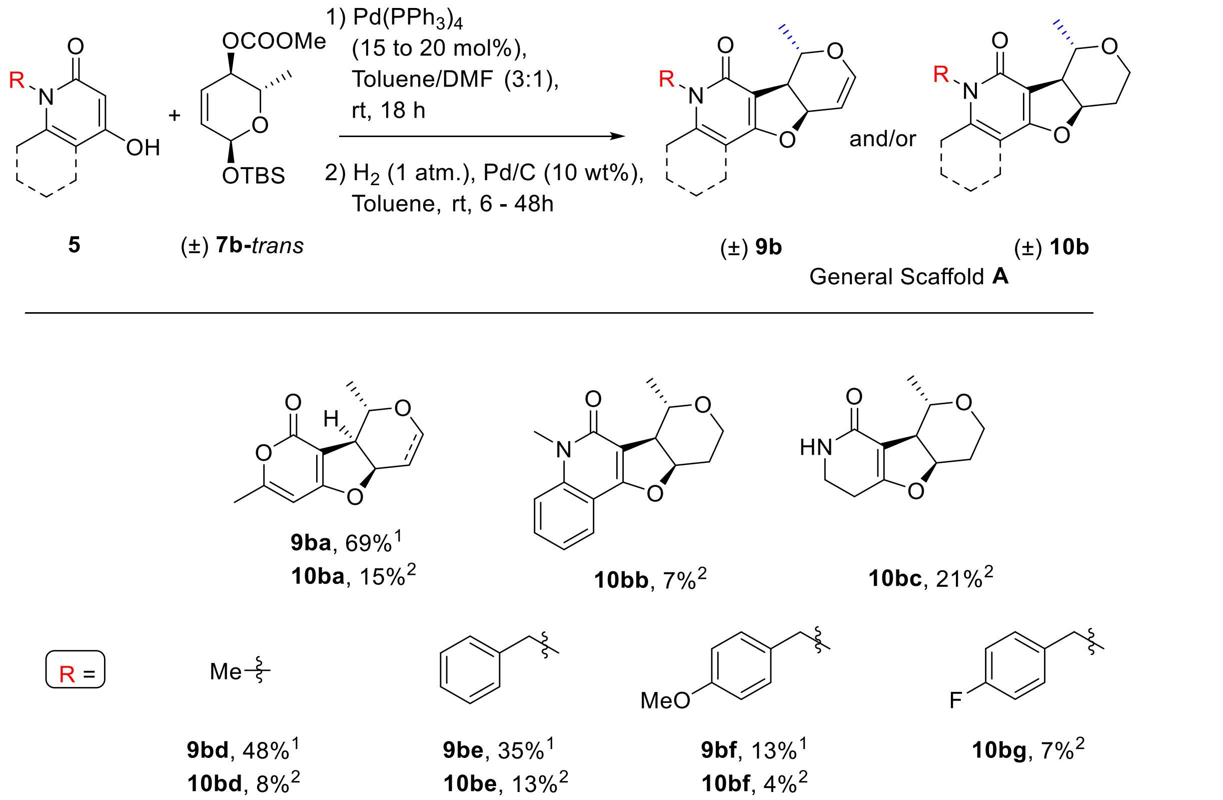


**Scheme S3.** Synthesis of general scaffold **A** derivatives by Pd-AAC employing various pyridones **5** and dihydropyran **7b**-*trans*. 1) Yield for the Pd-AAC step. 2) Yield after the heterogenous reduction over two steps. For compound numbering the first digit indicates the general scaffold according to Scheme 1 in the main text, the first letter indicates a specific sub-scaffold (blue) and the second letter indicates consecutive derivatives (red).

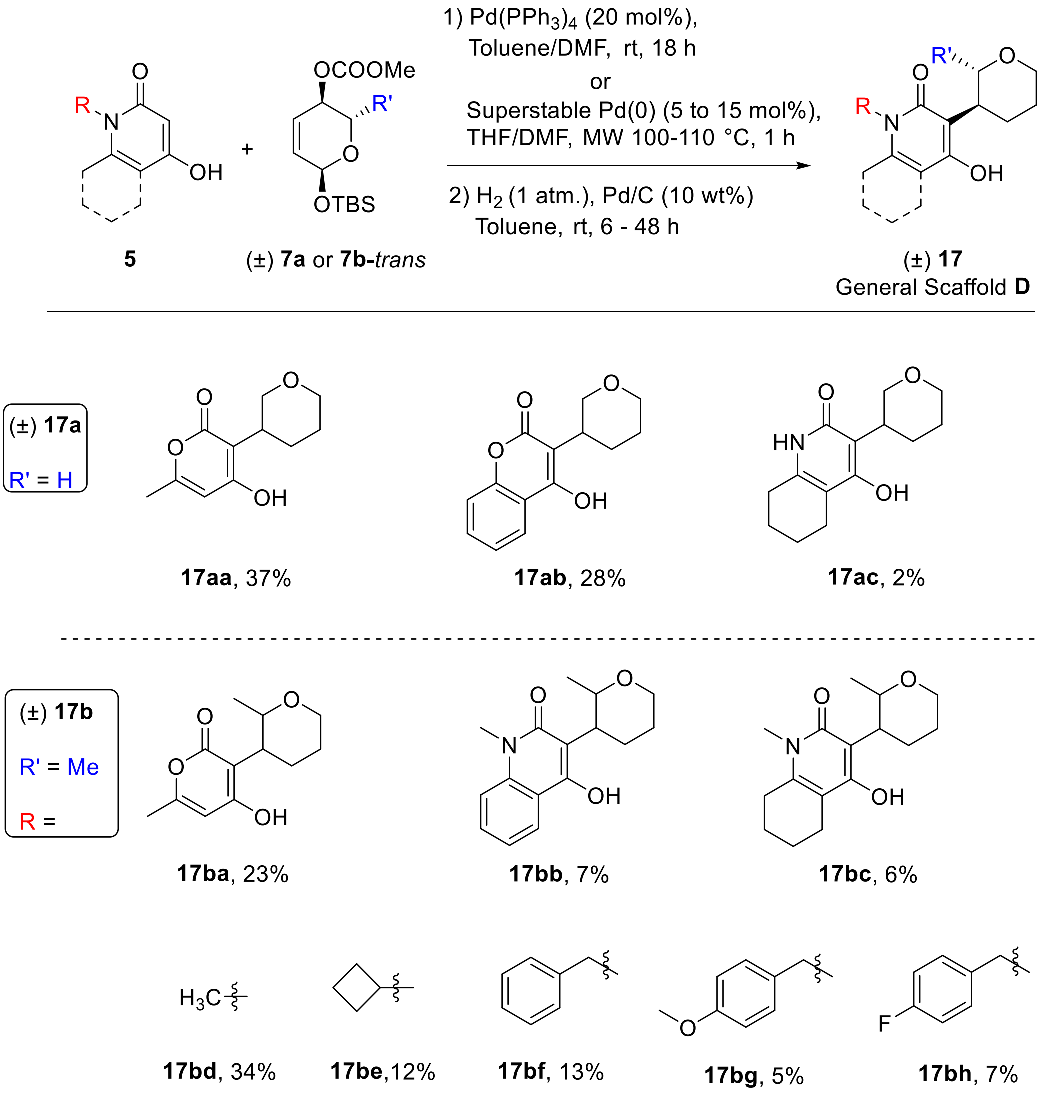


**Scheme S4.** Isolated monopodal connected side products **17a** and **17b** after heterogenous reduction of Pd-catalyzed allylic alkylation products. Yields are given over two steps. For compound numbering the first digit indicates the general scaffold according to Scheme 1 in the main text, the first letter indicates a specific sub-scaffold (blue) and the second letter indicates consecutive derivatives (red).

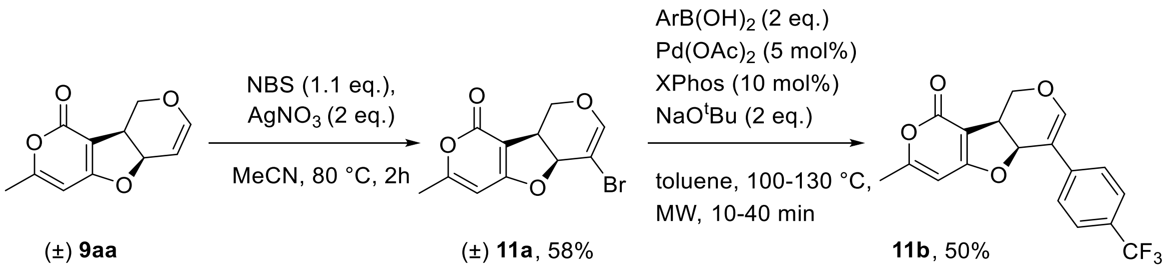


**Scheme S5.** Functionalization of **9aa** at C3 of the glycal moiety.

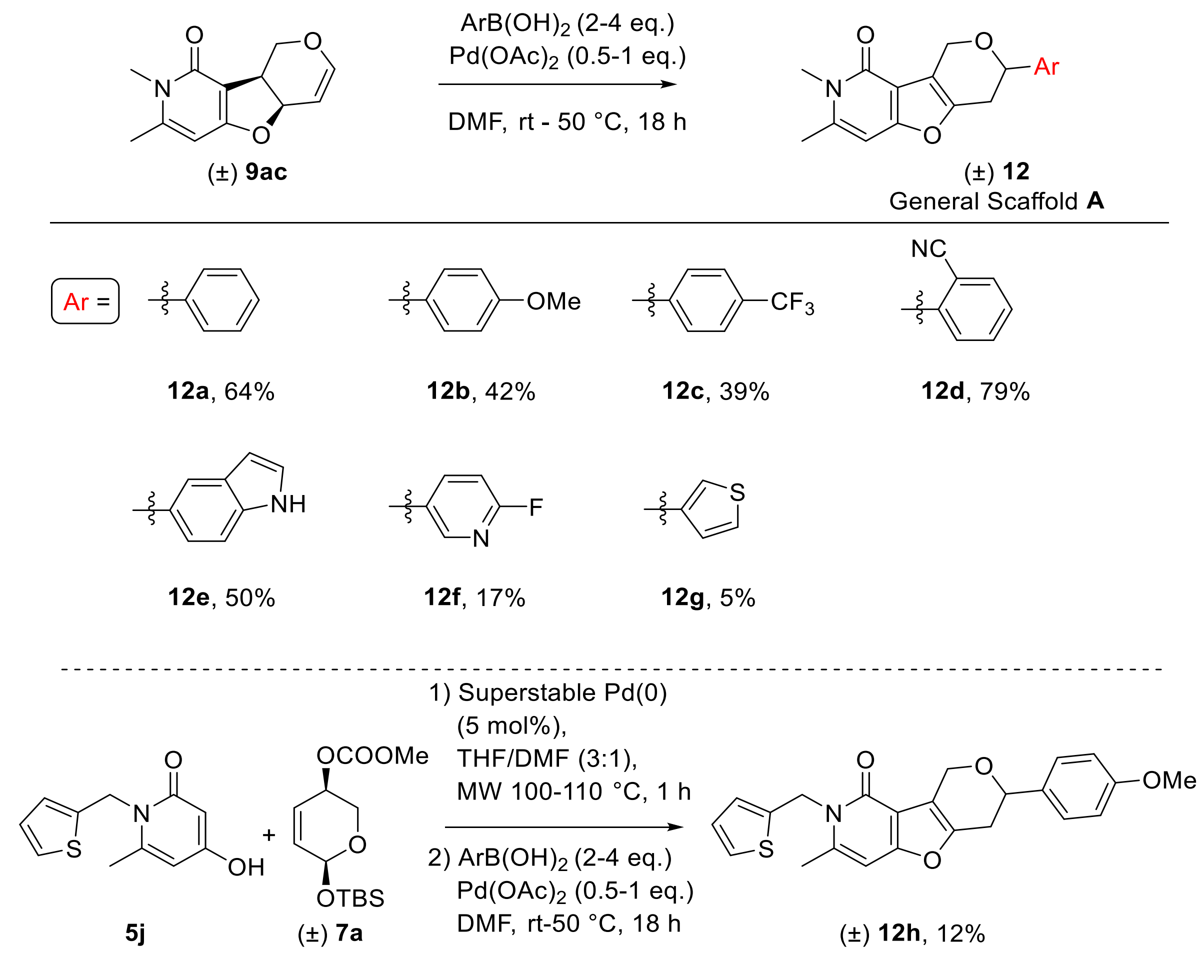

**Scheme S6.** Pd(II) catalyzed Heck-type *C*-glycosidation of **9ac** and **9ak** with arylboronic acids.

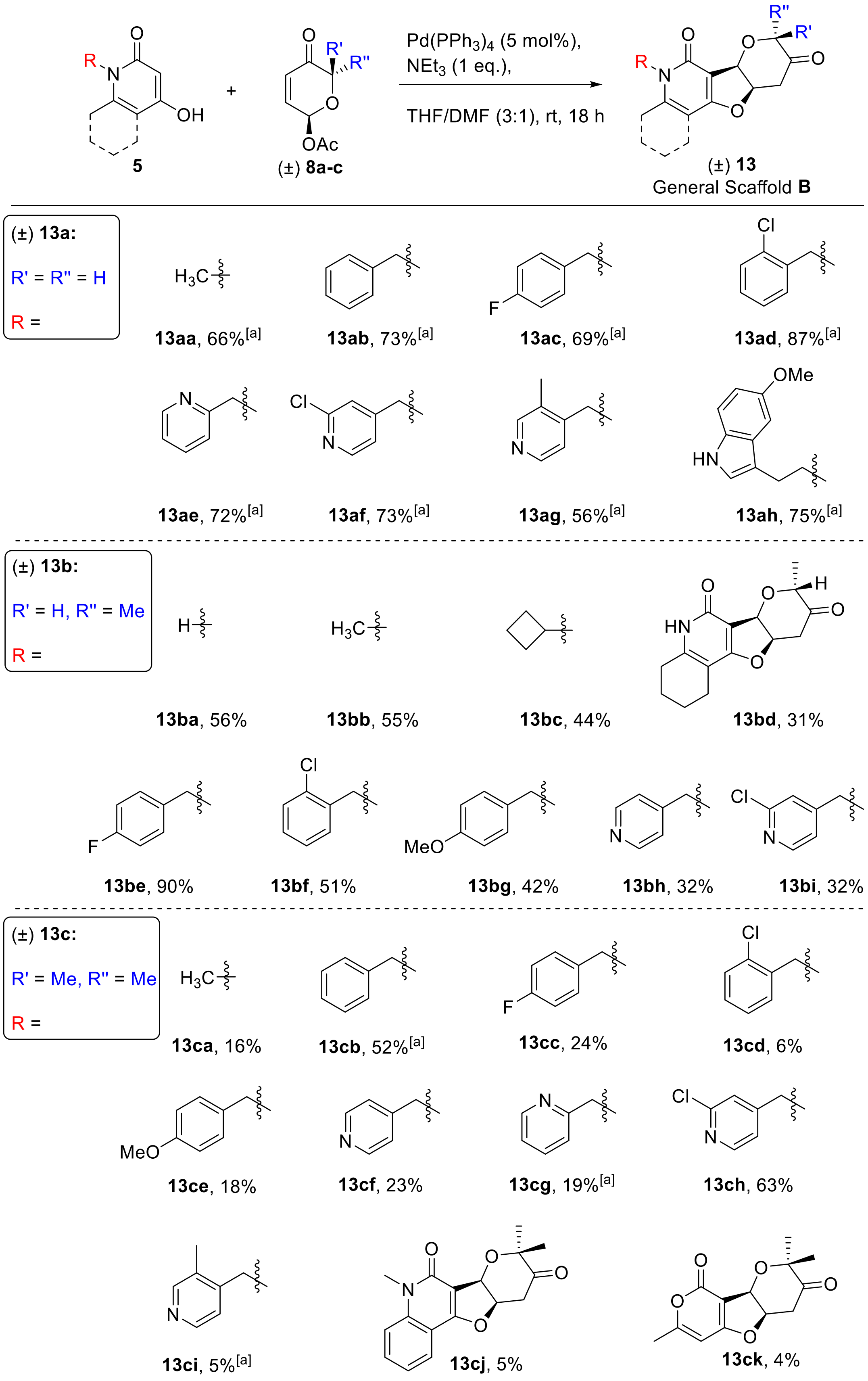


**Scheme S7.** Synthesis of derivatives of general scaffold **B** by Tsuji-Trost oxa-Michael cascade employing various pyridones **5** and dihydropyranones **8a**-**c**. [a] Allyl-Pd(II)-chloride dimer + Xanthphos was used as a catalyst. For compound numbering the first digit indicates the general scaffold according to Scheme 1 in the main text, the first letter indicates a specific sub-scaffold (blue) and the second letter indicates consecutive derivatives (red).

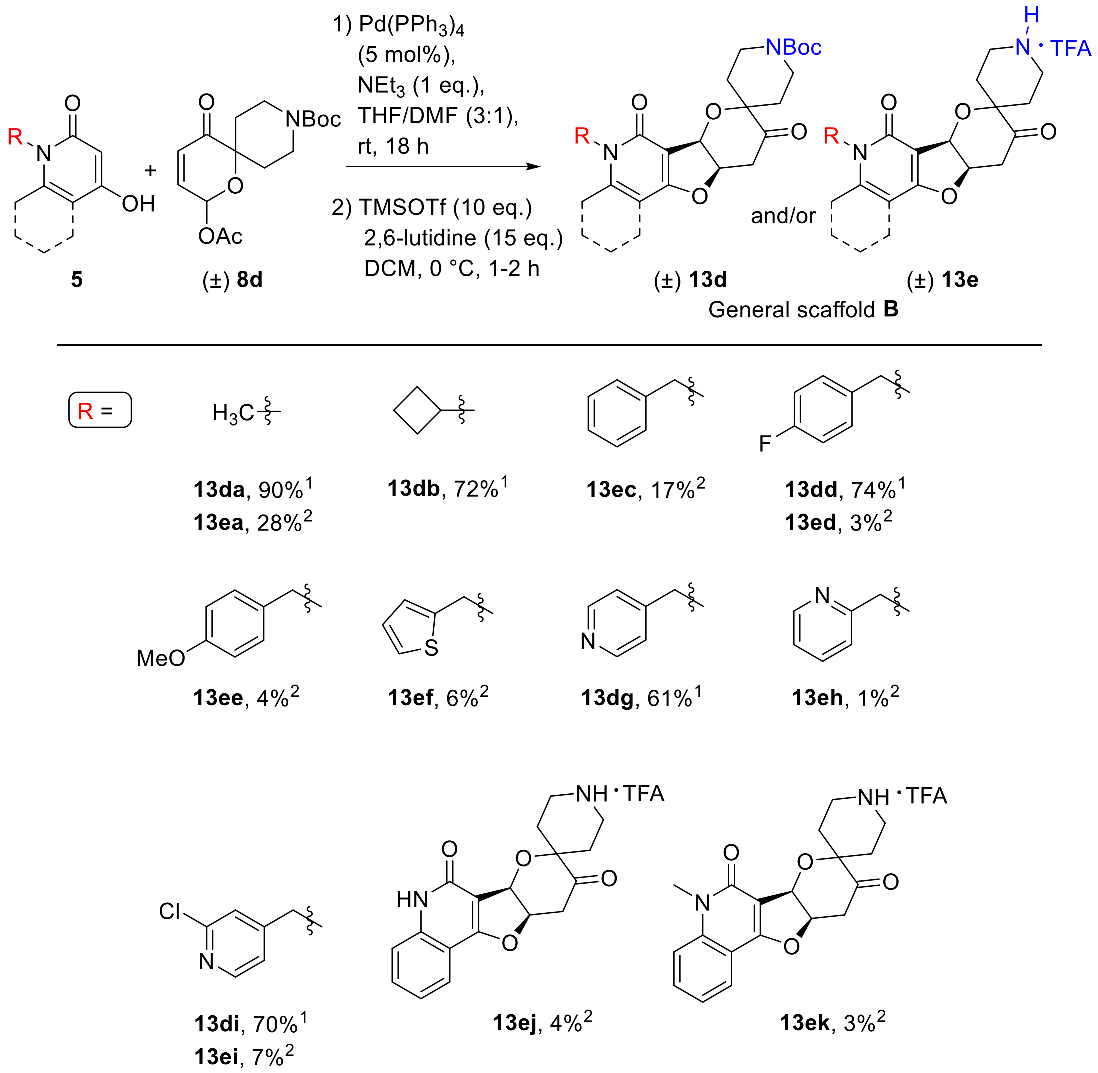


**Scheme S8.** Synthesis of Tsuji-Trost oxa-Michael cascade products employing various pyridones **5** and dihydropyranone **8d**. 1) Yield for the Tsuji-Trost oxa-Michael cascade step. 2) Yield after the Boc-deprotection over two steps. For compound numbering the first digit indicates the general scaffold according to Scheme 1 in the main text, the first letter indicates a specific sub-scaffold (blue) and the second letter indicates consecutive derivatives (red).

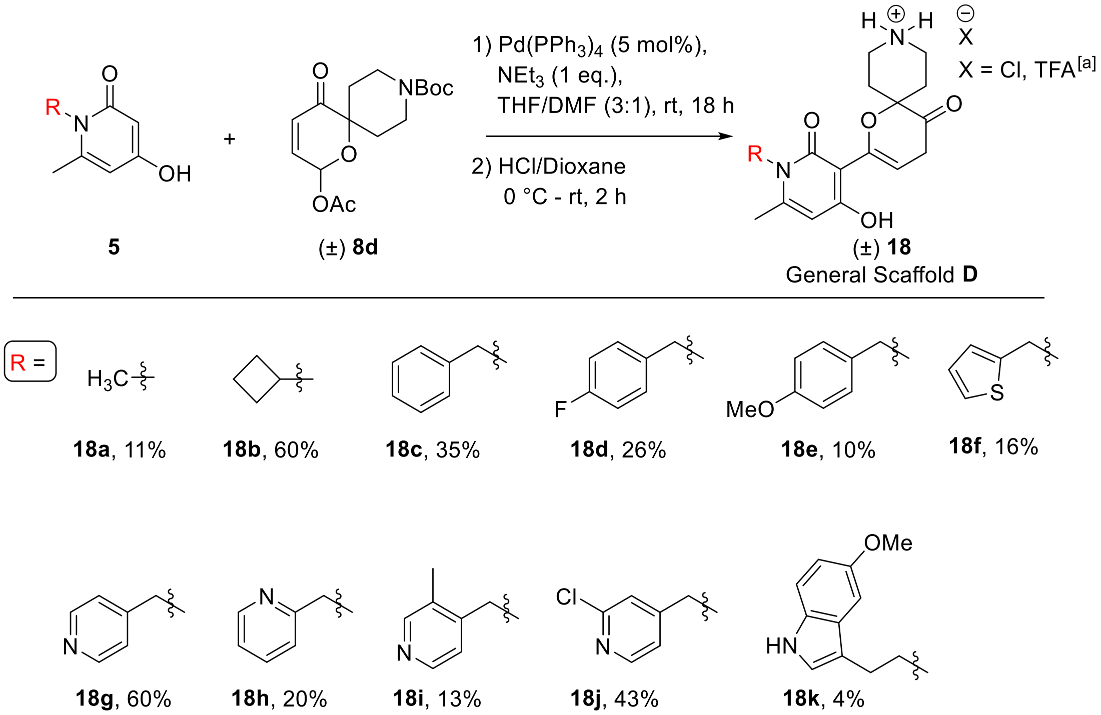


**Scheme S9.** Isolated monopodal connected side products after treatment with HCl of Tsuji-Trost oxa-Michael products. Yields are given over two steps. [a] Products were isolated as TFA salts when purified by prep. HPLC.

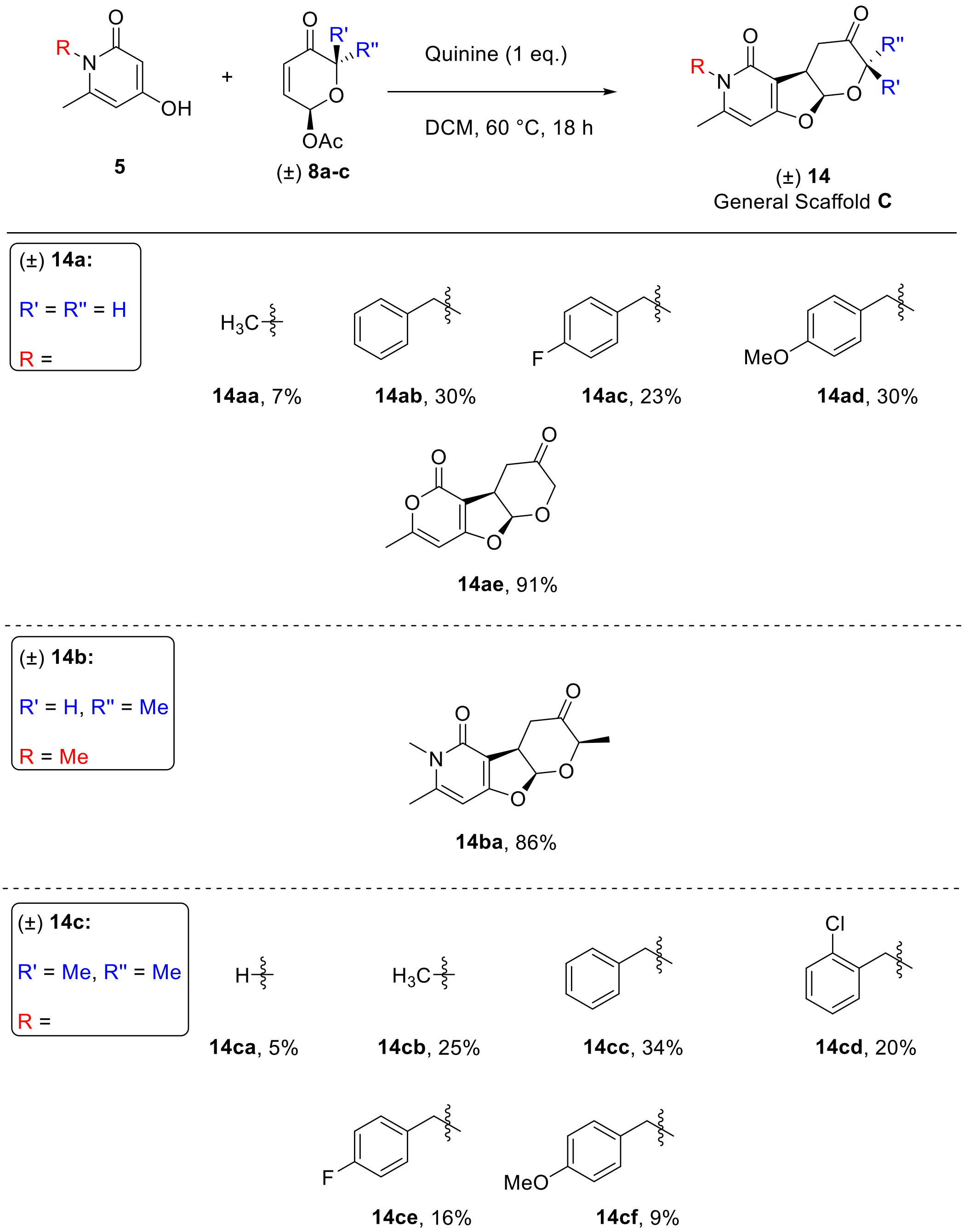


**Scheme S10.** Synthesis of derivatives of general scaffold **C** by Michael-transacetalization cascade employing various pyridones **5** and dihydropyranones **8a-c**. For compound numbering the first digit indicates the general scaffold according to Scheme 1 in the main text, the first letter indicates a specific sub-scaffold (blue) and the second letter indicates consecutive derivatives (red).

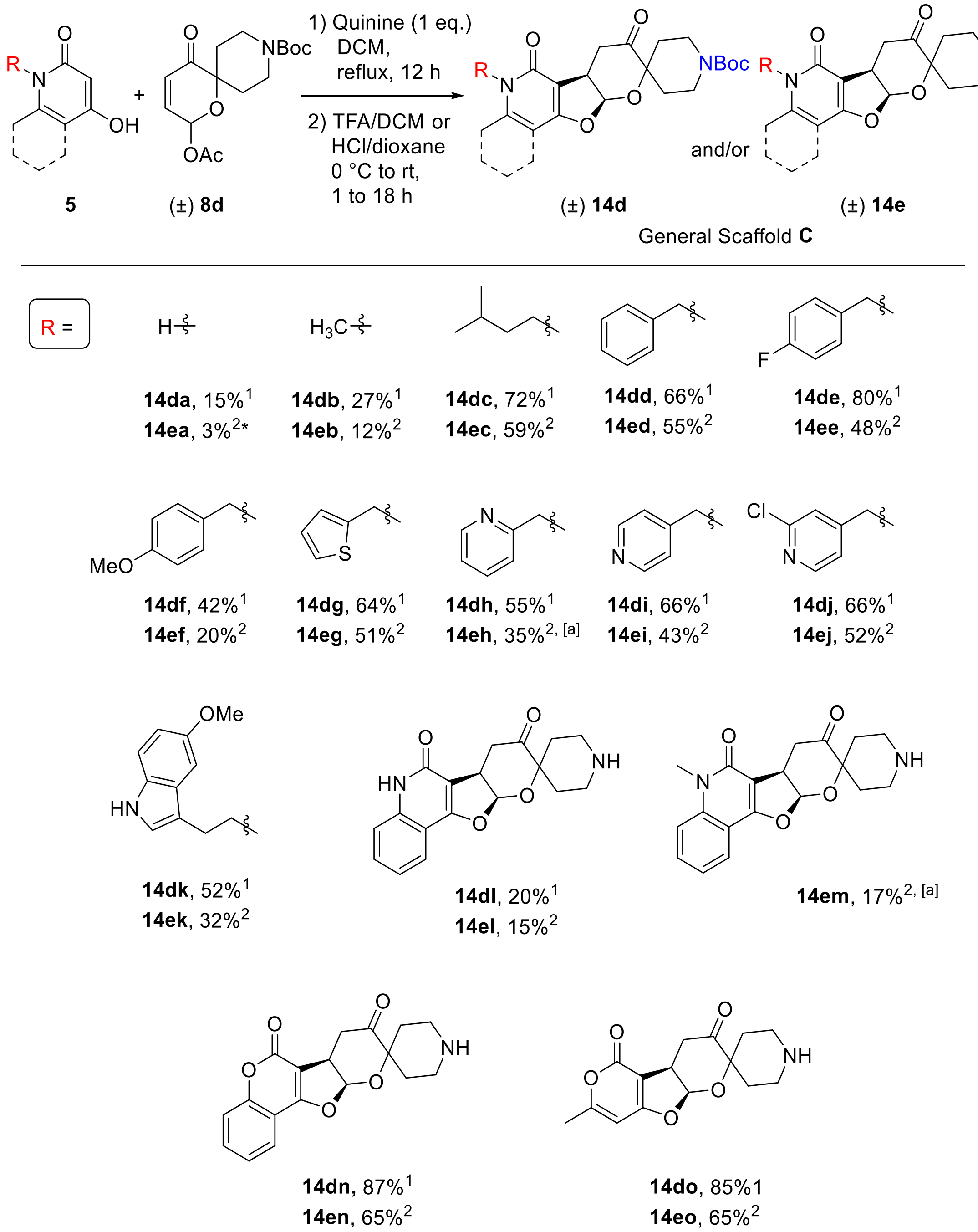


**Scheme S11.** Synthesis of Michael-transacetalization cascade products employing various pyridones **5** and dihydropayranone **8d**. 1) Yield for the Michael-transacetalization cascade step. 2) Yield after the Boc-deprotection over two steps. [a] Isolated as TFA-salt after purification with prep. HPLC. For compound numbering the first digit indicates the general scaffold according to Scheme 1 in the main text, the first letter indicates a specific sub-scaffold (blue) and the second letter indicates consecutive derivatives (red).

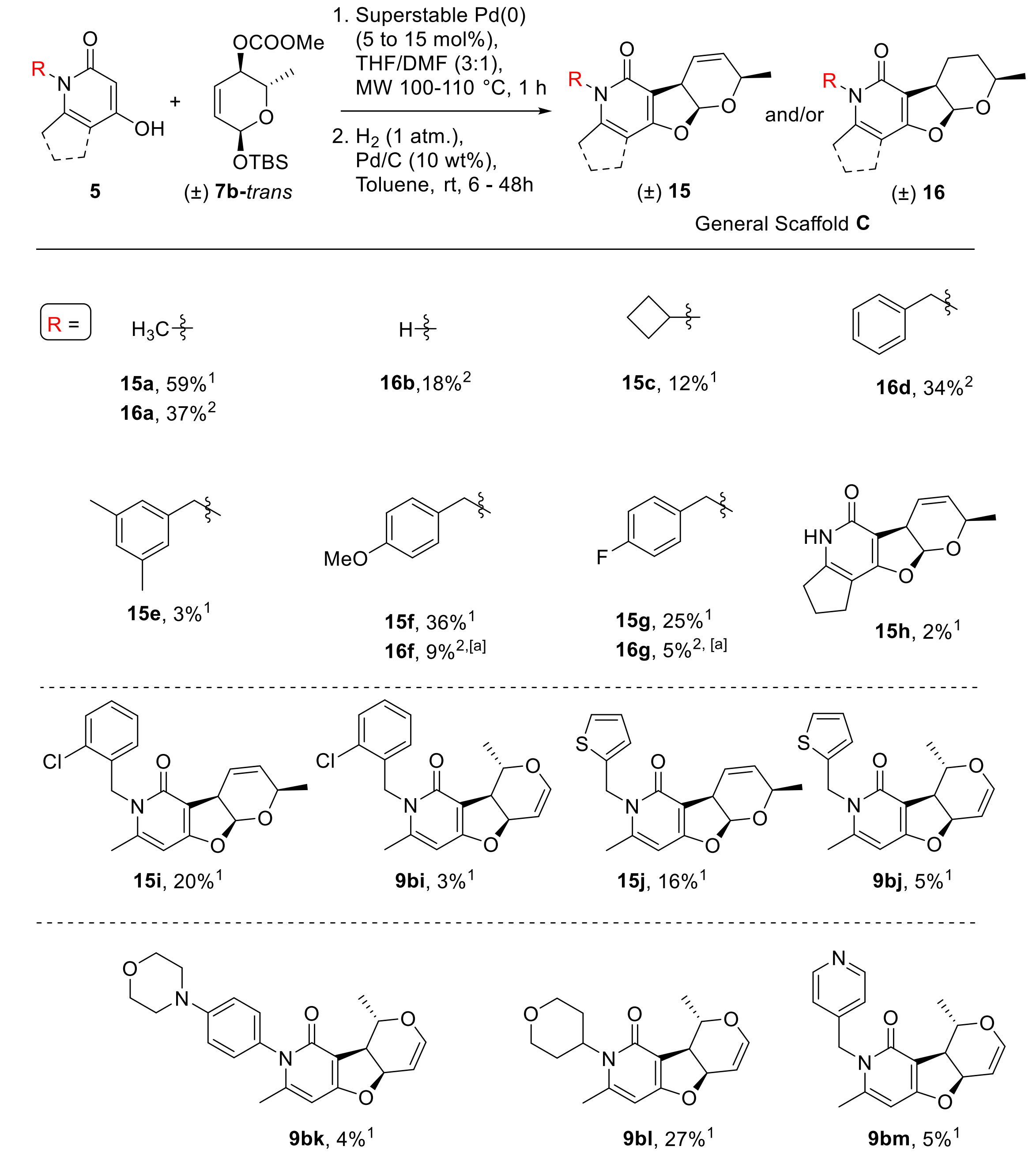


**Scheme S12.** Synthesis of PFP cyclic acetal isomers **15** and **16** through a superstable Pd(0) catalyzed Tsuji-Trost transacetalization cascade. 1) Yield for the Tsuji-Trost transacetalization cascade step. 2) Yield after the heterogenous reduction over two steps. [a] Pd-tetrakis was used as a catalyst. For compound numbering the first digit indicates the general scaffold according to Scheme 1 in the main text, the first letter indicates indicates consecutive derivatives (red).

### 1.3.1. General Procedures

General procedure 2 (GP2): An oven-dried microwave vial was loaded with 5 mol% superstable Pd(0) catalyst[54] ($Pd[P[3,5-(CF_3)_2C_6H_3]_3]_3$) and the bis-electrophile under Argon atmosphere. THF (0.2 M) was added and the vial was sealed. After stirring for 10 minutes the bis-nucleophile was added (for pyridones: DMF was added subsequently to afford a 3:1 mixture of

THF/DMF with a final concentration of 0.1 M). The sealed vial was then subjected for microwave irradiation (200 W, 100 to 110 °C, 30 to 60 minutes). The reaction mixture was concentrated in vacuo, immobilized on isolute and purified by FC or MPLC.

General procedure 3 (GP3):[49] An oven-dried schlenck-tube was filled with Argon, charged with 10 mol% Pd-tetrakis, evacuated and then back-filled with Argon. The bis-electrophile was dissolved in toluene (0.1 M) in a separate vessel under Argon atmosphere and then added to the Pd-catalyst and allowed to stir for 20 min before the bis-nucleophile was added (for pyridones: DMF was added subsequently to afford a 3:1 mixture of toluene/DMF with a final concentration of 0.05 M). After stirring for 3-6 h at room temperature, additional 10 mol% Pd-catalyst were added to the reaction mixture. The mixture was then allowed to stir at room temperature overnight, filtered through celite and concentrated in vacuo. The crude was immobilized on isolute and purified by FC or MPLC.

General procedure 4 (GP4): The glycal substrate was dissolved in DMF (0.1 M) and $Pd(OAc)_2$ together with boronic acid were added. The mixture was stirred overnight, filtered through a short pad of silica and concentrated under reduced pressure. The crude was purified by MPLC.

General procedure 5 (GP5): The glycal substrate was dissolved in acetonitrile/$H_2O$ (1:1, 0.075 M) and NBS (1.5 eq.) was added at room temperature. The mixture was stirred overnight before being quenched by the addition of saturated $NaHCO_3$ and diluted with EtOAc. The layers were separated and the aqueous phase was extracted with EtOAc three times. The combined organic layers were washed with brine, dried over $MgSO_4$ and concentrated in vacuo. The crude was immobilized on isolute and purified by FC or MPLC. The hydrobromination product was dissolved in MeOH (0.1 M) and cooled to 0 °C. $NaBH_4$ (1.2 eq) was added and the reaction mixture was stirred at 0 °C for 30 minutes. The reaction was quenched by addition of acetone (1 mL) and the solvents were removed in vacuo. The crude was immobilized on isolute and purified by MPLC or prep. HPLC.

General procedure 6 (GP6):[112] An oven-dried schlenck-tube was filled with Argon, charged with 5 mol% Pd-tetrakis, evacuated and then back-filled with Argon. The bis-electrophile was dissolved in THF (0.15 M) in a separate vessel under Argon atmosphere and then added to the Pd-catalyst and allowed to stir for 20 min before the bis-nucleophile was added as a solution in DMF (0.4 M) and triethylamine (1 equiv). The mixture was then allowed to stir at room temperature overnight, before being quenched by addition of saturated $NaHCO_3$ solution. The mixture was diluted with EtOAc, the phases were separated, and the aqueous phase was

extracted with EtOAc three times. The combined organic layers were washed with brine, dried over $MgSO_4$ and concentrated in vacuo. The crude was immobilized on isolute and purified by FC or MPLC.

General procedure 7 (GP7):[112] To a stirred solution of the bis-electrophile in dry DCM (0.075 M) was added the bis-nucleophile and quinine (1 equiv). After stirring at 60 °C in a sealed vial for 18 h, the solvent was removed under reduced pressure and the crude was purified by MPLC.

### 1.3.2. Synthesis of General Scaffold A Derivatives

**(±) 3-methyl-5a,9a-dihydro-1H,9H-furo[3,2-c:4,5-c']dipyran-1-one (9aa)**[49]

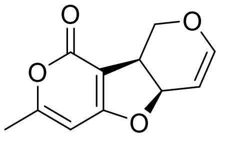

According to GP2, 7**a** (200.0 mg, 0.69 mmol) was reacted with 4-hydroxy-6-methyl-pyrone (86.9 mg, 0.69 mmol) at 100 °C for 1 hour. Purification by MPLC (cyclohexane/EtOAc) afforded the product as a white solid (122.4 mg, 86%). **$^1$H-NMR** (600 MHz, DMSO-d$_6$): δ 6.86 (d, *J* = 6.2 Hz, 1H,), 6.31 (s, 1H,), 5.24 (dd, *J* = 8.3 Hz, 4.3 Hz, 1H), 5.18 (dd, 6.2 Hz, 4.3 Hz, 1H), 4.14 (dd, 10.9 Hz, 4.7 Hz, 1H), 3.55 (dd 10.9 Hz, 9.0 Hz, 1H), 3.44 (td, 9.0 Hz, 8.3 Hz, 4.7 Hz, 1H), 2.22 (s, 3H). **HRMS**-ESI (m/z): $[M + H]^+$ calculated for $C_{11}H_{11}O_4^+$, 207.0652; found, 207.0653.

**(±) 3-methyl-5a,6,9,9a-tetrahydro-1H,7H-furo[3,2-c:4,5-c']dipyran-1-one (10aa); (±) 4-hydroxy-6-methyl-3-(tetrahydro-2H-pyran-3-yl)-2H-pyran-2-one (17aa)**

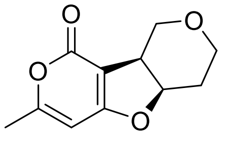

**10aa**

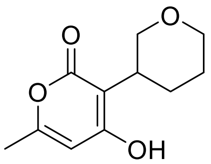

**17aa**

A suspension of **9aa** (13.5 mg, 0.07 mmol) and Pd/C (1.3 mg, 10 wt%) in toluene (0.7 mL) was hydrogenated at 20 °C for 7 hours using a $H_2$-balloon. The catalyst was filtered off and the solvent removed in vacuo. The crude was purified by prep. HPLC to afford the product **17aa** (5.0 mg, 37%) and **10aa** (7.6 mg, 55%) in separated fractions. **$^1$H-NMR** product **10aa** (700 MHz, $CDCl_3$): δ 5.96 (s, 1H), 4.99-4.95 (bm, 1H), 4.05 (dd, *J* = 11.8 Hz, 5.7 Hz, 1H), 3.81 (m, 1H), 3.65 (dd, *J* = 10.9 Hz, 10.9 Hz 1H), 3.54 (dd, *J* = 11.8 Hz, 7.4 Hz, 1H), 3.35 (dd, *J* = 13.4 Hz, 6.7 Hz, 1H), 2.27 (s, 3H) 2.21-2.09 (m, 2H).**$^{13}$C-NMR** product **10aa** (176 MHz, $CDCl_3$): δ 172.5, 166.0, 162.1, 102.4, 96.1, 82.9, 66.6, 62.7, 37.1, 26.7, 20.6. **HRMS**-ESI (m/z) product **10aa**: $[M + H]^+$ calculated for $C_{11}H_{13}O_4^+$, 209.0808; found, 209.0807. **$^1$H-NMR** product **17aa** (600 MHz, $CDCl_3$): δ 11.50 (s, 1H), 5.83 (s, 1H), 4.16 (bdd, *J* = 11.9 Hz, 4.3 Hz, 1H), 4.09 (bd, *J* = 12.3 Hz, 1H), 3.89 (dd, *J* = 12.3 Hz,

3.3 Hz, 1H), 3.64 (td, *J* = 12.7 Hz, 11.9 Hz, 2.4 Hz, 1H), 3.24 (m, 1H), 2.19 (s, 3H) 1.95 (bd, *J* = 13.9 Hz, 1H), 1.85 (tt, *J* = 13.9 Hz, 13.7 Hz, 4.4 Hz, 4.3 Hz, 1H), 1.69 (ddt, *J* = 20.3 Hz, 12.7 Hz, 4.3 Hz, 4.3 Hz, 1H), 1.56 (bd, *J* = 20.3 Hz, 1H). **$^{13}$C-NMR** product **17aa** (151 MHz, $CDCl_3$): δ 166.5, 166.2, 159.7, 102.8, 102.2, 70.2, 69.7, 33.0, 28.2, 23.0, 19.7. **HRMS**-ESI (m/z) product **17aa**: $[M + H]^+$ calculated for $C_{11}H_{15}O_4^+$, 211.0965; found, 211.0965.

**(±) 6b,9,10,10a-tetrahydro-6H,7H-pyrano[3',4':4,5]furo[3,2-c]chromen-6-one (10ab); (±) 4-hydroxy-3-(tetrahydro-2H-pyran-3-yl)-2H-chromen-2-one (17ab)**

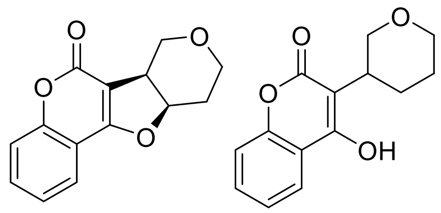


**10ab** **17ab**

According to GP3, **7a** (50.0 mg, 0.17 mmol) was reacted with 4-hydroxycoumarine (28.1 mg, 0.17 mmol). Purification by MPLC (cyclohexane/EtOAc 1:0 to 0:1) afforded the product (22.4 mg, 53%). The product was directly suspended with Pd/C (2.5 mg, 10 wt%) in toluene (1.5 mL) and was hydrogenated at 20 °C for 4 hours using a $H_2$-balloon. The catalyst was filtered off and the solvent removed in vacuo. The crude was purified by prep. HPLC to afford the product **10ab** (7.5 mg, 33%) and **17ab** (5.4 mg, 28%) in separated fractions. **$^{1}$H-NMR** product **10ab** (700 MHz, $CDCl_3$): δ 7.68 (dd, *J* = 7.9 Hz, 1.5 Hz, 1H) 7.58 (ddd, *J* = 8.5 Hz, 7.7 Hz, 1.5 Hz, 1H), 7.39 (d, *J* = 8.5 Hz, 1H), 7.30 (dd, *J* = 7.9 Hz, 7.7 Hz, 1H), 5.17 (dt, *J* = 10.9 Hz, 4.3 Hz, 4.3 Hz, 1H), 4.13 (dd, *J* = 12.1 Hz, 5.9 Hz, 1H), 3.87 (ddd, *J* = 11.3 Hz, 5.5 Hz, 4.0 Hz, 1H), 3.72 (ddd, *J* = 11.3 Hz, 10.4 Hz, 4.3 Hz, 1H), 3.65 (dd, *J* = 12.1 Hz, 7.4 Hz, 1H) 3.51 (dd, *J* = 10.9 Hz, 7.4 Hz, 1H), 2.30-2.21 (m, 2H). **$^{13}$C-NMR** product **10ab** (176 MHz, $CD_2Cl_2$): 167.9, 160.7, 155.2, 132.8, 124.2, 122.9, 117.3, 112.8, 105.2, 83.6, 66.5, 62.7, 38.2, 26.7. **HRMS**-ESI (m/z) product **10ab**: $[M + H]^+$ calculated for $C_{14}H_{13}O_4^+$, 245.0814; found, 245.0808. **$^{1}$H-NMR** product **17ab** (700 MHz, $CDCl_3$): δ 12.14 (s, 1H), 7.95 (dd, *J* = 7.9 Hz, 1.3 Hz, 1H) 7.52 (dd, *J* = 8.5 Hz, 8.5 Hz, 1H), 7.32-7.28 (m, 2H), 4.25 (dd, *J* = 11.6 Hz, 4.2 Hz, 1H), 4.22 (d, *J* = 12.5 Hz, 1H), 3.98 (d, *J* = 12.5 Hz, 3.2 Hz, 1H), 3.74-3.69 (m, 1H), 3.44-3.40 (m, 1H), 2.03 (d, *J* = 14.1 Hz, 1H), 1.98-1.92 (m, 1H), 1.77 (ddd, *J* = 14 Hz, 4.2 Hz, 4.2 Hz, 1H), 1.61 (bd, *J* = 14.0 Hz, 1H). **$^{13}$C-NMR** product **17ab** (176 MHz, $CD_2Cl_2$): 167.8, 161.8, 152.4, 131.8, 124.0, 123.9, 117.2, 116.3, 105.2, 70.2, 69.8, 33.5, 28.3, 22.9. **HRMS**-ESI (m/z) product **17ab**: $[M + H]^+$ calculated for $C_{14}H_{15}O_4^+$, 247.0965; found, 247.0967.

**(±) 7,8-dimethyl-8,9b-dihydro-1H-pyrano[3',4':4,5]furo[3,2-c]pyridin-9(4aH)-one (9ac)**

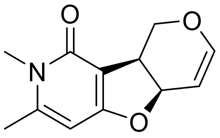

According to GP2, **7a** (1.00 g, 3.47 mmol) was reacted with **5b** (0.48 g, 3.47 mmol) at 110 °C for 1 hour. Purification by MPLC (DCM/MeOH 1:0 to 95:5) afforded the product (0.50 g, 66%). **$^1$H-NMR** (700 MHz, DMSO-$d_6$): δ 6.81 (d, *J* = 6.2 Hz, 1H), 6.00 (s, 1H), 5.16 (dd, *J* = 6.2 Hz, 4.3 Hz, 1H), 5.04 (dd, *J* = 7.7 Hz, 4.3 Hz, 1H), 4.16 (dd, *J* = 10.7 Hz, 4.8 Hz, 1H), 3.47 (dd, *J* = 10.2 Hz, 10.2 Hz, 1H), 3.39-3.32 (m, 4H), 2.32 (s, 3H). **$^{13}$C-NMR** (176 MHz, DMSO-$d_6$): δ 166.4, 160.7, 150.0, 149.5, 105.1, 99.7, 94.3, 76.2, 64.2, 37.5, 29.8, 20.7. **LCMS**-ESI (m/z): 220.16 $[M + H]^+$.

**(±) 7,8-dimethyl-4,4a,8,9b-tetrahydro-1H-pyrano[3',4':4,5]furo[3,2-c]pyridin-9(3H)-one (10ac)**

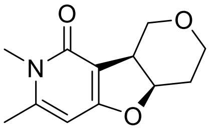

According to GP2, **7a** (50.0 mg, 0.17 mmol) was reacted with **5b** (24.1 mg, 0.17 mmol) at 110 °C for 1 hour. After filtration over celite the crude product was directly suspended with Pd/C (5 mg, 10 wt%) in toluene (1.5 mL) and was hydrogenated at 20 °C for 6 hours using a $H_2$-balloon. The catalyst was filtered off and the filtrate was diluted with EtOAc (10 mL) and washed with saturated $Na_2CO_3$ solution (15 mL). The aqueous phase was extracted with EtOAc (3 x 10 mL) and the combined organic layers were washed with brine (50 mL), dried over $MgSO_4$ and concentrated in vacuo. The crude was purified by MPLC (DCM/MeOH 1:0 to 95:5) to afford the product (8.3 mg, 21%). **$^1$H-NMR** (700 MHz, $CDCl_3$): δ 5.88 (s. 1H), 4.87-4.83 (m, 1H), 4.13 (dd, *J* = 11.4, 6.0 Hz, 1H), 3.84-3.80 (m, 1H), 3.63 (td, *J* = 11.4, 3.6 Hz, 1H), 3.50-3.44 (m, 4H), 3.41 (dd, *J* = 14.2, 7.2 Hz, 1H), 2.33 (s, 3H), 2.15 (ddd, *J* = 20.7, 10.6, 5.2 Hz, 1H), 2.08 (dq, *J* = 15.0, 3.3 Hz, 1H). **$^{13}$C-NMR** (176 MHz, $CDCl_3$): δ 167.5, 161.9, 148.5, 109.1, 95.7, 81.6, 67.4, 62.9, 38.2, 30.7, 27.0, 21.8. **HRMS**-ESI (m/z): $[M + H]^+$ calculated for $C_{12}H_{16}NO_3^+$, 222.1125; found, 222.1124.

**(±) 7-methyl-8,9b-dihydro-1H-pyrano[3',4':4,5]furo[3,2-c]pyridin-9(4aH)-one (9ad)**

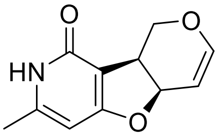

According to GP2, **7a** (20.0 mg, 0.07 mmol) was reacted with **5a** (8.68 mg, 0.07 mmol) at 110 °C for 1 hour. Purification by FC (DCM/MeOH 1:0 to 9:1) afforded the product (15.2 mg, 98%). **$^1$H-NMR** (700 MHz, $CD_2Cl_2$): δ 6.77 (d, *J* = 6.2 Hz, 1H), 5.95 (s, 1H), 5.33 (s, 1H), 5.20 (dd, *J* = 6.2 Hz, 4.4 Hz, 1H), 5.01 (dd, *J* = 7.0 Hz, 4.4 Hz, 1H), 4.32 (dd, *J* = 10.4 Hz, 4.5 Hz, 1H), 3.55 (dd, *J* = 10.3 Hz, 10.3 Hz, 1H), 3.52-3.49 (m, 1H), 2.31 (s, 3H). **$^{13}$C-NMR** (176 MHz, $CD_2Cl_2$): δ 171.5, 162.1, 150.5, 149.0, 106.9, 99.3, 96.8, 78.3, 65.0, 37.5, 19.6. **HRMS**-ESI (m/z): $[M + H]^+$ calculated for $C_{11}H_{12}O_3N^+$, 206.0812; found, 206.0821.

**(±) 7-methyl-4,4a,8,9b-tetrahydro-1H-pyrano[3',4':4,5]furo[3,2-c]pyridin-9(3H)-one (10ad)**

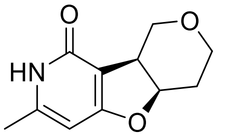

According to GP2, **7a** (50.0 mg, 0.17 mmol) was reacted with **5a** (22.0 mg, 0.17 mmol) at 110 °C for 1 hour. After filtration over celite the crude was purified by FC (EtOAc/MeOH 1:0 to 9:1) and the product was directly suspended with Pd/C (5 mg, 10 wt%) in toluene (2 mL) and was hydrogenated at 20 °C for 6 hours using a $H_2$-balloon. The catalyst was filtered off and the solvent removed in vacuo. The crude was purified by MPLC (EtOAc/MeOH 1:0 to 9:1) to afford the product (3.6 mg, 10%). $^1$**H-NMR** (700 MHz, $CD_2Cl_2$): δ 11.80 (s, 1H), 5.82 (s, 1H), 4.89-4.85 (m, 1H), 4.01 (dd, *J* = 11.8, 6.0 Hz, 1H), 3.77-3.73 (m, 1H), 3.61 (td, *J* = 10.9, 3.8 Hz, 1H), 3.52 (dd, *J* = 11.8, 7.5 Hz, 1H), 3.34 (dd, *J* = 13.8, 7.5 Hz, 1H), 2.27 (s, 3H), 2.12 (ddt, *J* = 15.2, 10.3, 5.2 Hz, 1H), 2.04 (dq, *J* = 14.9, 3.8 Hz, 1H). $^{13}$**C-NMR** (176 MHz, $CD_2Cl_2$): δ 170.7, 163.5, 148.2, 109.0, 95.0, 82.0, 67.2, 63.2, 38.0, 27.5, 19.5. **HRMS**-ESI (m/z): $[M + H]^+$ calculated for $C_{11}H_{14}NO_3^+$, 208.0968; found, 208.0970.

**(±) 8-cyclobutyl-7-methyl-8,9b-dihydro-1H-pyrano[3',4':4,5]furo[3,2-c]pyridin-9(4aH)-one (9ae)**

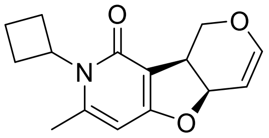

According to GP2, **7a** (50.0 mg, 0.17 mmol) was reacted with **5e** (31.1 mg, 0.17 mmol) at 110 °C for 30 minutes. Purification by MPLC (Pet. Ether/EtOAc 1:0 to 0:1) afforded the product (33.4 mg, 74%). $^1$**H-NMR** (700 MHz, $CD_2Cl_2$): δ 6.75 (d, *J* = 6.2 Hz, 1H),5.74 (s, 1H), 5.17 (dd, *J* = 6.2, 4.5 Hz, 1H), 4.96 (dd, *J* = 7.5, 4.5 Hz, 1H), 4.72 (p, *J* = 8.8 Hz, 1H), 4.34 (dd, *J* = 10.6, 5.0 Hz, 1H), 3.51 (t, *J* = 10.6 Hz, 1H), 3.45-3.40 (m, 1H), 3.26-3.19 (m, 2H), 2.28-2.18 1.73 (m, 2H), 1.96-1.90 (m, 1H), 1.73 (dq, *J* = 18.3, 9.2 Hz, 1H). $^{13}$**C-NMR** (176 MHz, $CD_2Cl_2$): δ 167.2, 163.5, 150.2, 149.1, 108.2, 99.7, 96.1, 77.2, 65.4, 53.2, 38.6, 28.4, 28.1, 22.5, 15.1. **LC-MS**-ESI (m/z): 260.16 $[M + H]^+$.

**(±) 8-cyclobutyl-7-methyl-4,4a,8,9b-tetrahydro-1H-pyrano[3',4':4,5]furo[3,2-c]pyridin-9(3H)-one (10ae)**

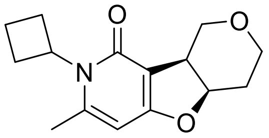

A suspension of **9ae** (10.0 mg, 0.04 mmol) and Pd/C (1.0 mg, 10 wt%) in toluene (0.5 mL) was hydrogenated at 20 °C for 6 hours using a $H_2$-balloon. The catalyst was filtered off and the solvent removed in vacuo. The crude was purified by prep. HPLC to afford product (2.3 mg, 23%). $^1$**H-NMR** (700 MHz,

$CDCl_3$): δ 6.01 (s, 1H), 4.93 (s,10H), 4.81-4.76 (m, 1H), 4.15 (dd, *J* = 11.9, 6.0 Hz, 1H), 3.89-3.84 (m, 1H), 3.67-3.60 (m, 2H), 3.43 (dd, *J* = 11.9, 7.8 Hz, 1H), 3.20 (dp, *J* = 14.8, 9.9 Hz, 2H), 2.40 (s, 3H), 2.37-2.27 (m, 4H), 2.18 (ddd, *J* = 15.8, 10.5, 5.0 Hz, 1H), 2.11 (dd, *J* = 15.2, 2.6 Hz, 1H), 2.01 (q, *J* = 11.1 Hz, 1H), 1.76 (dt, *J* = 19.2, 9.4 Hz, 2H). **HRMS**-ESI (m/z): [M + H]$^+$ calculated for $C_{15}H_{20}NO_3^+$, 262.1438; found, 262.1439.

**(±) 7-methyl-8-(tetrahydro-2H-pyran-4-yl)-4,4a,8,9b-tetrahydro-1H-pyrano[3',4':4,5]furo[3,2-c]pyridin-9(3H)-one (10af)**

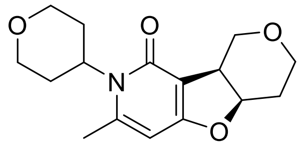

According to GP2, **7a** (75.0 mg, 0.26 mmol) was reacted with **5g** (40.8 mg, 0.20 mmol) at 100 °C for 1 hour. After purification by FC (Hep/EtOAc 1:0 to 1:1) the product was directly suspended with Pd/C (5 mg, 10 wt%) in THF (1.5 mL) and was hydrogenated at 20 °C for 2 hours using a $H_2$-balloon. The catalyst was filtered off and the solvent removed in vacuo. The crude was purified by FC (EtOAc/MeOH 1:0 to 9:1) to afford the product (7.4 mg, 10%). **$^1$H-NMR** (700 MHz, $CD_2Cl_2$): δ 5.81 (s, 1H), 4.84-4.81 (m, 1H), 4.03 (dd, *J* = 11.5, 4.6 Hz, 2H), 3.99 (dd, *J* = 11.8, 5.9 Hz, 1H), 3.75-3.71 (m, 1H), 3.61 (td, *J* = 10.5, 3.8 Hz, 1H), 3.54 (dd, *J* = 11.8, 7.5 Hz, 1H), 3.42-3.37 (m, 2H), 3.31 (dd, *J* = 13.7, 7.5 Hz, 1H), 2.37 (s, 3H), 2.10 (ddt, *J* = 15.2, 10.5, 5.2 Hz, 1H), 2.06.1.99 (m, 2H), 1.54-1.45 (m, 2H), 1.34-1.25 (m, 2H). **$^{13}$C-NMR** (176 MHz, $CD_2Cl_2$): δ 167.2, 162.8, 148.3, 96.7, 81.8, 68.4, 67.1, 63.1, 39.0, 29.4, 27.5, 22.6. **HRMS**-ESI (m/z): [M + H]$^+$ calculated for $C_{16}H_{22}NO_4^+$, 292.1543; found, 292.1547.

**(±) 7-methyl-8-(4-morpholinophenyl)-8,9b-dihydro-1H-pyrano[3',4':4,5]furo[3,2-c]pyridin-9(4aH)-one (9ag)**

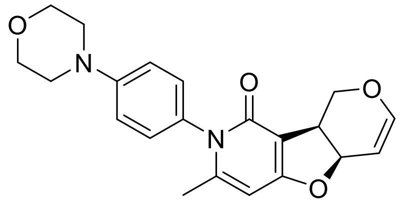

According to GP2, **7a** (50.0 mg, 0.17 mmol) was reacted with **5f** (49.6 mg, 0.17 mmol) at 110 °C for 30 minutes. Purification by FC (Pet. Ether/EtOAc 1:0 to 95:5 + 1% $NEt_3$) afforded the product (21.0 mg, 33%). **$^1$H-NMR** (700 MHz, $CD_2Cl_2$): δ 7.07-6.97 (m, 4H), 6.78 (d, *J* = 6.2 Hz, 1H), 5.91 (s, 1H), 5.21 (dd, *J* = 6.2, 4.5 Hz, 1H), 5.04 (dd, *J* = 7.6, 4.5 Hz, 1H), 4.33 (dd, *J* = 10.8, 4.9 Hz, 1H), 3.86-3.82 (m, 4H), 3.55 (t, *J* = 10.8 Hz, 1H), 3.47 (ddd, *J* = 10.8, 7.6, 5.1 Hz, 1H), 3.22-3.19 (m, 4H), 1.94 (s, 1H). **$^{13}$C-NMR** (176 MHz, $CD_2Cl_2$): δ 168.2, 162.5, 151.7, 150.3, 150.2, 130.8, 129.3, 129.2, 116.1, 106.9, 99.6, 95.5, 77.5, 67.2, 65.4, 49.3, 38.5, 22.5. **HRMS**-ESI (m/z): [M + H]$^+$ calculated for $C_{21}H_{23}N_2O_4^+$, 367.1652; found, 367.1649.

**(±) 7-methyl-8-(4-morpholinophenyl)-4,4a,8,9b-tetrahydro-1H-pyrano[3',4':4,5]furo[3,2-c]pyridin-9(3H)-one (10ag)**

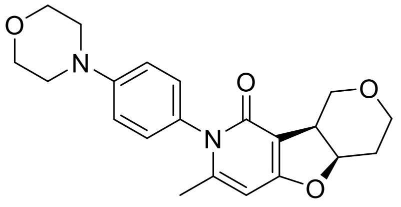

According to GP2, **7a** (50.0 mg, 0.17 mmol) was reacted with **5f** (49.6 mg, 0.17 mmol) at 110 °C for 1 hour. After filtration over celite the crude was purified by FC (Hep/EtOAc 1:0 to 0:1) and the product was directly suspended with Pd/C (5 mg, 10 wt%) in toluene (2 mL) and was hydrogenated at 20 °C for 6 hours using a $H_2$-balloon. The catalyst was filtered off and the filtrate was diluted with EtOAc (10 mL) and washed with saturated $Na_2CO_3$ solution (15 mL). The aqueous phase was extracted with EtOAc (3 x 10 mL) and the combined organic layers were washed with brine (50 mL), dried over $MgSO_4$ and concentrated in vacuo. The crude was purified by MPLC (EtOAc/MeOH 1:0 to 9:1 + 0.1% DIPEA) to afford the product (2.0 mg, 3%). **$^1$H-NMR** (700 MHz, $CD_2Cl_2$): δ 7.07-6.97 (m 4H), 5.93 (s, 1H), 4.92-4.89 (m, 1H), 4.00 (dd, *J* = 11.8, 5.9 Hz, 1H), 3.86-3.82 (m, 4H), 3.78-3.74 (m, 1H), 3.66 (td, *J* = 10.8, 3.9 Hz, 1H), 3.58 (dd, *J* = 11.8, 7.4 Hz, 1H), 3.35 (dd, *J* = 13.6, 7.4 Hz, 1H), 3.21-3.19 (m, 4H), 2.17-2.11 (m, 1H), 2.06 (dq, *J* = 14.9, 3.9 Hz, 1H), 1.94 (s, 1H). **$^{13}$C-NMR** (176 MHz, $CD_2Cl_2$): δ 168.4, 162.6, 151.6, 149.8, 131.0, 129.4, 129.2, 116.1, 116.1, 109.2, 95.4, 82.0, 67.2, 67.1, 63.2, 49.3, 38.8, 27.5, 22.5. **HRMS**-ESI (m/z): $[M + H]^+$ calculated for $C_{21}H_{25}N_2O_4^+$, 369.1809; found, 369.1816.

**(±) 8-benzyl-7-methyl-8,9b-dihydro-1H-pyrano[3',4':4,5]furo[3,2-c]pyridin-9(4aH)-one (9ah)**[49]

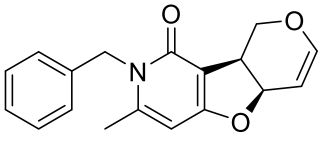

According to GP3, **7a** (50.0 mg, 0.17 mmol) was reacted with **5c** (37.3 mg, 0.17 mmol) in THF (2 mL + 24 µL $NEt_3$) for 2 days at room temperature. Purification by FC (Hep/EtOAc 4:1 to 1:1 + 1% $NEt_3$) afforded the product (24.1 mg, 48%). **$^1$H-NMR** (600 MHz, DMSO-$d_6$): δ 7.33 (t, *J* = 7.5 Hz, 2H), 7.25 (t, *J* = 7.5 Hz, 1H), 7.10 (d, *J* = 7.5 Hz, 2H), 6.84 (d, *J* = 6.2 Hz, 1H), 6.05 (s, 1H), 5.37 (d, *J* = 15.8 Hz, 1H), 5.19 (dd, *J* = 6.2 Hz, 4.3 Hz, 1H), 5.12 (d, *J* = 15.8 Hz, 1H), 5.10 (dd, *J* = 7.5 Hz, 4.3 Hz, 1H), 4.20 (dd, *J* = 10.5 Hz, 4.7 Hz, 1H), 3.52 (dd, *J* = 10.2 Hz, 10.2 Hz, 1H), 3.47-3.42 (m, 1H), 2.22 (s, 3H). **$^{13}$C-NMR** (151 MHz, DMSO-$d_6$): δ 166.8, 160.9, 149.8, 149.7, 137.4, 128.7, 127.0, 126.1, 105.3, 99.7, 95.2, 76.4, 64.2, 45.6, 37.6, 20.4. **LCMS**-ESI (m/z): 296.23 $[M + H]^+$.

**(±) 8-benzyl-7-methyl-4,4a,8,9b-tetrahydro-1H-pyrano[3',4':4,5]furo[3,2-c]pyridin-9(3H)-one (10ah)**

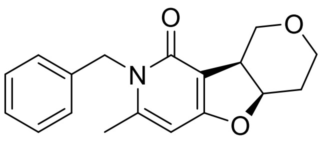

A suspension of **9ah** (75.2 mg, 0.25 mmol) and Pd/C (7.5 mg, 10 wt%) in toluene (2 mL) was hydrogenated at 20 °C for 6 hours using a $H_2$-balloon. The catalyst was filtered off and the solvent removed in vacuo. The crude was purified by prep. HPLC to afford the product (37.0 mg, 49%). **$^1$H-NMR** (700 MHz, $CD_2Cl_2$): δ 7.33 (t, *J* = 7.5 Hz, 2H), 7.28 (t, *J* = 7.5 Hz, 1H), 7.11 (d, *J* = 7.5 Hz, 2H), 6.10 (s, 1H), 5.45 (d, *J* = 15.6 Hz, 1H), 5.25 (d, *J* = 15.6 Hz, 1H), 4.96 (dt, *J* = 7.3, 3.8 Hz, 1H), 4.09 (dd, *J* = 11.5, 5.8 Hz, 1H), 3.83 (ddd, *J* = 11.0, 5.6, 2.9 Hz, 1H), 3.63 (td, *J* = 11.0, 3.8 Hz, 1H), 3.47 (dd, *J* = 13.9, 7.3 Hz, 1H), 3.43 (dd, *J* = 11.5, 8.1 Hz, 1H), 2.31 (s, 3H), 2.18 (ddd, *J* = 21.1, 10.6, 5.2 Hz, 1H), 2.11 (dq, *J* = 15.1, 3.1 Hz, 1H). **$^{13}$C-NMR** (176 MHz, $CD_2Cl_2$): δ 169.7, 162.4, 150.3, 136.6, 129.2, 127.9, 126.6, 110.2, 98.4, 83.0, 67.1, 63.0, 47.8, 38.3, 26.9, 21.5. **HRMS**-ESI (m/z): $[M + H]^+$ calculated for $C_{18}H_{20}NO_3^+$, 298.1438; found, 298.1430.

**(±) 8-(4-methoxybenzyl)-7-methyl-8,9b-dihydro-1H-pyrano[3',4':4,5]furo[3,2-c]pyridin-9(4aH)-one (9ai)**

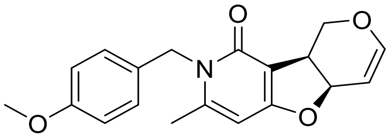

According to GP2, **7a** (28.0 mg, 0.10 mmol) was reacted with **5d** (23.8 mg, 0.10 mmol) at 100 °C for 1 hour. Purification by FC (Hep/EtOAc 1:0 to 1:1) afforded the product (24.3 mg, 77%). **$^1$H-NMR** (700 MHz, $CD_2Cl_2$): δ 7.07 (d, *J* = 8.7 Hz, 2H), 6.85 (d, *J* = 8.7 Hz, 2H), 6.79 (d, *J* = 6.2 Hz, 1H), 5.97 (s, 1H), 5.34 (d, *J* = 16.4 Hz, 1H), 5.22 (dd, *J* = 6.2 Hz, 4.3 Hz, 1H), 5.14 (d, *J* = 16.4 Hz, 1H), 5.06 (dd, *J* = 7.1 Hz, 4.3 Hz, 1H), 4.39 (dd, *J* = 9.9 Hz, 4.3 Hz, 1H), 3.55-3.47 (m, 2H), 2.29 (s, 3H). **$^{13}$C-NMR** (176 MHz, $CD_2Cl_2$): δ 168.7, 162.3, 159.4, 150.5, 150.2, 128.9, 128.1, 114.5, 107.5, 99.2, 97.5, 78.0, 65.2, 55.7, 46.9, 38.2, 21.5. **HRMS**-ESI (m/z): $[M + H]^+$ calculated for $C_{19}H_{20}O_4N^+$, 326.1387; found, 326.1387.

**(±) 8-(4-methoxybenzyl)-7-methyl-4,4a,8,9b-tetrahydro-1H-pyrano[3',4':4,5]furo[3,2-c]pyridin-9(3H)-one (10ai)**

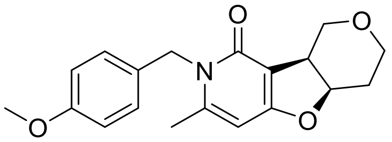

According to GP2, **7a** (50.0 mg, 0.17 mmol) was reacted with **5d** (42.5 mg, 0.17 mmol) at 110 °C for 1 hour. After filtration over celite the crude was suspended with Pd/C (5 mg, 10 wt%) in toluene (1.7 mL) and was hydrogenated at 20 °C for 6 hours using a $H_2$-balloon. The catalyst

was filtered off and the filtrate was diluted with EtOAc (10 mL) and washed with saturated $Na_2CO_3$ solution (15 mL). The aqueous phase was extracted with EtOAc (3 x 10 mL) and the combined organic layers were washed with brine (50 mL), dried over $MgSO_4$ and concentrated in vacuo. The crude was purified by MPLC (cyclohexane/EtOAc 1:0 to 0:1) to afford the product (7.2 mg, 13%). **$^1$H-NMR** (600 MHz, $CD_2Cl_2$): δ 7.07 (d, *J* = 8.7 Hz, 2H), 6.84 (d, *J* = 8.7 Hz, 2H), 5.86 (s, 1H), 5.29 (d, *J* = 15.4 Hz, 1H), 5.07 (d, *J* = 15.4 Hz, 1H), 4.91-4.87 (m, 1H), 4.05 (dd, *J* = 11.7, 5.9 Hz, 1H), 3.79-3.75 (m, 4H), 3.63 (td, *J* = 10.9, 3.7 Hz, 1H), 3.54 (dd, *J* = 11.7, 7.5 Hz, 1H), 3.39 (dd, *J* = 13.7, 7.5 Hz, 1H), 2.25 (s, 3H), 2.17-2.11 (m, 1H), 2.08-2.02 (m, 1H). **$^{13}$C-NMR** (151 MHz, $CD_2Cl_2$): δ 170.0, 162.1, 159.2, 149.3, 129.6, 128.1, 114.4, 109.2, 96.1, 82.0, 67.3, 63.1, 55.6, 46.3, 38.7, 27.5, 21.5. **HRMS**-ESI (m/z): $[M + H]^+$ calculated for $C_{19}H_{22}NO_4^+$, 328.1543; found, 328.1544.

**(±) 8-(4-fluorobenzyl)-7-methyl-4,4a,8,9b-tetrahydro-1H-pyrano[3',4':4,5]furo[3,2-c]pyridin-9(3H)-one (10aj)**

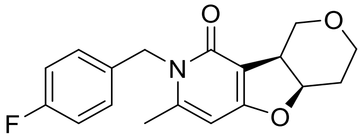

According to GP2, **7a** (75.9 mg, 0.26 mmol) was reacted with **5k** (45.0 mg, 0.20 mmol) at 100 °C for 1 hour. After filtration over celite the crude was purified by FC (Hep/EtOAc 1:0 to 1:1) and the product was directly suspended with Pd/C (5 mg, 10 wt%) in toluene (2 mL) and was hydrogenated at 20 °C for 9 hours using a $H_2$-balloon. The catalyst was filtered off and the solvent removed in vacuo. The crude was purified by MPLC (EtOAc/MeOH 1:0 to 9:1 + 0.1% DIPEA) to afford the product (24.7 mg, 40%). **$^1$H-NMR** (600 MHz, $CD_2Cl_2$): δ 7.13 (dd, *J* = 8.7, 5.4 Hz, 2H), 7.01 (t, *J* = 8.7 Hz, 2H), 5.88 (s, 1H), 5.35 (d, *J* = 15.4 Hz, 1H), 5.11 (d, *J* = 15.4 Hz, 1H), 4.92-4.88 (m, 1H), 4.04 (dd, *J* = 11.7, 5.9 Hz, 1H), 3.79-3.74 (m, 1H), 3.63 (td, *J* = 10.8, 3.8 Hz, 1H), 3.56 (dd, *J* = 11.7, 7.5 Hz, 1H), 3.39 (dd, *J* = 13.7, 7.5 Hz, 1H), 2.24 (s, 3H), 2.14 (ddd, *J* = 20.5, 10.3, 5.2 Hz, 1H), 2.05 (dq, *J* = 15.0, 3.8 Hz, 1H). **$^{13}$C-NMR** (151 MHz, $CD_2Cl_2$): δ 168.1, 163.2, 161.5, 161.9, 149.1, 133.6, 128.6, 128.5, 115.9, 115.8, 109.3, 96.3, 82.0, 67.2, 63.1, 46.1, 38.8, 27.4, 21.4. **HRMS**-ESI (m/z): $[M + H]^+$ calculated for $C_{18}H_{19}FNO_3^+$, 316.1344; found, 316.1348.

**(±) 7-methyl-8-(thiophen-2-ylmethyl)-4,4a,8,9b-tetrahydro-1H-pyrano[3',4':4,5]furo[3,2-c]pyridin-9(3H)-one (10ak)**

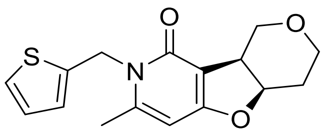

According to GP2, **7a** (50.0 mg, 0.17 mmol) was reacted with **5j** (43.2 mg, 0.17 mmol) at 100 °C for 1 hour. After filtration over celite the crude was purified by FC (Hep/EtOAc 1:0 to 1:1) and the

product was directly suspended with Pd/C (5 mg, 10 wt%) in toluene (2 mL) and was hydrogenated at 20 °C for 14 hours using a $H_2$-balloon. The catalyst was filtered off and the solvent removed in vacuo. The crude was purified by MPLC (cyclohexane/EtOAc 1:0 to 0:1) to afford the product (9.7 mg, 16%). **$^1$H-NMR** (700 MHz, $CD_2Cl_2$): δ 7.22 (dd, *J* = 5.1, 1.0 Hz, 1H), 6.97 (dd, *J* = 3.4, 1.0 Hz, 1H), 6.93 (dd, *J* = 5.1, 3.4 Hz, 1H), 5.85 (s, 1H), 5.43 (d, *J* = 15.4 Hz, 1H), 5.24 (d, *J* = 15.4 Hz, 1H), 4.89-4.85 (m, 1H), 4.04 (dd, *J* = 11.8, 5.9 Hz, 1H), 3.78-3.73 (m, 1H), 3.61 (td, *J* = 10.6, 3.8 Hz, 1H), 3.55 (dd, *J* = 11.8, 7.5 Hz, 1H), 3.38 (dd, *J* = 13.7, 7.5 Hz, 1H), 2.40 (s, 3H), 2.12 (ddt, *J* = 15.2, 10.6, 5.2 Hz, 1H), 2.05-2.00 (m, 1H). **$^{13}$C-NMR** (176 MHz, $CD_2Cl_2$): δ 168.3, 161.7, 148.7, 140.2, 126.9, 126.6, 125.8, 109.3, 96.2, 82.0, 67.1, 63.1, 42.5, 38.7, 27.4, 21.2. **HRMS**-ESI (m/z): $[M + H]^+$ calculated for $C_{16}H_{18}NO_3S^+$, 304.1002; found, 304.1003.

**(±) 7-methyl-8-(pyridin-2-ylmethyl)-4,4a,8,9b-tetrahydro-1H-pyrano[3',4':4,5]furo[3,2-c]pyridin-9(3H)-one (10al)**

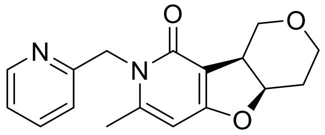


According to GP2, **7a** (63.5 mg, 0.22 mmol) was reacted with **5m** (43.2 mg, 0.20 mmol) at 100 °C for 18 hours. After filtration over celite the crude was purified by MPLC (cyclohexane/EtOAc 1:0 to 0:1 + 0.1% $NEt_3$) and an aliquot of the product (10 mg, 0.03 mmol) was directly suspended with Pd/C (5 mg, 10 wt%) in toluene (2 mL) and was hydrogenated at 20 °C for 24 hours using a $H_2$-balloon. The catalyst was filtered off and the solvent removed in vacuo and the crude was purified by prep. HPLC to afford the product (3.8 mg, 38%). **$^1$H-NMR** (600 MHz, $CDCl_3$): δ 8.73 (dt, *J* = 5.5, 1.6 Hz, 1H), 8.05 (td, *J* = 7.8, 1.6 Hz, 1H), 7.57 (ddd, *J* = 7.8, 5.5, 1.2 Hz, 1H), 7.44 (d, *J* = 7.8 Hz, 1H), 6.06 (s, 1H), 5.70 (d, *J* = 16.7 Hz, 1H), 5.60 (d, *J* = 16.7 Hz, 1H), 4.97 (dt, *J* = 7.5, 3.8 Hz, 1H), 4.11 (dd, *J* = 11.3, 5.4 Hz, 1H), 3.86 (ddd, *J* = 11.3, 5.8, 3.2 Hz, 1H), 3.67 (td, *J* = 11.3, 3.8 Hz, 1H), 3.54-3.45 (m, 2H), 2.36 (s, 3H), 2.20 (dddd, *J* = 15.2, 10.5, 5.8, 4.5 Hz, 1H), 2.13 (dq, *J* = 15.2, 3.8 Hz, 1H). **$^{13}$C-NMR** (151 MHz, $CDCl_3$): δ 169.5, 161.9, 154.3, 149.2, 145.0, 142.4, 124.5, 123.9, 109.8, 98.2, 82.5, 66.7, 62.7, 46.4, 38.0, 26.7, 21.6. **HRMS**-ESI (m/z): $[M + H]^+$ calculated for $C_{17}H_{19}N_2O_3^+$, 299.1390; found, 299.1392.

**(±) 7-methyl-8-(pyridin-4-ylmethyl)-4,4a,8,9b-tetrahydro-1H-pyrano[3',4':4,5]furo[3,2-c]pyridin-9(3H)-one and (±) 4-hydroxy-6-methyl-1-(pyridin-4-ylmethyl)-3-(tetrahydro-2H-pyran-3-yl)pyridin-2(1H)-one (10am)**

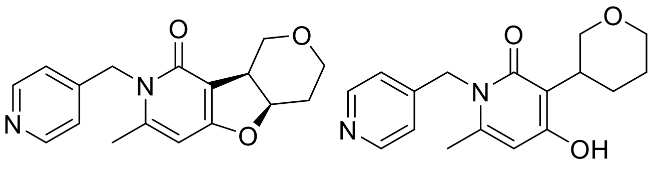

**10am**

According to GP2, **7a** (50.0 mg, 0.17 mmol) was reacted with **5i** (37.5 mg, 0.17 mmol) at 110 °C for 1 hour. After filtration over celite the crude was purified by FC (Hep/EtOAc 1:0 to 1:1) and the product was directly suspended with Pd/C (5 mg, 10 wt%) in toluene (2 mL) and was hydrogenated at 20 °C for 12 hours using a $H_2$-balloon. The catalyst was filtered off and the solvent removed in vacuo. The crude was purified by MPLC (EtOAc/MeOH 1:0 to 4:1 + 0.1% DIPEA) to afford an inseparable mixture of ring-closed and ring-opened products (ratio of 2:1 by NMR, 15.0 mg, 30%). **$^1$H-NMR** ring-closed product (500 MHz, $CDCl_3$): δ 8.51 (d, *J* = 6.0 Hz, 2H), 7.06 (d, *J* = 6.0 Hz, 2H), 6.13 (s, 1H), 5.35 (d, *J* = 17.0 Hz, 1H), 5.17 (d, *J* = 17.0 Hz, 2H), 4.99-4.95 (m, 1H), 3.85 (dd, *J* = 11.5, 5.6 Hz, 1H), 3.67 (dt, *J* = 10.1, 4.9 Hz, 1H), 3.53 (dd, *J* = 11.5, 6.6 Hz, 1H), 3.51-3.48 (m, 1H), 3.30 (dd, *J* = 13.6, 6.6 Hz, 1H), 2.21 (s, 3H), 2.09-2.05 (m, 1H), 1.92 (dq, *J* = 12.8, 4.1 Hz, 1H). **$^{13}$C-NMR** ring-closed product (126 MHz, $CDCl_3$): δ 167.3, 160.7, 149.8, 149.2, 146.7, 121.2, 107.7, 95.6, 81.0, 65.5, 62.0, 44.9, 37.9, 26.5, 20.5. **HRMS**-ESI (m/z) ring-closed product: [M + H]$^+$ calculated for $C_{17}H_{19}N_2O_3^+$, 299.1390, found, 299.1391. **$^1$H-NMR** ring-opened product (500 MHz, $CDCl_3$): δ 10.35 (s, 1H), 8.50-8.48 (m, 2H), 7.03 (d, *J* = 6.0 Hz, 2H), 5.86 (s, 1H), 5.20-5.16 (m, 2H), 3.91 (d, *J* = 10.8 Hz, 1H), 3.82-3.77 (m, 1H), 3.56-3.49 (m, 1H), 3.29-3.24 (m, 1H), 3.22-3.15 (m, 1H), 2.34-2.27 (m, 1H), 2.10 (s, 3H), 1.57-1.50 (m, 3H). **UHPCL-MS**-ESI (m/z) ring-opened product: 301.0 [M + H]$^+$.

**(±) 7-methyl-8-((3-methylpyridin-4-yl)methyl)-8,9b-dihydro-1H-pyrano[3',4':4,5]furo[3,2-c]pyridin-9(4aH)-one (9an)**

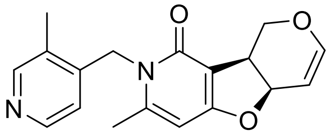

According to GP2, **7a** (95.2 mg, 0.33 mmol) was reacted with **5q** (69.1 mg, 0.30 mmol) at 100 °C for 18 hours. Purification by MPLC (cyclohexane/EtOAc 1:0 to 0:1) afforded the product (68.6 mg, 74%). **$^1$H-NMR** (600 MHz, $CD_2Cl_2$): δ 8.38 (s, 1H), 8.30 (d, *J* = 5.1 Hz, 1H), 6.78 (d, *J* = 5.8 Hz, 1H), 6.51 (d, *J* = 5.1 Hz, 1H), 5.94 (s, 1H), 5.29 (d, *J* = 17.3 Hz, 1H), 5.22 (dd, *J* = 5.8, 4.3 Hz, 1H), 5.09-5.04 (m, 2H), 4.34 (dd, *J* = 10.4, 4.7 Hz, 1H), 3.57 (t, *J* = 10.4 Hz, 1H), 3.53-3.50 (m, 1H), 2.36 (s, 3H), 2.16 (s, 3H). **$^{13}$C-NMR** (151 MHz, $CD_2Cl_2$): δ 167.8, 161.4, 150.8,

150.1, 148.3, 148.2, 144.3, 130.5, 118.8, 106.6, 99.3, 96.3, 77.4, 65.0, 43.7, 38.2, 20.9, 15.8. **HRMS**-ESI (m/z): $[M + H]^+$ calculated for $C_{18}H_{19}O_3N_2$, 311.1390; found, 311.1390.

**(±) 8-(2-(5-methoxy-1H-indol-3-yl)ethyl)-7-methyl-4,4a,8,9b-tetrahydro-1H-pyrano[3',4':4,5]furo[3,2-c]pyridin-9(3H)-one (10ao)**

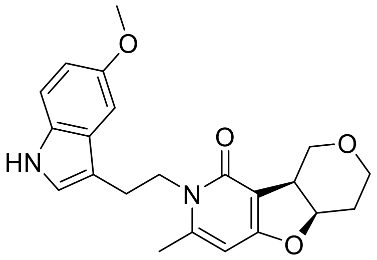


According to GP2, **7a** (63.5 mg, 0.22 mmol) was reacted with **5p** (59.7 mg, 0.20 mmol) at 110 °C for 1 hour. After filtration over celite the crude was purified by MPLC (cyclohexane/EtOAc 1:0 to 0:1 + 0.1% $NEt_3$) and the product was directly suspended with Pd/C (5 mg, 10 wt%) in toluene (2 mL) and was hydrogenated at 20 °C for 24 hours using a $H_2$-balloon. The catalyst was filtered off and the solvent removed in vacuo and the crude was purified by prep. HPLC to afford the product (17.4 mg, 23%). **$^1$H-NMR** (500 MHz, $CDCl_3$): δ 8.02 (s, 1H), 7.24 (d, *J* = 8.7, 1H), 7.08 (d, *J* = 2.4 Hz, 1H), 6.98 (d, *J* = 2.3 Hz, 1H), 6.86 (dd, *J* = 8.7, 2.4 Hz, 1H), 5.96 (s, 1H), 4.92 (dt, *J* = 7.6, 3.6 Hz, 1H), 4.35 (dt, *J* = 14.6, 7.5 Hz, 1H), 4.21 (dd, *J* = 14.6, 6.7 Hz, 1H), 4.16 (dd, *J* = 11.8, 5.9 Hz, 1H), 3.64 (td, *J* = 11.2, 3.9 Hz, 1H), 3.52 (td, *J* = 7.6, 5.9 Hz, 1H), 3.44 (dd, *J* = 11.8, 8.1 Hz, 1H), 3.13 (t, *J* = 7.5 Hz, 2H), 2.23 (s, 3H), 2.21-2.15 (m, 1H), 2.12 (dq, *J* = 15.0, 3.6 Hz, 1H). **$^{13}$C-NMR** (126 MHz, $CDCl_3$): δ 168.9, 161.9, 154.3, 149.0, 131.4, 127.8, 123.2, 112.6, 112.1, 110.3, 100.4, 97.8, 82.4, 67.1, 62.7, 56.0, 46.0, 37.9, 26.7. **HRMS**-ESI (m/z): $[M + H]^+$ calculated for $C_{22}H_{25}N_2O_4^+$, 381.1809; found, 381.1807.

**(±) 1,2,3,4,5b,8,9,9a-octahydro-5H,6H-cyclopenta[b]pyrano[3',4':4,5]furo[2,3-d]pyridin-5-one (10ap)**

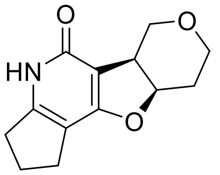


According to GP2, **7a** (67.5 mg, 0.23 mmol) was reacted with 4-hydroxy-1,5,6,7-tetrahydro-2H-cyclopenta[b]pyridin-2-one (29.5 mg, 0.20 mmol) at 110 °C for 1 hour. After filtration over celite the crude was purified by FC (cyclohexane/EtOAc 1:0 to 0:1) and the product was directly suspended with Pd/C (5 mg, 10 wt%) in toluene (2 mL) and was hydrogenated at 20 °C for 12 hours using a $H_2$-balloon. The catalyst was filtered off and the solvent removed in vacuo. The crude was purified by MPLC (EtOAc/MeOH 1:0 to 9:1) to afford the product (15.5 mg, 32%). **$^1$H-NMR** (600 MHz, $CDCl_3$): δ 5.00-4.96 (m, 1H), 4.13-4.07 (m, 1H), 3.85 (ddd, *J* = 11.3, 5.7, 3.4 Hz, 1H), 3.66 (td, *J* = 11.3, 4.0 Hz, 1H), 3.49-3.44 (m, 2H), 2.93 (t, *J* = 7.7 Hz, 2H), 2.80-2.76 (m, 2H), 2.23-2.12 (m, 4H). **$^{13}$C-NMR** (151 MHz, $CDCl_3$): δ 169.0, 161.5, 153.9, 111.3, 109.0,

83.0, 62.8, 37.1, 31.3, 26.7, 26.7, 23.2. **HRMS**-ESI (m/z): $[M + H]^+$ calculated for $C_{13}H_{16}NO_3^+$, 234.1125; found, 234.1127.

**(±) 11-oxo-4a,7,8,9,11,11b-hexahydro-1H-pyrano[3',4':4,5]furo[3,2-f]indolizine-6-carboxylic acid (9aq)**

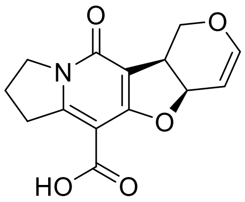


According to GP2, **7a** (63.5 mg, 0.22 mmol) was reacted with **5u** (39.0 mg, 0.20 mmol) at 110 °C for 1 hour. After filtration over celite the crude was purified by FC (cyclohexane/EtOAc 1:0 to 0:1 + 0.1% acetic acid) and repurified by prep. HPLC to afford the product (4.0 mg, 7%). **$^1$H-NMR** (700 MHz, Acetone): δ 9.59 (d, *J* = 8.0 Hz, 1H), 7.87 (dd, *J* = 15.4, 11.4 Hz, 1H), 7.41 (ddd, *J* = 15.0, 11.4, 0.8 Hz, 1H), 7.34 (d, *J* = 15.4 Hz, 1H), 6.13 (dd, *J* = 15.0, 8.0 Hz, 1H), 4.17-4.12 (m, 2H), 3.64 (t, *J* = 7.9 Hz, 2H), 2.24 (p, *J* = 7.9 Hz, 2H), 2.07-2.06 (m, 2H). **$^{13}$C-NMR** (176 MHz, Acetone): δ 193.7, 172.5, 160.9, 160.9, 156.4, 135.0, 130.3, 127.6, 105.6, 95.9, 95.9, 50.4, 36.3, 20.5. **HRMS**-ESI (m/z): $[M + H]^+$ calculated for $C_{14}H_{14}NO_5^+$, 276.0867; found, 276.0874.

**(±) 6b,9,10,10a-tetrahydro-7H-pyrano[3',4':4,5]furo[3,2-c]quinolin-6(5H)-one (10ar)**

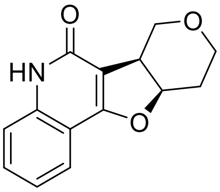


According to GP2, **7a** (75.9 mg, 0.26 mmol) was reacted with 4-hydroxyquinolin-2(1H)-one (31.4 mg, 0.20 mmol) at 100 °C for 1 hour. After filtration over celite the crude was purified by FC (cyclohexane/EtOAc 1:0 to 1:1) and the product was directly suspended with Pd/C (5 mg, 10 wt%) in toluene (2 mL) and was hydrogenated at 20 °C for 48 hours using a $H_2$-balloon. The catalyst was filtered off and the solvent removed in vacuo. The crude was purified by MPLC (EtOAc/MeOH 1:0 to 95:5) to afford the product (4.5 mg, 10%). **$^1$H-NMR** (500 MHz, $CD_2Cl_2$): δ 11.30 (s, 1H), 7.72 (dd, *J* = 8.3, 1.0 Hz, 1H), 7.53 (ddd, *J* = 8.3, 7.7, 1.4 Hz, 1H), 7.36 (d, *J* = 8.3 Hz, 1H), 7.22 (dd, *J* = 8.3, 7.7 Hz, 1H), 5.13-5.09 (m, 1H), 4.14 (dd, *J* = 11.7, 5.9 Hz, 1H), 3.86-3.80 (m, 1H), 3.69 (ddd, *J* = 11.7, 10.1, 4.5 Hz, 1H), 3.64 (dd, *J* = 11.7, 7.7 Hz, 1H), 3.55 (dd, *J* = 13.6, 7.7 Hz, 1H), 2.28-2.15 (m, 2H). **$^{13}$C-NMR** (126 MHz, $CD_2Cl_2$): δ 165.8, 163.1, 140.0, 131.4, 122.7, 122.4, 116.3, 112.3, 111.4, 82.9, 67.3, 63.2, 38.8, 27.4. **HRMS**-ESI (m/z): $[M + H]^+$ calculated for $C_{14}H_{14}NO_3^+$, 244.0968; found, 244.0970.

**(±) 5-methyl-6b,9,10,10a-tetrahydro-7H-pyrano[3',4':4,5]furo[3,2-c]quinolin-6(5H)-one (10as)**

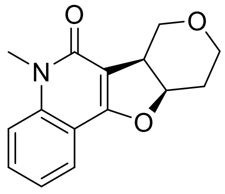

According to GP2, **7a** (75.9 mg, 0.26 mmol) was reacted with 4-hydroxy-1-methylquinolin-2(1H)-one (34.2 mg, 0.20 mmol) at 100 °C for 1 hour. After filtration over celite the crude was purified by FC (cyclohexane/EtOAc 1:0 to 1:1) and the product was directly suspended with Pd/C (5 mg, 10 wt%) in toluene (2 mL) and was hydrogenated at 20 °C for 6 hours using a $H_2$-balloon. The catalyst was filtered off and the solvent removed in vacuo. The crude was purified by MPLC (cyclohexane/EtOAc 1:0 to 0:1) to afford the product (26.1 mg, 52%). **$^{1}$H-NMR** (500 MHz, $CDCl_3$): δ 7.78 (dd, *J* = 7.5, 1.4 Hz, 1H), 7.60 (ddd, *J* = 8.7, 7.3, 1.4 Hz, 1H), 7.38 (d, *J* = 8.7 Hz, 1H), 7.25 (dd, *J* = 7.5, 7.3 Hz, 2H), 5.07-5.03 (m, 1H), 4.25-4.17 (m, 1H), 3.88 (dt, *J* = 11.4, 4.5 Hz, 1H), 3.72-3.66 (m, 4H), 3.60-3.53 (m, 2H), 2.27-2.20 (m, 2H). **$^{13}$C-NMR** (126 MHz, $CDCl_3$): δ 163.6, 161.3, 140.9, 131.4, 123.3, 121.8, 114.7, 112.9, 111.3, 82.2, 67.5, 63.0, 39.0, 29.2, 27.1. **HRMS**-ESI (m/z): $[M + H]^+$ calculated for $C_{15}H_{16}NO_3^+$, 258.1125; found, 258.1125.

**(±) 1,3,4,5,6b,9,10,10a-octahydro-7H-pyrano[3',4':4,5]furo[3,2-c]quinolin-6(2H)-one (10at); (±) 4-hydroxy-3-(tetrahydro-2H-pyran-3-yl)-5,6,7,8-tetrahydroquinolin-2(1H)-one (17ac)**

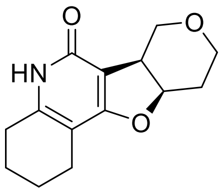

**10at**

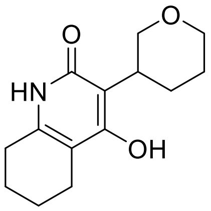

**17ac**

According to GP2, **3a** (73.3 mg, 0.25 mmol) was reacted with 4-hydroxy-5,6,7,8-tetrahydroquinolin-2(1H)-one (35.0 mg, 0.21 mmol) at 110 °C for 1 hour. After filtration over celite the crude was purified by MPLC (cyclohexane/EtOAc 1:0 to 0:1) and the product was directly suspended with Pd/C (5 mg, 10 wt%) in toluene (2 mL) and was hydrogenated at 20 °C for 12 hours using a $H_2$-balloon. The catalyst was filtered off and the solvent removed in vacuo. The crude was purified by MPLC (EtOAc/MeOH 1:0 to 9:1 + 0.1% DIPEA) to afford a mixture of products a and b. The product mixture was subjected for separation by prep. HPLC to afford pure product **10at** (2.3 mg, 4%) and **17ac** (1.1 mg, 2%). **$^{1}$H-NMR** product **10at** (700 MHz, $CD_2Cl_2$): δ 14.5 (bs, 1H), 5.07 (dt, *J* = 7.7, 4.3 Hz, 1H), 4.02 (dd, *J* = 11.3, 5.1 Hz, 1H), 3.79 (ddd, *J* = 11.5, 5.7, 3.8 Hz, 1H), 3.65 (ddd, *J* = 11.5, 10.3, 4.0 Hz, 1H), 3.56-3.49 (m, 2H), 2.75 (t, *J* = 6.3 Hz, 2H), 2.51 (t, *J* = 6.4 Hz, 2H), 2.22-2.17 (m, 1H), 2.13-2.09 (m, 1H), 1.87-1.83 (m, 2H), 1.81-1.77 (m, 2H). **$^{13}$C-NMR** product **10at** (176 MHz, $CD_2Cl_2$): δ 172.8, 164.9,

158.8, 148.0, 108.7, 83.8, 66.0, 62.5, 37.2, 26.6, 26.6, 21.2, 21.2, 20.7. **HRMS**-ESI (m/z) product **10at**: [M + H]$^+$ calculated for $C_{14}H_{18}NO_3^+$, 248.1281; found, 248.1282. **$^1$H-NMR** product **17ac** (700 MHz, $CD_2Cl_2$): δ 14.5 (bs, 1H), 12.2 (s, 1H), 4.21 (dd, *J* = 11.6, 4.5 Hz, 1H), 4.14 (d, *J* = 12.8 Hz, 1H), 3.96 (dd, *J* = 12.8, 3.4 Hz, 1H), 3.70 (ddd, *J* = 12.6, 11.6, 2.5 Hz, 1H), 3.43-3.40 (m, 1H), 2.73 (t, *J* = 6.2 Hz, 2H), 2.54 (t, *J* = 6.4 Hz, 1H), 2.00-1.90 (m, 2H), 1.84-1.77 (m, 4H), 1.70-1.57 (m, 2H). **UPHLC-MS**-ESI (m/z) product **17ac**: 250.0 [M + H]$^+$.

**(±) (5aR,9S,9aS)-3,9-dimethyl-5a,9a-dihydro-1H,9H-furo[3,2-c:4,5-c']dipyran-1-one (9ba)**

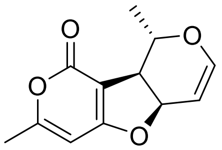

A reaction vessel was charged with 4-Hydroxy-6-methyl-2-pyron (27.1 mg, 0.21 mmol, 1.0 eq), evacuated and back-filled with argon. Toluene (1.5 mL) and Triethylamine (30.0 µL, 0.21 mmol, 1.0 equiv) were added to the reaction vessel. A separate reaction vessel was charged with $Pd(PPh_3)_4$ (5 mol%), evacuated and back-filled with argon, then charged with toluene (0.5 mL) followed by **7b**-*trans* (65.0 mg, 0.17 mmol, 1.0 equiv) The contents of this flask were stirred for approximately 20 min. before being added via syringe to the bis-nucleophile solution. The reaction was stirred for one day at room temperature before being concentrated in vacuo and purified by flash column chromatography (Heptane/EtOAc 9:1 to 4:1 to 7:3 + 1% $NEt_3$) to afford the product (69% yield). **$^1$H-NMR** (500 MHz, DMSO-$d_6$): δ 6.88 (d, *J* = 6.2 Hz, 1H), 6.31 (s, 1H), 5.29 (dd, *J* = 6.2 Hz, 4.7 Hz, 1H), 5.11 (ddd, *J* = 7.2 Hz, 4.7 Hz, 1.0 Hz, 1H), 3.39 (dd, *J* = 10.8 Hz, 6.2 Hz, 1H), 2.92 (dd, *J* = 10.8 Hz, 7.2 Hz, 1H), 2.22 (s, 3H), 1.35 (d, *J* = 6.2 Hz, 3H). **$^{13}$C-NMR** (126 MHz, DMSO-$d_6$): δ 172.2, 166.5, 161.2, 149.8, 99.7, 97.9, 95.8, 79.5, 72.5, 41.7, 20.0, 19.1. **HRMS**-ESI (m/z): [M + H]$^+$ calculated for $C_{12}H_{13}O_4^+$, 221.0808; found, 221.0807.

**(±) 3,9-dimethyl-5a,6,9,9a-tetrahydro-1H,7H-furo[3,2-c:4,5-c']dipyran-1-one (10ba); (±) 4-hydroxy-6-methyl-3-(2-methyltetrahydro-2H-pyran-3-yl)-2H-pyran-2-one (17ba)**

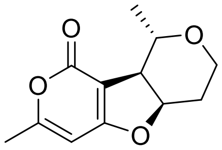

**10ba**

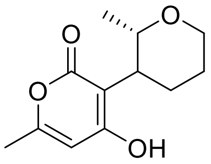

**17ba**

A suspension of **9ba** (16.3 mg, 0.07 mmol) and Pd/C (1.6 mg, 10 wt%) in toluene (0.7 mL) was hydrogenated at 20 °C for 20 hours using a $H_2$-balloon. The catalyst was filtered off and the solvent removed in vacuo. The crude was purified by prep. HPLC to afford product **10ba** (3.7 mg, 22%) and **17ba** (5.4 mg, 33%) in separated fractions. **$^1$H-NMR** product **10ba** (500 MHz, $CDCl_3$): δ 5.97 (s, 1H), 4.88-4.81 (m, 1H), 3.97-3.92 (m, 1H), 3.58 (td, *J* = 11.6, 4.0 Hz, 1H), 3.17 (dq, *J* = 9.9, 6.1 Hz, 1H), 2.83 (dd, *J* = 9.8, 6.5 Hz, 1H), 2.27 (s, 3H), 2.12-2.07 (m, 2H), 1.41 (d, *J* = 6.2 Hz, 3H). **$^{13}$C-NMR**

product **10ba** (126 MHz, $CDCl_3$): δ 166.4, 162.3, 147.0, 103.6, 96.2, 84.8, 63.1, 43.1, 23.8, 21.4, 20.7. **HRMS**-ESI (m/z) product **10ba**: $[M + H]^+$ calculated for $C_{12}H_{15}O_4^+$, 223.0965; found, 223.0964. **$^1$H-NMR** product **17ba** (500 MHz, $CDCl_3$): δ 11.86 (s, 1H), 5.86 (s, 1H), 4.37-4.26 (m, 1H), 3.93 (td, *J* = 11.3, 10.8, 3.1 Hz, 1H), 3.88-3.76 (m, 1H), 2.19 (s, 3H), 2.12-2.00 (m, 1H), 1.78-1.61 (m, 2H), 1.57-1.43 (m, 2H). **HRMS**-ESI (m/z) product **17ba**: $[M + H]^+$ calculated for $C_{12}H_{17}O_4^+$, 225.1121; found, 225.1120.

**(±) 5,7-dimethyl-6b,9,10,10a-tetrahydro-7H-pyrano[3',4':4,5]furo[3,2-c]quinolin-6(5H)-one (10bb); (±) 4-hydroxy-1-methyl-3-(2-methyltetrahydro-2H-pyran-3-yl)quinolin-2(1H)-one (17bb)**

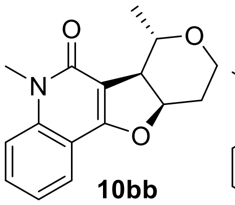
**10bb**

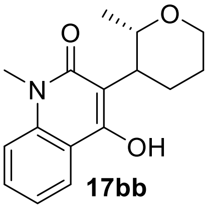
**17bb**

According to GP3, **7b**-*trans* (66.5 mg, 0.22 mmol) was reacted with 4-hydroxy-1-methylquinolin-2(1H)-one (35.0 mg, 0.20 mmol). After purification by MPLC (cyclohexane/EtOAc 1:0 to 0:1), the product was directly suspended with Pd/C (5 mg, 10 wt%) in toluene (2 mL) and was hydrogenated at 20 °C for 48 hours using a $H_2$-balloon. The catalyst was filtered off and the solvent removed in vacuo. The crude was purified by prep. HPLC to afford product **10bb** (3.6 mg, 7%) and **17bb** (3.6 mg, 7%) in separated fractions. **$^1$H-NMR** product **10bb** (500 MHz, $CD_2Cl_2$): δ 7.80 (dd, *J* = 8.0, 1.6 Hz, 1H), 7.63 (ddd, *J* = 8.7, 7.6, 1.6 Hz, 1H), 7.43 (d, *J* = 8.7 Hz, 1H), 7.27 (dd, *J* = 8.0, 7.6 Hz, 1H), 4.93 (m, 1H), 3.94 (ddt, *J* = 12.0, 5.9, 1.3 Hz, 1H), 3.69 (s, 3H), 3.64 (td, *J* = 12.0, 2.7 Hz, 1H), 3.21 (dq, *J* = 9.9, 6.1 Hz, 1H), 3.00 (dd, *J* = 9.9, 6.4 Hz, 1H), 2.27 (dq, *J* = 15.4, 2.2 Hz, 1H), 2.16 (dddd, *J* = 15.4, 12.8, 5.9, 4.3 Hz, 1H), 1.39 (d, *J* = 6.1 Hz, 3H). **$^{13}$C-NMR** product **10bb** (126 MHz, $CD_2Cl_2$): δ 164.6, 161.5, 141.0, 131.8, 123.4, 122.1, 115.1, 113.1, 112.6, 84.7, 77.3, 63.3, 45.3, 29.6, 27.4, 21.6. **HRMS**-ESI (m/z) product **10bb**: $[M + H]^+$ calculated for $C_{16}H_{18}NO_3^+$, 272.1261; found, 272.1278. **$^1$H-NMR** product **17bb** (500 MHz, MeOH-$d_4$): δ 8.07-7.06 (m, 1H), 7.65-7.58 (m, 1H), 7.57-7.48 (m, 1H), 7.34-7.26 (m, 1H), 4.54-4.38 (m, 1H), 4.02-3.95 (m, 1H), 3.72-3.62 (m, 4H), 3.07-2.93 (m 1H), 2.53-2.39 (m, 1H), 1.86-1.58 (m, 3H), 1.01 (bs, 3H). **$^{13}$C-NMR** product **17bb** (126 MHz, MeOH-$d_4$): δ 159.5, 159.4, 140.0, 131.9, 124.7, 123.1, 118.0, 115.4, 113.5, 76.4, 69.4, 43.1, 29.7, 27.8, 27.7, 20.2. **HRMS**-ESI (m/z) product **17bb**: $[M + H]^+$ calculated for $C_{16}H_{20}NO_3^+$, 274.1438; found, 274.1445.

**(±) 1-methyl-4,4a,6,7,8,9b-hexahydro-1H-pyrano[3',4':4,5]furo[3,2-c]pyridin-9(3H)-one (10bc)**

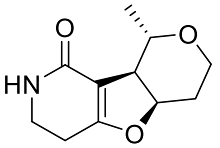

According to GP3, **7b**-*trans* (66.5 mg, 0.22 mmol) was reacted with 4-hydroxy-5,6-dihydropyridin-2(1H)-one (22.6 mg, 0.20 mmol). After purification by MPLC (cyclohexane/EtOAc 1:0 to 0:1), the product was directly suspended with Pd/C (5 mg, 10 wt%) in toluene (2 mL) and was hydrogenated at 20 °C for 48 hours using a $H_2$-balloon. The catalyst was filtered off and the solvent removed in vacuo. The crude was purified by prep. HPLC to afford the product (8.8 mg, 21%). **$^1$H-NMR** (600 MHz, $CD_2Cl_2$): δ 5.72 (s, 1H), 4.74-4.71 (m, 1H), 3.90-3.85 (m, 1H), 3.55 (td, *J* = 11.5, 4.2 Hz, 1H), 3.52-3.42 (m, 2H), 3.13 (dt, *J* = 12.3, 6.1 Hz, 1H), 2.62-2.59 (m, 1H), 2.58-2.54 (m, 1H), 2.49 (dt, *J* = 17.3, 6.4 Hz, 1H), 2.08-2.03 (m, 1H), 1.28 (d, *J* = 6.1 Hz, 3H). **$^{13}$C-NMR** (151 MHz, $CD_2Cl_2$): δ 171.9, 168.3, 110.0, 84.7, 78.0, 63.2, 43.9, 39.7, 27.4, 23.8, 21.1. **HRMS**-ESI (m/z): $[M + H]^+$ calculated for $C_{11}H_{16}NO_3^+$, 210.1125; found, 210.1122.

**(±) 1,7,8-trimethyl-8,9b-dihydro-1H-pyrano[3',4':4,5]furo[3,2-c]pyridin-9(4aH)-one (9bd)**

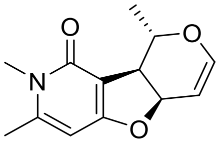

According to GP3, **7b**-*trans* (100.0 mg, 0.33 mmol) was reacted with **5b** (46.0 mg, 0.33 mmol). Purification by FC (Hep/EtOAc 7:3 to 1:1 + 1% $NEt_3$) afforded the product (37 mg, 48%). **$^1$H-NMR** (700 MHz DMSO-$d_6$): δ 6.82 (d, *J* = 6.1 Hz, 1H) 6.01 (s, 1H), 5.24 (dd, *J* = 6.1 Hz, 5.0 Hz, 1H), 4.92 (dd, *J* = 6.7 Hz, 5.0 Hz, 1H), 3.37 (s, 3H), 3.34 (dd, *J* = 10.7 Hz, 6.2 Hz, 1H), 2.88 (dd, *J* = 10.7 Hz, 6.7 Hz, 1H), 2.32 (s, 3H), 1.37 (d, *J* = 6.2 Hz, 3H). **$^{13}$C-NMR** (176 MHz, DMSO-$d_6$): δ 167.3, 161.3, 150.7, 149.5, 106.3, 99.0, 94.7, 78.2, 73.4, 43.5, 30.6, 21.5, 19.9. **HRMS**-ESI (m/z): $[M + H]^+$ calculated for $C_{13}H_{16}O_3N^+$, 234.1125; found, 234.1124.

**(±) 1,7,8-trimethyl-4,4a,8,9b-tetrahydro-1H-pyrano[3',4':4,5]furo[3,2-c]pyridin-9(3H)-one (10bd); (±) 4-hydroxy-1,6-dimethyl-3-(2-methyltetrahydro-2H-pyran-3-yl)pyridin-2(1H)-one (17bd)**

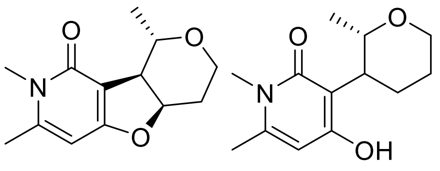

**10bd** **17bd**

According to GP3, **7b**-*trans* (69.6 mg, 0.23 mmol) was reacted with **5b** (27.8 mg, 0.20 mmol). After purification by MPLC (cyclohexane/EtOAc 1:0 to 0:1), the product was directly suspended with Pd/C (5 mg, 10 wt%) in toluene (2 mL) and was hydrogenated at 20 °C for 48 hours using a $H_2$-balloon. The catalyst

was filtered off and the solvent removed in vacuo. The crude was purified by prep. HPLC to afford product **10bd** (3.8 mg, 8%) and **17bd** (16.0 mg, 34%) in separated fractions. **$^{1}$H-NMR** product **10bd** (500 MHz, $CDCl_3$): δ 6.01 (s, 1H), 4.75 (dt, *J* = 6.2, 3.3 Hz, 1H), 3.96-3.91 (m, 1H), 3.61 (td, *J* = 11.2, 4.9 Hz, 1H), 3.54 (s, 3H), 3.15 (dq, *J* = 9.9, 6.2 Hz, 1H), 2.95 (dd, *J* = 9.9, 6.2 Hz, 1H), 2.38 (s, 3H), 2.12 (ddd, *J* = 11.2, 5.4, 3.3 Hz, 2H), 1.41 (d, *J* = 6.2 Hz, 3H). **$^{13}$C-NMR** product **10bd** (126 MHz, $CDCl_3$): δ 168.8, 162.1, 149.0, 110.9, 97.2, 83.9, 77.0, 63.1, 44.1, 31.6, 27.0, 22.0, 21.5. **HRMS**-ESI (m/z) product **10bd**: $[M + H]^+$ calculated for $C_{13}H_{18}NO_3^+$, 236.1281; found, 236.1286. **$^{1}$H-NMR** product **17bd** (500 MHz, $CD_2Cl_2$): δ 5.90 (s, 1H), 4.34-4.21 (m, 1H), 3.97-3.89 (m, 1H), 3.75-3.68 (m, 1H), 3.19-3.11 (m, 1H), 2.32 (s, 3H), 1.97-1.88 (m, 1H), 1.85-1.76 (m, 1H), 1.67-1.57 (m, 2H), 1.33-1.29 (bs, 3H). **$^{13}$C-NMR** product **17bd** (126 MHz, $CD_2Cl_2$): δ 165.4, 164.2, 144.9, 111.0, 103.6, 73.3, 63.8, 38.5, 32.2, 24.6, 24.4, 20.9, 18.0. **HRMS**-ESI (m/z) product **17bd**: $[M + H]^+$ calculated for $C_{13}H_{20}NO_3^+$, 238.1438; found, 238.1443.

**(±) 8-benzyl-1,7-dimethyl-8,9b-dihydro-1H-pyrano[3',4':4,5]furo[3,2-c]pyridin-9(4aH)-one (9be)**

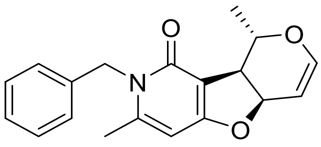

According to GP3, **7b**-*trans* (100.0 mg, 0.33 mmol) was reacted with **5c** (71.2 mg, 0.33 mmol). Purification by FC (Hep/EtOAc 7:3 to 1:1 + 1% $NEt_3$) afforded the product (36 mg, 35%). **$^{1}$H-NMR** (700 MHz DMSO-$d_6$): δ 7.33 (dd, *J* = 7.5 Hz, 7.4 Hz, 2H), 7.25 (dd, *J* = 7.4 Hz, 7.4 Hz, 1H), 7.10 (d, *J* = 7.5 Hz, 2H), 6.85 (d, *J* = 6.2 Hz, 1H) 6.07 (s, 1H), 5.37 (d, *J* = 15.2 Hz, 1H), 5.27 (dd, *J* = 6.2 Hz, 4.8 Hz, 1H), 5.16 (d, *J* = 15.2 Hz, 1H), 5.00 (m, 1H), 3.44-3.39 (m, 1H), 2.94 (dd, *J* = 10.7 Hz, 7.0 Hz, 1H), 2.23 (s, 3H), 1.39 (d, *J* = 6.3 Hz, 3H). **$^{13}$C-NMR** (176 MHz, DMSO-$d_6$): δ 167.7, 161.5, 150.5, 149.7, 137.9, 129.1, 127.5, 126.6, 106.8, 99.0, 95.6, 78.5, 73.5, 46.2, 43.4, 21.0, 19.9. **LCMS**-ESI (m/z): 310.28 $[M + H]^+$.

**(±) 8-benzyl-1,7-dimethyl-4,4a,8,9b-tetrahydro-1H-pyrano[3',4':4,5]furo[3,2-c]pyridin-9(3H)-one (10be); (±) 1-benzyl-4-hydroxy-6-methyl-3-(2-methyltetrahydro-2H-pyran-3-yl)pyridin-2(1H)-one (17bf)**

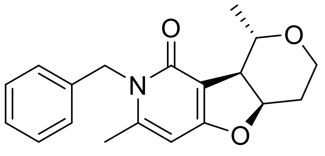

**10be**

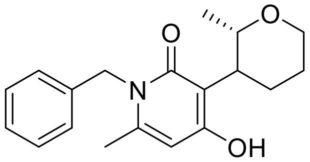

**17bf**

A suspension of **9be** (13.9 mg, 0.04 mmol) and Pd/C (1.4 mg, 10 wt%) in toluene (0.5 mL) was hydrogenated at 20 °C for 24 hours using a $H_2$-balloon. The catalyst was filtered off and the solvent removed in vacuo. The crude was purified by prep. HPLC to afford

product **10be** (5.0 mg, 36%) and **17bf** (5 mg, 36%) in separated fractions. **$^{1}$H-NMR** product **10be** (600 MHz, $CDCl_3$): δ 7.34-7.21 (m, 3H), 7.13 (d, *J* = 7.0 Hz, 2H), 5.92 (s, 1H), 5.46 (d, *J* = 16.0 Hz, 1H), 5.19 (d, *J* = 16.0 Hz, 1H), 4.80-4.76 (m, 1H), 3.97-3.92 (m, 1H), 3.63 (td, *J* = 11.2, 4.9 Hz, 1H), 3.26-3.19 (m, 1H), 2.97 (dd, *J* = 9.8, 6.3 Hz, 1H), 2.28 (s, 3H), 2.16-2.09 (m, 2H), 1.46 (d, *J* = 6.2 Hz, 3H). **HRMS**-ESI (m/z) product **10be**: $[M + H]^+$ calculated for $C_{19}H_{22}NO_3^+$, 312.1594; found, 312.1599. **$^{1}$H-NMR** product **17bf** (600 MHz, $CDCl_3$): δ 11.03 (s, 1H), 7.29 (t, *J* = 7.5 Hz, 2H), 7.23 (t, *J* = 7.5 Hz, 1H), 7.11 (d, *J* = 7.5 Hz, 2H), 5.86 (s, 1H), 5.38 (d, *J* = 16.1 Hz, 1H), 5.21 (d, *J* = 16.1 Hz, 1H), 4.42-4.34 (m, 1H), 3.93 (t, *J* = 11.7 Hz, 1H), 3.86-3.80 (m, 1H), 3.36-3.30 (m, 1H), 2.22 (s, 3H), 2.13-2.06 (m, 1H), 1.79-1.67 (m, 2H), 1.58-1.50 (m, 1H), 1.47 (d, *J* = 6.5 Hz, 3H). **$^{13}$C-NMR** product **17bf** (151 MHz, $CDCl_3$): δ 165.2, 163.4, 144.0, 137.2, 129.9, 127.3, 126.4, 110.8, 103.4, 72.4, 61.9, 47.7, 36.9, 23.5, 23.2, 20.4, 17.2. **HRMS**-ESI (m/z) product **17bf**: $[M + H]^+$ calculated for $C_{19}H_{24}NO_3^+$, 314.1751; found, 314.1751.

**(±) 8-(4-methoxybenzyl)-1,7-dimethyl-8,9b-dihydro-1H-pyrano[3',4':4,5]furo[3,2-c]pyridin-9(4aH)-one (9bf); (±) 6-(4-methoxybenzyl)-2,7-dimethyl-4a,9a-dihydro-2H-pyrano[3',2':4,5]furo[3,2-c]pyridin-5(6H)-one (15f)**

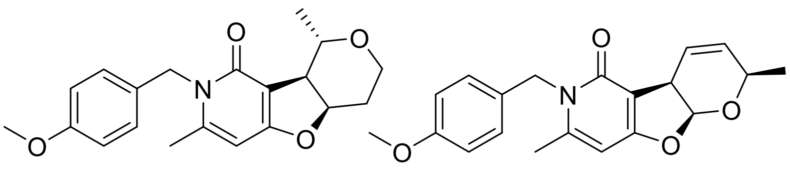

**9bf** **15f**

According to GP3, **7b**-*trans* (50.0 mg, 0.17 mmol) was reacted with **5d** (35.0 mg, 0.15 mmol). Purification by FC (Hep/EtOAc 7:3 to 1:1 + 1% $NEt_3$) afforded the product **9bf** (7.5 mg, 13%) and product **15f** (8.1 mg, 14%) in separated fractions. **$^{1}$H-NMR** product **9bf** (500 MHz, $CD_2Cl_2$): δ 7.08 (d, *J* = 8.6 Hz, 2H), 6.84 (d, *J* = 8.6 Hz, 2H), 6.78 (d, *J* = 6.2 Hz, 1H), 5.84 (s, 1H), 5.28 (d, *J* = 15.7 Hz, 1H), 5.24 (dd, *J* = 6.2, 4.7 Hz, 1H), 5.13 (d, *J* = 15.7 Hz, 1H), 4.95 (ddd, *J* = 6.9, 4.7, 1.1 Hz, 1H), 3.76 (s, 3H), 3.51 (dq, *J* = 11.5, 6.3 Hz, 1H), 3.01 (dd, *J* = 11.5, 6.9 Hz, 1H), 2.25 (s, 3H), 1.48 (d, *J* = 6.3 Hz, 3H). **$^{13}$C-NMR** product **9bf** (126 MHz, $CD_2Cl_2$): δ 168.1, 162.1, 159.2, 149.9, 149.7, 129.6, 128.1, 114.4, 107.6, 98.6, 95.4, 79.2, 74.0, 55.6, 46.4, 44.0, 21.5, 19.8. **UHPCL-MS**-ESI product **9bf** (m/z): 340.0 $[M + H]^+$. **$^{1}$H-NMR** product **15f** (700 MHz, $CD_2Cl_2$): δ 7.07 (d, *J* = 8.7 Hz, 2H), 6.84 (d, *J* = 8.7 Hz, 2H), 6.26 (ddd, *J* = 10.3, 3.9, 2.0 Hz, 1H), 6.13 (d, *J* = 6.6 Hz, 1H), 5.92 (s, 1H), 5.88 (ddd, *J* = 10.3, 2.6, 1.9 Hz, 1H), 5.33 (m, 1H), 5.08 (d, *J* = 15.9 Hz, 1H), 4.36 (qq, *J* = 6.9, 2.6 Hz, 1H), 3.77-3.75 (m, 4H), 2.27 (s, 3H), 1.28 (d, *J* = 6.9 Hz, 1H). **$^{13}$C-NMR** product **15f** (176 MHz, $CD_2Cl_2$): δ 165.5, 161.8, 159.3, 149.6,

130.5, 129.3, 128.1, 122.1, 114.4, 109.0, 105.8, 96.4, 68.2, 55.6, 46.5, 38.4, 22.2, 21.5. **HRMS**-ESI (m/z) product **15f**: $[M + H]^+$ calculated for $C_{20}H_{22}NO_4^+$, 340.1543; found, 340.1545.

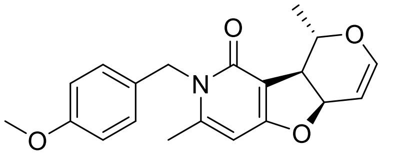

A reaction vessel was evacuated and back-filled with argon. Allyl-Pd-Cl dimer (1.37 mg, 2.5 mol%) and Xantphos (6.51 mg, 7.5 mol%) was loaded into the vessel and dissolved in THF (0.5 mL). After 5 min, 7**b**-*trans* (50.0 mg, 0.17 mmol) dissolved in THF (0.65 mL) was added. A mixture of **5d** (35.0 mg, 0.15 mmol, 1 equiv) and $NEt_3$ (41 microL, 2 equiv) in DMF (0.35 mL) was added and the mixture was stirred at room temperature overnight. The mixture was diluted with EtOAc and concentrated in vacuo. The crude was dissolved in DCM and immobilized on isolute for purification by MPLC (cyclohexane/EtOAc 1:0 to 3:7 + 0.1% $NEt_3$) to afford the product (30.9 mg, 61%).

**(±) 8-(4-methoxybenzyl)-1,7-dimethyl-4,4a,8,9b-tetrahydro-1H-pyrano[3',4':4,5]furo[3,2-c]pyridin-9(3H)-one (10bf); (±) 4-hydroxy-1-(4-methoxybenzyl)-6-methyl-3-(2-methyltetrahydro-2H-pyran-3-yl)pyridin-2(1H)-one (17bg)**

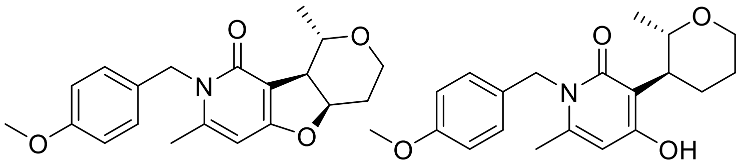

**10bf** **17bg**

**9bf** (22.5 mg, 0.07 mmol) was suspended with Pd/C (2.5 mg, 10 wt%) in toluene (1 mL) and hydrogenated at 20 °C for 48 hours using a $H_2$-balloon. The catalyst was filtered off and the solvent removed in vacuo. The crude was purified by prep. HPLC to afford the product **10bf** (7.6 mg, 34%) and **17bg** (9.0 mg, 40%) in separated fractions. **$^1$H-NMR** product **10bf** (500 MHz, $CD_2Cl_2$): δ 7.07 (d, *J* = 8.5 Hz, 2H), 6.85 (d, *J* = 8.5 Hz, 2H), 6.02 (s, 1H), 5.34 (d, , *J* = 15.6 Hz, 1H), 5.18 (d, *J* = 15.6 Hz, 1H), 4.79 (dt, *J* = 6.0, 2.9 Hz, 1H), 3.92-3.88 (m, 1H), 3.59 (td, *J* = 11.4, 4.4 Hz, 1H), 3.19-3.13 (m, 1H), 2.93 (dd, *J* = 9.8, 6.0 Hz, 1H), 2.31 (s, 3H), 2.15-2.08 (m, 2H), 1.36 (d, *J* = 6.2 Hz, 1H). **$^{13}$C-NMR** product **10bf** (126 MHz, $CD_2Cl_2$): δ 169.5, 162.3, 159.4, 150.0, 128.9, 128.1, 114.5, 111.1, 97.8, 84.4, 77.2, 63.3, 55.6, 47.1, 44.5, 27.2, 21.6, 21.5. **HRMS**-ESI (m/z) product **10bf**: $[M + H]^+$ calculated for $C_{20}H_{24}NO_4^+$, 342.1700; found, 342.1698. **$^1$H-NMR** product **17bg** (400 MHz, MeOH-$d_4$): δ 7.01 (d, *J* = 8.6 Hz, 2H), 6.86 (d, *J* = 8.6 Hz, 2H), 5.86 (s, 1H), 5.31-5.21 (m, 2H), 4.36-4.18 (m, 1H), 3.93 (dd, *J* = 11.3, 4.3 Hz, 1H), 3.76 (bs, 4H), 3.56 (t, *J* = 11.3 Hz, 1H), 2.16 (s, 3H), 1.79-1.52 (m, 4H), 1.02 (d, *J* = 6.2 Hz, 3H). **$^{13}$C-NMR** product **17bg** (126 MHz, MeOH-$d_4$): δ 164.8, 160.4, 160.3, 146.7, 130.3, 128.4, 115.2, 111.4, 101.0, 76.5 69.4,

55.7, 47.3, 40.1, 28.2, 28.1, 20.4. **HRMS**-ESI (m/z) product **17bg**: $[M + H]^+$ calculated for $C_{20}H_{26}NO_4^+$, 344.1856; found, 344.1865.

**(±) 8-(4-fluorobenzyl)-1,7-dimethyl-4,4a,8,9b-tetrahydro-1H-pyrano[3',4':4,5]furo[3,2-c]pyridin-9(3H)-one (10bg); (±) 1-(4-fluorobenzyl)-4-hydroxy-6-methyl-3-(2-methyltetrahydro-2H-pyran-3-yl)pyridin-2(1H)-one (17bh); (±) 6-(4-fluorobenzyl)-2,7-dimethyl-3,4,4a,9a-tetrahydro-2H-pyrano[3',2':4,5]furo[3,2-c]pyridin-5(6H)-one (16g);**

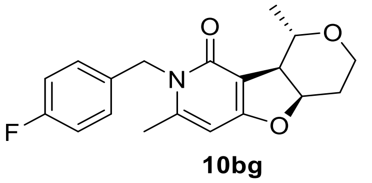


**10bg**

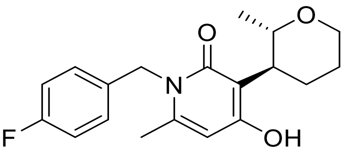


**17bh**

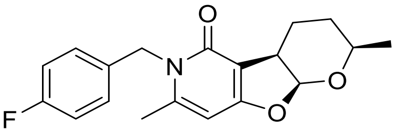


**16g**

According to GP3, **7b**-*trans* (69.6 mg, 0.23 mmol) was reacted with **5k** (46.6 mg, 0.20 mmol). After purification by MPLC (cyclohexane/EtOAc 1:0 to 0:1), the product was directly suspended with Pd/C (5 mg, 10 wt%) in toluene (2 mL) and was hydrogenated at 20 °C for 48 hours using a $H_2$-balloon. The catalyst was filtered off and the solvent removed in vacuo. The crude was purified by prep. HPLC to afford product **10bg** (4.5 mg, 7%), **17bh** (4.9 mg, 7%) and **16g** (3.0 mg, 5%) in separated fractions. **$^1$H-NMR** product **10bg** (500 MHz, $CD_2Cl_2$): δ 7.13 (dd, *J* = 8.6, 5.4 Hz, 2H), 7.01 (t, *J* = 8.6 Hz, 2H), 5.91 (s, 1H), 5.36 (d, *J* = 14.4 Hz, 1H) 5.15 (d, *J* = 14.4 Hz, 1H), 4.76 (dt, *J* = 6.3, 3.0 Hz, 1H), 3.91-3.87 (m, 1H), 3.58 (td, *J* = 11.4, 4.2 Hz, 1H), 3.16 (dq, *J* = 9.8, 6.2 Hz, 1H), 2.87 (dd, *J* = 9.8, 6.3 Hz, 1H), 2.25 (s, 1H), 2.12-2.07 (m, 2H), 1.37 (d, *J* = 6.2 Hz, 3H). **$^{13}$C-NMR** product **10bg** (126 MHz, $CD_2Cl_2$): δ 168.4, 162.9, 161.0, 161.6, 148.9, 133.1, 128.1, 115.5, 110.3, 96.3, 83.6, 77.0, 62.9, 46.1, 44.3, 27.0, 21.3, 21.2. **HRMS**-ESI (m/z) product **10bg**: $[M + H]^+$ calculated for $C_{19}H_{21}FNO_3^+$, 330.1500; found, 330.1505. **$^1$H-NMR** product **17bh** (600 MHz, $CD_2Cl_2$): δ 7.14-6.96 (m, 4H), 5.91 (s, 1H), 5.31-5.16 (m, 2H), 4.34-4.25 (m, 1H), 3.96-3.89 (m, 1H), 3.79-3.69 (m, 1H), 3.24-3.14 (m, 1H), 2.19 (s, 3H), 2.04-1.94 (m, 1H), 1.80-1.52 (m, 3H), 1.37 (bs, 3H). **$^{13}$C-NMR** product **17bh** (151 MHz, $CD_2Cl_2$): δ 165.4, 164.1, 163.2, 161.6, 144.5, 133.3, 128.4, 115.9, 111.1, 103.8, 73.1, 63.3, 47.4, 38.1, 24.4, 24.1, 20.4, 17.8. **HRMS**-ESI (m/z) product **17bh**: $[M + H]^+$ calculated for $C_{19}H_{23}FNO_3^+$, 332.1657; found, 332.1660. **$^1$H-NMR** product **16g** (500 MHz, $CD_2Cl_2$): δ 7.12 (dd, *J* = 8.6, 5.5 Hz, 2H), 7.01 (t, *J* = 8.6 Hz, 2H), 5.70 (s, 1H), 5.41 (d, *J* = 5.0 Hz, 1H), 5.27 (d, *J* = 15.8 Hz, 1H), 5.18 (d, *J* = 15.8 Hz, 1H), 4.46 (q, *J* = 6.6 Hz, 1H), 3.22-3.19 (s, 1H), 2.22-1.92 (m, 7H), 1.35 (d, *J* = 6.6 Hz, 3H). **$^{13}$C-NMR** product **16g** (126 MHz, $CD_2Cl_2$): δ 164.0, 163.4, 163.3, 144.9, 133.3, 128.4, 115.9, 114.3, 102.5, 98.1, 77.4, 47.1, 34.3,

28.2, 21.3, 20.6, 20.5. **HRMS**-ESI (m/z) product **16g**: [M + H]$^+$ calculated for $C_{19}H_{21}FNO_3^+$, 330.1500; found, 330.1506.

**(±) 4-hydroxy-3-(2-methyltetrahydro-2H-pyran-3-yl)-5,6,7,8-tetrahydroquinolin-2(1H)-one (17bc)**

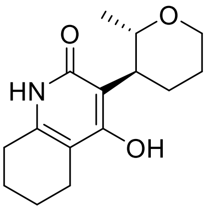


According to GP3, **7b**-*trans* (66.5 mg, 0.22 mmol) was reacted with 4-hydroxy-5,6,7,8-tetrahydroquinolin-2(1H)-one (33.0 mg, 0.20 mmol). After purification by MPLC (cyclohexane/EtOAc 1:0 to 0:1), the product was directly suspended with Pd/C (5 mg, 10 wt%) in toluene (2 mL) and was hydrogenated at 20 °C for 48 hours using a $H_2$-balloon. The catalyst was filtered off and the solvent removed in vacuo. The crude was purified by prep. HPLC to afford the product (3.1 mg, 6%). **$^1$H-NMR** (600 MHz, $CD_2Cl_2$): δ 14.65 (s, 1H), 12.42 (s, 1H), 4.38 (q, *J* = 7.0 Hz, 1H), 3.97 (td, *J* = 12.2, 2.6 Hz, 1H), 3.89 (dd, *J* = 12.2, 4.5 Hz, 1H), 3.27-3.21 (m, 1H), 2.74 (t, *J* = 6.1 Hz, 2H), 2.53 (t, *J* = 5.9 Hz, 3H), 2.21-2.14 (m, 1H), 1.86-1.77 (m, 4H), 1.72 (bd, *J* = 14.3 Hz, 1H), 1.68-1.61 (m, 1H), 1.58-1.53 (m, 1H), 1.52 (d, *J* = 7.0 Hz, 3H). **$^{13}$C-NMR** (151 MHz, $CD_2Cl_2$): δ 167.9, 159.9, 143.5, 116.0, 109.2, 71.4, 61.4, 35.1, 26.3, 23.1, 22.3, 21.6, 21.4, 21.2, 16.5. **HRMS**-ESI (m/z): [M + H]$^+$ calculated for $C_{15}H_{22}NO_3^+$, 264.1594; found, 264.1597.

**(±) 1-cyclobutyl-4-hydroxy-6-methyl-3-(2-methyltetrahydro-2H-pyran-3-yl)pyridin-2(1H)-one (17be)**

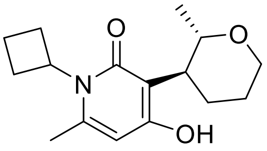


According to GP3, **7b**-*trans* (66.5 mg, 0.22 mmol) was reacted with **5e** (35.8 mg, 0.20 mmol). After purification by MPLC (cyclohexane/EtOAc 1:0 to 0:1), the product was directly suspended with Pd/C (5 mg, 10 wt%) in toluene (2 mL) and was hydrogenated at 20 °C for 48 hours using a $H_2$-balloon. The catalyst was filtered off and the solvent removed in vacuo. The crude was purified by prep. HPLC to afford the product (6.9 mg, 12%). **$^1$H-NMR** (500 MHz, MeOH-$d_4$): δ 5.68 (s, 1H), 4.81 (q, *J* = 8.7 Hz, 1H), 4.38-4.14 (m, 1H), 3.94-3.90 (m, 1H), 3.61-3.48 (m, 1H), 3.18-3.08 (m, 2H), 2.32-2.22 (m, 6H) 1.93 (qt, *J* = 10.4, 2.9 Hz, 1H), 1.82-1.74 (m, 1H), 1.72-1.59 (m, 2H), 0.98 (d, *J* = 6.2 Hz, 3H). **$^{13}$C-NMR** (126 MHz, MeOH-$d_4$): δ **HRMS**-ESI (m/z): [M + H]$^+$ calculated for $C_{16}H_{24}NO_3^+$, 278.1751; found, 278.1755.

**(±) 1,7-dimethyl-8-(4-morpholinophenyl)-8,9b-dihydro-1H-pyrano[3',4':4,5]furo[3,2-c]pyridin-9(4aH)-one (9bk)**

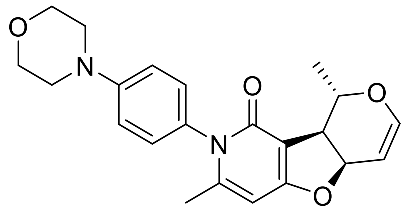

According to GP2, **7b**-*trans* (66.5 mg, 0.22 mmol) was reacted with **5f** (57.3 mg, 0.20 mmol) at 110 °C for 2 hours. The catalyst was filtered off and the filtrate was diluted with DCM (10 mL) and washed with saturated $NaHCO_3$ solution (15 mL). The aqueous phase was extracted with DCM (3 x 10 mL) and the combined organic layers were washed with brine (50 mL), dried over $MgSO_4$ and concentrated in vacuo. The crude was purified by MPLC (EtOAc/MeOH 1:0 to 9:1) to afford the product (3.3 mg, 4%). $^{1}$**H-NMR** (600 MHz, $CD_2Cl_2$): δ 7.06-6.97 (m 4H), 6.78 (d, *J* = 6.1 Hz, 1H), 5.92 (s, 1H), 5.25 (dd, *J* = 6.1, 4.9 Hz, 1H), 4.96 (dd, *J* = 6.7, 4.9 Hz, 1H), 3.86-3.83 (m, 5H), 3.55-3.50 (m, 1H), 3.21-3.19 (m, 4H), 2.99 (dd, *J* = 10.7, 6.7 Hz, 1H), 1.95 (s, 3H), 1.44 (d, *J* = 6.2 Hz, 3H). $^{13}$**C-NMR** (151 MHz, $CD_2Cl_2$): δ 168.6, 162.6, 151.6, 150.2, 149.9, 131.0, 129.3, 129.3, 116.1, 116.1, 107.6, 98.6, 95.3, 79.2, 74.0, 67.2, 54.2, 54.0, 53.8, 53.7, 53.5, 49.3, 44.0, 22.5, 19.8. **HRMS**-ESI (m/z): $[M + H]^+$ calculated for $C_{22}H_{25}N_2O_4^+$, 381.1809; found, 381.1810.

**(±) 1,7-dimethyl-8-(tetrahydro-2H-pyran-4-yl)-8,9b-dihydro-1H-pyrano[3',4':4,5]furo[3,2-c]pyridin-9(4aH)-one (9bl)**

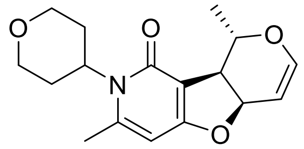

According to GP2, **7b**-*trans* (39.7 mg, 0.13 mmol) was reacted with **5g** (25.0 mg, 0.12 mmol) at 110 °C for 1 hour. The catalyst was filtered off and the filtrate was diluted with DCM (10 mL) and washed with saturated $NaHCO_3$ solution (15 mL). The aqueous phase was extracted with DCM (3 x 10 mL) and the combined organic layers were washed with brine (50 mL), dried over $MgSO_4$ and concentrated in vacuo. The crude was purified by MPLC (cyclohexane/EtOAc 1:0 to 1:1) to afford the product (9.6 mg, 27%). $^{1}$**H-NMR** (600 MHz, $CD_2Cl_2$): δ 6.76 (d, *J* = 6.2 Hz, 1H), 5.80 (s, 1H), 5.21 (dd, *J* = 6.2, 4.7 Hz, 1H), 4.89 (ddd, *J* = 6.9, 4.7, 1.1 Hz, 1H), 4.20-4.09 (bm, 1H), 4.04 (ddd, *J* = 11.7, 5.1, 1.5 Hz, 2H), 3.45 (dq, *J* = 10.7, 6.3 Hz, 1H), 3.42-3.36 (bm, 2H), 3.24-3.08 (bm, 2H), 2.94 (dd, *J* = 10.7, 6.9 Hz, 1H), 2.36 (s, 3H), 1.55-1.48 (bm, 2H), 1.45 (d, *J* = 6.3 Hz, 3H). $^{13}$**C-NMR** (151 MHz, $CD_2Cl_2$): δ 167.5, 162.7, 149.9, 148.8, 109.4, 98.6, 96.5, 79.2, 74.0, 68.3, 57.6, 44.0, 28.9, 22.7. 19.8. **HRMS**-ESI (m/z): $[M + H]^+$ calculated for $C_{17}H_{22}NO_4^+$, 304.1543; found, 304.1544.

**(±) 1,7-dimethyl-8-(pyridin-4-ylmethyl)-8,9b-dihydro-1H-pyrano[3',4':4,5]furo[3,2-c]pyridin-9(4aH)-one (9bm)**

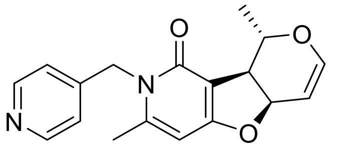

According to GP2, **7b**-*trans* (66.5 mg, 0.22 mmol) was reacted with **5i** (43.3 mg, 0.20 mmol) at 110 °C for 2 hours. The catalyst was filtered off and the filtrate was diluted with DCM (10 mL) and washed with saturated $NaHCO_3$ solution (15 mL). The aqueous phase was extracted with DCM (3 x 10 mL) and the combined organic layers were washed with brine (50 mL), dried over $MgSO_4$ and concentrated in vacuo. The crude was purified by MPLC (EtOAc/MeOH 1:0 to 9:1) to afford the product (2.9 mg, 5%). **$^1$H-NMR** (600 MHz, $CD_2Cl_2$): δ 8.75 (d, *J* = 6.5 Hz, 2H), 7.53 (d, *J* = 6.5 Hz, 2H), 6.80 (d, *J* = 6.1 Hz, 1H), 6.01 (s, 1H), 5.55 (d, *J* = 17.2 Hz, 1H), 5.34 (d, *J* = 17.2 Hz, 1H), 5.26 (dd, *J* = 6.1, 5.0 Hz, 1H), 5.02 (dd, *J* = 6.7, 5.0 Hz, 1H), 3.55-3.50 (m, 1H), 3.03 (dd, *J* = 10.7, 6.7 Hz, 1H), 2.25 (s, 3H), 1.43 (d, *J* = 6.3 Hz, 2H). **$^{13}$C-NMR** (151 MHz, $CD_2Cl_2$): δ 169.1, 161.7, 156.0, 150.2, 148.7, 143.6, 124.2, 108.0, 98.3, 97.5, 79.8, 73.7, 46.8, 43.9, 21.6, 19.7. **HRMS**-ESI (m/z): $[M + H]^+$ calculated for $C_{18}H_{19}N_2O_3^+$, 311.1317; found, 311.1390.

**(±) 6-bromo-3-methyl-5a,9a-dihydro-1H,9H-furo[3,2-c:4,5-c']dipyran-1-one (11a)**

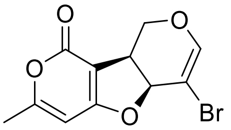

To a stirred solution of **9aa** (70 mg, 0.34 mmol) in dry acetonitrile (3 mL) was added NBS (66.5 mg, 0.37 mmol, 1.1 equiv) and AgNO3 (115.3 mg, 0.68 mmol, 2 equiv) successively. The reaction tube was sealed and the mixture was stirred for 2 h at 80 °C. The reaction mixture was filtered and the filtrate was evaporated to afford a crude product which was purified by silica gel column chromatography (Pet. Ether/EtOAc 4:1). The product was obtained as an off-white powder (56.5 mg, 58%). **$^1$H-NMR** (700 MHz, $CDCl_3$): δ 7.01 (s, 1H), 6.01 (s, 1H), 5.15 (d, *J* = 7.4 Hz, 1H), 4.42-4.37 (m, 1H), 3.65-3.56 (m, 2H), 2.28 (s, 3H). **$^{13}$C-NMR** (176 MHz, $CDCl_3$): δ 171.8, 166.7, 161.6, 149.8, 99.7, 96.2, 96.1, 82.3, 65.0, 38.6, 20.7. **HRMS**-ESI (m/z): $[M + H]^+$ calculated for $C_{11}H_{10}O_4{}^{79}Br^+$, 284.9757; found, 284.9760; calculated for $C_{11}H_{10}O_4{}^{81}Br^+$, 286.9737; found, 286.9734.

**(±) 3-methyl-6-(4-(trifluoromethyl)phenyl)-5a,9a-dihydro-1H,9H-furo[3,2-c:4,5-c']dipyran-1-one (11b)**

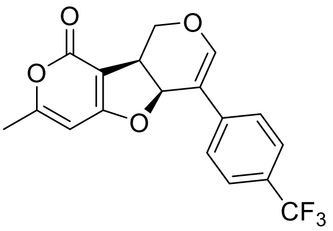


A microwave vial was flushed with Argon and loaded with **11a** (10.0 mg, 0.04 mmol), (4-(trifluoromethyl)phenyl)boronic acid (13.3 mg, 0.07 mmol, 2 equiv), NaO$^t$Bu (6.7 mg, 0.07 mmol, 2 equiv), $Pd(OAc)_2$ (0.4 mg, 5 mol%), Xphos (1.7 mg, 10 mol%) and toluene (0.04 M). The mixture was irradiated at 130 °C for 40 minutes. After cooling back to room temperature the mixture was directly loaded on a silica column and eluted with Pet. Ether/EtOAc (9:1 to 7:3) and repurified by prep. HPLC to afford the product (6.1 mg, 50%). **$^1$H-NMR** (700 MHz DMSO-d$_6$): δ 7.73-7.70 (m, 4H), 7.63 (s, 1H), 6.33 (s, 1H), 5.93 (d, *J* = 7.5 Hz, 1H), 4.31 (dd, *J* = 10.2 Hz, 4.3 Hz, 1H), 3.62-3.58 (m, 1H), 3.56 (dd, *J* = 10.4 Hz, 10.2 Hz, 1H), 2.23 (s, 3H). **$^{13}$C-NMR** (176 MHz, DMSO-d$_6$): δ 172.1, 167.0, 161.1, 150.2, 140.9, 127.4, 125.9, 125.6, 124.1, 111.4, 99.4, 96.1, 79.0, 64.8, 36.4, 20.4. **UHPLC-MS**-ESI (m/z): 351.0 $[M + H]^+$.

**(±) 7,8-dimethyl-3-phenyl-4,8-dihydro-1H-pyrano[3',4':4,5]furo[3,2-c]pyridin-9(3H)-one (12a)**

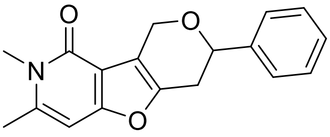


According to GP4, **9ac** (40.0 mg, 0.18 mmol) was reacted with phenylboronic acid (44.5 mg, 0.36 mmol, 2 equiv) and $Pd(OAc)_2$ (20.5 mg, 0.09 mmol, 0.5 equiv) at room temperature for 18 hours. The crude was purified by MPLC (cyclohexane/EtOAc 1:0 to 0:1) to afford the product (35.0 mg, 64%). **$^1$H-NMR** (700 MHz DMSO-d$_6$): δ 7.47 (d, *J* = 7.4 Hz, 2H), 7.39 (t, *J* = 7.4 Hz, 2H), 7.32 (t, *J* = 7.4 Hz, 1H), 6.63 (d, *J* = 0.6 Hz, 1H), 4.96 (dd, *J* = 14.7, 1.8 Hz, 1H), 4.89 (dt, *J* = 14.7, 2.8 Hz, 1H), 4.79 (dd, *J* = 10.3, 3.5 Hz, 1H), 3.47 (s, 3H), 3.10-3.05 (m, 1H), 2.88 (ddt, *J* = 15.8, 10.3, 2.6 Hz, 1H), 2.43 (s, 3H). **$^{13}$C-NMR** (176 MHz, DMSO-d$_6$): δ 158.9, 158.1, 148.2, 143.6, 141.3, 128.3, 127.7, 126.0, 113.6, 110.4, 94.9, 75.3, 63.0, 31.2, 30.1, 20.9. **HRMS**-ESI (m/z): $[M + H]^+$ calculated for $C_{18}H_{18}NO_3^+$, 296.1281; found, 296.1291.

**(±) 3-(4-methoxyphenyl)-7,8-dimethyl-4,8-dihydro-1H-pyrano[3',4':4,5]furo[3,2-c]pyridin-9(3H)-one (12b)**

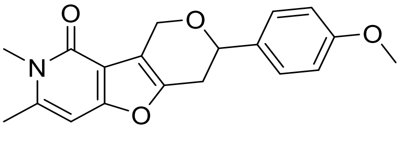


According to GP4, **9ac** (40.0 mg, 0.18 mmol) was reacted with (4-methoxyphenyl)boronic acid (55.5 mg, 0.36 mmol, 2 equiv) and $Pd(OAc)_2$ (20.5 mg, 0.09 mmol, 0.5 equiv) at room

temperature for 18 hours. The crude was purified by MPLC (cyclohexane/EtOAc 1:0 to 0:1) and repurified by prep. HPLC to afford the product (24.9 mg, 42%). **$^1$H-NMR** (700 MHz, $CDCl_3$): δ 7.37 (d, *J* = 8.5 Hz, 2H), 6.92 (d, *J* = 8.7 Hz, 2H), 6.41 (s, 1H), 5.18 (dd, *J* = 14.9, 2.0 Hz, 1H), 5.00 (dt, *J* = 14.9, 2.9 Hz, 1H), 4.70 (dd, *J* = 9.8, 3.8 Hz, 1H), 3.82 (s, 3H), 3.59 (s, 3H), 3.03-2.97 (m, 1H), 2.96-2.92 (m, 1H), 2.44 (s, 2H). **$^{13}$C-NMR** (176 MHz, $CDCl_3$): δ 160.4, 158.9, 159.5, 150.0, 142.1, 133.4, 127.6, 114.5, 114.1, 112.1, 96.6, 76.0, 64.0, 55.5, 31.9, 31.0, 21.8. **HRMS**-ESI (m/z): $[M + H]^+$ calculated for $C_{19}H_{20}NO_4^+$, 326.1387; found, 326.1397.

**(±) 7,8-dimethyl-3-(4-(trifluoromethyl)phenyl)-4,8-dihydro-1H-pyrano[3',4':4,5]furo[3,2-c]pyridin-9(3H)-one (12c)**

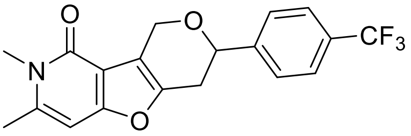


According to GP4, **9ac** (20.0 mg, 0.09 mmol) was reacted with (4-(trifluoromethyl)phenyl)boronic acid (34.7 mg, 0.18 mmol, 2 equiv) and $Pd(OAc)_2$ (10.2 mg, 0.05 mmol, 0.5 equiv) at room temperature for 18 hours. The crude was purified by MPLC (cyclohexane/EtOAc 1:0 to 0:1) and repurified by prep. HPLC to afford the product (12.9 mg, 39%). **$^1$H-NMR** (500 MHz, $CDCl_3$): δ 7.65 (d, *J* = 8.2 Hz, 2H), 7.58 (d, *J* = 8.2 Hz, 2H), 6.39 (s, 1H), 5.25 (dd, *J* = 15.1, 2.0 Hz, 1H), 5.03 (dt, *J* = 15.1, 2.8 Hz, 1H), 4.81 (dd, *J* = 10.0, 3.8 Hz, 1H), 3.58 (s, 3H), 3.06-2.98 (m, 1H), 2.93 (ddt, *J* = 16.0, 10.0, 2.7 Hz, 1H), 2.44 (s, 3H). **$^{13}$C-NMR** (126 MHz, $CDCl_3$): δ 160.3, 158.9, 148.0, 145.3, 142.3, 130.4, 126.3, 125.7, 123.1, 114.6, 111.9, 96.2, 75.6, 64.2, 32.1, 30.8, 21.9. **HRMS**-ESI (m/z): $[M + H]^+$ calculated for $C_{19}H_{17}F_3NO_3^+$, 364.1155; found, 364.1155.

**(±) 2-(7,8-dimethyl-9-oxo-3,4,8,9-tetrahydro-1H-pyrano[3',4':4,5]furo[3,2-c]pyridin-3-yl)benzonitrile (12d)**

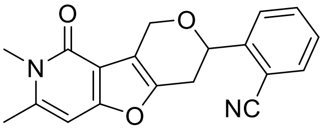


According to GP4, **9ac** (25.0 mg, 0.11 mmol) was reacted with (2-cyanophenyl)boronic acid (33.5 mg, 0.23 mmol, 2 equiv) and $Pd(OAc)_2$ (12.8 mg, 0.06 mmol, 0.5 equiv) at 50 °C for 18 hours. The crude was purified by MPLC (cyclohexane/EtOAc 1:0 to 0:1) and repurified by prep. HPLC to afford the product (28.7 mg, 79%). **$^1$H-NMR** (700 MHz, $CDCl_3$): δ 7.77 (d, *J* = 8.0 Hz, 1H), 7.70-7.65 (m, 2H), 7.43 (td, *J* = 7.6, 1.2 Hz, 1H), 6.43 (s, 1H), 5.27 (dd, *J* = 15.0, 2.0 Hz, 1H), 5.10 (dd, *J* = 10.4, 3.6 Hz, 1H), 5.07 (ddd, *J* = 15.0, 3.3, 2.4 Hz, 1H), 3.60 (s, 3H), 3.16 (dt, *J* = 16.1, 3.0 Hz, 1H), 2.88 (dddt, *J* = 13.3, 10.4, 5.5, 2.3 Hz, 1H), 2.46 (s, 3H). **$^{13}$C-NMR** (176 MHz, $CDCl_3$): δ 160.4, 159.1, 147.9, 145.2, 142.4, 133.6, 132.9, 128.5, 126.7,

117.4, 114.4, 112.0, 110.3, 96.7, 74.1, 64.3, 31.7, 31.0, 21.8. **HRMS**-ESI (m/z): $[M + H]^+$ calculated for $C_{19}H_{17}N_2O_3^+$, 321.1234; found, 321.1235.

**(±) 3-(1H-indol-5-yl)-7,8-dimethyl-4,8-dihydro-1H-pyrano[3',4':4,5]furo[3,2-c]pyridin-9(3H)-one (12e)**

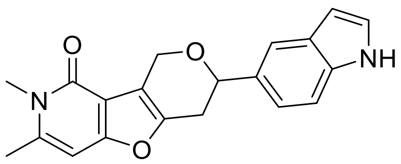


According to GP4, **9ac** (25.0 mg, 0.11 mmol) was reacted with (1H-indol-5-yl)boronic acid (36.7 mg, 0.23 mmol, 2 equiv) and $Pd(OAc)_2$ (12.8 mg, 0.06 mmol, 0.5 equiv) at 50 °C for 18 hours. The crude was purified by MPLC (cyclohexane/EtOAc 1:0 to 0:1) and repurified by prep. HPLC to afford the product (18.9 mg, 50%). **$^1$H-NMR** (500 MHz, $CDCl_3$): δ 8.23 (s, 1H),7.68 (s, 1H), 7.41 (d, *J* = 8.5 Hz, 1H), 7.30 (dd, *J* = 8.5, 1.7 Hz, 1H), 7.25-7.20 (m, 1H), 6.57-6.54 (m, 1H), 6.36 (s, 1H), 5.24 (dd, *J* = 15.0, 2.0 Hz, 1H), 5.06 (dt, *J* = 15.0, 2.8 Hz, 1H), 4.84 (dd, *J* = 10.0, 3.6 Hz, 1H), 3.54 (s, 3H), 3.16-3.08 (m, 1H), 3.00 (dt, *J* = 16.4, 3.1 Hz, 1H), 2.40 (s, 3H). **$^{13}$C-NMR** (126 MHz, $CDCl_3$): δ 160.4, 158.7, 149.1, 141.9, 135.7, 132.7, 128.0, 125.0, 120.6, 118.6, 114.6, 112.0, 111.3, 102.9, 96.3, 64.1, 32.4, 30.8, 21.8. **HRMS**-ESI (m/z): $[M + H]^+$ calculated for $C_{20}H_{19}N_2O_3^+$, 335.1390; found, 335.1391.

**(±) 3-(6-fluoropyridin-3-yl)-7,8-dimethyl-4,8-dihydro-1H-pyrano[3',4':4,5]furo[3,2-c]pyridin-9(3H)-one (12f)**

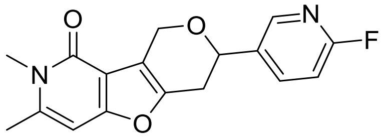


According to GP4, **9ac** (40.0 mg, 0.18 mmol) was reacted with (6-fluoropyridin-3-yl)boronic acid (51.4 mg, 0.36 mmol, 2 equiv) and $Pd(OAc)_2$ (20.5 mg, 0.09 mmol, 0.5 equiv) at room temperature for 18 hours. The crude was purified by MPLC (cyclohexane/EtOAc 1:0 to 0:1) to afford the product (9.4 mg, 17%). **$^1$H-NMR** (500 MHz, $CDCl_3$): δ 8.27 (d, *J* = 2.5 Hz, 1H), 7.92 (td, *J* = 8.3, 2.5 Hz, 1H), 6.97 (dd, *J* = 8.3, 2.9 Hz, 1H), 6.39 (s, 1H), 5.22 (dd, *J* = 15.1, 1.9 Hz, 1H), 5.03 (dt, *J* = 15.1, 2.8 Hz, 1H), 4.80 (dd, *J* = 9.8, 4.1 Hz, 1H), 3.55 (s, 3H), 3.04-2.91 (m, 2H), 2.44 (s, 3H). **$^{13}$C-NMR** (126 MHz, $CDCl_3$): δ 164.3, 162.4, 160.1, 158.8, 147.4, 145.4, 142.3, 139.2, 135.4, 114.5, 111.7, 109.5, 95.1, 73.3, 31.9, 30.7, 21.7. **HRMS**-ESI (m/z): $[M + H]^+$ calculated for $C_{17}H_{16}FN_2O_3^+$, 315.1140; found, 315.1145.

**(±) 7,8-dimethyl-3-(thiophen-3-yl)-4,8-dihydro-1H-pyrano[3',4':4,5]furo[3,2-c]pyridin-9(3H)-one (12g)**

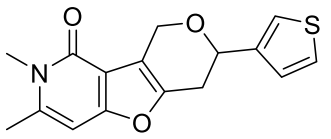

According to GP4, **9ac** (35.0 mg, 0.16 mmol) was reacted with thiophen-3-ylboronic acid (40.9 mg, 0.32 mmol, 2 equiv) and Pd(OAc)$_2$ (17.9 mg, 0.08 mmol, 0.5 equiv) at room temperature for 18 hours. The crude was purified by MPLC (cyclohexane/EtOAc 1:0 to 0:1) to afford the product (2.6 mg, 5%). **$^1$H-NMR** (700 MHz, CDCl$_3$): δ 7.35 (dd, *J* = 5.0, 3.0 Hz, 1H), 7.34-7.30 (m, 1H), 7.17 (dd, *J* = 5.0, 1.3 Hz, 1H), 6.38 (s, 1H), 5.17 (dt, *J* = 14.9, 1.3 Hz, 1H), 5.02 (dt, *J* = 14.9, 2.8 Hz, 1H), 4.87 (dd, *J* = 8.7, 4.7 Hz, 1H), 3.57 (s, 3H), 3.08-3.01 (m, 2H), 2.43 (s, 3H). **HRMS**-ESI (m/z): [M + H]$^+$ calculated for $C_{16}H_{16}NO_3S^+$, 302.0845; found, 302.0847.

**(±) 3-(4-methoxyphenyl)-7-methyl-8-(thiophen-2-ylmethyl)-4,8-dihydro-1H-pyrano[3',4':4,5]furo[3,2-c]pyridin-9(3H)-one (12h)**

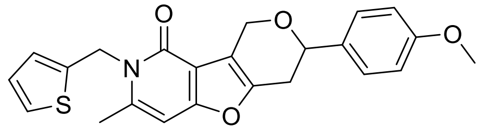

According to GP2, **7a** (100.0 mg, 0.35 mmol) was reacted with **5j** (70.0 mg, 0.32 mmol) at 100 °C for 1 hour. After filtration over celite the crude was purified by FC (Hep/EtOAc 1:0 to 1:1) and an aliquot of the product (35.0 mg, 0.12 mmol) was directly reacted according to GP4, (35.0 mg, 0.12 mmol) with (4-methoxyphenyl)boronic acid (35.3 mg, 0.23 mmol, 2 equiv), Pd(OAc)$_2$ (5.2 mg, 20 mol%) and Cu(OAc)$_2$ (42.2 mg, 0.23 mmol, 2 equiv) at 50 °C for 18 hours under $O_2$ atmosphere. The crude was purified by MPLC (cyclohexane/EtOAc 1:0 to 0:1) and repurified by prep. HPLC to afford the product (5.7 mg, 12%). **$^1$H-NMR** (700 MHz, CDCl$_3$): δ 7.38 (d, *J* = 8.5 Hz, 2H), 7.21 (d, *J* = 5.0 Hz, 1H), 7.00 (d, *J* = 3.1 Hz, 1H), 6.97-6.95 (m, 1H), 6.93 (d, *J* = 8.5 Hz, 2H), 6.33 (s, 1H), 5.52-5.40 (m, 2H), 3.80 (s, 3H), 5.22 (d, *J* = 15.0 Hz, 1H), 5.05-5.02 (m, 1H), 4.70 (dd, *J* = 9.8, 3.8 Hz, 1H), 3.03-2.98 (m, 1H), 2.96-2.93 (m, 1H), 2.50 (s, 3H). **$^{13}$C-NMR** (176 MHz, CDCl$_3$): δ 160.0, 159.5, 159.0, 148.9, 141.6, 139.5, 133.4, 127.6, 126.8, 126.5, 125.6, 114.4, 114.1, 112.4, 96.9, 76.0, 64.4, 55.5, 42.9, 32.2, 21.3. **HRMS**-ESI (m/z): [M + H]$^+$ calculated for $C_{23}H_{22}NO_4S^+$, 408.1191; found, 408.1264.

### 1.3.3. Synthesis of General Scaffold B Derivatives

**(±) 7,8-dimethyl-4,4a,8,9b-tetrahydro-2H-pyrano[2',3':4,5]furo[3,2-c]pyridine-3,9-dione (13aa)**

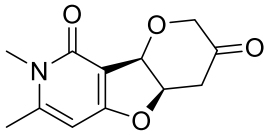

According to GP6, **8a** (50.0 mg, 0.32 mmol) was reacted with **5b** (44.5 mg, 0.32 mmol) employing allylpalladium(II) chloride dimer (2.5 mol%) and Xantphos (7.5 mol%) as catalyst. Purification by MPLC (cyclohexane/EtOAc 1:0 to 0:1) afforded the product (49.5 mg, 66%). **$^1$H NMR** (700 MHz, $CDCl_3$): δ 5.85 (s, 1H), 5.60 (d, *J* = 7.4 Hz, 1H), 5.20 (dt, *J* = 7.4, 3.9 Hz, 1H), 4.02 (d, *J* = 18.4 Hz, 1H), 3.71 (d, *J* = 18.4 Hz, 1H), 3.51 (s, 3H), 3.07 (dd, *J* = 16.1, 3.9 Hz, 1H), 2.96 (dd, *J* = 16.1, 3.9 Hz, 1H), 2.37 (s, 3H). **$^{13}$C NMR** (176 MHz, $CDCl_3$): δ 207.7, 169.2, 161.7, 151.6, 102.8, 94.6, 82.0, 74.8, 68.9, 39.7, 30.8, 22.1. **HRMS**-ESI (m/z): $[M + H]^+$ calculated for $C_{12}H_{14}O_4N^+$,236.09173; found, 236.09173.

**(±) 8-benzyl-7-methyl-4,4a,8,9b-tetrahydro-2H-pyrano[2',3':4,5]furo[3,2-c]pyridine-3,9-dione (13ab)**

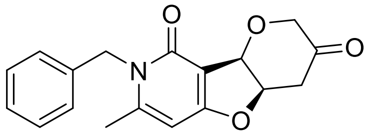

According to GP6, **8a** (50.0 mg, 0.32 mmol) was reacted with **5c** (68.9 mg, 0.32 mmol) employing allylpalladium(II) chloride dimer (2.5 mol%) and Xantphos (7.5 mol%) as catalyst. Purification by MPLC (cyclohexane/EtOAc 1:0 to 0:1) afforded the product (73.0 mg, 73%). **$^1$H NMR** (600 MHz, $CDCl_3$): δ 7.32 (t, *J* = 7.4 Hz, 2H), 7.28 (d, *J* = 7.4 Hz, 1H), 7.14 (d, *J* = 7.4 Hz, 2H), 5.96 (s, 1H), 5.68 (d, *J* = 7.4 Hz, 1H, 5.49 (d, *J* = 15.7 Hz, 1H), 5.28 (dt, *J* = 7.4, 3.9 Hz, 1H), 5.24 (d, *J* = 15.7 Hz, 1H), 4.05 (d, *J* = 18.4 Hz, 1H), 3.75 (d, *J* = 18.4 Hz, 1H), 3.10 (dd, *J* = 16.1, 3.9 Hz, 1H), 3.00 (dd, *J* = 16.1, 3.9 Hz, 1H), 2.33 (s, 1H). **$^{13}$C NMR** (151 MHz, $CDCl_3$): δ 207.2, 169.8, 162.1, 152.1, 135.9, 128.9, 127.6, 126.3, 103.3, 96.2, 82.4, 74.3, 68.8, 47.1, 39.5, 21.5. **HRMS**-ESI (m/z): $[M + H]^+$ calculated for $C_{18}H_{18}O_4N^+$,312.12303; found, 312.12330.

**(±) 8-(4-fluorobenzyl)-7-methyl-4,4a,8,9b-tetrahydro-2H-pyrano[2',3':4,5]furo[3,2-c]pyridine-3,9-dione (13ac)**

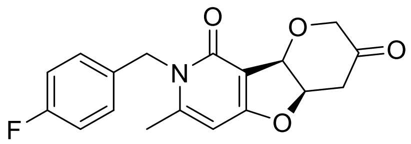

According to GP6, **8a** (50.0 mg, 0.32 mmol) was reacted with **5k** (74.7 mg, 0.32 mmol) employing allylpalladium(II) chloride dimer (2.5 mol%) and Xantphos (7.5 mol%) as catalyst. Purification by MPLC (cyclohexane/EtOAc 1:0 to 0:1) afforded the product (72.5 mg,

69%). **¹H NMR** (500 MHz, Chloroform-d): δ 7.16 (m, 2H), 7.00 (m, 2H), 5.86 (d, *J* = 1.1 Hz, 1H), 5.64 (d, *J* = 7.4 Hz, 1H), 5.42 (d, *J* = 15.7 Hz, 1H), 5.25 (dt, *J* = 7.4, 3.9 Hz, 1H), 5.15 (d, *J* = 15.7 Hz, 1H), 4.07 (d, *J* = 18.3 Hz, 1H), 3.76 (d, *J* = 18.3 Hz, 1H), 3.10 (dd, *J* = 16.1, 3.9 Hz, 1H), 2.99 (dd, *J* = 16.1, 3.9 Hz, 1H), 2.30 (s, 3H). **¹³C NMR** (126 MHz, $CDCl_3$): δ 207.7, 169.5, 163. 2, 161. 7, 151.8, 132.4, 116.0, 115.8, 103.1, 95.4, 82.2, 74.8, 69.0, 46.2, 39.7, 21.7. **HRMS**-ESI (m/z): $[M + H]^+$ calculated for $C_{18}H_{17}O_4NF^+$,330.11361; found, 330.11400.

**(±) 8-(2-chlorobenzyl)-7-methyl-4,4a,8,9b-tetrahydro-2H-pyrano[2',3':4,5]furo[3,2-c]pyridine-3,9-dione (13ad)**

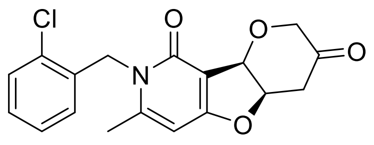

According to GP6, **8a** (50.0 mg, 0.32 mmol) was reacted with **5l** (79.9 mg, 0.32 mmol) employing allylpalladium(II) chloride dimer (2.5 mol%) and Xantphos (7.5 mol%) as catalyst. Purification by MPLC (cyclohexane/EtOAc 1:0 to 0:1) afforded the product (96.8 mg, 87%). **¹H NMR** (500 MHz, $CDCl_3$): δ 7.39 (dd, *J* = 7.6, 1.6 Hz, 1H), 7.19 (dtd, *J* = 16.7, 7.6, 1.6 Hz, 2H), 6.78 (dd, *J* = 7.6, 1.6 Hz, 1H), 5.90 (s, 1H), 5.66 (d, *J* = 7.4 Hz, 1H), 5.51 (d, *J* = 17.3 Hz, 1H), 5.31 (d, *J* = 17.3 Hz, 1H), 5.27 (m, 1H), 4.08 (d, *J* = 18.3 Hz, 1H), 3.78 (d, *J* = 18.3 Hz, 1H), 3.11 (dd, *J* = 16.1, 3.9 Hz, 1H), 3.01 (dd, *J* = 16.1, 3.9 Hz, 1H), 2.24 (s, 3H). **¹³C NMR** (126 MHz, $CDCl_3$): δ 207.7, 169.7, 161.6, 151.9, 133.9, 132.5, 129.7, 128.7, 127.6, 126.5, 102.9, 95.5, 82.3, 74.8, 69.0, 44.4, 39.8, 21.4. **HRMS**-ESI (m/z): $[M + H]^+$ calculated for $C_{18}H_{17}O_4NCl^+$,346.08406; found, 346.08453.

**(±) 7-methyl-8-(pyridin-2-ylmethyl)-4,4a,8,9b-tetrahydro-2H-pyrano[2',3':4,5]furo[3,2-c]pyridine-3,9-dione (13ae)**

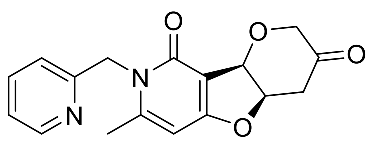

According to GP6, **8a** (50.0 mg, 0.32 mmol) was reacted with **5m** (69.2 mg, 0.32 mmol) employing allylpalladium(II) chloride dimer (2.5 mol%) and Xantphos (7.5 mol%) as catalyst. Purification by MPLC (cyclohexane/EtOAc 1:0 to 0:1) afforded the product (71.6 mg, 72%). **¹H NMR** (700 MHz, $CDCl_3$): δ 8.51 (d, *J* = 4.1 Hz, 1H), 7.63 (td, *J* = 7.6, 1.5 Hz, 1H), 7.27 (d, *J* = 4.8 Hz, 1H), 7.18 (ddd, *J* = 7.6, 4.8, 1.5 Hz, 1H), 5.88 (s, 1H), 5.61 (d, *J* = 7.4 Hz, 1H), 5.49 (d, *J* = 16.0 Hz, 1H), 5.28 (d, *J* = 16.0 Hz, 1H), 5.23 (dt, *J* = 7.4, 3.9 Hz, 1H), 4.03 (d, *J* = 18.3 Hz, 1H), 3.75 (d, *J* = 18.3 Hz, 1H), 3.07 (dd, *J* = 16.1, 3.9 Hz, 1H), 2.97 (dd, *J* = 16.1, 3.9 Hz, 1H), 2.43 (s, 3H). **¹³C NMR** (176 MHz, $CDCl_3$) δ 207.6, 169.6, 161.5, 156.3, 152.5, 149.4, 137.1, 122.7, 122.2, 102.8, 95.2, 82.1, 77.3, 77.2, 76.9, 74.7, 68.9, 60.5, 48.7, 39.7, 22.1, 14.3. **HRMS**-ESI (m/z): $[M + H]^+$ calculated for $C_{17}H_{17}O_4N_2^+$,313.11828; found, 313.11845.

**(±) 8-((2-chloropyridin-4-yl)methyl)-7-methyl-4,4a,8,9b-tetrahydro-2H-pyrano[2',3':4,5]furo[3,2-c]pyridine-3,9-dione (13af)**

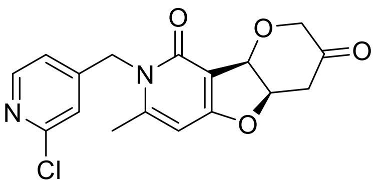


According to GP6, **8a** (50.0 mg, 0.32 mmol) was reacted with **5r** (80.3 mg, 0.32 mmol) employing allylpalladium(II) chloride dimer (2.5 mol%) and Xantphos (7.5 mol%) as catalyst. Purification by MPLC (cyclohexane/EtOAc 1:0 to 0:1) afforded the product (81.2 mg, 73%). **$^1$H NMR** (600 MHz, $CDCl_3$): δ 8.37 (d, *J* = 5.2 Hz, 1H), 7.09 (s, 1H), 7.04 (d, *J* = 5.2 Hz, 1H), 6.02 (s, 1H), 5.64 (d, *J* = 7.4 Hz, 1H), 5.50 (d, *J* = 15.9 Hz, 1H), 5.30 (dt, *J* = 7.4, 4.0 Hz, 1H), 5.15 (d, *J* = 15.9 Hz, 1H), 4.04 (d, *J* = 18.3 Hz, 1H), 3.73 (d, *J* = 18.3 Hz, 1H), 3.10 (dd, *J* = 16.1, 4.0 Hz, 1H), 3.01 (dd, *J* = 16.1, 4.0 Hz, 1H), 2.31 (s, 3H). **$^{13}$C NMR** (151 MHz, $CDCl_3$): δ 207.0, 170.3, 161.8, 152.2, 151.3, 150.0, 149.4, 122.1, 120.4, 103.7, 97.0, 82.8, 74.4, 69.1, 46.0, 39.6, 21.7. **HRMS**-ESI (m/z): $[M + H]^+$ calculated for $C_{17}H_{16}O_4N_2Cl^+$,347.07931; found, 347.07978.

**(±) 7-methyl-8-((3-methylpyridin-4-yl)methyl)-4,4a,8,9b-tetrahydro-2H-pyrano[2',3':4,5]furo[3,2-c]pyridine-3,9-dione (13ag)**

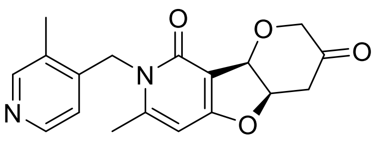


According to GP6, **8a** (50.0 mg, 0.32 mmol) was reacted with **5q** (104.3 mg, 0.32 mmol) employing allylpalladium(II) chloride dimer (2.5 mol%) and Xantphos (7.5 mol%) as catalyst. Purification by MPLC (cyclohexane/EtOAc 1:0 to 0:1) afforded the product (58.0 mg, 56%). **$^1$H NMR** (500 MHz, $CDCl_3$): δ 8.69 (s, 1H), 8.53 (d, *J* = 5.8 Hz, 1H), 7.02 (d, *J* = 5.8 Hz, 1H), 6.04 (s, 1H), 5.61 (d, *J* = 7.6 Hz, 1H), 5.52 (d, *J* = 17.7 Hz, 1H), 5.31 (dt, *J* = 7.6, 4.0 Hz, 1H), 5.16 (d, *J* = 17.7 Hz, 1H), 4.07 (d, *J* = 18.3 Hz, 1H), 3.76 (d, *J* = 18.3 Hz, 1H), 3.12 (dd, *J* = 16.1, 4.0 Hz, 1H), 3.03 (dd, *J* = 16.1, 4.0 Hz, 1H), 2.58 (s, 3H), 2.28 (s, 3H). **$^{13}$C NMR** (176 MHz, $CDCl_3$): δ 207.1, 170.0, 161.1, 152.8, 150.2, 143.7, 142.1, 134.2, 121.7, 96.26, 82.63, 74.62, 69.15, 41.14, 39.67, 31.08, 21.49, 14.27. **HRMS**-ESI (m/z): $[M + H]^+$ calculated for $C_{18}H_{19}O_4N_2^+$,327.13393; found, 327.13412.

**(±) 8-(2-(5-methoxy-1H-indol-3-yl)ethyl)-7-methyl-4,4a,8,9b-tetrahydro-2H-pyrano[2',3':4,5]furo[3,2-c]pyridine-3,9-dione (13ah)**

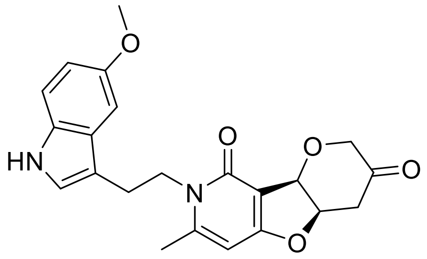

According to GP6, **8a** (12.5 mg, 0.08 mmol) was reacted with **5p** (23.8 mg, 0.08 mmol) employing allylpalladium(II) chloride dimer (2.5 mol%) and Xantphos (7.5 mol%) as catalyst. Purification by MPLC (cyclohexane/EtOAc 1:0 to 0:1) afforded the product (23.7 mg, 75%). **$^1$H NMR** (700 MHz, $CDCl_3$): δ 8.15 (s, 1H), 7.25 (s, 1H), 7.12 (d, *J* = 2.4 Hz, 1H), 6.97 (d, *J* = 2.4 Hz, 1H), 6.85 (dd, *J* = 8.8, 2.4 Hz, 1H), 5.76 (s, 1H), 5.64 (d, *J* = 7.4 Hz, 1H), 5.21 (dt, *J* = 7.4, 3.9 Hz, 1H), 4.26 (ddt, *J* = 28.7, 13.8, 6.9 Hz, 2H), 4.05 (d, *J* = 18.4 Hz, 1H), 3.86 (s, 3H), 3.75 (d, *J* = 18.4 Hz, 1H), 3.14 (t, *J* = 7.6 Hz, 2H), 3.08 (dd, *J* = 16.2, 4.0 Hz, 1H), 2.98 (dd, *J* = 16.2, 4.0 Hz, 1H), 2.19 (s, 3H). **$^{13}$C NMR** (176 MHz, $CDCl_3$) δ 207.7, 169.3, 161.6, 154.3, 151.6, 131.6, 127.9, 123.2, 112.5, 112.3, 112.1, 103.2, 100.8, 94.8, 82.1, 74.8, 69.0, 56.1, 45.4, 39.7, 24.6, 21.6. **HRMS**-ESI (m/z): $[M + H]^+$ calculated for $C_{22}H_{23}O_5N_2^+$ ,395.16015; found, 395.15963.

**(±) 2,7-dimethyl-4,4a,8,9b-tetrahydro-2H-pyrano[2',3':4,5]furo[3,2-c]pyridine-3,9-dione (13ba)**

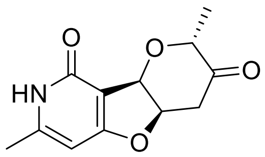

According to GP6, **8b** (25.5 mg, 0.15 mmol) was reacted with **5a** (18.8 mg, 0.15 mmol). Purification by MPLC (cyclohexane/EtOAc 1:0 to 0:1) afforded the product (19.7 mg, 56%). **$^1$H-NMR** (700 MHz, $CD_2Cl_2$): δ 11.83 (s, 1H), 5.81 (s, 1H), 5.55 (d, *J* = 7.2 Hz, 1H), 5.24 (dt, *J* = 7.2, 3.2 Hz, 1H), 3.64 (q, *J* = 6.8 Hz, 1H), 3.05 (dd, *J* = 16.5, 3.4 Hz, 1H), 2.89 (dd, *J* = 16.5, 3.1 Hz, 1H), 2.33 (s, 3H), 1.25 (d, *J* = 6.8 Hz, 3H). **$^{13}$C-NMR** (176 MHz, $CD_2Cl_2$): δ 209.6, 172.3, 163.4, 151.1, 103.5, 94.1, 83.1, 73.9, 73.5, 39.1, 19.8, 15.9. **HRMS**-ESI (m/z): $[M + H]^+$ calculated for $C_{12}H_{14}NO_4^+$, 236.0917; found, 236.0916.

**(±) 2,7,8-trimethyl-4,4a,8,9b-tetrahydro-2H-pyrano[2',3':4,5]furo[3,2-c]pyridine-3,9-dione (13bb)**

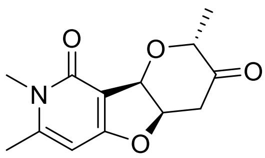

According to GP6, **8b** (24.0 mg, 0.14 mmol) was reacted with **5b** (19.6 mg, 0.14 mmol). Purification by MPLC (cyclohexane/EtOAc 1:0 to 0:1) afforded the product (19.3 mg, 55%). **$^1$H-NMR** (700 MHz, $CD_2Cl_2$): δ 5.82 (s, 1H), 5.53 (d, *J* = 7.4 Hz, 1H), 5.20 (dt, *J* = 7.4, 3.2 Hz, 1H), 3.60 (q, *J* = 6.8 Hz, 1H), 3.45 (s, 3H), 3.04 (ddd, *J* = 16.3, 3.2, 0.6 Hz, 1H), 2.85 (ddd, *J* = 16.3, 3.2, 0.6 Hz, 1H), 2.33

(s, 3H), 1.24 (d, *J* = 6.8 Hz, 3H). **$^{13}$C-NMR** (176 MHz, $CD_2Cl_2$): δ 209.9, 169.4, 161.7, 152.1, 103.2, 94.4, 82.9, 74.8, 73.5, 39.2, 30.8, 22.1, 15.9. **HRMS**-ESI (m/z): $[M + H]^+$ calculated for $C_{13}H_{16}NO_4^+$, 250.1074; found, 250.1071.

**(±) 8-cyclobutyl-2,7-dimethyl-4,4a,8,9b-tetrahydro-2H-pyrano[2',3':4,5]furo[3,2-c]pyridine-3,9-dione (13bc)**

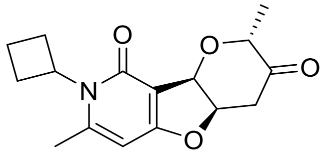

According to GP6, **8b** (25.5 mg, 0.15 mmol) was reacted with **5e** (26.8 mg, 0.15 mmol). Purification by MPLC (cyclohexane/EtOAc 1:0 to 0:1) afforded the product (19.0 mg, 44%). **$^1$H-NMR** (700 MHz, $CD_2Cl_2$): δ 5.84 (s, 1H), 5.57 (d, *J* = 7.5 Hz, 1H), 5.22 (dt, *J* = 7.5, 3.3 Hz, 1H), 4.77 (p, *J* = 9.3 Hz, 1H), 3.60 (q, *J* = 6.9 Hz, 1H), 3.27-3.14 (m, 2H), 3.04 (dd, *J* = 16.4, 3.5 Hz, 1H), 2.86 (dd, *J* = 16.4, 3.1 Hz, 1H), 2.36 (s, 3H), 2.33-2.25 (m, 2H), 2.00-1.94 (m, 1H), 1.80-1.73 (m, 1H), 1.24 (d, *J* = 6.9 Hz, 3H). **$^{13}$C-NMR** (176 MHz, $CD_2Cl_2$): δ 209.8, 169.6, 163.8, 152.3, 105.2, 96.3, 83.3, 74.5, 73.6, 53.8, 39.1, 28.5, 22.8, 15.8, 15.1. **HRMS**-ESI (m/z): $[M + H]^+$ calculated for $C_{16}H_{20}NO_4^+$, 290.1387; found, 290.1386.

**(±) 8-methyl-1,2,3,4,5,6b,10,10a-octahydro-6H-pyrano[2',3':4,5]furo[3,2-c]quinoline-6,9(8H)-dione (13bd)**

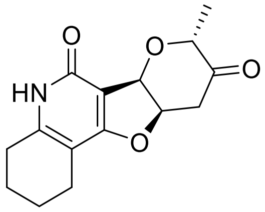

According to GP6, **8b** (25.5 mg, 0.15 mmol) was reacted with 4-hydroxy-5,6,7,8-tetrahydroquinolin-2(1H)-one (24.8 mg, 0.15 mmol). Purification by MPLC (cyclohexane/EtOAc 1:0 to 0:1) afforded the product (13.0 mg, 31%). **$^1$H-NMR** (700 MHz, $CD_2Cl_2$): δ 11.76, 5.55 (d, *J* = 7.2 Hz, 1H), 5.23 (dt, *J* = 7.2, 3.2 Hz, 1H), 3.65 (q, *J* = 6.8 Hz, 1H), 3.05 (dd, *J* = 16.5, 3.4 Hz, 1H), 2.91 (dd, *J* = 16.5, 3.1 Hz, 1H), 2.63 (t, *J* = 6.4 Hz, 2H), 2.34 (t, *J* = 6.3 Hz, 2H), 1.79 (p, *J* = 5.9 Hz, 2H), 1.74-1.69 (m, 2H), 1.25 (d, *J* = 6.8 Hz, 3H). **$^{13}$C-NMR** (176 MHz, $CD_2Cl_2$): δ 209.8, 171.0, 162.6, 148.2, 103.5, 103.1, 82.9, 74.3, 73.4, 39.2, 27.3, 22.1, 22.0, 20.9, 15.8. **HRMS**-ESI (m/z): $[M + H]^+$ calculated for $C_{15}H_{18}NO_4^+$, 276.1230; found. 276.1233.

**(±) 8-(4-fluorobenzyl)-2,7-dimethyl-4,4a,8,9b-tetrahydro-2H-pyrano[2',3':4,5]furo[3,2-c]pyridine-3,9-dione (13be)**

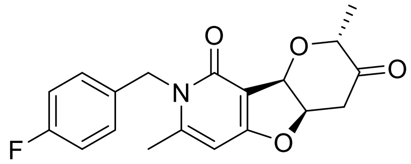

According to GP6, **8b** (25.5 mg, 0.15 mmol) was reacted with **5k** (35.0 mg, 0.15 mmol). Purification by MPLC (cyclohexane/EtOAc 1:0 to 0:1) afforded the product (56.1 mg, 90%). **$^1$H-NMR** (700 MHz, $CD_2Cl_2$): δ 7.14 (dd, *J* = 8.6,

5.4 Hz, 2H), 7.02 (t, *J* = 8.6 Hz, 2H), 5.85 (s, 1H), 5.59 (d, *J* = 7.5 Hz, 1H), 5.43 (d, *J* = 15.7 Hz, 1H), 5.26 (dt, *J* = 7.5, 3.2 Hz, 1H), 5.08 (d, *J* = 15.7 Hz, 1H), 3.66 (q, *J* = 6.9 Hz, 1H), 3.07 (dd, *J* = 16.3, 3.4 Hz, 1H), 2.88 (dd, *J* = 16.3, 3.1 Hz, 1H), 2.26 (s, 3H), 1.27 (d, *J* = 6.9 Hz, 3H). **$^{13}$C-NMR** (176 MHz, $CD_2Cl_2$): δ 209.8, 169.7, 163.1, 161.7, 161.6, 152.2, 133.2, 128.6, 116.0, 103.6, 95.2, 83.1, 74.8, 73.6, 46.2, 39.1, 21.7, 15.9. **HRMS**-ESI (m/z): $[M + H]^+$ calculated for $C_{19}H_{19}FNO_4^+$, 344.1293; found, 344.1293.

**(±) 8-(2-chlorobenzyl)-2,7-dimethyl-4,4a,8,9b-tetrahydro-2H-pyrano[2',3':4,5]furo[3,2-c]pyridine-3,9-dione (13bf)**

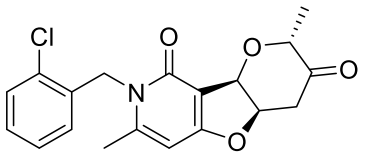


According to GP6, **8b** (25.5 mg, 0.15 mmol) was reacted with **5l** (37.5 mg, 0.15 mmol). Purification by MPLC (cyclohexane/EtOAc 1:0 to 0:1) afforded the product (27.5 mg, 51%). **$^{1}$H-NMR** (700 MHz, $CD_2Cl_2$): δ 7.43 (dd, *J* = 7.9, 1.4 Hz, 1H), 7.26-7.23 (m, 1H), 7.20 (td, *J* = 7.6, 1.4 Hz, 1H), 6.72 (dd, *J* = 7.6, 1.7 Hz, 1H), 5.91 (s, 1H), 5.62 (d, *J* = 7.5 Hz, 1H), 5.50 (d, *J* = 16.1 Hz, 1H), 5.29 (dt, *J* = 7.5, 3.2 Hz, 1H), 5.21 (d, *J* = 16.9 Hz, 1H), 3.68 (q, *J* = 6.8 Hz, 1H), 3.08 (dd, *J* = 16.3, 3.4 Hz, 1H), 2.90 (dd, *J* = 16.3, 3.1 Hz, 1H), 2.21 (s, 3H), 1.28 (d, *J* = 6.8 Hz, 3H). **$^{13}$C-NMR** (176 MHz, $CD_2Cl_2$): δ 209.8, 169.9, 161.7, 152.2, 134.4, 132.7, 130.0, 129.0, 127.7, 126.5, 103.6, 95.3, 83.2, 74.7, 73.6, 44.7. 39.1, 21.4, 15.9. **HRMS**-ESI (m/z): $[M + H]^+$ calculated for $C_{19}H_{19}ClNO_4^+$, 360.0997; found, 360.0999.

**(±) 8-(4-methoxybenzyl)-2,7-dimethyl-4,4a,8,9b-tetrahydro-2H-pyrano[2',3':4,5]furo[3,2-c]pyridine-3,9-dione (13bg)**

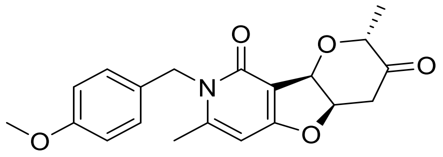


According to GP6, **8b** (50.0 mg, 0.29 mmol) was reacted with **5d** (72.1 mg, 0.29 mmol). Purification by MPLC (cyclohexane/EtOAc 1:0 to 0:1) afforded the product (44.0 mg, 42%). **$^{1}$H-NMR** (500 MHz, $CD_2Cl_2$): δ 7.08 (d, *J* = 8.7 Hz, 2H), 6.86 (d, *J* = 8.7 Hz, 2H), 5.95 (s, 1H), 5.64 (d, *J* = 7.6 Hz, 1H), 5.45 (d, *J* = 15.7 Hz, 1H), 5.29 (dt, *J* = 7.6, 3.4 Hz, 1H), 5.08 (d, *J* = 15.7 Hz, 1H), 3.77 (s, 3H), 3.63 (q, *J* = 6.8 Hz, 1H), 3.08 (dd, *J* = 16.4, 3.4 Hz, 1H), 2.89 (dd, *J* = 16.4, 3.1 Hz, 1H), 2.32 (s, 3H), 1.26 (d, *J* = 6.8 Hz, 3H). **$^{13}$C-NMR** (126 MHz, $CD_2Cl_2$): δ 209.6, 170.1, 162.4, 159.4, 152.7, 128.6, 128.1, 114.5, 104.0, 96.4, 83.4, 74.4, 73.6, 55.6, 46.9, 39.1, 21.8, 15.8. **HRMS**-ESI (m/z): $[M + H]^+$ calculated for $C_{20}H_{22}NO_4^+$, 356.1493; found, 356.1498.

**(±) 2,7-dimethyl-8-(pyridin-4-ylmethyl)-4,4a,8,9b-tetrahydro-2H-pyrano[2',3':4,5]furo[3,2-c]pyridine-3,9-dione (13bh)**

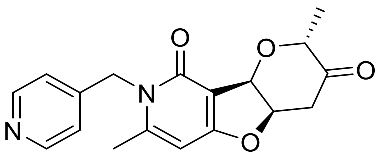

According to GP6, **8b** (25.5 mg, 0.15 mmol) was reacted with **5i** (32.4 mg, 0.15 mmol). Purification by MPLC (EtOAc/MeOH 1:0 to 4:1) afforded the product (18.7 mg, 32%). **$^1$H-NMR** (700 MHz, $CDCl_3$): δ 8.57 (m, 2H), 7.07 (d, *J* = 5.0 Hz, 2H), 5.89 (s, 1H), 5.67 (d, *J* = 7.1 Hz, 1H), 5.54 (d, *J* = 16.0 Hz, 1H), 5.28 (dt, *J* = 7.1, 3.2 Hz, 1H), 5.11 (d, *J* = 16.0 Hz, 1H), 3.69 (q, *J* = 6.7 Hz, 1H), 3.08 (dd, *J* = 16.2, 3.4 Hz, 1H), 2.94 (dd, *J* = 16.2, 3.1 Hz, 1H), 2.26 (s, 1H), 1.33 (d, *J* = 6.7 Hz, 3H). **$^{13}$C-NMR** (176 MHz, $CDCl_3$): δ 209.7, 169.6, 161.4, 151.2, 150.1, 146.3, 121.6, 103.6, 95.7, 82.9, 74.4, 73.6, 45.9, 38.9, 21.6, 16.0. **HRMS**-ESI (m/z): $[M + H]^+$ calculated for $C_{18}H_{19}N_2O_4^+$, 327.1339; found, 327.1339.

**(±) 8-((2-chloropyridin-4-yl)methyl)-2,7-dimethyl-4,4a,8,9b-tetrahydro-2H-pyrano[2',3':4,5]furo[3,2-c]pyridine-3,9-dione (13bi)**

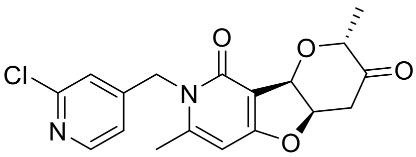

According to GP6, **8b** (25.5 mg, 0.15 mmol) was reacted with **5r** (37.6 mg, 0.15 mmol). Purification by MPLC (cyclohexane/EtOAc 1:0 to 0:1) afforded the product (17.3 mg, 32%). **$^1$H-NMR** (700 MHz, $CD_2Cl_2$): δ 8.31 (d, *J* = 5.2 Hz, 1H), 7.04 (s, 1H), 7.02 (dd, *J* = 5.2, 1.5 Hz, 1H), 5.90 (s, 1H), 5.59 (d, *J* = 7.5 Hz, 1H), 5.49-5.46 (m, 1H), 5.29 (dt, *J* = 7.5, 3.2 Hz, 1H), 5.07 (d, *J* = 16.4 Hz, 1H), 3.65 (q, *J* = 6.8 Hz, 1H), 3.08 (dd, *J* = 16.3, 3.4 Hz, 1H), 2.90 (dd, *J* = 16.3, 3.1 Hz, 1H), 2.23 (s, 3H), 1.28 (d, *J* = 6.8 Hz, 3H). **$^{13}$C-NMR** (176 MHz, $CD_2Cl_2$): δ 209.6, 170.0, 161.3, 152.5, 151.6, 150.5, 149.9, 122.0, 120.7, 103.7, 95.8, 83.3, 74.7, 73.6, 45.7, 39.1, 21.7, 15.9. **HRMS**-ESI (m/z): $[M + H]^+$ calculated for $C_{18}H_{18}ClN_2O_4^+$, 361.0945; found, 361.0951.

**(±) 2,2,7,8-tetramethyl-4,4a,8,9b-tetrahydro-2H-pyrano[2',3':4,5]furo[3,2-c]pyridine-3,9-dione (13ca)**

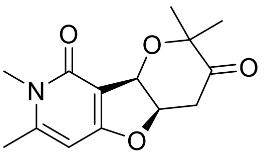

According to GP6, **8c** (27.6 mg, 0.15 mmol) was reacted with **5b** (20.9 mg, 0.15 mmol). Purification by MPLC (cyclohexane/EtOAc 1:0 to 0:1) afforded the product (6.3 mg, 16%). **$^1$H-NMR** (700 MHz, $CD_2Cl_2$): δ 5.99 (s, 1H), 5.37 (d, *J* = 6.6 Hz, 1H), 5.01 (dt, *J* = 7.7, 6.6 Hz, 1H), 3.51 (s, 3H), 3.06 (dd, *J* = 13.8, 7.7 Hz, 1H), 2.98 (dd, *J* = 13.8, 6.9 Hz, 1H), 2.38 (s, 3H), 1.38 (s, 3H), 1.24 (s, 3H). **$^{13}$C-NMR** (176 MHz, $CD_2Cl_2$): δ 212.4, 169.3, 162.1, 152.2, 106.4, 96.2, 84.3, 81.7, 71.6, 40.0,

31.4, 25.6, 22.1, 21.5. **HRMS**-ESI (m/z): $[M + H]^+$ calculated for $C_{14}H_{18}NO_4^+$, 264.1230; found, 264.1230.

**(±) 8-benzyl-2,2,7-trimethyl-4,4a,8,9b-tetrahydro-2H-pyrano[2',3':4,5]furo[3,2-c]pyridine-3,9-dione (13cb)**

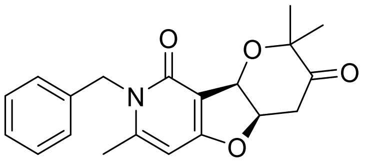

According to GP6, **8c** (50.0 mg, 0.32 mmol) was reacted with **5c** (58.4 mg, 0.32 mmol) employing allylpalladium(II) chloride dimer (2.5 mol%) and Xantphos (7.5 mol%) as catalyst. Purification by MPLC (cyclohexane/EtOAc 1:0 to 0:1) afforded the product (48.0 mg 52%). **$^1$H NMR** (500 MHz, $CDCl_3$): δ 7.31 (m, 2H), 7.16 (d, *J* = 6.7 Hz, 2H), 5.96 (s, 1H), 5.55 (d, *J* = 15.7 Hz, 1H), 5.44 (d, *J* = 6.7 Hz, 1H), 5.14 (d, *J* = 15.7 Hz, 1H), 5.03 (dt, *J* = 7.9, 6.7 Hz, 1H), 3.14 (dd, *J* = 13.7, 7.3 Hz, 1H), 3.02 (dd, *J* = 13.7, 7.3 Hz, 1H), 2.32 (s, 1H), 1.43 (s, 3H), 1.31 (s, 3H). **$^{13}$C NMR** (126 MHz, $CDCl_3$): δ 212.7, 169.1, 161.9, 151.6, 136.2, 128.9, 127.5, 126.6, 106.4, 96.4, 83.9, 81.5, 71.3, 47.1, 39.7, 25.5, 21.6, 21.2. **HRMS**-ESI (m/z): $[M + H]^+$ calculated for $C_{20}H_{22}O_4N^+$,340.15433; found, 340.15470.

**(±) 8-(4-fluorobenzyl)-2,2,7-trimethyl-4,4a,8,9b-tetrahydro-2H-pyrano[2',3':4,5]furo[3,2-c]pyridine-3,9-dione (13cc)**

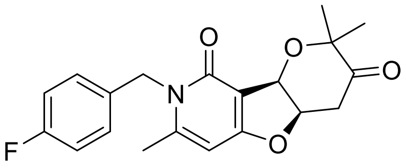

According to GP6, **8c** (50.0 mg, 0.27 mmol) was reacted with **5k** (63.3 mg, 0.27 mmol). Purification by MPLC (cyclohexane/EtOAc 1:0 to 0:1) afforded the product (23.7 mg, 24%). **$^1$H-NMR** (600 MHz, $CDCl_3$): δ δ 7.18-7.15 (m, 2H), 7.01 (dd, *J* = 10.6, 6.0 Hz, 2H), 5.97 (s, 1H), 5.48 (d, *J* = 16.0 Hz, 1H), 5.44 (d, *J* = 6.3 Hz, 1H), 5.14-5.10 (m, 1H), 5.05-5.00 (m, 1H), 3.13 (dd, *J* = 13.7, 7.2 Hz, 1H), 3.02 (dd, *J* = 13.7, 6.0 Hz, 1H), 2.33 (s, 3H), 1.43 (s, 3H), 1.31 (s, 3H). **$^{13}$C-NMR** (151 MHz, $CDCl_3$): δ 212.6, 169.3, 163.1, 161.5, 162.0, 151.5, 132.1, 128.6, 115.9, 106.6, 96.8, 84.2, 81.7, 71.4, 46.8, 39.8, 25.6, 21.8, 21.4. **HRMS**-ESI (m/z): $[M + H]^+$ calculated for $C_{20}H_{21}NO_4F^+$, 358.1449; found, 358.1466.

**(±) 8-(2-chlorobenzyl)-2,2,7-trimethyl-4,4a,8,9b-tetrahydro-2H-pyrano[2',3':4,5]furo[3,2-c]pyridine-3,9-dione (13cd)**

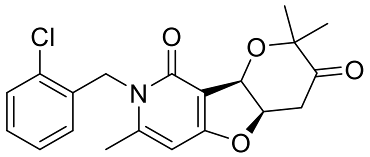

According to GP6, **8c** (50.0 mg, 0.27 mmol) was reacted with **5l** (67.8 mg, 0.27 mmol). Purification by MPLC (cyclohexane/EtOAc 1:0 to 0:1) afforded the product (5.8 mg, 6%). **$^{1}$H-NMR** (600 MHz, $CD_2Cl_2$): δ 7.43 (dd, *J* = 7.7, 1.4 Hz, 1H), 7.24 (td, *J* = 7.7, 1.7 Hz, 1H), 7.20 (td, *J* = 7.7, 1.4 Hz, 1H), 6.75 (dd, *J* = 7.7, 1.7 Hz, 1H), 5.99 (s, 1H), 5.44 (d, *J* = 16.9 Hz, 1H), 5.41 (d, *J* = 6.6 Hz, 1H), 5.29 (d, *J* = 16.9 Hz, 1H), 5.11-5.04 (m, 1H), 3.10 (dd, *J* = 13.8, 7.5 Hz, 1H), 3.02 (dd, *J* = 13.8, 6.7 Hz, 1H), 2.23 (s, 3H), 1.40 (s, 3H), 1.28 (s, 3H). **$^{13}$C-NMR** (151 MHz, $CD_2Cl_2$): δ 212.5, 169.5, 161.8, 152.0, 134.3, 132.7, 130.0, 129.0, 127.7, 126.6, 106.5, 96.4, 84.4, 81.7, 71.8, 44.9, 40.0, 25.5, 22.0. 21.4. **HRMS**-ESI (m/z): $[M + H]^+$ calculated for $C_{20}H_{21}NO_4Cl^+$, 374.1154; found, 374.1167.

**(±) 8-(4-methoxybenzyl)-2,2,7-trimethyl-4,4a,8,9b-tetrahydro-2H-pyrano[2',3':4,5]furo[3,2-c]pyridine-3,9-dione (13ce)**

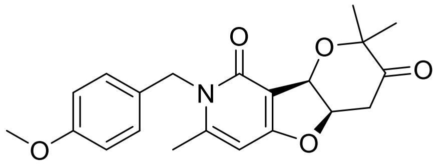

According to GP6, **8c** (27.7 mg, 0.15 mmol) was reacted with **5d** (55.2 mg, 0.15 mmol). Purification by MPLC (cyclohexane/EtOAc 1:0 to 0:1) afforded the product (10.1 mg, 18%). **$^{1}$H-NMR** (600 MHz, $CDCl_3$): δ 7.10 (d, *J* = 8.8 Hz, 2H), 6.81 (d, *J* = 8.8 Hz, 2H), 5.88 (s, 1H), 5.42 (d, *J* = 15.4 Hz, 1H), 5.40 (d, *J* = 6.6 Hz, 1H), 5.03 (d, *J* = 15.4 Hz, 1H), 4.98 (dt, *J* = 7.9, 6.6 Hz, 1H), 3.74 (s, 3H), 3.10 (dd, *J* = 13.6, 7.9 Hz, 1H), 2.98 (dd, *J* = 13.6, 6.9 Hz, 1H), 2.29 (s, 3H), 1.40 (s, 3H), 1.28 (s, 3H). **$^{13}$C-NMR** (151 MHz, $CDCl_3$): δ 212.9, 169.0, 161.9, 159.1, 151.6, 128.6, 128.2, 114.3, 106.4, 96.1, 84.0, 81.6, 71.6, 55.4, 46.6, 39.8, 21.7, 21.5. **HRMS**-ESI (m/z): $[M + H]^+$ calculated for $C_{21}H_{24}NO_5^+$, 370.1649; found, 370.1637.

**(±) 2,2,7-trimethyl-8-(pyridin-4-ylmethyl)-4,4a,8,9b-tetrahydro-2H-pyrano[2',3':4,5]furo[3,2-c]pyridine-3,9-dione (13cf)**

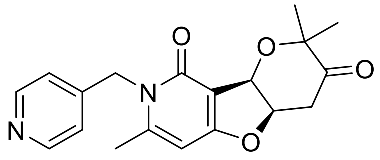

According to GP6, **8cc** (27.7 mg, 0.15 mmol) was reacted with **5i** (32.2 mg, 0.15 mmol). Purification by MPLC (cyclohexane/EtOAc 1:0 to 0:1 then EtOAc/MeOH 1:0 to 9:1) afforded the product (11.5 mg, 23%). **$^{1}$H-NMR** (700 MHz, $CD_2Cl_2$): δ 8.53-8.51 (m, 2H), 7.05-7.04 (m, 2H), 5.93 (s, 1H), 5.38 (d, *J* = 6.7 Hz, 1H), 5.37 (d, *J* = 16.0 Hz, 1H), 5.16 (d, *J* = 16.0

Hz, 1H), 5.05 (dt, $J$ = 7.5, 6.7 Hz, 1H), 3.09 (dd, $J$ = 13.8, 7.5 Hz, 1H), 3.01 (dd, $J$ = 13.8, 6.7 Hz, 1H), 2.24 (s, 3H), 1.40 (s, 3H), 1.28 (s, 3H). **$^{13}$C-NMR** (176 MHz, $CD_2Cl_2$): δ 212.6, 169.2, 161.3, 151.5, 150.5, 146.5, 121.6, 106.4, 95.8, 84.3, 81.6, 72.0, 46.1, 40.0, 25.4, 22.1, 21.7. **HRMS**-ESI (m/z): $[M + H]^+$ calculated for $C_{19}H_{21}N_2O_4^+$, 341.1496; found, 341.1493.

**(±) 8-((2-chloropyridin-4-yl)methyl)-2,2,7-trimethyl-4,4a,8,9b-tetrahydro-2H-pyrano[2',3':4,5]furo[3,2-c]pyridine-3,9-dione (13cg)**

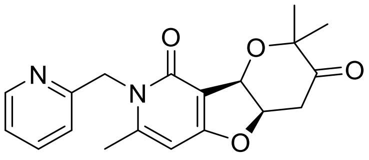


According to GP6, **8cc** (50.0 mg, 0.32 mmol) was reacted with **5m** (58.7 mg, 0.32 mmol) employing allylpalladium(II) chloride dimer (2.5 mol%) and Xantphos (7.5 mol%) as catalyst. Purification by MPLC (cyclohexane/EtOAc 1:0 to 0:1) afforded the product (17.8 mg, 19%). $^1$H NMR (700 MHz, $CDCl_3$): δ 8.70 (d, $J$ = 4.5 Hz, 1H), 7.99 (td, $J$ = 7.9, 1.7 Hz, 1H), 7.52 (m, 1H), 7.49 (d, $J$ = 7.9 Hz, 1H), 6.04 (s, 1H), 5.69 (d, $J$ = 16.5 Hz, 1H), 5.55 (d, $J$ = 16.5 Hz, 1H), 5.40 (d, $J$ = 6.5 Hz, 1H), 5.03 (m, 1H), 3.13 (dd, $J$ = 13.7, 7.5 Hz, 1H), 3.02 (dd, $J$ = 13.7, 7.5 Hz, 1H), 2.41 (s, 3H), 1.42 (s, 3H), 1.30 (s, 3H). **$^{13}$C NMR** (176 MHz, $CDCl_3$): δ 212.3, 169.8, 161.8, 154.4, 151.7, 145.6, 141.6, 124.3, 123.9, 106.5, 97.3, 84.2, 81.7, 71.2, 46.8, 39.7, 25.6, 21.9, 21.2. **HRMS**-ESI (m/z): $[M + H]^+$ calculated for $C_{19}H_{21}O_4N_2^+$,341.14958; found, 341.14984

**(±) 8-((2-chloropyridin-4-yl)methyl)-2,2,7-trimethyl-4,4a,8,9b-tetrahydro-2H-pyrano[2',3':4,5]furo[3,2-c]pyridine-3,9-dione (13ch)**

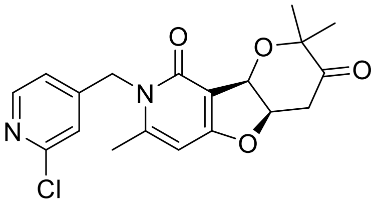


According to GP6, **8c** (27.7 mg, 0.15 mmol) was reacted with **5r** (37.3 mg, 0.15 mmol). Purification by MPLC (cyclohexane/EtOAc 1:0 to 0:1) afforded the product (35.2 mg, 63%). **$^1$H-NMR** (700 MHz, $CD_2Cl_2$): δ 8.31 (dd, $J$ = 5.1, 0.8 Hz, 1H), 7.06 (dd, $J$ = 1.6, 0.8 Hz, 1H), 7.03-7.02 (m, 1H), 5.94 (s, 1H), 5.38 (d, $J$ = 6.7 Hz, 1H), 5.37 (d, $J$ = 15.2 Hz, 1H), 5.13 (d, $J$ = 15.2 Hz, 1H), 5.07 (dt, $J$ = 7.5, 6.7 Hz, 1H), 3.09 (dd, $J$ = 13.8, 7.5 Hz, 1H), 3.02 (dd, $J$ = 13.8, 6.7 Hz, 1H), 2.24 (s, 3H), 1.39 (s, 3H), 1.28 (s, 3H). **$^{13}$C-NMR** (176 MHz, $CD_2Cl_2$): δ 212.4, 169.3, 161.2, 152.5, 151.2, 150.4, 150.0, 122.0, 120.7, 106.4, 96.1, 84.4, 81.6, 71.9, 45.8, 39.9, 25.5, 22.2, 21.7. **HRMS**-ESI (m/z): $[M + H]^+$ calculated for $C_{19}H_{20}ClN_2O_4^+$, 375.1106; found, 375.1105.

**(±) 2,2,7-trimethyl-8-((3-methylpyridin-4-yl)methyl)-4,4a,8,9b-tetrahydro-2H-pyrano[2',3':4,5]furo[3,2-c]pyridine-3,9-dione (13ci)**

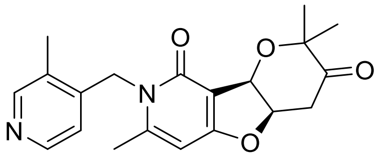

According to GP6, **8c** (50.0 mg, 0.32 mmol) was reacted with **5q** (35.4 mg, 0.32 mmol) employing Allylpalladium(II) chloride dimer (2.5 mol%) and Xantphos (7.5 mol%) as catalyst. Purification by MPLC (cyclohexane/EtOAc 1:0 to 0:1) afforded the product (4.7 mg, 5%). **$^1$H NMR** (700 MHz, $CDCl_3$): δ 8.71 (s, 1H), 8.57 (d, *J* = 5.7 Hz, 1H), 7.10 (d, *J* = 5.7 Hz, 1H), 6.11 (s, 1H), 5.48 (d, *J* = 17.6 Hz, 1H), 5.38 (d, *J* = 6.7 Hz, 1H), 5.23 (d, *J* = 17.6 Hz, 1H), 5.06 (dt, *J* = 8.1, 6.7 Hz, 1H), 3.15 (dd, *J* = 13.7, 7.6 Hz, 1H), 3.05 (dd, *J* = 13.7, 7.6 Hz, 1H), 2.60 (s, 3H), 2.30 (s, 3H), 1.41 (s, 3H), 1.31 (s, 3H). **$^{13}$C NMR** (176 MHz, $CDCl_3$): δ 212.1, 169.8, 161.2, 154.0, 150.0, 142.8, 140.9, 134.9, 121.8, 106.9, 97.5, 84.4, 81.8, 77.3, 77.2, 77.0, 71.1, 44.8, 39.7, 25.7, 21.5, 21.2, 16.6. **HRMS**-ESI (m/z): $[M + H]^+$ calculated for $C_{20}H_{23}O_4N_2^+$,355.16523; found, 355.16537.

**(±) 5,8,8-trimethyl-5,6b,10,10a-tetrahydro-6H-pyrano[2',3':4,5]furo[3,2-c]quinoline-6,9(8H)-dione (13cj)**

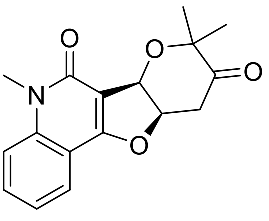

According to GP6, **8c** (27.7 mg, 0.15 mmol) was reacted with 4-hydroxy-1-methylquinolin-2(1H)-one (26.3 mg, 0.15 mmol). Purification by MPLC (cyclohexane/EtOAc 1:0 to 0:1) and repurification by prep. HPLC afforded the product (2.1 mg, 5%). **$^1$H-NMR** (500 MHz, $CDCl_3$): δ 7.82 (dd, *J* = 8.0, 1.7 Hz, 1H), 7.68 (ddd, *J* = 8.7, 7.2, 1.7 Hz, 1H), 7.43 (d, *J* = 8.7 Hz, 1H), 7.30 (ddd, *J* = 8.0, 7.2, 0.9 Hz, 1H), 5.54 (d, *J* = 6.7 Hz, 1H), 5.18 (dt, *J* = 8.0, 6.7 Hz, 1H), 3.74 (s, 3H), 3.24 (dd, *J* = 13.7, 8.0 Hz, 1H), 3.12 (dd, *J* = 13.7, 7.0 Hz, 1H), 1.48 (s, 3H), 1.32 (s, 3H). **$^{13}$C-NMR** (126 MHz, $CDCl_3$): δ 212.5, 161.1, 158.4, 141.7, 132.8, 124.0, 122.4, 115.1, 112.2, 107.7, 84.3, 81.9, 72.0, 39.8, 29.5, 25.5. 21.3. **HRMS**-ESI (m/z): $[M + H]^+$ calculated for $C_{17}H_{18}NO_4^+$, 300.1230; found, 300.1234.

**(±) 2,2,7-trimethyl-4a,9b-dihydro-2H,9H-furo[3,2-b:4,5-c']dipyran-3,9(4H)-dione (13ck)**[112]

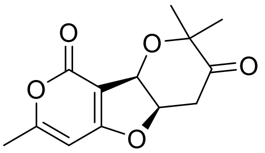

According to GP6, **8c** (27.6 mg, 0.15 mmol) was reacted with 4-hydroxy-6-methyl-2H-pyran-2-one (18.9 mg, 0.15 mmol). Purification by MPLC (cyclohexane/EtOAc 1:0 to 0:1) afforded the product (1.4 mg, 4%). **$^1$H-NMR** (700 MHz, $CD_2Cl_2$): δ 5.98 (s, 1H), 5.28 (d, *J* = 6.7 Hz, 1H), 5.07 (dt, *J* = 7.8,

6.7 Hz, 1H), 3.08 (dd, *J* = 13.8, 7.8 Hz, 1H), 3.01 (dd, *J* = 13.8, 6.8 Hz, 1H), 2.27 (s, 3H), 1.39 (s, 3H), 1.29 (s, 3H). ). **HRMS**-ESI (m/z): $[M + H]^+$ calculated for $C_{13}H_{15}O_5^+$, 251.0914; found, 251.0915.

**(±) *Tert*-butyl-7',8'-dimethyl-3',9'-dioxo-3',4',4a',8',9',9b'-hexahydrospiro[piperidine-4,2'-pyrano[2',3':4,5]furo[3,2-c]pyridine]-1-carboxylate (13da)**

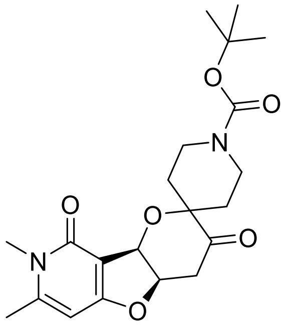

According to GP6, **8d** (50.0 mg, 0.15 mmol) was reacted with **5b** (21.4 mg, 0.15 mmol). Purification by MPLC (cyclohexane/EtOAc 1:0 to 0:1) afforded the product (56.1 mg, 90%). **$^1$H-NMR** (700 MHz, $CD_2Cl_2$): δ 5.85 (s, 1H), 5.35 (d, *J* = 6.7 Hz, 1H), 5.03 (q, *J* = 6.7 Hz, 1H), 3.98-3.73 (m 2H), 3.45 (s, 3H), 3.26-2.98 (m, 4H), 2.34 (s, 3H), 1.88 (d, *J* = 14.1 Hz, 1H), 1.72 (ddd, *J* = 13.8, 12.1, 4.5 Hz, 1H), 1.65-1.59 (m, 1H), 1.53-1.48 (m, 1H), 1.47 (m, 9H). **$^{13}$C-NMR** (176 MHz, $CD_2Cl_2$): δ 211.5; 168.6, 161.4, 154.8, 152.1, 105.7, 94.6, 83.9, 81.0, 79.5, 71.1, 40.1, 39.5, 38.6, 33.1, 30.7, 29.6, 28.5. **HRMS**-ESI (m/z): $[M + H]^+$ calculated for $C_{21}H_{29}N_2O_6^+$, 405.2020; found, 405.2015.

**(±) 7',8'-dimethyl-3',9'-dioxo-3',4',4a',8',9',9b'-hexahydrospiro[piperidine-4,2'-pyrano[2',3':4,5]furo[3,2-c]pyridin]-1-ium triflate (13ea)**

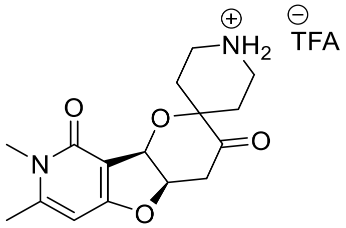

**13da** (35.0 mg, 0.09 mmol) was dissolved in DCM (1 mL) and cooled to 0 °C. A mixture of TMSOTf (0.16 mL, 10 equiv) and 2,6-lutidine (0.15 mL, 15 equiv) in DCM (0.1 mL) was added slowly and the mixture was stirred at 0 °C for 1 hour and at room temperature for 30 min. The reaction was quenched by slow addition of saturated $Na_2CO_3$ solution at 0 °C and dilution with EtOAc (10 mL). The layers were separated and the aqueous phase was extracted with EtOAc (3 x 10 mL). The combined organic layers were washed with brine (20 mL), dried over $MgSO_4$ and concentrated in vacuo. Purification by prep. HPLC afforded the product (8.2 mg, 31%). **$^1$H-NMR** (500 MHz, DMSO): δ 8.56 (d, *J* = 11.2 Hz, 1H), 8.42 (d, *J* = 13.7 Hz, 1H), 6.07 (s, 1H), 5.33 (d, *J* = 6.5 Hz, 1H), 5.18 (q, *J* = 6.5 Hz, 1H), 3.34 (s, 3H), 3.21-2.91 (m, 6H), 2.36 (s, 3H), 1.87-1.57 (m, 4H). **$^{13}$C-NMR** (126 MHz, DMSO): δ 210.9, 168.2, 160.8, 153.2, 104.7, 94.1, 83.6, 77.7, 71.6, 40.5, 39.2, 30.4, 29.6, 26.5, 21.6. **HRMS**-ESI (m/z): $[M + H]^+$ calculated for $C_{16}H_{21}N_2O_4^+$, 305.1496; found, 305.1487.

**(±) *Tert*-butyl 8'-cyclobutyl-7'-methyl-3',9'-dioxo-3',4',4a',8',9',9b'-hexahydrospiro[piperidine-4,2'-pyrano[2',3':4,5]furo[3,2-c]pyridine]-1-carboxylate (13db)**

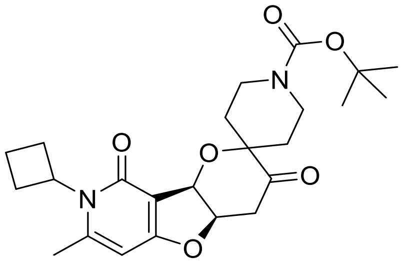

According to GP6, **8d** (48.8 mg, 0.15 mmol) was reacted with **5e** (26.9 mg, 0.15 mmol). Purification by MPLC (cyclohexane/EtOAc 1:0 to 0:1) afforded the product (47.7 mg, 72%). **$^1$H-NMR** (500 MHz, $CD_2Cl_2$): δ 5.76 (s, 1H), 5.36 (d, *J* = 6.8 Hz, 1H), 5.03 (q, *J* = 6.8 Hz, 1H), 4.72 (p, *J* = 8.9 Hz, 1H), 3.99-3.71 (m, 2H), 3.29-2.99 (m, 6H), 2.33 (s, 3H), 2.23 (ddd, *J* = 11.4, 7.9, 2.9 Hz, 2H), 1.99-1.47 (m, 6H), 1.42 (s, 9H). **$^{13}$C-NMR** (126 MHz, $CD_2Cl_2$): δ 211.6, 168.2, 162.9, 154.8, 151.7, 107.2, 95.3, 83.9, 81.0, 79.5, 72.1, 53.3, 40.2, 38.6, 38.5, 32.9, 30.1, 28.5, 28.1, 22.7, 15.0. **HRMS**-ESI (m/z): [M + H]$^+$ calculated for $C_{24}H_{33}N_2O_6^+$, 445.2333; found, 445.2328.

**(±) 8'-benzyl-7'-methyl-3',9'-dioxo-3',4',4a',8',9',9b'-hexahydrospiro[piperidine-4,2'-pyrano[2',3':4,5]furo[3,2-c]pyridin]-1-ium triflate (13ec)**

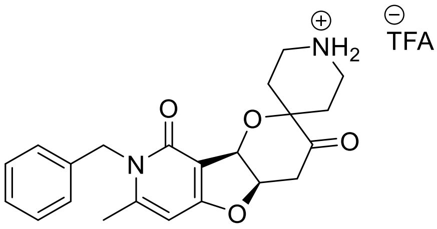


According to GP6, **8d** (39.0 mg, 0.12 mmol) was reacted with **5c** (25.8 mg, 0.12 mmol). Purification by MPLC (cyclohexane/EtOAc 1:0 to 0:1) afforded the product which was directly dissolved in DCM (1 mL) and cooled to 0 °C. A mixture of TMSOTf (0.21 mL, 10 equiv) and 2,6-lutidine (0.21 mL, 15 equiv) in DCM (0.1 mL) was added slowly and the mixture was stirred at 0 °C for 30 min. The reaction was quenched by slow addition of saturated $Na_2CO_3$ solution at 0 °C and dilution with EtOAc (10 mL). The layers were separated and the aqueous phase was extracted with EtOAc (3 x 10 mL). The combined organic layers were washed with brine (20 mL), dried over $MgSO_4$ and concentrated in vacuo. Purification by prep. HPLC afforded the product (7.3 mg, 17% over two steps). **$^1$H-NMR** (700 MHz, DMSO): δ 8.56 (s, 1H), 8.45 (s, 1H), 7.37-7.32 (m, 2H), 7.27 (t, *J* = 7.4 Hz, 1H), 7.10 (d, *J* = 7.7 Hz, 2H), 6.13 (s, 1H), 5.42 (d, *J* = 6.7 Hz, 1H), 5.32 (d, *J* = 16.0 Hz, 1H), 5.28 (q, *J* = 6.4 Hz, 1H), 5.22 (d, *J* = 13.8 Hz, 1H), 3.29-3.23 (m, 1H), 3.18-3.14 (m, 1H), 3.10-3.00 (m, 3H), 2.97-2.93 (m, 1H), 2.28 (s, 3H), 1.86-1.78 (m, 2H), 1.73-1.68 (m, 1H), 1.66-1.61 (m, 1H). **$^{13}$C-NMR** (176 MHz, DMSO): δ 210.5,168.1, 160.1, 152.4, 137.7, 129.2, 127.6, 126.5, 104.9, 94.9, 83.9, 77.5, 71.4, 45.6, 38.8,

28.4, 26.7, 24.4, 20.7. **HRMS**-ESI (m/z): $[M + H]^+$ calculated for $C_{22}H_{25}N_2O_4^+$, 381.1809; found, 381.1810.

**(±) *Tert*-butyl-8'-(4-fluorobenzyl)-7'-methyl-3',9'-dioxo-3',4',4a',8',9',9b'-hexahydrospiro[piperidine-4,2'-pyrano[2',3':4,5]furo[3,2-c]pyridine]-1-carboxylate (13dd)**

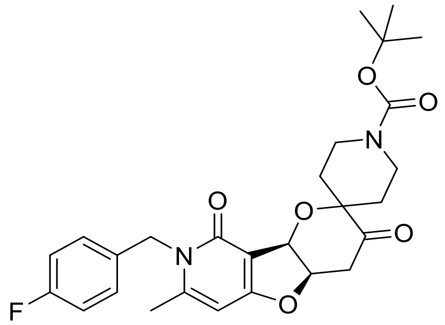

According to GP6, **8d** (48.8 mg, 0.15 mmol) was reacted with **5k** (35.0 mg, 0.15 mmol). Purification by MPLC (cyclohexane/EtOAc 1:0 to 0:1) afforded the product (55.2 mg, 74%). **$^1$H-NMR** (700 MHz, $CD_2Cl_2$): δ 7.15 (dd, *J* = 8.6, 5.3 Hz, 2H), 7.03 (t, *J* = 8.6 Hz, 2H), 5.88 (s, 1H), 5.47-5.38 (m, 1H), 5.27-5.07 (m, 3H), 3.99-3.70 (m, 2H), 3.25-3.05 (m, 4H), 2.28 (s, 3H), 1.87 (dq, *J* = 13.9, 2.9 Hz, 1H), 1.76-1.63 (m, 2H), 1.55-1.49 (m, 1H), 1.42 (s, 9H). **$^{13}$C-NMR** (176 MHz, $CD_2Cl_2$): δ 211.3, 168.9, 163.2, 161.8, 161.5, 154.8, 152.1, 133.2, 128.6, 116.0, 106.2, 95.5, 84.3, 81.2, 79.6, 72.1, 46.3, 40.0, 39.6, 38.6, 32.9, 30.1, 28.5, 21.8. **HRMS**-ESI (m/z): $[M + H]^+$ calculated for $C_{27}H_{32}FN_2O_6^+$, 499.2239; found, 499.2230.

**(±) 8'-(4-fluorobenzyl)-7'-methyl-3',9'-dioxo-3',4',4a',8',9',9b'-hexahydrospiro[piperidine-4,2'-pyrano[2',3':4,5]furo[3,2-c]pyridin]-1-ium triflate (13ed)**

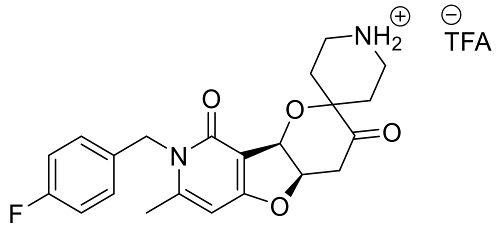


According to GP6, **8d** (97.6 mg, 0.30 mmol) was reacted with **5k** (70.0 mg, 0.30 mmol). Purification by MPLC (cyclohexane/EtOAc 1:0 to 0:1) afforded the product which was directly dissolved in DCM (1 mL) and cooled to 0 °C. A mixture of TMSOTf (0.13 mL, 10 equiv) and 2,6-lutidine (0.13 mL, 15 equiv) in DCM (0.1 mL) was added slowly and the mixture was stirred at 0 °C for 1 hour and at room temperature overnight. The reaction was quenched by slow addition of saturated $Na_2CO_3$ solution at 0 °C and dilution with EtOAc (10 mL). The layers were separated and the aqueous phase was extracted with EtOAc (3 x 10 mL). The combined organic layers were washed with brine (20 mL), dried over $MgSO_4$ and concentrated in vacuo. Purification by prep. HPLC afforded the product (3.8 mg, 3% over two steps). **$^1$H-NMR** (700 MHz, DMSO): δ 8.51 (s, 1H), 8.43 (s, 1H), 7.24-7.13 (m, 4H), 6.13 (s, 1H), 5.41 (d, *J* = 6.7 Hz, 1H), 5.31-5.24 (m, 2H), 5.20 (d, , *J* = 12.7 Hz, 1H), 3.28-3.24 (m, 1H), 3.16 (dd, *J* = 14.4, 6.4 Hz, 1H), 3.11-3.07 (m, 1H), 3.03-3.00 (m, 1H), 2.96-2.92 (m, 1H), 3.05

(dd, $J$ = 14.4, 6.2 Hz, 1H), 2.30 (s, 3H), 2.23-2.20 (m, 1H), 1.86-1.76 (m, 2H), 1.72-1.68 (m, 1H). **$^{13}$C-NMR** (176 MHz, DMSO): δ 210.3, 168.1, 161.9, 160.6, 160.4, 152.4, 133.4, 128.2, 115.4, 104.8, 94.6, 83.5, 77.4, 71.2, 45.0, 40.0, 38.8, 28.9, 26.7, 20.7. **HRMS**-ESI (m/z): [M + H]$^+$ calculated for $C_{22}H_{24}FN_2O_4^+$, 399.1715; found, 399.1713.

**(±) 8'-(4-methoxybenzyl)-7'-methyl-3',9'-dioxo-3',4',4a',8',9',9b'-hexahydrospiro[piperidine-4,2'-pyrano[2',3':4,5]furo[3,2-c]pyridin]-1-ium triflate (13ee)**

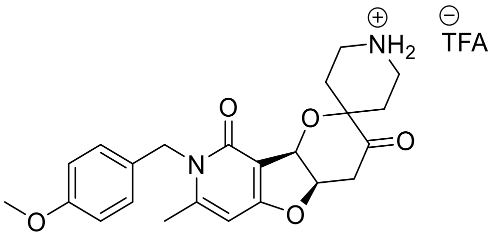


According to GP6, **8d** (97.6 mg, 0.30 mmol) was reacted with **5d** (76.6 mg, 0.30 mmol). Purification by MPLC (cyclohexane/EtOAc 1:0 to 0:1) afforded the product which was directly dissolved in DCM (1 mL) and cooled to 0 °C. A mixture of TMSOTf (0.20 mL, 10 equiv) and 2,6-lutidine (0.20 mL, 15 equiv) in DCM (0.1 mL) was added slowly and the mixture was stirred at 0 °C for 1 hour and at room temperature for 30 min. The reaction was quenched by slow addition of saturated $Na_2CO_3$ solution at 0 °C and dilution with EtOAc (10 mL). The layers were separated and the aqueous phase was extracted with EtOAc (3 x 10 mL). The combined organic layers were washed with brine (20 mL), dried over $MgSO_4$ and concentrated in vacuo. Purification by prep. HPLC afforded the product (5.2 mg, 4% over two steps). **$^1$H-NMR** (700 MHz, DMSO): δ 8.54 (s, 1H), 8.43 (s, 1H), 7.06 (d, $J$ = 8.5 Hz, 2H), 6.90 (d, $J$ = 8.5 Hz, 2H), 6.10 (s, 1H), 5.41 (d, $J$ = 6.5 Hz, 1H), 5.27 (q, $J$ = 6.5 Hz, 1H), 5.24-5.11 (m, 2H), 3.72 (s, 3H), 3.29-3.24 (m, 1H), 3.15 (dd, $J$ = 14.4, 6.5 Hz, 1H), 3.11-3.07 (m, 1H), 3.06-3.01 (m, 2H), 2.99-2.93 (m, 1H), 2.29 (s, 3H), 2.24-2.20 (m, 1H), 1.86-1.77 (m, 2H), 1.72-1.66 (m, 1H). **$^{13}$C-NMR** (176 MHz, DMSO): δ 210.4, 168.0, 160.5, 1588.3, 152.5, 129.1, 127.6, 114.1, 104.8, 94.5, 83.4, 77.4, 71.3, 55.1, 45.1, 40.0, 38.8, 28.9, 26.7, 20.7. **HRMS**-ESI (m/z): [M + H]$^+$ calculated for $C_{23}H_{27}N_2O_5^+$, 411.1915; found, 411.1908.

**(±) 7'-methyl-3',9'-dioxo-8'-(thiophen-2-ylmethyl)-3',4',4a',8',9',9b'-hexahydrospiro[piperidine-4,2'-pyrano[2',3':4,5]furo[3,2-c]pyridin]-1-ium triflate (13ef)**

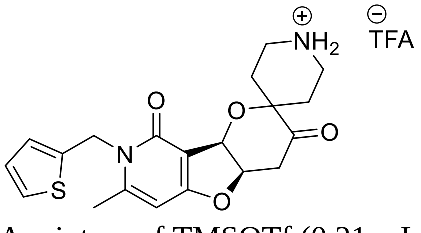


According to GP6, **8d** (48.8 mg, 0.15 mmol) was reacted with **5j** (33.2 mg, 0.15 mmol). Purification by MPLC (cyclohexane/EtOAc 1:0 to 0:1) afforded the product which was directly dissolved in DCM (1 mL) and cooled to 0 °C. A mixture of TMSOTf (0.21 mL, 10 equiv) and 2,6-lutidine (0.20 mL, 15 equiv) in DCM (0.1 mL) was added slowly and the mixture was stirred at 0 °C for 30 min. The reaction was

quenched by slow addition of saturated $Na_2CO_3$ solution at 0 °C and dilution with EtOAc (10 mL). The layers were separated and the aqueous phase was extracted with EtOAc (3 x 10 mL). The combined organic layers were washed with brine (20 mL), dried over $MgSO_4$ and concentrated in vacuo. Purification by prep. HPLC afforded the product (3.6 mg, 6% over two steps). **$^1$H-NMR** (700 MHz, DMSO): δ 8.50 (s, 1H), 8.34 (s, 1H), 7.42 (d, *J* = 5.1 Hz, 1H), 7.07 (d, *J* = 3.7 Hz, 1H), 6.98 (dd, *J* = 5.1, 3.7 Hz, 1H), 6.09 (s, 1H), 5.42 (d, *J* = 6.6 Hz, 1H), 5.38 (d, *J* = 15.5 Hz, 1H), 5.33 (d, *J* = 15.5 Hz, 1H), 5.26 (q, *J* = 6.6 Hz, 1H), 3.17-2.99 (m, 6H), 2.44 (s, 3H), 2.25-2.21 (m, 1H), 1.85-1.75 (m, 2H), 1.69-1.65 (m, 1H). **$^{13}$C-NMR** (176 MHz, DMSO): δ 210.5, 168.1, 160.1, 152.0, 139.5, 126.7, 126.6, 126.1, 104.8, 94.6, 83.6, 77.4, 71.3, 41.5, 38.9, 38.8, 28.8, 26.8, 20.7. **HRMS**-ESI (m/z): $[M + H]^+$ calculated for $C_{20}H_{23}N_2O_4S^+$, 387.1373; found, 387.1374.

**(±) *Tert*-butyl-7'-methyl-3',9'-dioxo-8'-(pyridin-4-ylmethyl)-3',4',4a',8',9',9b'-hexahydrospiro[piperidine-4,2'-pyrano[2',3':4,5]furo[3,2-c]pyridine]-1-carboxylate (13dg)**

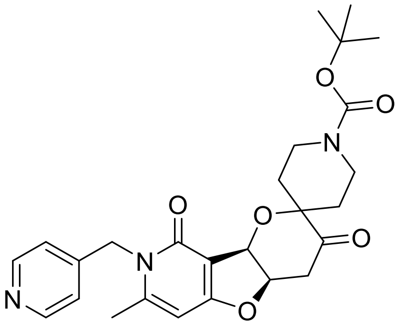

According to GP6, **8d** (48.8 mg, 0.15 mmol) was reacted with **5i** (32.4 mg, 0.15 mmol). Purification by MPLC (cyclohexane/EtOAc 1:0 to 0:1 then EtOAc/MeOH 1:0 to 9:1) afforded the product (43.9 mg, 61%). **$^1$H-NMR** (700 MHz, $CDCl_3$): δ 8.53 (d, *J* = 6.1 Hz, 2H), 7.04 (d, *J* = 6.1 Hz, 2H), 5.93 (s, 1H), 5.53-5.31 (m, 2H), 5.22-4.99 (m, 2H), 4.01-3.71 (m, 2H), 3.27-3.01 (m, 2H), 2.26 (s, 3H), 1.91-1.51 (m, 4H), 1.41 (s, 9H). **$^{13}$C-NMR** (176 MHz, $CDCl_3$): δ 211.2, 173.7, 171.2, 161.2, 151.1, 150.2, 146.0, 121.3, 106.0, 96.0, 83.9, 79.6, 71.2, 60.5, 45.9, 39.8, 39.2, 38.2, 33.2, 33.0, 28.5, 21.6. **HRMS**-ESI (m/z): $[M + H]^+$ calculated for $C_{26}H_{32}N_3O_6^+$, 482.2286; found, 482.2279.

**(±) 7'-methyl-3',9'-dioxo-8'-(pyridin-2-ylmethyl)-3',4',4a',8',9',9b'-hexahydrospiro[piperidine-4,2'-pyrano[2',3':4,5]furo[3,2-c]pyridin]-1-ium triflate (13eh)**

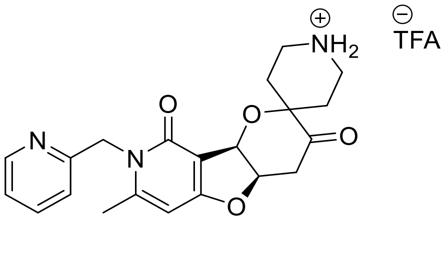


According to GP6, **8d** (48.8 mg, 0.15 mmol) was reacted with **5m** (32.4 mg, 0.15 mmol). Purification by MPLC (cyclohexane/EtOAc 1:0 to 0:1) afforded the product which was directly dissolved in DCM (1 mL) and cooled to 0 °C. A mixture of TMSOTf (0.12 mL, 10 equiv) and 2,6-lutidine (0.12 mL, 15 equiv) in DCM (0.1 mL) was added slowly and the mixture was stirred

at 0 °C for 30 min. The reaction was quenched by slow addition of saturated $Na_2CO_3$ solution at 0 °C and dilution with EtOAc (10 mL). The layers were separated and the aqueous phase was extracted with EtOAc (3 x 10 mL). The combined organic layers were washed with brine (20 mL), dried over $MgSO_4$ and concentrated in vacuo. Purification by prep. HPLC afforded the product (1.0 mg, 1% over two steps). **$^1$H-NMR** (700 MHz, DMSO): δ 8.47 (d, *J* = 5.2 Hz, 2H), 8.40 (s, 1H), 8.27 (s, 1H), 7.79-7.77 (m, 1H), 7.29 (dd, *J* = 7.8, 4.9 Hz, 1H), 7.23 (d, *J* = 7.8 Hz, 1H), 6.13 (s, 1H), 5.40 (d, *J* = 6.9 Hz, 1H), 5.33-5.27 (m, 3H), 3.27-3.22 (m, 1H), 3.17 (dd, *J* = 14.4, 6.2 Hz, 1H), 3.01 (dd, *J* = 14.4, 5.9 Hz, 1H), 2.99-2.94 (m, 2H), 2.92-2.87 (m, 1H), 2.33 (s, 1H), 2.19-2.15 (m, 1H), 1.82-1.75 (m, 2H), 1.69-1.65 (m, 1H). **$^{13}$C-NMR** (176 MHz, DMSO): δ 210.4, 168.1, 160.3, 158.1, 157.9, 149.1, 137.0, 122.5, 121.3, 104.5, 94.2, 83.4, 77.4, 71.4, 47.4, 39.2, 38.8, 28.6, 27.1, 21.1. **HRMS**-ESI (m/z): $[M + H]^+$ calculated for $C_{21}H_{24}N_3O_4^+$, 382.1761; found, 382.1763.

**(±) *Tert*-butyl-8'-((2-chloropyridin-4-yl)methyl)-7'-methyl-3',9'-dioxo-3',4',4a',8',9',9b'-hexahydrospiro[piperidine-4,2'-pyrano[2',3':4,5]furo[3,2-c]pyridine]-1-carboxylate (13di)**

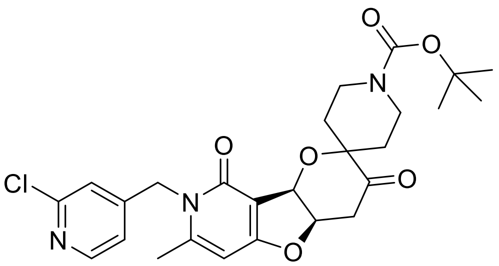


According to GP6, **8dd** (48.8 mg, 0.15 mmol) was reacted with **5r** (37.6 mg, 0.15 mmol). Purification by MPLC (cyclohexane/EtOAc 1:0 to 0:1) afforded the product (54.1 mg, 70%). **$^1$H-NMR** (500 MHz, $CD_2Cl_2$): δ 8.31 (d, *J* = 5.2 Hz, 1H), 7.05 (s, 1H), 7.02 (dd, *J* = 5.2, 1.5 Hz, 1H), 5.95 (s, 1H), 5.46-5.46 (m, 2H), 5.22-5.09 (m, 2H), 3.94-3.73 (m, 2H), 3.23-3.00 (m, 4H), 2.25 (s, 3H), 1.91-1.85 (m, 1H), 1.69 (dtd, *J* = 30.4, 12.2, 4.5 Hz, 2H), 1.54 (dt, *J* = 15.1, 4.7 Hz, 1H), 1.41 (s, 9H). **$^{13}$C-NMR** (126 MHz, $CD_2Cl_2$): δ 211.2, 169.3, 161.2, 154.8, 152.4, 151.5, 150.4, 149.9, 121.9, 120.6, 106.3, 96.2, 84.4, 81.1, 79.6, 71.8, 45.8, 40.0, 39.5, 38.6, 33.1, 28.5, 21.7. **HRMS**-ESI (m/z): $[M + H]^+$ calculated for $C_{26}H_{31}ClN_3O_6^+$, 516.1896; found, 516.1892.

**(±) 8'-((2-chloropyridin-4-yl)methyl)-7'-methyl-3',9'-dioxo-3',4',4a',8',9',9b'-hexahydrospiro[piperidine-4,2'-pyrano[2',3':4,5]furo[3,2-c]pyridin]-1-ium triflate (13ei)**

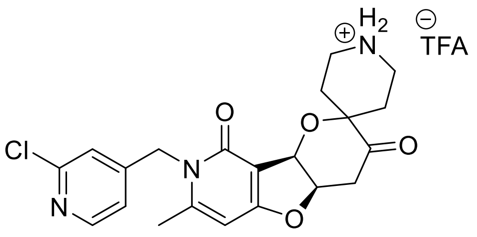

**13di** (42.1 mg, 0.08 mmol) was dissolved in DCM (1 mL) and cooled to 0 °C. A mixture of TMSOTf (0.15 mL, 10 equiv) and 2,6-lutidine (0.14 mL, 15 equiv) in DCM (0.1 mL) was added slowly and the mixture was stirred at 0 °C for 1 hour and at room temperature for 30 min. The reaction was quenched by slow addition of saturated $Na_2CO_3$ solution at 0 °C and dilution with EtOAc (10 mL). The layers were separated and the aqueous phase was extracted with EtOAc (3 x 10 mL). The combined organic layers were washed with brine (20 mL), dried over $MgSO_4$ and concentrated in vacuo. Purification by prep. HPLC afforded the product (3.5 mg, 10%). **$^1$H-NMR** (700 MHz, DMSO): δ 8.47 (s, 1H), 8.41-8.32 (m, 2H), 7.21 (s, 1H), 7.09 (d, *J* = 5.2 Hz, 1H), 6.20 (s, 1H), 5.42 (d, *J* = 6.8 Hz, 1H), 5.33-5.23 (m, 3H), 3.27-3.14 (m, 2H), 3.10-3.04 (m, 2H), 3.01-2.96 (m, 1H), 2.94-2.87 (m, 1H), 2.28 (s, 3H), 2.23-2.19 (m, 1H), 1.85-1.78 (m, 2H), 1.71 (dd, *J* = 14.4, 3.1 Hz, 1H). **$^{13}$C-NMR** (176 MHz, DMSO): δ 210.6, 168.9, 160.7, 152.8, 151.2, 151.1, 150.1, 122.0, 121.0, 105.3, 95.5, 84.1, 77.9, 71.5, 45.4, 39.4, 39.3, 29.4, 27.1, 21.4. **HRMS**-ESI (m/z): $[M + H]^+$ calculated for $C_{21}H_{23}ClN_3O_4^+$, 416.1372; found, 416.1369.

**(±) 6',9'-dioxo-5',6',6b',9',10',10a'-hexahydrospiro[piperidine-4,8'-pyrano[2',3':4,5]furo[3,2-c]quinolin]-1-ium triflate (13ej)**

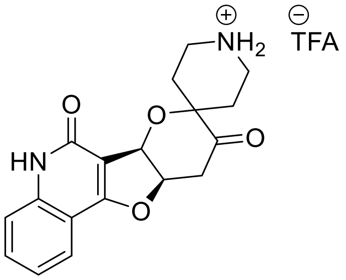

According to GP6, **8d** (50.0 mg, 0.15 mmol) was reacted with 4-hydroxyquinolin-2(1H)-one (24.8 mg, 0.15 mmol). Purification by MPLC (cyclohexane/EtOAc 1:0 to 0:1) afforded the product which was directly dissolved in HCl/dioxane (0.5 mL, 4M) at 0 °C and stirred at room temperature for 90 min. The reaction mixture was diluted by addition of DCM (10 mL) and 1 M HCl (10 mL). The layers were separated and the organic phase was extracted with 1 M HCl (3 x 10 mL). The combined aqueous layers were concentrated in vacuo and purified by prep. HPLC to afford the product (2.6 mg, 4% over two steps). **$^1$H-NMR** (500 MHz, MeOH-$d_4$): δ 7.80 (dd, *J* = 8.1, 1.4 Hz, 1H), 7.66 (ddd, *J* = 8.5, 8.1, 1.4 Hz, 1H), 7.43 (d, *J* = 8.5 Hz, 1H), 7.31 (t, *J* = 8.1 Hz, 1H), 5.61 (d, *J* = 6.8 Hz, 1H), 5.43 (qd, *J* = 6.8, 2.5 Hz, 1H), 3.43 (td, *J* = 13.0, 3.2 Hz, 1H), 3.37-3.31 (m, 2H), 3.28-3.18 (m, 3H), 2.37 (dq, *J* = 15.2, 3.1 Hz, 1H), 2.04 (ddd, *J* = 15.2, 13.0,

4.5 Hz, 1H), 1.94 (ddd, *J* = 14.8, 12.9, 4.5 Hz, 1H), 1.84 (dq, *J* = 14.8, 3.2 Hz, 1H). **$^{13}$C-NMR** (126 MHz, MeOH-d$_4$): δ 210.3, 168.3, 163.5, 141.9, 133.9, 124.1, 124.0, 117.2, 112.3, 108.7, 86.0, 79.3, 72.9, 40.5, 40.4, 40.1, 31.0, 27.4. **HRMS**-ESI (m/z): [M + H]$^+$ calculated for $C_{18}H_{19}N_2O_4^+$, 327.1339; found, 327.1342.

**(±) 5'-methyl-6',9'-dioxo-5',6',6b',9',10',10a'-hexahydrospiro[piperidine-4,8'-pyrano[2',3':4,5]furo[3,2-c]quinolin]-1-ium triflate (13ek)**

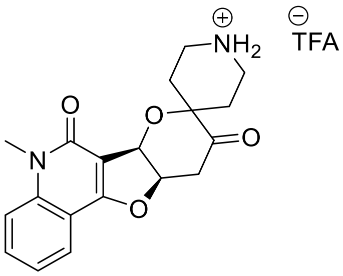


According to GP6, **8d** (97.6 mg, 0.30 mmol) was reacted with 4-hydroxy-1-methylquinolin-2(1H)-one (52.6 mg, 0.30 mmol). Purification by MPLC (cyclohexane/EtOAc 1:0 to 0:1) afforded the product which was directly dissolved in DCM (1 mL) and cooled to 0 °C. A mixture of TMSOTf (0.34 mL, 10 equiv) and 2,6-lutidine (0.32 mL, 15 equiv) in DCM (0.1 mL) was added slowly and the mixture was stirred at 0 °C for 30 min. The reaction was quenched by slow addition of saturated $Na_2CO_3$ solution at 0 °C and dilution with EtOAc (10 mL). The layers were separated and the aqueous phase was extracted with EtOAc (3 x 10 mL). The combined organic layers were washed with brine (20 mL), dried over $MgSO_4$ and concentrated in vacuo. Purification by prep. HPLC afforded the product (2.6 mg, 3% over two steps). **$^1$H-NMR** (700 MHz, DMSO): δ 8.52 (s, 1H), 8.39 (s, 1H), 7.76 (m, 2H), 7.63 (d, *J* = 8.9 Hz, 1H), 7.34 (t, *J* = 7.5 Hz, 1H), 5.50 (d, *J* = 6.7 Hz, 1H), 5.43 (q, *J* = 6.7 Hz, 1H), 3.62 (s, 3H), 3.32-3.29 (m, 1H), 3.22 (d, *J* = 6.7 Hz, 2H), 3.15 (d, *J* = 12.6 Hz, 1H), 3.10-3.03 (m, 1H), 2.99-2.92 (m, 1H), 2.32 (dd, *J* = 14.9, 3.0 Hz, 1H), 1.90-1.85 (m, 1H), 1.81-1.72 (m, 2H). **$^{13}$C-NMR** (176 MHz, DMSO): δ 210.0, 163.9, 159.5, 141.3, 132.8, 123.1, 122.1, 115.6, 110.9, 107.1, 84.1, 77.6, 71.5, 40.0, 38.9, 38.8, 29.3, 28.7, 25.8. **HRMS**-ESI (m/z): [M + H]$^+$ calculated for $C_{19}H_{21}N_2O_4^+$, 341.1496; found, 341.1500.

**2-(4-hydroxy-1,6-dimethyl-2-oxo-1,2-dihydropyridin-3-yl)-5-oxo-1-oxa-9-azaspiro[5.5]undec-2-en-9-ium triflate (18a)**

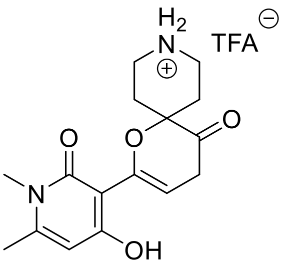


**13da** (30.0 mg, 0.07 mmol) was dissolved in dioxane (0.5 mL) and cooled to 0 °C. HCl in dioxane (0.18 mL, 4 M, 10 equiv) was added slowly and the mixture was stirred at 0 °C for 1 hour and at room temperature for 30 min. The reaction was quenched by slow addition of saturated $Na_2CO_3$ solution at 0 °C and diluted with EtOAc (10 mL). The layers were seperated and the aqueous phase was extracted with EtOAc (3 x 10 mL). The

combined organic layers were washed with brine (20 mL), dried over $MgSO_4$ and concentrated in vacuo. The crude was purified by prep. HPLC to afford the product (2.7 mg, 11%). **$^1$H-NMR** (700 MHz, DMSO): δ 8.77 (s, 1H), 8.51 (s, 1H), 6.65 (s, 1H), 6.58 (s, 1H), 4.25 (bs, 2H), 3.47 (s, 3H), 2.41 (s, 3H), 3.21 (dt, *J* = 12.7, 3.4 Hz, 1H), 3.12-3.06 (m, 1H), 1.92 (td, *J* = 13.6, 4.4 Hz, 1H), 1.80-1.76 (m, 1H). **$^{13}$C-NMR** (176 MHz, DMSO): δ 209.3, 158.9, 158.6, 149.6, 143.5, 113.2, 105.4, 94.6, 73.8, 39.0, 35.6, 30.3, 29.5, 20.9. **HRMS**-ESI (m/z): $[M + H]^+$ calculated for $C_{16}H_{21}O_4N_2^+$, 305.1496; found, 305.1491.

**2-(1-cyclobutyl-4-hydroxy-6-methyl-2-oxo-1,2-dihydropyridin-3-yl)-5-oxo-1-oxa-9-azaspiro[5.5]undec-2-en-9-ium triflate (18b)**

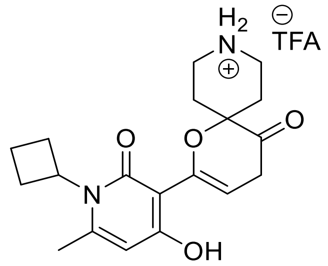


**13db** (46.0 mg, 0.10 mmol) was dissolved in dioxane (1 mL) and cooled to 0 °C. HCl in dioxane (0.26 mL, 4 M, 10 equiv) was added slowly and the mixture was stirred at 0 °C for 1 hour and at room temperature for 30 min. The reaction was evaporated to dryness and treated with $Et_2O$. The precipitate was filtered off and dried in vacuo. The crude was purified by prep. HPLC to afford the product (39.4 mg, 83%). **$^1$H-NMR** (700 MHz, DMSO): δ 8.76 (s, 1H), 8.48 (s, 1H), 6.61 (s, 1H), 6.50 (s, 1H), 4.86 (p, *J* = 8.8 Hz, 1H), 4.25 (bs, 2H), 3.24-3.14 (m, 4H), 3.10 (p, *J* = 12.3, 11.1 Hz, 2H), 2.41 (s, 3H), 2.19 (qt, *J* = 8.6, 2.6 Hz, 2H), 1.92 (td, *J* = 13.9, 4.1 Hz, 2H), 1.88-1.82 (m, 1H), 1.78 (d, *J* = 13.7 Hz, 2H), 1.74-1.68 (m, 1H). **$^{13}$C-NMR** (176 MHz, DMSO): δ 209.3, 160.3, 158.3, 149.6, 142.9, 115.1, 105.4, 95.4, 73.7, 51.9, 39.0, 35.5, 29.5, 26.9, 21.5, 14.1. **HRMS**-ESI (m/z): $[M + H]^+$ calculated for $C_{19}H_{25}N_2O_4^+$, 345.1801; found, 345.1808.

**2-(1-benzyl-4-hydroxy-6-methyl-2-oxo-1,2-dihydropyridin-3-yl)-5-oxo-1-oxa-9-azaspiro[5.5]undec-2-en-9-ium (18c)**

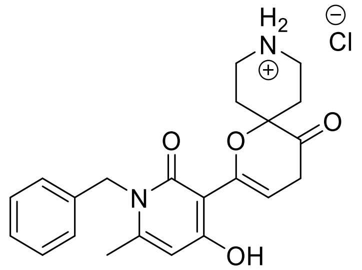


According to GP6, **8d** (50.0 mg, 0.15 mmol) was reacted with **5c** (33.1 mg, 0.15 mmol). Purification by MPLC (cyclohexane/EtOAc 1:0 to 0:1) afforded the product which was directly dissolved in HCl/dioxane (0.5 mL, 4M) at 0 °C and stirred at room temperature for 90 min. The reaction mixture was diluted by addition of DCM (10 mL) and 1 M HCl (10 mL). The layers were separated and the organic phase was extracted with 1 M HCl (3 x 10 mL). The combined aqueous layers were concentrated in vacuo to afford the product (22.0 mg, 35% over two steps). **$^1$H-NMR** (400 MHz, MeOH-$d_4$): δ 7.31 (t, *J* = 7.5 Hz, 2H), 7.25 (d, *J* = 7.5 Hz, 1H), 7.10 (d, *J* = 7.5 Hz, 2H),

6.80 (s, 1H), 6.67 (s, 1H), 5.48 (s, 2H), 4.30-4.28 (m, 2H), 3.36-3.29 (m, 4H), 2.40 (s, 3H), 2.20-2.09 (m, 2H), 1.98-1.90 (m, 2H). **$^{13}$C-NMR** (400 MHz, MeOH-d$_4$): δ 210.0, 161.9, 161.4, 152.2, 145.0, 138.0, 129.9, 128.4, 127.1, 115.5, 106.3, 98.5, 75.3, 48.2, 40.8, 36.7, 31.1, 21.1. **HRMS**-ESI (m/z): $[M + H]^+$ calculated for $C_{22}H_{25}N_2O_4^+$, 381.1809; found, 381.1810.

**2-(1-(4-fluorobenzyl)-4-hydroxy-6-methyl-2-oxo-1,2-dihydropyridin-3-yl)-5-oxo-1-oxa-9-azaspiro[5.5]undec-2-en-9-ium triflate (18d)**

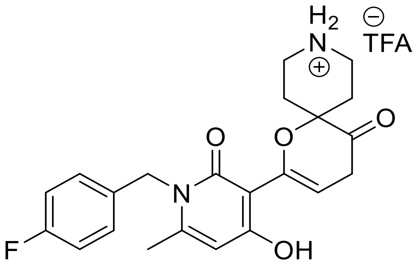


**13dd** (40.0 mg, 0.08 mmol) was dissolved in dioxane (1 mL) and cooled to 0 °C. HCl in dioxane (0.20 mL, 4 M, 10 equiv) was added slowly and the mixture was stirred at 0 °C for 1 hour and at room temperature for 30 min. The reaction was quenched by slow addition of saturated $Na_2CO_3$ solution at 0 °C and diluted with EtOAc (10 mL). The layers were seperated and the aqueous phase was extracted with EtOAc (3 x 10 mL). The combined organic layers were washed with brine (20 mL), dried over $MgSO_4$ and concentrated in vacuo. The crude was purified by prep. HPLC to afford the product (11.1 mg, 35%). **$^1$H-NMR** (700 MHz, MeOH-d$_4$): δ 7.15 (dd, *J* = 8.6, 5.4 Hz, 2H), 7.05 (t, *J* = 8.6 Hz, 2H), 6.77 (s, 1H), 6.64 (s, 1H), 5.45 (bs, 2H), 4.28 (bs, 2H), 3.35-3.32 (m, 4H), 2.41 (s, 3H), 2.14-2.08 (m, 2H), 1.95-1.90 (m, 2H). **$^{13}$C-NMR** (126 MHz, $CD_2Cl_2$): δ 210.0, 164.1, 162.7, 162.1, 161.3, 151.9, 144.7, 134.2, 129.2, 116.5, 115.6, 106.4, 98.2, 75.2, 47.4, 40.8, 36.6, 31.1, 21.1. **HRMS**-ESI (m/z): $[M + H]^+$ calculated for $C_{22}H_{24}N_2O_4F^+$, 399.1715; found, 399.1705.

**2-(4-hydroxy-1-(4-methoxybenzyl)-6-methyl-2-oxo-1,2-dihydropyridin-3-yl)-5-oxo-1-oxa-9-azaspiro[5.5]undec-2-en-9-ium triflate (18e)**

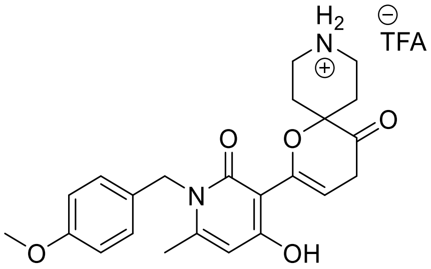


According to GP6, **8d** (50.0 mg, 0.15 mmol) was reacted with **5d** (37.7 mg, 0.15 mmol). Purification by MPLC (cyclohexane/EtOAc 1:0 to 0:1) afforded the product which was directly dissolved in HCl/dioxane (0.5 mL, 4M) at 0 °C and stirred at room temperature for 90 min. The reaction mixture was diluted by addition of DCM (10 mL) and 1 M HCl (10 mL). The layers were separated and the organic phase was extracted with 1 M HCl (3 x 10 mL). The combined aqueous layers were concentrated in vacuo and purified by prep. HPLC to afford the product (7.7 mg, 10% over two steps). **$^1$H-NMR** (700 MHz, MeOH-d$_4$): δ 7.06 (d, *J* = 8.8 Hz, 2H), 6.87 (d, *J* = 8.8 Hz, 2H), 6.77 (s, 1H), 6.61 (s, 1H), 5.43-5.38 (m, 2H), 4.28 (s, 2H), 3.76 (s, 3H),

3.33 (dd, $J$ = 7.4, 2.9 Hz, 4H), 2.41 (s, 3H), 2.11 (dt, $J$ = 14.3, 8.7 Hz, 2H), 1.92 (dq, $J$ = 15.0, 2.8 Hz, 2H). **$^{13}$C-NMR** (176 MHz, MeOH-d$_4$): δ 210.0, 162.1, 161.3, 160.5, 151.7, 144.9, 130.1, 128.6, 115.6, 115.2, 106.4, 98.0, 75.2, 55.7, 47.5, 40.8, 36.6, 31.2, 21.1. **HRMS**-ESI (m/z): [M + H]$^+$ calculated for $C_{23}H_{27}N_2O_5^+$, 411.1915; found, 411.1933.

**2-(4-hydroxy-6-methyl-2-oxo-1-(thiophen-2-ylmethyl)-1,2-dihydropyridin-3-yl)-5-oxo-1-oxa-9-azaspiro[5.5]undec-2-en-9-ium chloride (18f)**

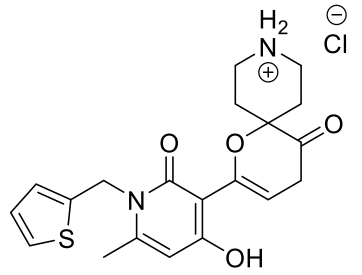


According to GP6, **8d** (50.0 mg, 0.15 mmol) was reacted with **5j** (34.0 mg, 0.15 mmol). Purification by MPLC (cyclohexane/EtOAc 1:0 to 0:1) afforded the product which was directly dissolved in HCl/dioxane (0.5 mL, 4M) at 0 °C and stirred at room temperature for 90 min. The reaction mixture was diluted by addition of DCM (10 mL) and 1 M HCl (10 mL). The layers were separated and the organic phase was extracted with 1 M HCl (3 x 10 mL). The combined aqueous layers were concentrated in vacuo to afford the product (10.1 mg, 16% over two steps). **$^1$H-NMR** (500 MHz, MeOH-d$_4$): δ 7.31 (d, $J$ = 5.2 Hz, 1H), 7.05 (d, $J$ = 3.6 Hz, 1H), 6.95 (dd, $J$ = 5.2, 3.6 Hz, 1H), 6.78 (s, 1H), 6.62 (s, 1H), 4.30-4.28 (m, 2H), 3.36-3.30 (m, 4H), 2.56 (s, 3H), 2.16-2.08 (m, 2H), 1.95-1.89 (m, 2H). **$^{13}$C-NMR** (126 MHz, MeOH-d$_4$): δ 210.0, 161.6, 161.4, 152.0, 144.4, 140.5, 127.7, 127.6, 126.7, 115.5, 106.3, 98.4, 75.2, 43.9, 40.8, 36.7, 31.1, 21.0. **HRMS**-ESI (m/z): [M + H]$^+$ calculated for $C_{20}H_{23}N_2O_4S^+$, 387.1373; found, 387.1371.

**(±) 7'-methyl-3',9'-dioxo-8'-(pyridin-4-ylmethyl)-3',4',4a',8',9',9b'-hexahydrospiro[piperidine-4,2'-pyrano[2',3':4,5]furo[3,2-c]pyridin]-1-ium chloride (18g)**

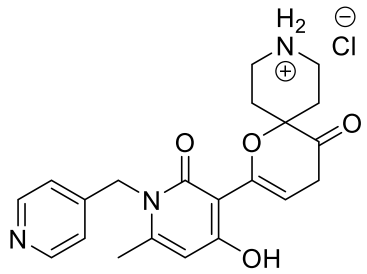


**13dg** (37.3 mg, 0.08 mmol) was dissolved in dioxane (1 mL) and cooled to 0 °C. HCl in dioxane (0.19 mL, 4 M, 10 equiv) was added slowly and the mixture was stirred at 0 °C for 1 hour and at room temperature for 30 min. The reaction was evaporated to dryness and treated with $Et_2O$. The precipitate was filtered off and dried in vacuo to afford the product (29.5 mg, quant.). **$^1$H-NMR** (500 MHz, MeOH-d$_4$): δ 8.78 (bs, 2H), 7.81 (bs, 2H), 6.77 (bs, 2H), 5.69 (bs, 2H), 4.28 (bs, 2H), 3.61 (bs, 1H), 3.31-3.25 (m, 4H), 2.40 (s, 3H), 2.15-2.07 (m, 2H), 1.94-1.86 (m, 2H). **$^{13}$C-NMR** (126 MHz, MeOH-d$_4$): δ 210, 161.6, 161.0, 152.6, 144.1, 142.9, 125.9, 115.6, 109.0, 106.4, 98.8. 75.3, 68.1, 49.5, 40.8, 31.0. **HRMS**-ESI (m/z): [M + H]$^+$ calculated for $C_{21}H_{24}N_3O_4^+$, 382.1761; found, 382.1760.

**2-(4-hydroxy-6-methyl-2-oxo-1-(pyridin-2-ylmethyl)-1,2-dihydropyridin-3-yl)-5-oxo-1-oxa-9-azaspiro[5.5]undec-2-en-9-ium chloride (18h)**

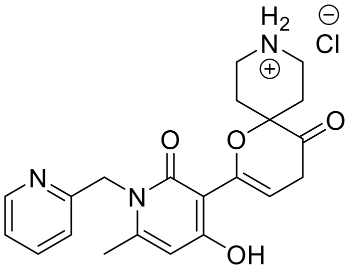

According to GP6, **8d** (50.0 mg, 0.23 mmol) was reacted with **5m** (33.2 mg, 0.23 mmol). Purification by MPLC (cyclohexane/EtOAc 1:0 to 0:1) afforded the product which was directly dissolved in HCl/dioxane (0.5 mL, 4M) at 0 °C and stirred at room temperature for 90 min. The reaction mixture was diluted by addition of DCM (10 mL) and 1 M HCl (10 mL). The layers were separated and the organic phase was extracted with 1 M HCl (3 x 10 mL). The combined aqueous layers were concentrated in vacuo and freeze-dried to afford the product (19.7 mg, 20 % over two steps).**$^1$H NMR** (500 MHz, MeOH-$d_4$): δ 8.79 (d, *J* = 5.7 Hz, 1H), 8.52 (t, *J* = 7.9 Hz, 1H), 7.97 (t, *J* = 6.6 Hz, 1H), 7.70 (d, *J* = 7.9 Hz, 1H), 6.74 (s, 1H), 6.68 (s, 1H), 5.68 (s, 1H), 4.25 (s, 1H), 3.28 (dd, *J* = 7.2, 3.4 Hz, 2H), 2.52 (s, 2H), 2.08 (ddd, *J* = 14.6, 10.7, 6.6 Hz, 1H), 1.87 (d, *J* = 14.6 Hz, 1H). **$^{13}$C NMR** (126 MHz, MeOH-$d_4$): δ 210.0, 161.8, 154.1, 152.5, 148.8, 144.1, 142.8, 127.2, 126.2, 115.6, 106.2, 99.0, 75.3, 49.5, 49.3, 49.2, 49.0, 48.8, 48.7, 48.5, 46.9, 40.8, 36.7, 31.0, 21.4. **HRMS**-ESI (m/z): $[M + H]^+$ calculated for $C_{21}H_{24}O_4N_3^+$,382.17613; found, 382.17593.

**2-(4-hydroxy-6-methyl-1-((3-methylpyridin-4-yl)methyl)-2-oxo-1,2-dihydropyridin-3-yl)-5-oxo-1-oxa-9-azaspiro[5.5]undec-2-en-9-ium chloride (18i)**

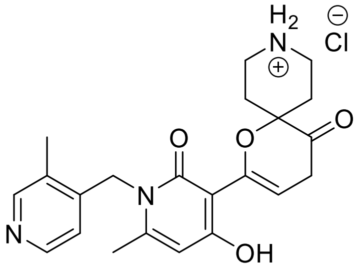

According to GP6, **8d** (50.0 mg, 0.23 mmol) was reacted with **5q** (62.5 mg, 0.23 mmol). Purification by MPLC (cyclohexane/EtOAc 1:0 to 0:1) afforded the product which was directly dissolved in HCl/dioxane (0.5 mL, 4M) at 0 °C and stirred at room temperature for 90 min. The reaction mixture was diluted by addition of DCM (10 mL) and 1 M HCl (10 mL). The layers were separated and the organic phase was extracted with 1 M HCl (3 x 10 mL). The combined aqueous layers were concentrated in vacuo and freeze-dried to afford the product (13.1 mg, 13% over two steps). **$^1$H NMR** (500 MHz, Methanol-d4): δ 8.74 (s, 1H), 8.54 (d, J = 5.5 Hz, 1H), 7.17 (d, J = 5.4 Hz, 1H), 6.77 (s, 1H), 6.73 (s, 1H), 5.60 (s, 2H), 4.29 (s, 2H), 3.30 (s, 4H), 2.65 (s, 2H), 2.41 (s, 2H), 2.11 (m, 2H), 1.90 (d, J = 14.4 Hz, 2H). **$^{13}$C NMR** (126 MHz, MeOH-$d_4$): δ 208.6, 160.3, 157.5, 151.1, 142.8, 140.8, 139.4, 136.6, 122.1, 114.2, 105.0, 98.0, 74.0, 48.1, 48.0, 48.0, 47.6, 47.5, 47.3, 47.1, 45.5, 39.4, 35.3, 29.7, 19.8, 15.0. **HRMS**-ESI (m/z): $[M + H]^+$ calculated for $C_{22}H_{26}O_4N_3^+$,396.19178; found, 396.19155.

**2-(1-((2-chloropyridin-1-ium-4-yl)methyl)-4-hydroxy-6-methyl-2-oxo-1,2-dihydropyridin-3-yl)-5-oxo-1-oxa-9-azaspiro[5.5]undecan-9-ium chloride (18j)**

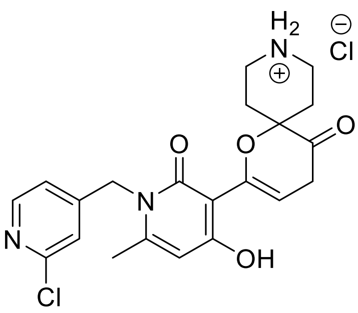


**13di** (33.2 mg, 0.06 mmol) was dissolved in dioxane (1 mL) and cooled to 0 °C. HCl in dioxane (0.16 mL, 4 M, 10 equiv) was added slowly and the mixture was stirred at 0 °C for 1 hour and at room temperature for 30 min. The reaction was evaporated to dryness and treated with $Et_2O$. The precipitate was filtered off and dried in vacuo to afford the product (29.1 mg, 99%). **$^1$H-NMR** (700 MHz, DMSO): δ 9.06 (s, 1H), 8.84 (s, 1H), 8.34 (d, *J* = 5.0 Hz, 1H), 7.21 (s, 1H), 7.06 (d, *J* = 5.0 Hz, 1H), 6.71 (bs, 2H), 5.38 (bs, 2H), 4.31 (bs, 2H), 3.21-3.15 (m, 2H), 3.10-3.04 (m, 2H), 2.31 (s, 3H), 2.01-1.95 (m, 2H), 1.84-1.79 (m 2H). **$^{13}$C-NMR** (176 MHz, DMSO): δ 209.3, 158.9, 158.8, 150.9, 150.6, 150.4, 150.3, 142.8, 121.5, 120.6, 113.5, 105.5, 95.8, 73.8, 45.1, 38.8, 35.6, 29.3, 20.5. **HRMS**-ESI (m/z): $[M + H]^+$ calculated for $C_{21}H_{23}ClN_3O_4^+$, 416.1372; found, 416.1362.

**2-(4-hydroxy-1-(2-(5-methoxy-1H-indol-3-yl)ethyl)-6-methyl-2-oxo-1,2-dihydropyridin-3-yl)-5-oxo-1-oxa-9-azaspiro[5.5]undec-2-en-9-ium triflate (18k)**

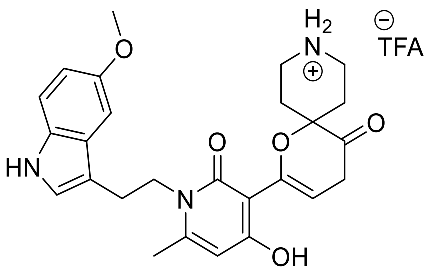


According to GP6, **8d** (50.0 mg, 0.15 mmol) was reacted with **5p** (45.8 mg, 0.15 mmol). Purification by MPLC (cyclohexane/EtOAc 1:0 to 0:1) afforded the product which was directly dissolved in HCl/dioxane (0.5 mL, 4M) at 0 °C and stirred at room temperature for 90 min. The reaction mixture was diluted by addition of DCM (10 mL) and 1 M HCl (10 mL). The layers were separated and the organic phase was extracted with 1 M HCl (3 x 10 mL). The combined aqueous layers were concentrated in vacuo and purified by prep. HPLC to afford the product (3.2 mg, 4% over two steps). **$^1$H-NMR** (500 MHz, MeOH-$d_4$): δ 7.20 (d, *J* = 9.0 Hz, 1H), 7.03 (s, 1H), 6.88 (d, *J* = 2.4 Hz, 1H), 6.80 (d, *J* = 0.8 Hz, 1H), 6.70 (dd, *J* = 9.0, 2.4 Hz, 1H), 6.43 (s, 1H), 4.39 (t, *J* = 7.1 Hz, 2H), 4.27 (s, 2H), 3.65 (s, 3H), 3.34 (dd, *J* = 7.1, 2.9 Hz, 4H), 3.17 (t, *J* = 7.1 Hz, 2H), 2.18 (s, 3H), 2.11 (ddd, *J* = 14.7, 9.7, 7.6 Hz, 2H), 1.95-1.89 (m, 2H). **$^{13}$C-NMR** (126 MHz, MeOH-$d_4$): δ 210.0, 161.9, 161.3, 155.1, 151.5, 144.9, 133.2, 129.1, 124.5, 115.7, 113.1, 113.0, 112.7, 106.3, 100.6, 97.6, 75.2, 56.1, 47.4, 40.8, 36.6, 31.1, 25.3, 21.1. **HRMS**-ESI (m/z): $[M + H]^+$ calculated for $C_{26}H_{30}N_3O_5^+$, 464.2180; found, 464.2177.

### 1.3.4. Synthesis of General Scaffold C Derivatives

**(±) 6,7-dimethyl-4a,9a-dihydro-2H-pyrano[3',2':4,5]furo[3,2-c]pyridine-3,5(4H,6H)-dione (14aa)**

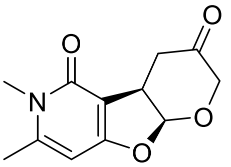


According to GP7, **8a** (50.0 mg, 0.32 mmol) was reacted with **5b** (44.6 mg, 0.32 mmol). Purification by MPLC (cyclohexane/EtOAc 1:0 to 0:1) afforded the product (4.9 mg, 7%). **$^1$H-NMR** (700 MHz, $CDCl_3$): δ 5.91 (s, 1H), 5.86 (bs, 1H), 3.87 (d, *J* = 11.7 Hz, 1H), 3.73 (dd, *J* = 11.7, 3.0 Hz, 1H), 3.55-3.45 (m, 4H), 2.56 (dd, *J* = 12.7, 3.0 Hz, 1H), 2.33 (s, 3H), 1.80 (dt, *J* = 12.7, 3.3 Hz, 1H). **$^{13}$C-NMR** (176 MHz, $CDCl_3$): δ 208.5, 163.6, 163.0, 146.3, 103.9, 101.0, 90.6, 69.5, 32.9, 31.5, 28.3, 21.2. **HRMS**-ESI (m/z): $[M + H]^+$ calculated for $C_{12}H_{14}NO_4^+$, 236.0917; found, 236.0917.

**(±) 6-benzyl-7-methyl-4a,9a-dihydro-2H-pyrano[3',2':4,5]furo[3,2-c]pyridine-3,5(4H,6H)-dione (14ab)**

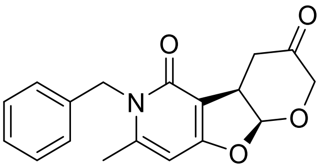


According to GP7, **8a** (100.0 mg, 0.64 mmol) was reacted with **5c** (137.9 mg, 0.64 mmol). Purification by MPLC (cyclohexane/EtOAc 1:0 to 0:1) afforded the product (56.0 mg, 30%). **$^1$H-NMR** (500 MHz, $CDCl_3$): δ 7.33-7.28 (m, 3H), 7.12-7.09 (m, 2H), 5.93 (s, 1H), 5.88 (bs, 1H), 5.33-5.29 (m, 2H), 3.89 (d, *J* = 11.6 Hz, 1H), 3.76 (dd, *J* = 11.6, 3.0 Hz, 1H), 3.55-3.52 (m, 1H), 2.59 (dd, *J* = 12.7, 3.0 Hz, 1H), 2.26 (s, 3H), 1.85 (dt, *J* = 12.7, 3.2 Hz, 1H). **$^{13}$C-NMR** (126 MHz, $CDCl_3$): δ 169.5, 164.1, 163.1, 146.8, 136.2, 129.0, 127.7, 126.5, 104.1, 101.8, 90.6, 69.4, 47.7, 32.9, 28.2, 20.8. **HRMS**-ESI (m/z): $[M + H]^+$ calculated for $C_{18}H_{18}NO_4^+$, 312.1230; found, 312.1234.

**(±) 6-(4-fluorobenzyl)-7-methyl-4a,9a-dihydro-2H-pyrano[3',2':4,5]furo[3,2-c]pyridine-3,5(4H,6H)-dione (14ac)**

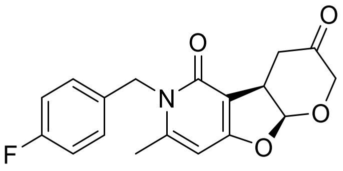


According to GP7, **8a** (50.0 mg, 0.32 mmol) was reacted with **5k** (74.7 mg, 0.32 mmol). Purification by MPLC (cyclohexane/EtOAc 1:0 to 0:1) afforded the product (24.5 mg, 23%). **$^1$H-NMR** (700 MHz, $CDCl_3$): δ 7.11 (dd, *J* = 8.6, 5.2 Hz, 2H), 7.00 (t, *J* = 8.6 Hz, 2H), 5.90-5.87 (m, 2H), 5.25 (bs, 2H), 3.88 (d, *J* = 11.6 Hz, 1H), 3.75 (dd, *J* = 11.6, 3.0 Hz, 1H), 3.52 (d, *J* = 2.5 Hz 1H), 2.57 (dd, *J* = 12.8, 3.0 Hz, 1H), 2.25 (s, 3H), 1.83 (dt, *J* = 12.8, 3.2 Hz, 1H). **$^{13}$C-NMR** (176 MHz, $CDCl_3$): δ 207.5, 169.4, 164.0, 163.0,

161.6, 146.4, 132.2, 128.4, 115.9, 104.1, 101.6, 90.7, 69.5, 46.9, 32.9, 28.2, 20.8. **HRMS**-ESI (m/z): $[M + H]^+$ calculated for $C_{18}H_{17}FNO_4^+$, 330.1136; found, 330.1141.

**(±) 6-(4-methoxybenzyl)-7-methyl-4a,9a-dihydro-2H-pyrano[3',2':4,5]furo[3,2-c]pyridine-3,5(4H,6H)-dione (14ad)**

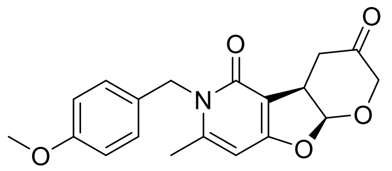

According to GP7, **8a** (50.0 mg, 0.32 mmol) was reacted with **5d** (78.5 mg, 0.32 mmol). Purification by MPLC (cyclohexane/EtOAc 1:0 to 0:1) afforded the product (33.3 mg, 30%). **$^1$H-NMR** (700 MHz, $CDCl_3$): δ 7.08 (d, *J* = 8.5 Hz, 2H), 6.83 (d, *J* = 8.5 Hz, 2H), 5.89-5.88 (m, 1H), 5.83 (s, 1H), 5.24-5.17 (m, 2H), 3.87 (d, *J* = 11.6 Hz, 1H), 3.77 (s, 3H), 3.74 (dd, *J* = 11.6, 3.0 Hz, 1H), 3.52 (q, *J* = 2.8 Hz, 1H), 2.56 (dd, *J* = 12.7, 3.0 Hz, 1H), 2.25 (s, 3H), 1.82 (dt, *J* = 12.7, 3.2 Hz, 1H). **$^{13}$C-NMR** (176 MHz, $CDCl_3$): δ 207.2, 163.5, 162.8, 158.9, 146.3, 128.5, 128.0, 114.2, 103.8, 100.9, 90.6. 69.4, 55.3, 46.8, 32.8, 28.2, 20.7. **HRMS**-ESI (m/z): $[M + H]^+$ calculated for $C_{19}H_{20}NO_5^+$, 342.1336; found, 342.1340.

**(±) 7-methyl-4a,9a-dihydro-2H,5H-furo[2,3-b:4,5-c']dipyran-3,5(4H)-dione (14ae)**

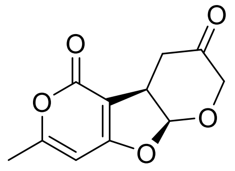

According to GP7, **8a** (50.0 mg, 0.32 mmol) was reacted with 4-hydroxy-6-methyl-2H-pyran-2-one (40.4 mg, 0.32 mmol). Purification by MPLC (cyclohexane/EtOAc 1:0 to 0:1) afforded the product (65.1 mg, 91%). **$^1$H-NMR** (500 MHz, $CDCl_3$): δ 6.38 (d, *J* = 8.3 Hz, 1H), 5.99 (s, 1H), 4.11 (d, *J* = 18.3 Hz, 1H), 4.04-3.97 (m, 1H), 3.90 (d, *J* = 18.3 Hz, 1H), 3.15 (dd, *J* = 16.1, 2.0 Hz, 1H), 2.80 (dd, *J* = 16.1, 6.3 Hz, 1H), 2.26 (s, 3H). **$^{13}$C-NMR** (126 MHz, $CDCl_3$): δ 207.1, 170.5, 167.4, 160.6, 105.4, 99.3, 94.8, 67.4, 36.5, 35.2, 20.7. **HRMS**-ESI (m/z): $[M + H]^+$ calculated for $C_{11}H_{11}O_5^+$, 223.0601; found, 223.0600.

**(±) 2,6,7-trimethyl-4a,9a-dihydro-2H-pyrano[3',2':4,5]furo[3,2-c]pyridine-3,5(4H,6H)-dione (14ba)**

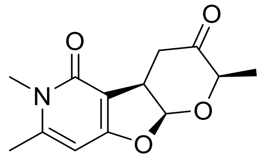

According to GP7, **8b** (53.0 mg, 0.31 mmol) was reacted with **5b** (43.3 mg, 0.31 mmol). Purification by MPLC (cyclohexane/EtOAc 1:0 to 0:1) afforded the product (66.9 mg, 86%). **$^1$H-NMR** (500 MHz, $CD_2Cl_2$): δ 6.15 (d, *J* = 7.8 Hz, 1H), 5.87 (s, 1H), 4.21 (q, *J* = 7.3 Hz, 1H), 3.90 (q, *J* = 6.7 Hz, 1H), 3.44 (s, 3H), 2.97 (dd, *J* = 14.7, 5.4 Hz, 1H), 2.89 (dd, *J* = 14.7, 6.7 Hz, 1H), 2.31 (s, 3H), 1.28 (d, *J* = 7.3 Hz, 3H).**$^{13}$C-NMR** (126 MHz, $CD_2Cl_2$): δ 211.1, 164.9, 161.8,

150.3, 106.5, 105.2, 94.6, 77.7, 39.8, 36.6, 30.6, 22.0, 17.7. **HRMS**-ESI (m/z): $[M + H]^+$ calculated for $C_{13}H_{16}NO_4^+$, 250.1074; found, 250.1073.

**(±) 2,2,7-trimethyl-4a,9a-dihydro-2H-pyrano[3',2':4,5]furo[3,2-c]pyridine-3,5(4H,6H)-dione (14ca)**

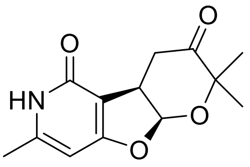

According to GP7, **8c** (50.0 mg, 0.27 mmol) was reacted with **5a** (34.0 mg, 0.27 mmol). Purification by MPLC (cyclohexane/EtOAc 1:0 to 0:1) afforded the product (3.5 mg, 5%). **$^1$H-NMR** (700 MHz, $CDCl_3$): δ 6.36 (d, *J* = 8.3 Hz, 1H), 5.93 (s, 1H), 4.10-4.06 (m, 1H), 3.08 (dt, *J* = 14.4, 2.5 Hz, 1H), 2.93 (dd, *J* = 14.4, 6.8 Hz, 1H), 2.34 (s, 3H), 1.36 (s, 3H), 1.27 (s, 3H). **$^{13}$C-NMR** (176 MHz, $CDCl_3$): δ 207.1, 186.6, 161.7, 149.3, 106.4, 105.4, 95.6, 82.4, 39.2, 34.6, 27.2, 25.6, 19.7. **HRMS**-ESI (m/z): $[M + H]^+$ calculated for $C_{13}H_{16}NO_4^+$, 250.1001; found, 250.1070.

**(±) 2,2,6,7-tetramethyl-4a,9a-dihydro-2H-pyrano[3',2':4,5]furo[3,2-c]pyridine-3,5(4H,6H)-dione (14cb)**

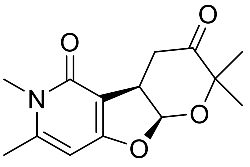

According to GP7, **8c** (50.0 mg, 0.27 mmol) was reacted with **5b** (37.7 mg, 0.27 mmol). Purification by MPLC (cyclohexane/EtOAc 1:0 to 0:1) afforded the product (18.2 mg, 25%). **$^1$H-NMR** (700 MHz, $CDCl_3$): δ 6.32 (d, *J* = 8.3 Hz, 1H), 5.94 (s, 1H), 4.12-4.08 (m, 1H), 3.50 (s, 3H), 3.06 (dd, *J* = 14.3, 2.9 Hz, 1H), 2.92 (dd, *J* = 14.3, 7.0 Hz, 1H), 2.35 (s, 3H), 1.34 (s, 3H), 1.26 (s, 3H). **$^{13}$C-NMR** (176 MHz, $CDCl_3$): δ 212.2, 165.7, 161.3, 150.1, 106.9, 105.2, 96.0, 82.3, 40.1, 34.5, 31.2, 27.2, 25.1, 21.9. **HRMS**-ESI (m/z): $[M + H]^+$ calculated for $C_{14}H_{18}NO_4^+$, 264.1230; found, 264.1235.

**(±) 6-benzyl-2,2,7-trimethyl-4a,9a-dihydro-2H-pyrano[3',2':4,5]furo[3,2-c]pyridine-3,5(4H,6H)-dione (14cc)**

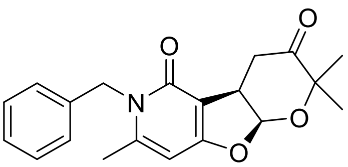

According to GP7, **8c** (50.0 mg, 0.27 mmol) was reacted with **5c** (58.4 mg, 0.27 mmol). Purification by MPLC (cyclohexane/EtOAc 1:0 to 0:1) afforded the product (31.2 mg, 34%). **$^1$H-NMR** (700 MHz, $CDCl_3$): δ 7.31 (t, *J* = 7.4 Hz, 2H), 7.24 (t, *J* = 7.4 Hz, 1H), 7.07 (d, *J* = 7.4 Hz, 2H), 6.36 (d, *J* = 8.3 Hz, 1H), 5.94 (s, 1H), 5.44 (d, *J* = 15.9 Hz, 1H), 5.19 (d, *J* = 15.9 Hz, 1H), 4.19-4.13 (m, 1H), 3.12 (dd, *J* = 14.2, 3.0 Hz, 1H), 2.95 (dd, *J* = 14.2, 6.9 Hz, 1H), 2.27 (s, 3H), 1.37 (s, 3H), 1.30 (s, 3H). **$^{13}$C-NMR** (176 MHz, $CDCl_3$): δ 212.2, 165.9, 161.3, 150.5, 136.2, 129.0, 127.6, 126.4, 107.0, 105.4, 96.5, 82.3,

47.2, 40.2, 34.4, 27.1, 25.0, 21.5. **HRMS**-ESI (m/z): [M + H]$^+$ calculated for $C_{20}H_{22}NO_4^+$, 340.1543; found, 340.1546.

**(±) 6-(2-chlorobenzyl)-2,2,7-trimethyl-4a,9a-dihydro-2H-pyrano[3',2':4,5]furo[3,2-c]pyridine-3,5(4H,6H)-dione (14cd)**

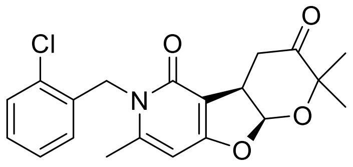


According to GP7, **8c** (50.0 mg, 0.27 mmol) was reacted with **5l** (67.8 mg, 0.27 mmol). Purification by MPLC (cyclohexane/EtOAc 1:0 to 0:1) afforded the product (19.9 mg, 20%). **$^1$H-NMR** (700 MHz, $CDCl_3$): δ 7.40-7.36 (m, 1H), 7.22-7.18 (m, 2H), 6.63 (dd, *J* = 7.0, 2.3 Hz, 1H), 6.38 (d, *J* = 8.3 Hz, 1H), 5.99 (s, 1H), 5.45 (d, *J* = 16.8 Hz, 1H), 5.33 (d, *J* = 16.8 Hz, 1H), 4.19-4.15 (m, 1H), 3.10 (dd, *J* = 14.2, 2.9 Hz, 1H), 2.96 (dd, *J* = 14.2, 6.9 Hz, 1H), 2.22 (s, 3H), 1.37 (s, 3H), 1.31 (s, 3H). **$^{13}$C-NMR** (176 MHz, $CDCl_3$): δ 212.2, 166.1, 161.3, 150.3, 133.5, 132.4, 129.7, 128.8, 127.7, 126.4, 107.0, 105.5, 96.7, 82.4, 44.8, 40.2, 34.4, 27.2, 25.0, 21.2. **HRMS**-ESI (m/z): [M + H]$^+$ calculated for $C_{20}H_{21}NO_4Cl^+$, 374.1154; found, 374.1156.

**(±) 6-(4-fluorobenzyl)-2,2,7-trimethyl-4a,9a-dihydro-2H-pyrano[3',2':4,5]furo[3,2-c]pyridine-3,5(4H,6H)-dione (14ce)**

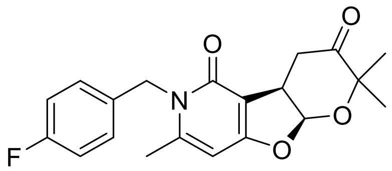


According to GP7, **8c** (50.0 mg, 0.27 mmol) was reacted with **5k** (63.3 mg, 0.27 mmol). Purification by MPLC (cyclohexane/EtOAc 1:0 to 0:1) afforded the product (15.6 mg, 16%). **$^1$H-NMR** (700 MHz, $CDCl_3$): δ 7.08 (dd, *J* = 8.7, 5.3 Hz, 2H), 7.00 (t, *J* = 8.7 Hz, 2H), 6.35 (d, *J* = 8.3 Hz, 1H), 5.92 (s, 1H), 5.37 (d, *J* = 15.7 Hz, 1H), 5.15 (d, *J* = 15.7 Hz, 1H), 4.17-4.12 (m, 1H), 3.12 (dd, *J* = 14.1, 2.9 Hz, 1H), 2.95 (dd, *J* = 14.1, 6.8 Hz, 1H), 2.27 (s, 3H), 1.37 (s, 3H), 1.29 (s, 3H). **$^{13}$C-NMR** (176 MHz, $CDCl_3$): δ 212.2, 165.8, 163.0, 161.6, 161.2, 150.1, 132.1, 128.3, 116.0, 106.9, 105.3, 96.3, 82.4, 46.5, 40.3, 34.4, 27.2, 24.8, 21.5. (700 MHz, $CDCl_3$): δ **HRMS**-ESI (m/z): [M + H]$^+$ calculated for $C_{20}H_{21}NO_4F^+$, 358.1449; found, 358.1453.

**(±) 6-(4-methoxybenzyl)-2,2,7-trimethyl-4a,9a-dihydro-2H-pyrano[3',2':4,5]furo[3,2-c]pyridine-3,5(4H,6H)-dione (14cf)**

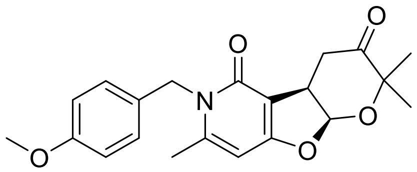

According to GP7, **8cc** (50.0 mg, 0.27 mmol) was reacted with **5d** (66.6 mg, 0.27 mmol). Purification by MPLC (cyclohexane/EtOAc 1:0 to 0:1) afforded the product (9.3 mg, 9%). **$^1$H-NMR** (700 MHz, $CDCl_3$): δ 7.04 (d, *J* = 8.8 Hz, 2H), 6.85 (d, *J* = 8.8 Hz, 2H), 6.36 (d, *J* = 8.3 Hz, 1H), 5.94 (s, 1H), 5.36 (d, *J* = 15.7 Hz, 1H), 5.14 (d, *J* = 15.7 Hz, 1H), 4.19-4.14 (m, 1H), 3.77 (s, 3H), 3.11 (dd, *J* = 14.2, 2.9 Hz, 1H), 2.96 (dd, *J* = 14.2, 6.9 Hz, 1H), 2.30 (s, 3H), 1.37 (s, 3H), 1.29 (s, 3H). **$^{13}$C-NMR** (176 MHz, $CDCl_3$): δ 212.2, 166.0, 161.4, 159.2, 150.5, 128.1, 128.0, 114.5, 107.1, 105.4, 96.7, 82.4, 55.4, 46.8, 34.5, 27.2, 25.1, 21.6. (700 MHz, $CDCl_3$): δ **HRMS**-ESI (m/z): $[M + H]^+$ calculated for $C_{21}H_{23}NO_5Na^+$, 392.1468; found, 392.1457.

**(±) *Tert*-butyl-7'-methyl-3',5'-dioxo-3',4',4a',5',6',9a'-hexahydrospiro[piperidine-4,2'-pyrano[3',2':4,5]furo[3,2-c]pyridine]-1-carboxylate (14da)**

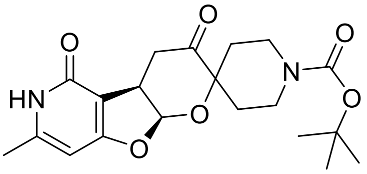

According to GP7, **8d** (97.6 mg, 0.30 mmol) was reacted with **5a** (37.5 mg, 0.30 mmol). Purification by prep. HPLC afforded the product (17.4 mg, 15%). **$^1$H-NMR** (700 MHz, $CDCl_3$): δ 6.47 (d, *J* = 8.4 Hz, 1H), 6.06 (s, 1H), 4.14 (td, *J* = 8.4, 7.3, 2.6 Hz, 1H), 4.00-3.81 (m, 2H), 3.25-3.13 (m, 2H), 3.07 (dd, *J* = 14.5, 2.6 Hz, 1H), 2.93 (dd, *J* = 14.5, 7.3 Hz, 1H), 2.40 (s, 3H), 1.81-1.63 (m, 3H), 1.46-1.42 (m, 10H). **$^{13}$C-NMR** (176 MHz, $CDCl_3$): δ 210.0, 169.4, 161.5, 154.8, 150.4, 106.5, 105.7, 97.0, 82.0, 80.0, 39.2, 38.8, 38.2, 34.6, 31.8, 28.6, 19.6. **HRMS**-ESI (m/z): $[M + H]^+$ calculated for $C_{20}H_{27}N_2O_6^+$, 391.1864; found, 391.1866.

**(±) 7'-methyl-3',5'-dioxo-3',4',4a',5',6',9a'-hexahydrospiro[piperidine-4,2'-pyrano[3',2':4,5]furo[3,2-c]pyridin]-1-ium triflate (14ea)**

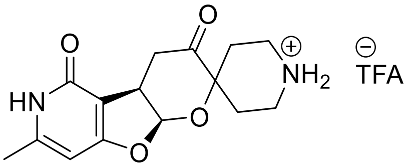

**14da** (17.4 mg, 0.04 mmol) was dissolved in DCM (3 mL) and cooled to 0 °C. HCl in dioxane (0.03 mL, 4 M, 10 equiv) was added slowly and the mixture was stirred at 0 °C for 1 hour and at room temperature for 30 min. The reaction was quenched by slow addition of saturated $Na_2CO_3$ solution at 0 °C and dilution with EtOAc (10 mL). The layers were separated and the aqueous phase was extracted with EtOAc (3 x 10 mL).

The combined organic layers were washed with brine (20 mL), dried over $MgSO_4$ and concentrated in vacuo. Purification by prep. HPLC afforded the product (2.4 mg, 18%). **$^{1}$H-NMR** (700 MHz, DMSO): δ 11.34 (s, 1H), 8.72 (s, 1H), 8.34 (s, 1H), 6.45 (d, *J* = 8.3 Hz, 1H), 5.84 (s, 1H), 4.09-4.06 (m, 1H), 3.27-3.19 (m, 2H), 3.11-3.03 (m, 3H), 2.90 (dd, *J* = 14.4, 2.7 Hz, 1H), 2.14 (s, 3H), 2.01-1.97 (m, 1H), 1.93-1.87 (m, 1H), 1.78-1.73 (m, 1H), 1.55-1.51 (m, 1H). **$^{13}$C-NMR** (176 MHz, DMSO): δ 210.8, 165.9, 160.3, 149.1, 105.1, 103.9, 92.1, 78.0, 38.9, 38.7, 38.2, 34.4, 30.8, 28.2, 18.8. **HRMS**-ESI (m/z): $[M + H]^+$ calculated for $C_{15}H_{19}N_2O_4^+$, 291.1339; found, 291.1342.

**(±) *Tert*-butyl-6',7'-dimethyl-3',5'-dioxo-3',4',4a',5',6',9a'-hexahydrospiro[piperidine-4,2'-pyrano[3',2':4,5]furo[3,2-c]pyridine]-1-carboxylate (14db)**

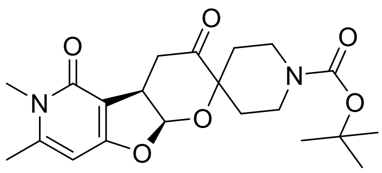

According to GP7, **8d** (116.9 mg, 0.36 mmol) was reacted with **5b** (50.0 mg, 0.36 mmol). Purification by MPLC (EtOAc/MeOH 1:0 to 9:1) afforded the product (39.8 mg, 27%). **$^{1}$H-NMR** (700 MHz, $CD_2Cl_2$): δ 6.35 (d, *J* = 8.6 Hz, 1H), 5.80 (s, 1H), 4.06 (ddd, *J* = 8.6, 6.8, 2.7 Hz, 1H), 3.90-3.88 (m, 2H), 3.41 (s, 3H), 3.22-3.10 (m, 2H), 3.08 (dd, *J* = 14.4, 2.7 Hz, 1H), 2.88 (dd, *J* = 14.4, 6.8 Hz, 1H), 2.30 (s, 3H), 1.71-1.64 (m, 2H), 1.59 (ddd, *J* = 13.4, 11.9, 4.7 Hz, 1H), 1.49-1.44 (m, 1H), 1.41 (s, 9H). **$^{13}$C-NMR** (176 MHz, $CD_2Cl_2$): δ 211.6, 165.1, 161.0, 154.8, 150.7, 105.8, 105.0, 94.4, 81.8, 79.6, 40.2, 39.6, 38.6, 35.1, 34.9, 31.9, 30.6, 28.5, 21.9. **HRMS**-ESI (m/z): $[M + H]^+$ calculated for $C_{21}H_{29}N_2O_6^+$, 405.2020; found, 405.2016.

**(±) 6',7'-dimethyl-4a',9a'-dihydrospiro[piperidine-4,2'-pyrano[3',2':4,5]furo[3,2-c]pyridine]-3',5'(4'H,6'H)-dione (14eb)**

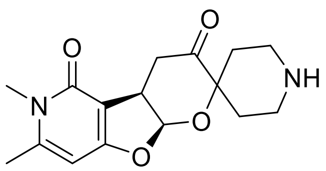

**14db** (19.5 mg, 0.05 mmol) was dissolved in dioxane (0.5 mL) and cooled to 0 °C. HCl in dioxane (0.12 mL, 4 M, 10 equiv) was added slowly and the mixture was stirred at 0 °C for 1 hour and at room temperature for 30 min. The reaction was quenched by slow addition of saturated $Na_2CO_3$ solution at 0 °C and diluted with EtOAc (10 mL). The layers were seperated and the aqueous phase was extracted with EtOAc (3 x 10 mL). The combined organic layers were washed with brine (20 mL), dried over $MgSO_4$ and concentrated in vacuo to afford the product (6.6 mg, 45%). **$^{1}$H-NMR** (700 MHz, MeOH-$d_4$): δ 6.46 (d, *J* = 8.3 Hz, 1H), 6.05 (s, 1H), 4.11-4.09 (m, 1H), 3.54- 3.47 (m, 4H), 3.21-3.11 (m, 1H), 3.04-2.98 (m, 2H), 2.87 (tt, *J* = 12.8, 4.0 Hz, 2H), 2.40 (s, 3H), 1.79-1.73 (m, 2H), 1.63 (ddd, *J* = 13.5, 11.9, 4.4

Hz, 1H), 1.52-1.45 (m, 1H). **$^{13}$C-NMR** (176 MHz, MeOH-$d_4$): δ 212.7, 167.1, 162.7, 152.7, 107.3, 106.5, 96.3, 82.4, 41.6, 41.4, 40.8, 35.8, 32.8, 31.2, 30.7, 21.5. **HRMS**-ESI (m/z): [M + H]$^+$ calculated for $C_{16}H_{20}N_2O_4^+$, 305.1496; found, 305.1501.

**(±) *Tert*-butyl-6'-isopentyl-7'-methyl-3',5'-dioxo-3',4',4a',5',6',9a'-hexahydrospiro[piperidine-4,2'-pyrano[3',2':4,5]furo[3,2-c]pyridine]-1-carboxylate (14dc)**

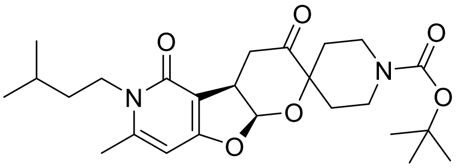

According to GP7, **8d** (97.6 mg, 0.30 mmol) was reacted with **5t** (58.6 mg, 0.30 mmol). Purification by MPLC (cyclohexane/EtOAc 1:0 to 0:1) afforded the product (100.0 mg, 72%). **$^1$H-NMR** (700 MHz, $CD_2Cl_2$): δ 6.34 (d, *J* = 8.4 Hz, 1H), 5.77 (s, 1H), 4.09-4.02 (m, 2H), 3.91-3.77 (m, 3H), 3.23-3.10 (m, 2H), 3.08 (dd, *J* = 14.3, 2.7 Hz, 1H), 2.87 (dd, *J* = 14.4, 6.7 Hz, 1H), 2.33 (s, 3H), 1.71-1.64 (m, 2H), 1.62-1.56 (m, 2H), 1.54-1.43 (m 3H), 1.42 (s, 9H), 0.97 (dd, *J* = 6.6, 1.6 Hz, 6H). **$^{13}$C-NMR** (176 MHz, $CD_2Cl_2$): δ 211.6, 165.0, 160.6, 154.8, 149.9, 106.1, 105.0, 94.6, 81.8, 79.6, 42.9, 40.3, 39.6, 38.6, 37.8, 35.0, 34.8, 32.0, 28.5, 27.0, 22.6, 21.3. **HRMS**-ESI (m/z): [M + H]$^+$ calculated for $C_{25}H_{37}N_2O_6^+$, 461.2646; found, 461.2638.

**(±) 6'-isopentyl-7'-methyl-4a',9a'-dihydrospiro[piperidine-4,2'-pyrano[3',2':4,5]furo[3,2-c]pyridine]-3',5'(4'H,6'H)-dione (14ec)**

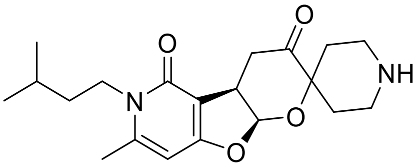

**14dc** (90.0 mg, 0.20 mmol) was dissolved in DCM (3 mL) and cooled to 0 °C. TFA (0.15 mL, 10 equiv) was added slowly and the mixture was stirred at 0 °C for 1 hour and at room temperature for 30 min. The reaction was quenched by slow addition of saturated $Na_2CO_3$ solution at 0 °C and dilution with EtOAc (10 mL). The layers were separated and the aqueous phase was extracted with EtOAc (3 x 10 mL). The combined organic layers were washed with brine (20 mL), dried over $MgSO_4$ and concentrated in vacuo to afford the product (61.0 mg, 87%). **$^1$H-NMR** (700 MHz, $CD_2Cl_2$): δ 6.33 (d, *J* = 8.3 Hz, 1H), 5.76 (s, 1H), 4.09-3.99 (m, 2H), 3.83-3.78 (m, 1H), 3.03 (dd, *J* = 14.2, 2.9 Hz, 1H), 3.01-2.95 (m, 2H), 2.89-2.83 (m, 2H), 2.33 (s, 3H), 1.72-1.65 (m, 3H), 1.59 (ddd, *J* = 13.4, 11.5, 4.5 Hz, 1H), 1.53-1.40 (m, 3H), 0.97 (dd, *J* = 6.6, 1.6 Hz, 6H). **$^{13}$C-NMR** (176 MHz, $CD_2Cl_2$): δ 211.7, 165.1, 160.7, 149.8, 106.3, 105.4, 94.7, 82.4, 42.9. 41.6, 41.4, 40.4, 37.8, 35.9, 34.8, 32.9, 27.0, 22.6, 21.3. **HRMS**-ESI (m/z): [M + H]$^+$ calculated for $C_{20}H_{29}N_2O_4^+$, 361.2122; found, 361.2119.

**(±) *Tert*-butyl 6'-benzyl-7'-methyl-3',5'-dioxo-3',4',4a',5',6',9a'-hexahydrospiro[piperidine-4,2'-pyrano[3',2':4,5]furo[3,2-c]pyridine]-1-carboxylate (14dd)**

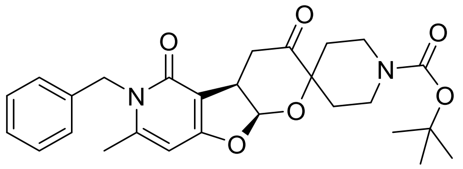

According to GP7, **8d** (97.6 mg, 0.30 mmol) was reacted with **5c** (64.6 mg, 0.30 mmol). Purification by MPLC (cyclohexane/EtOAc 1:0 to 0:1) afforded the product (94.7 mg, 66%). **$^{1}$H-NMR** (500 MHz, $CD_2Cl_2$): δ 7.35-7.20 (m, 3H), 7.10-7.02 (m, 2H), 6.40 (d, *J* = 8.3 Hz, 1H), 5.84 (s, 1H), 5.40 (d, *J* = 16.1 Hz, 1H), 5.10 (d, *J* = 16.1 Hz, 1H), 4.16-4.10 (m, 1H), 3.94-3.77 (m, 2H), 3.26-3.08 (m, 3H). 2.92 (dd, *J* = 14.5, 6.5 Hz, 1H), 2.22 (s, 3H), 1.93-1.55 (m, 4H), 1.42 (s, 9H). **$^{13}$C-NMR** (126 MHz, $CD_2Cl_2$): δ 211.5, 165.5, 160.1, 154.8, 150.8, 137.2, 129.1, 127.6, 126.5, 106.2, 105.2, 95.2, 81.9, 79.6, 46.7, 40.3, 39.5, 38.7, 35.0, 34.8, 31.8, 28.5, 21.6. **HRMS**-ESI (m/z): $[M + H]^+$ calculated for $C_{27}H_{33}N_2O_6^+$; 481.2333; found, 481.2328.

**(±) 6'-benzyl-7'-methyl-4a',9a'-dihydrospiro[piperidine-4,2'-pyrano[3',2':4,5]furo[3,2-c]pyridine]-3',5'(4'H,6'H)-dione (14ed)**

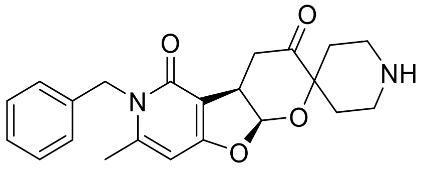

**14dd** (90.2 mg, 0.19 mmol) was dissolved in DCM (3 mL) and cooled to 0 °C. TFA (0.14 mL, 10 equiv) was added slowly and the mixture was stirred at 0 °C for 1 hour and at room temperature for 30 min. The reaction was quenched by slow addition of saturated $Na_2CO_3$ solution at 0 °C and dilution with EtOAc (10 mL). The layers were separated and the aqueous phase was extracted with EtOAc (3 x 10 mL). The combined organic layers were washed with brine (20 mL), dried over $MgSO_4$ and concentrated in vacuo to afford the product (60.1 mg, 84%). **$^{1}$H-NMR** (700 MHz, DMSO): δ 7.32 (t, *J* = 7.5 Hz, 2H), 7.25 (t, *J* = 7.5 Hz, 1H), 7.04 (d, *J* = 7.5 Hz, 2H), 6.48 (d, *J* = 8.3 Hz, 0H), 6.06 (s, 1H), 5.32 (d, *J* = 15.9 Hz, 1H), 5.16 (d, *J* = 15.9 Hz, 1H), 4.12-4.08 (m, 1H), 2.99 (dd, *J* = 14.1, 6.7 Hz, 1H), 2.86 (dd, *J* = 14.1, 2.7 Hz, 1H), 2.83-2.71 (m, 4H), 2.21 (s, 3H9, 1.67 (dd, *J* = 13.1, 2.9 Hz, 1H), 1.61 (ddd, *J* = 14.0, 11.4, 4.4 Hz, 1H), 1.45 (td, *J* = 12.4, 4.5 Hz, 1H), 1.38-1.33 (m, 1H). **$^{13}$C-NMR** (176 MHz, DMSO): δ 212.0, 165.0, 160.4, 160.0, 151.0, 137.7, 129.1, 127.5, 126.5, 105.9, 105.0, 94.8, 82.0, 45.9, 41.2, 40.9, 39.5, 35.2, 34.6, 32.3, 21.0. **HRMS**-ESI (m/z): $[M + H]^+$ calculated for $C_{22}H_{25}N_2O_4^+$, 381.1809; found, 381.1809.

**(±) *Tert*-butyl-6'-(4-fluorobenzyl)-7'-methyl-3',5'-dioxo-3',4',4a',5',6',9a'-hexahydrospiro[piperidine-4,2'-pyrano[3',2':4,5]furo[3,2-c]pyridine]-1-carboxylate (14de)**

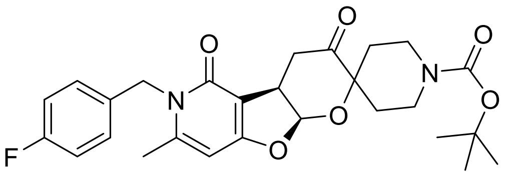


According to GP7, **8d** (97.6 mg, 0.30 mmol) was reacted with **5k** (70.0 mg, 0.30 mmol). Purification by MPLC (cyclohexane/EtOAc 1:0 to 0:1) afforded the product (119.8 mg, 80%). **$^1$H-NMR** (500 MHz, $CD_2Cl_2$): δ 7.10-6.99 (m, 4H), 6.40 (d, *J* = 8.0 Hz, 1H), 5.83 (s, 1H), 5.36 (d, *J* = 15.5 Hz, 1H), 5.07 (d, *J* = 15.5 Hz, 1H), 4.12 (ddd, *J* = 8.0, 6.5, 2.7 Hz, 1H), 3.95-3.79 (m, 2H), 3.25-3.13 (m, 2H), 3.12 (dd, *J* = 14.2, 2.7 Hz, 1H), 2.92 (dd, *J* = 14.2, 6.5 Hz, 1H), 2.23 (s, 3H), 1.77-1.57 (m, 3H), 1.51-1.46 (m, 1H), 1.42 (s, 9H). **$^{13}$C-NMR** (126 MHz, $CD_2Cl_2$): δ 211.2, 165.3, 163.1, 160.7, 154.4, 150.4, 132.9, 128.2, 115.6, 106.0, 105.0, 95.0, 81.7, 79.3, 45.9, 40.1, 38.7, 34.9, 34.5, 31.8, 28.2, 21.3. **HRMS**-ESI (m/z): $[M + H]^+$ calculated for $C_{27}H_{32}FN_2O_6^+$, 499.2239; found, 499.2236.

**(±) 6'-(4-fluorobenzyl)-7'-methyl-4a',9a'-dihydrospiro[piperidine-4,2'-pyrano[3',2':4,5]furo[3,2-c]pyridine]-3',5'(4'H,6'H)-dione (14ee)**

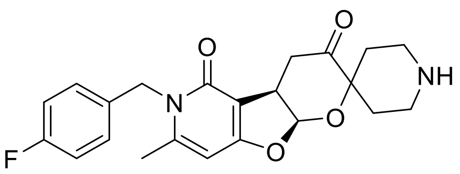


**14de** (119.8 mg, 0.24 mmol) was dissolved in DCM (3 mL) and cooled to 0 °C. TFA (0.19 mL, 10 equiv) was added slowly and the mixture was stirred at 0 °C for 1 hour and at room temperature for 30 min. The reaction was quenched by slow addition of saturated $Na_2CO_3$ solution at 0 °C and dilution with EtOAc (10 mL). The layers were separated and the aqueous phase was extracted with EtOAc (3 x 10 mL). The combined organic layers were washed with brine (20 mL), dried over $MgSO_4$ and concentrated in vacuo to afford the product (57.8 mg, 60%). **$^1$H-NMR** (500 MHz, DMSO): δ 7.18-7.08 (m, 4H), 6.47 (d, *J* = 8.7 Hz, 1H), 6.07 (s, 1H), 5.31 (d, *J* = 15.9 Hz, 1H), 5.11 (d, *J* = 15.9 Hz, 1H), 4.10 (ddd, *J* = 8.7, 6.8, 2.7 Hz, 1H), 3.00 (dd, *J* = 14.1, 6.8 Hz, 1H), 2.89-2.71 (m, 5H), 2.22 (s, 3H), 1.67 (dq, *J* = 13.3, 2.9 Hz, 1H), 1.61 (ddd, *J* = 13.6, 11.2, 4.7 Hz, 1H), 1.44 (ddd, *J* = 13.2, 11.4, 4.7 Hz, 1H), 1.35 (dq, *J* = 13.5, 2.9 Hz, 1H). **$^{13}$C-NMR** (126 MHz, DMSO): δ 211.6, 164.6, 162.2, 160.2, 160.0, 150.5, 133.4, 128.2, 115.5, 105.5, 104.5, 94.5, 81.5, 44.8, 40.7, 40.5, 39.0, 34.7,  34.2, 31.8. 20.5. **HRMS**-ESI (m/z): $[M + H]^+$ calculated for $C_{22}H_{24}FN_2O_4^+$, 399.1715; found, 399.1706.

**(±) *Tert*-butyl-6'-(4-methoxybenzyl)-7'-methyl-3',5'-dioxo-3',4',4a',5',6',9a'-hexahydrospiro[piperidine-4,2'-pyrano[3',2':4,5]furo[3,2-c]pyridine]-1-carboxylate (14df)**

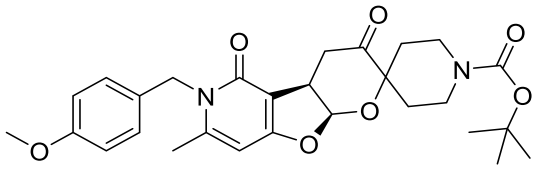

According to GP7, **8d** (97.6 mg, 0.30 mmol) was reacted with **5d** (73.6 mg, 0.30 mmol). Purification by MPLC (cyclohexane/EtOAc 1:0 to 0:1) afforded the product (65.0 mg, 42%). **$^{1}$H-NMR** (500 MHz, $CD_2Cl_2$): δ 7.01 (d, *J* = 8. Hz, 2H), 6.84 (d, *J* = 8.7 Hz, 2H), 6.39 (d, *J* = 8.4 Hz, 1H), 5.81 (s, 1H), 5.31 (d, *J* = 15.2 Hz, 1H), 5.03 (d, *J* = 15.2 Hz, 1H), 4.12 (ddd, *J* = 8.4, 6.6, 2.67Hz, 1H), 3.94-3.80 (m, 2H), 3.76 (s, 3H), 3.25-3.14 (m, 2H), 3.13 (dd, *J* = 14.2, 2.7 Hz, 1H), 2.91 (dd, *J* = 14.2, 6.6 Hz, 1H), 2.24 (s, 3H), 1.77-1.57 (m, 3H), 1.52-1.45 (m, 1H), 1.42 (s, 9H). **$^{13}$C-NMR** (126 MHz, $CD_2Cl_2$): δ 211.5, 165.4, 161.0, 159.3, 154.8, 150.8, 129.2, 128.0, 114.4, 106.1, 105.1, 95.1, 81.9, 79.6, 55.6, 46.2, 40.3, 39.1, 34.8, 31.8, 28.5, 21.6. **HRMS**-ESI (m/z): $[M + H]^+$ calculated for $C_{28}H_{35}N_2O_7^+$, 511.2439; found, 511.2436.

**(±) 6'-(4-methoxybenzyl)-7'-methyl-4a',9a'-dihydrospiro[piperidine-4,2'-pyrano[3',2':4,5]furo[3,2-c]pyridine]-3',5'(4'H,6'H)-dione (14ef)**

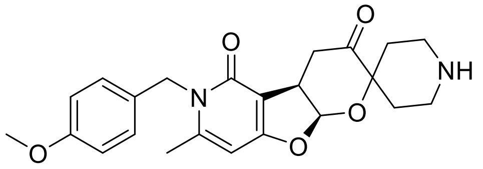

**14df** (55.0 mg, 0.11 mmol) was dissolved in DCM (3 mL) and cooled to 0 °C. TFA (0.08 mL, 10 equiv) was added slowly and the mixture was stirred at 0 °C for 1 hour and at room temperature for 30 min. The reaction was quenched by slow addition of saturated $Na_2CO_3$ solution at 0 °C and dilution with EtOAc (10 mL). The layers were separated and the aqueous phase was extracted with EtOAc (3 x 10 mL). The combined organic layers were washed with brine (20 mL), dried over $MgSO_4$ and concentrated in vacuo to afford the product (21.0 mg, 48%). **$^{1}$H-NMR** (500 MHz, DMSO): δ 7.01 (d, *J* = 8.8 Hz, 2H), 6.87 (d, *J* = 8.8 Hz, 2H), 6.49 (d, *J* = 8.7 Hz, 1H), 6.04 (s, 1H), 5.25 (d, *J* = 15.5 Hz, 1H), 5.08 (d, *J* = 15.5 Hz, 1H), 4.13 (ddd, *J* = 8.7, 6.9, 2.7 Hz, 1H), 3.72 (s, 3H), 3.05 (dd, *J* = 14.2, 6.9 Hz, 1H), 3.01-2.88 (m, 5H), 2.23 (s, 3H), 1.87-1.72 (m, 2H), 1.64-1.57 (m, 1H), 1.47-1.41 (m, 1H). **$^{13}$C-NMR** (126 MHz, DMSO): δ 211.6, 164.8, 160.4, 158.8, 151.2, 129.6, 128.1, 114.6, 105.9, 104.9, 94.7, 80.3, 55.5, 45.4, 40.5, 39.5, 34.6, 33.5, 30.4,

**(±) *Tert*-butyl-7'-methyl-3',5'-dioxo-6'-(thiophen-2-ylmethyl)-3',4',4a',5',6',9a'-hexahydrospiro[piperidine-4,2'-pyrano[3',2':4,5]furo[3,2-c]pyridine]-1-carboxylate (14dg)**

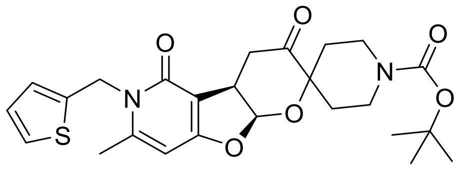

According to GP7, **8d** (97.6 mg, 0.30 mmol) was reacted with **5j** (66.4 mg, 0.30 mmol). Purification by MPLC (cyclohexane/EtOAc 1:0 to 0:1) afforded the product (92.5 mg, 64%). **$^{1}$H-NMR** (500 MHz, $CD_2Cl_2$): δ 7.25-7.20 (m, 1H), 6.95-6.92 (m, 2H), 6.37 (d, *J* = 8.4 Hz, 1H), 5.80 (s, 1H), 5.47 (d, *J* = 15.5 Hz, 1H), 5.18 (d, *J* = 15.5 Hz, 1H), 4.11 (ddd, *J* = 8.4, 6.7, 2.6 Hz, 1H), 3.95-3.78 (m, 2H), 3.24-3.14 (m, 2H), 3.13 (dd, *J* = 14.3, 2.6 Hz, 1H), 2.91 (dd, *J* = 14.3, 6.7 Hz, 1H), 2.38 (s, 3H), 1.75-1.65 (m, 2H), 1.63-1.59 (m 2H), 1.42 (s, 9H). **$^{13}$C-NMR** (126 MHz, $CD_2Cl_2$): δ 211.4, 165.5, 160.6, 154.8, 150.2, 139.8, 126.9, 126.6, 125.9, 106.2, 105.2, 95.2, 81.9, 79.6, 42.5, 40.2, 39.5, 35.0, 34.8, 31.9, 28.5, 21.4. **HRMS**-ESI (m/z): $[M + H]^+$ calculated for $C_{25}H_{31}N_2O_6S^+$, 487.1897; found, 487.1894.

**(±) 7'-methyl-6'-(thiophen-2-ylmethyl)-4a',9a'-dihydrospiro[piperidine-4,2'-pyrano[3',2':4,5]furo[3,2-c]pyridine]-3',5'(4'H,6'H)-dione (14eg)**

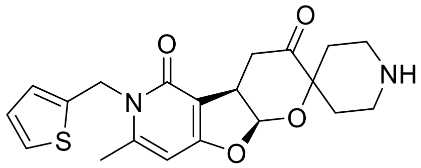

**14dg** (80.0 mg, 0.16 mmol) was dissolved in DCM (3 mL) and cooled to 0 °C. TFA (0.13 mL, 10 equiv) was added slowly and the mixture was stirred at 0 °C for 1 hour and at room temperature for 30 min. The reaction was quenched by slow addition of saturated $Na_2CO_3$ solution at 0 °C and dilution with EtOAc (10 mL). The layers were separated and the aqueous phase was extracted with EtOAc (3 x 10 mL). The combined organic layers were washed with brine (20 mL), dried over $MgSO_4$ and concentrated in vacuo to afford the product (51.0 mg, 80%). **$^{1}$H-NMR** (600 MHz, DMSO): δ 8.74 (s, 1H), 8.48 (s, 1H), 7.43 (dd, *J* = 5.1, 1.2 Hz, 1H), 7.02 (dd, *J* = 3.5, 1.2 Hz, 1H), 6.97 (dd, *J* = 5.1, 3.5 Hz, 1H), 6.50 (d, *J* = 8.3 Hz, 1H), 6.05 (s, 1H), 5.40 (d, *J* = 15.6 Hz, 1H), 5.27 (d, *J* = 15.6 Hz, 1H), 4.20-4.14 (m, 1H), 3.28-3.19 (m, 2H), 3.16-3.01 (m, 3H), 2.95 (dd, *J* = 14.4, 2.7 Hz, 1H), 2.39 (s, 3H), 2.01 (dq, *J* = 14.4, 3.1 Hz, 1H), 1.90 (ddd, *J* = 14.6, 12.5, 4.2 Hz, 1H), 1.76 (ddd, *J* = 14.2, 12.4, 4.5 Hz, 1H), 1.53 (dq, *J* = 14.7, 3.0 Hz, 1H). **$^{13}$C-NMR** (151 MHz, DMSO): δ 210.9, 164.8, 160.0, 150.9, 139.9, 127.1, 127.0, 126.6, 105.8, 104.7, 94.7, 78.6, 41.9, 39.3, 39.1, 34.8, 31.3, 28.8, 20.9. **HRMS**-ESI (m/z): $[M + H]^+$ calculated for $C_{20}H_{23}N_2O_4S^+$, 387.1373; found, 387.1375.

**(±) *Tert*-butyl-7'-methyl-3',5'-dioxo-6'-(pyridin-2-ylmethyl)-3',4',4a',5',6',9a'-hexahydrospiro[piperidine-4,2'-pyrano[3',2':4,5]furo[3,2-c]pyridine]-1-carboxylate (14dh)**

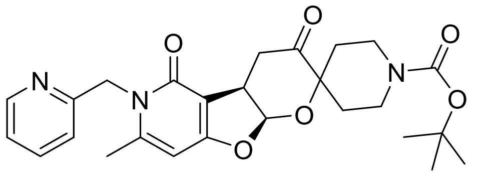

According to GP7, **8d** (97.6 mg, 0.30 mmol) was reacted with **5m** (64.9 mg, 0.30 mmol). Purification by MPLC (cyclohexane/EtOAc 4:1 to 0:1) afforded the product (79.2 mg, 55%). **$^{1}$H-NMR** (700 MHz, $CD_2Cl_2$): δ 8.48 (dd, *J* = 4.9, 1.8 Hz, 1H), 7.65 (td, *J* = 7.7, 1.8 Hz, 1H), 7.18 (dd, *J* = 7.7, 4.9 Hz, 1H), 7.10 (d, *J* = 7.7 Hz, 1H), 6.38 (d, *J* = 8.6 Hz, 1H), 5.85 (s, 1H), 5.47 (d, *J* = 16.0 Hz, 1H), 5.15 (d, *J* = 16.0 Hz, 1H), 4.09 (ddd, *J* = 8.6, 6.7, 2.7 Hz, 1H), 3.94-3.77 (m, 2H), 3.25-3.10 (m, 2H), 3.09 (dd, *J* = 14.3, 2.7 Hz, 1H), 2.89 (dd, *J* = 14.3, 6.7 Hz, 1H), 2.32 (s, 3H), 1.77-1.57 (m, 3H), 1.53-1.48 (m, 1H), 1.42 (s, 9H). **$^{13}$C-NMR** (176 MHz, $CD_2Cl_2$): δ 211.5, 165.6, 160.8, 157.0, 154.8, 151.2, 149.7, 137.2, 122.8, 121.8, 106.0, 105.2, 95.0, 81.9, 79.6, 48.8, 40.3, 39.7, 38.7, 35.0, 34.8, 31.9, 28.5, 21.9. **HRMS**-ESI (m/z): $[M + H]^+$ calculated for $C_{26}H_{32}N_3O_6^+$, 482.2286; found, 482.2278.

**(±) 7'-methyl-3',5'-dioxo-6'-(pyridin-2-ylmethyl)-3',4',4a',5',6',9a'-hexahydrospiro[piperidine-4,2'-pyrano[3',2':4,5]furo[3,2-c]pyridin]-1-ium triflate (14eh)**

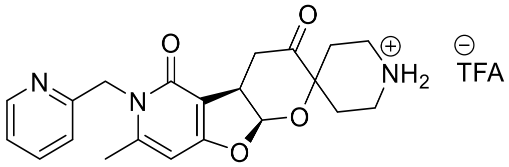


**14dh** (79.4 mg, 0.14 mmol) was dissolved in DCM (3 mL) and cooled to 0 °C. HCl in dioxane (0.13 mL, 4 M, 10 equiv) was added slowly and the mixture was stirred at 0 °C for 1 hour and at room temperature for 30 min. The reaction was quenched by slow addition of saturated $Na_2CO_3$ solution at 0 °C and dilution with EtOAc (10 mL). The layers were separated and the aqueous phase was extracted with EtOAc (3 x 10 mL). The combined organic layers were washed with brine (20 mL), dried over $MgSO_4$ and concentrated in vacuo. Purification by prep. HPLC afforded the product (41.8 mg, 66%). **$^{1}$H-NMR** (700 MHz, DMSO): δ 8.72 (s, 1H), 8.53 (s, 1H), 8.48 (d, *J* = 4.9 Hz, 1H), 7.77 (td, *J* = 7.7, 1.9 Hz, 1H), 7.30 (dd, *J* = 7.7, 4.9 Hz, 1H), 7.13 (d, *J* = 7.7 Hz, 1H), 6.49 (d, *J* = 8.3 Hz, 1H), 6.09 (s, 1H), 5.42 (d, *J* = 16.3 Hz, 1H), 5.16 (d, *J* = 16.3 Hz, 1H), 4.14-4.11 (m, 1H), 3.28-3.19 (m, 2H), 3.13-3.04 (m, 3H), 2.88 (dd, *J* = 14.4, 2.8 Hz, 1H), 2.30 (s, 3H), 2.00 (dq, *J* = 14.6, 3.3 Hz, 1H), 1.93 (ddd, *J* = 16.7, 13.4, 3.7 Hz, 1H), 1.77 (ddd, *J* = 16.5, 12.9, 4.2 Hz, 1H), 1.59-1.55 (m, 1H). **$^{13}$C-NMR** (176 MHz, DMSO): δ 210.6, 164.4, 159.7, 156.1, 151.3, 148.9, 137.2, 122.5, 121.2, 105.1, 104.2, 94.0, 78.1, 47.5, 40.0, 38.8, 38.6, 34.3, 30.7,

28.4, 20.9. **HRMS**-ESI (m/z): [M + H]$^+$ calculated for $C_{21}H_{24}N_3O_4^+$, 382.1761; found, 382.1762.

**(±) *Tert*-butyl-7'-methyl-3',5'-dioxo-6'-(pyridin-4-ylmethyl)-3',4',4a',5',6',9a'-hexahydrospiro[piperidine-4,2'-pyrano[3',2':4,5]furo[3,2-c]pyridine]-1-carboxylate (14di)**

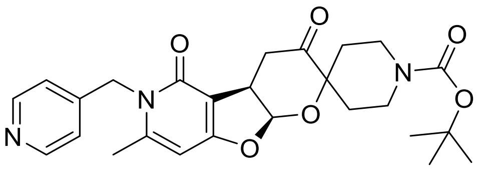

According to GP7, **8d** (97.6 mg, 0.30 mmol) was reacted with **5i** (64.8 mg, 0.30 mmol). Purification by MPLC (cyclohexane/EtOAc 1:0 to 0:1) afforded the product (95.0 mg, 66%). **$^1$H-NMR** (700 MHz, $CD_2Cl_2$): δ 8.74 (d, *J* = 6.4 Hz, 2H), 7.45 (d, *J* = 6.4 Hz, 2H), 6.45 (d, *J* = 8.2 Hz, 1H), 5.52 (d, *J* = 17.2 Hz, 1H), 5.30 (d, *J* = 17.2 Hz, 1H), 4.15 (ddd, *J* = 8.2, 6.6, 2.6 Hz, 1H), 3.93-3.81 (m, 2H), 3.25-3.13 (m, 2H), 3.06 (dd, *J* = 14.2, 2.6 Hz, 1H), 2.94 (dd, *J* = 14.2, 6.6 Hz, 1H), 2.22 (s, 3H), 1.77 (ddd, *J* = 14.1, 11.9, 4.5 Hz, 1H), 1.72 (d, *J* = 13.2 Hz, 1H), 1.60 (ddd, *J* = 13.4, 11.8, 4.7 Hz, 1H), 1.51-1.47 (m, 1H), 1.43 (s, 9H). **$^{13}$C-NMR** (176 MHz, $CD_2Cl_2$): δ 210.8, 165.8, 160.2, 154.7, 154.4, 149.3, 143.5, 123.7, 106.2, 105.2, 96.1, 81.7, 79.3, 46.0, 39.9, 38.4, 34.7, 34.3, 31.5, 28.1, 21.2. **HRMS**-ESI (m/z): [M + H]$^+$ calculated for $C_{26}H_{32}N_3O_6^+$, 482.2286; found, 482.2274.

**(±) 7'-methyl-6'-(pyridin-4-ylmethyl)-4a',9a'-dihydrospiro[piperidine-4,2'-pyrano[3',2':4,5]furo[3,2-c]pyridine]-3',5'(4'H,6'H)-dione (14ei)**

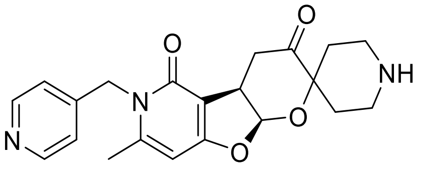

**14di** (87.1 mg, 0.18 mmol) was dissolved in DCM (2 mL) and cooled to 0 °C. TFA (0.14 mL, 10 equiv) was added slowly and the mixture was stirred at 0 °C for 1 hour and at room temperature for 30 min. The reaction was quenched by slow addition of saturated $Na_2CO_3$ solution at 0 °C and dilution with EtOAc (10 mL). The layers were separated and the aqueous phase was extracted with EtOAc (3 x 10 mL). The combined organic layers were washed with brine (20 mL), dried over $MgSO_4$ and concentrated in vacuo to afford the product (50.0 mg, 72%). **$^1$H-NMR** (500 MHz, DMSO): δ 8.51-8.49 (m, 2H), 7.02-6.99 (m, 2H), 6.49 (d, *J* = 8.6 Hz, 1H), 6.12 (s, 1H), 5.34 (d, *J* = 16.9 Hz, 1H), 5.17 (d, *J* = 17.0 Hz, 1H), 4.11 (ddd, *J* = 8.6, 7.0, 2.7 Hz, 1H), 2.99 (dd, *J* = 14.0, 6.7 Hz, 1H), 2.86-2.70 (m, 5H), 2.20 (s, 3H), 1.70-1.58 (m, 2H), 1.44 (td, *J* = 12.8, 12.3, 4.6 Hz, 1H), 1.38-1.32 (m, 1H). **$^{13}$C-NMR** (126 MHz, DMSO): δ 211.6, 164.8, 159.8, 150.4, 149.9, 146.4, 121.1,

105.5, 104.6, 94.6, 81.6, 44.8, 40.8, 40.5, 39.0, 34.8, 34.2, 31.9, 20.5. **HRMS**-ESI (m/z): [M + H]$^+$ calculated for $C_{21}H_{24}N_3O_4^+$, 382.1761; found, 382.1761.

**(±) *Tert*-butyl-6'-((2-chloropyridin-4-yl)methyl)-7'-methyl-3',5'-dioxo-3',4',4a',5',6',9a'-hexahydrospiro[piperidine-4,2'-pyrano[3',2':4,5]furo[3,2-c]pyridine]-1-carboxylate (14dj)**

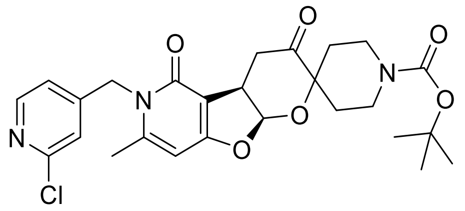

According to GP7, **8d** (97.6 mg, 0.30 mmol) was reacted with **5r** (75.2 mg, 0.30 mmol). Purification by MPLC (cyclohexane/EtOAc 1:0 to 0:1) afforded the product (94.7 mg, 66%). **$^1$H-NMR** (500 MHz, $CD_2Cl_2$): δ 8.28 (d, *J* = 5.2 Hz, 1H), 6.99 (s, 1H), 6.94 (dd, *J* = 5.2, 1.6 Hz, 1H), 6.42 (d, *J* = 8.7 Hz, 1H), 5.89 (s, 1H), 5.38 (d, *J* = 16.4 Hz, 1H), 5.07 (d, *J* = 16.4 Hz, 1H), 4.13 (ddd, *J* = 8.7, 6.6, 2.6 Hz, 1H), 3.94-3.80 (m, 2H), 3.26-3.09 (m, 2H), 3.08 (dd, *J* = 14.2, 2.6 Hz, 1H), 2.92 (dd, *J* = 14.2, 6.6 Hz, 1H), 2.19 (s, 3H), 1.78-1.66 (m, 2H), 1.63-1.56 (m, 1H), 1.51-1.45 (m, 1H), 1.41 (s, 9H). **$^{13}$C-NMR** (126 MHz, $CD_2Cl_2$): δ 211.3, 165.8, 160.6, 154.7, 152.4, 150.4, 150.1, 149.9, 121.9, 120.5, 106.3, 105.3, 95.8, 81.9, 79.6, 45.5, 40.2, 39.3, 38.6, 34.9, 34.7, 31.8, 28.4, 21.5. **HRMS**-ESI (m/z): [M + H]$^+$ calculated for $C_{26}H_{31}ClN_3O_6^+$, 516.1896; found, 516.1882.

**(±) 6'-((2-chloropyridin-4-yl)methyl)-7'-methyl-4a',9a'-dihydrospiro[piperidine-4,2'-pyrano[3',2':4,5]furo[3,2-c]pyridine]-3',5'(4'H,6'H)-dione (14ej)**

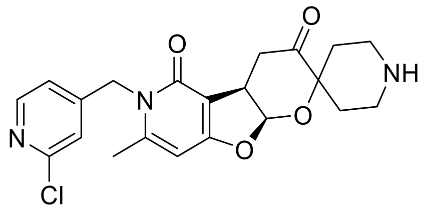

**14dj** (92.1 mg, 0.18 mmol) was dissolved in DCM (3 mL) and cooled to 0 °C. TFA (0.14 mL, 10 equiv) was added slowly and the mixture was stirred at 0 °C for 1 hour and at room temperature for 30 min. The reaction was quenched by slow addition of saturated $Na_2CO_3$ solution at 0 °C and dilution with EtOAc (10 mL). The layers were separated and the aqueous phase was extracted with EtOAc (3 x 10 mL). The combined organic layers were washed with brine (20 mL), dried over $MgSO_4$ and concentrated in vacuo to afford the product (58.7 mg, 79%). **$^1$H-NMR** (700 MHz, DMSO): δ 8.35 (d, *J* = 5.2 Hz, 1H), 7.08 (s, 1H), 7.04 (d, *J* = 5.2 Hz, 1H), 6.49 (d, *J* = 8.3 Hz, 1H), 6.14 (s, 1H), 5.31 (d, *J* = 17.1 Hz, 1H), 5.22 (d, *J* = 17.1 Hz, 1H), 4.14-4.11 (m, 1H), 3.01 (dd, *J* = 14.1, 6.7 Hz, 1H), 2.88-2.80 (m, 5H), 2.21 (s, 1H), 1.75-1.65 (m, 2H), 1.53-1.48 (m, 1H), 1.41-1.37 (m, 1H). **$^{13}$C-NMR** (176 MHz, DMSO): δ 211.3, 164.9, 160.0, 150.7, 150.6, 150.1, 121.4, 120.6, 105.4, 104.6, 94.8, 81.0, 44.6, 40.4, 40.2, 39.0, 34.1, 34.0, 31.2, 20.5. **HRMS**-ESI (m/z): [M + H]$^+$ calculated for $C_{21}H_{23}ClN_3O_4^+$, 416.1372; found, 416.1367.

**(±) *Tert*-butyl-6'-(2-(5-methoxy-1H-indol-3-yl)ethyl)-7'-methyl-3',5'-dioxo-3',4',4a',5',6',9a'-hexahydrospiro[piperidine-4,2'-pyrano[3',2':4,5]furo[3,2-c]pyridine]-1-carboxylate (14dk)**

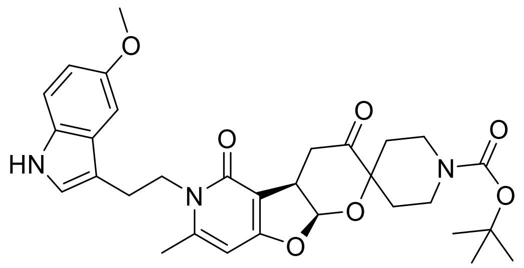

According to GP7, **8d** (97.6 mg, 0.30 mmol) was reacted with **5p** (98.5 mg, 0.30 mmol). Purification by MPLC (cyclohexane/EtOAc 1:0 to 0:1) afforded the product (89.0 mg, 52%). **$^1$H-NMR** (700 MHz, $CD_2Cl_2$): δ 8.18 (s, 1H), 7.26 (d, *J* = 8.7 Hz, 1H), 7.12 (d, *J* = 2.5 Hz, 1H), 6.91 (s, 1H), 6.82 (dd, *J* = 8.7, 2.5 Hz, 1H), 6.37 (d, *J* = 8.3 Hz, 1H), 5.71 (s, 1H), 4.32-4.25 (m, 1H), 4.14-4.08 (m, 2H), 3.93-3.80 (m, 5H), 3.24-3.08 (m, 4H), 3.05-3.00 (m, 1H), 2.92 (dd, *J* = 14.3, 6.7 Hz, 1H), 2.11 (s, 3H), 1.77-1.67 (m, 2H), 1.64-1.59 (m, 1H), 1.52-1.47 (m, 1H), 1.43 (s, 9H). **$^{13}$C-NMR** (176 MHz, $CD_2Cl_2$): δ 211.7, 165.2, 160.8, 154.9, 154.6, 150.4, 131.8, 128.2, 123.6, 112.4, 112.3, 106.3, 105.1, 100.9, 94.6, 81.8, 79.6, 56.1, 45.1, 40.4, 39.7, 38.8, 35.0, 34.9, 31.9, 28.5, 24.6, 21.5. **HRMS**-ESI (m/z): $[M + H]^+$ calculated for $C_{31}H_{38}N_3O_7^+$, 564.2704; found, 564.2700.

**(±) 6'-(2-(5-methoxy-1H-indol-3-yl)ethyl)-7'-methyl-4a',9a'-dihydrospiro[piperidine-4,2'-pyrano[3',2':4,5]furo[3,2-c]pyridine]-3',5'(4'H,6'H)-dione (14ek)**

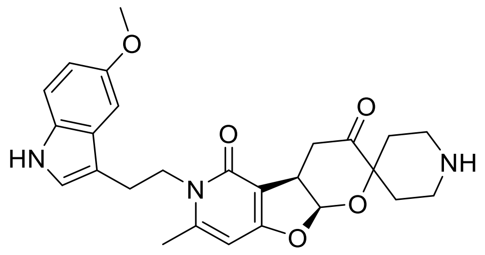

**14dk** (80.0 mg, 0.14 mmol) was dissolved in DCM (3 mL) and cooled to 0 °C. TFA (0.11 mL, 10 equiv) was added slowly and the mixture was stirred at 0 °C for 1 hour and at room temperature for 30 min. The reaction was quenched by slow addition of saturated $Na_2CO_3$ solution at 0 °C and dilution with EtOAc (10 mL). The layers were separated and the aqueous phase was extracted with EtOAc (3 x 10 mL). The combined organic layers were washed with brine (20 mL), dried over $MgSO_4$ and concentrated in vacuo to afford the product (40.0 mg, 61%). **$^1$H-NMR** (700 MHz, DMSO): δ 10.73 (s, 1H), 7.24 (dd, *J* = 9.0, 3.5 Hz, 1H), 7.12 (s, 1H), 7.02 (s, 1H), 6.73 (dt, *J* = 9.0, 2.0 Hz, 1H), 6.45 (d, *J* = 8.2 Hz, 1H), 5.94 (s, 1H), 4.24-4.16 (m, 1H), 4.13-4.07 (m, 1H), 4.06-3.98 (m, 1H), 3.76 (s, 1H), 3.01 (dd, *J* = 14.2, 6.7 Hz, 1H), 2.97-2.79 (m, 7H), 2.18 (s, 3H), 1.74-1.65 (m, 2H), 1.57-1.48 (m, 1H), 1.42-1.36 (m, 1H). **$^{13}$C-NMR** (176 MHz, DMSO): δ 212.1, 164.7, 160.2, 153.6, 150.8, 131.8, 127.9, 124.2, 112.5, 111.6, 111.0, 105.9, 104.7, 100.7, 94.2, 81.4, 55.8, 44.7, 40.8, 40.6, 39.5, 34.7, 34.4, 31.7, 24.3, 20.8. **HRMS**-ESI (m/z): $[M + H]^+$ calculated for $C_{26}H_{30}N_3O_5^+$, 464.2180; found, 464.2172.

**(±) *Tert*-butyl-6',8'-dioxo-5',6',6b',7',8',10a'-hexahydrospiro[piperidine-4,9'-pyrano[3',2':4,5]furo[3,2-c]quinoline]-1-carboxylate (14dl)**

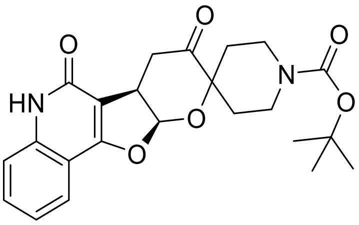


According to GP7, **8d** (97.6 mg, 0.30 mmol) was reacted with 4-hydroxyquinolin-2(1H)-one (48.3 mg, 0.30 mmol). Purification by MPLC (cyclohexane/EtOAc 1:0 to 0:1) afforded the product (24.5 mg, 20%). **$^{1}$H-NMR** (500 MHz, $CD_2Cl_2$): δ 11.75 (s, 1H), 7.68 (dd, *J* = 7.8, 1.5 Hz, 1H), 7.57 (ddd, *J* = 8.3, 7.2, 1.5 Hz, 1H), 7.41 (d, *J* = 8.3 Hz, 1H), 7.24 (td, *J* = 7.8, 7.2, 1.0 Hz, 1H), 6.61 (d, *J* = 8.7 Hz, 1H), 4.29 (ddd, *J* = 8.7, 6.9, 2.7 Hz, 1H), 3.99-3.75 (m, 2H), 3.31-3.17 (m, 3H), 3.04 (dd, *J* = 14.6, 6.9 Hz, 1H), 1.78-1.59 (m, 4H), 1.40 (s, 9H). **$^{13}$C-NMR** (126 MHz, $CD_2Cl_2$): δ 210.6, 162.7, 161.9, 154.4, 139.9, 131.8, 122.5, 122.4, 116.2, 110.8, 108.2, 105.3, 81.8, 79.3, 40.0, 39.1, 38.2, 34.8, 34.5, 31.7, 28.1. **HRMS**-ESI (m/z): $[M + H]^+$ calculated for $C_{23}H_{27}N_2O_6^+$, 427.1864; found, 427.1863.

**(±) 6b',10a'-dihydrospiro[piperidine-4,9'-pyrano[3',2':4,5]furo[3,2-c]quinoline]-6',8'(5'H,7'H)-dione (14el)**

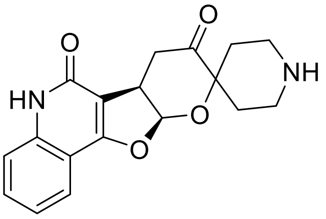


**14dl** (22.0 mg, 0.05 mmol) was dissolved in DCM (3 mL) and cooled to 0 °C. HCl in dioxane (0.13 mL, 4 M, 10 equiv) was added slowly and the mixture was stirred at 0 °C for 1 hour and at room temperature for 30 min. The reaction was quenched by slow addition of saturated $Na_2CO_3$ solution at 0 °C and dilution with EtOAc (10 mL). The layers were separated and the aqueous phase was extracted with EtOAc (3 x 10 mL). The combined organic layers were washed with brine (20 mL), dried over $MgSO_4$ and concentrated in vacuo to afford the product (12.3 mg, 73%). **$^{1}$H-NMR** (500 MHz, DMSO): δ 11.55 (s, 1H), 7.60-7.53 (m, 2H), 7.35 (d, *J* = 8.3 Hz, 1H), 7.23-7.19 (m, 1H), 6.66 (d, *J* = 8.5 Hz, 1H), 4.23 (ddd, *J* = 8.5, 6.9, 2.8 Hz, 1H), 3.10 (dd, *J* = 14.3, 6.9 Hz, 1H), 2.96-2.78 (m, 5H), 1.81-1.76 (m, 1H), 1.65-1.50 (m, 2H), 1.30-1.25 (m, 1H). **$^{13}$C-NMR** (126 MHz, DMSO): δ 211.3, 160.9, 159.9, 139.9, 131.6, 122.0, 121.9, 115.7, 110.0, 108.6, 104.8, 80.9, 40.1, 39.6, 34.5, 33.6. 31.0. **HRMS**-ESI (m/z): $[M + H]^+$ calculated for $C_{18}H_{19}N_2O_4^+$, 327.1339; found, 327.1343.

**(±) 5'-methyl-6',8'-dioxo-5',6',6b',7',8',10a'-hexahydrospiro[piperidine-4,9'-pyrano[3',2':4,5]furo[3,2-c]quinolin]-1-ium triflate (14em)**

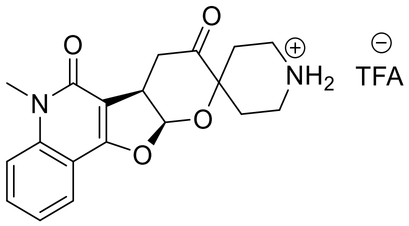

According to GP7, **8d** (97.6 mg, 0.30 mmol) was reacted with 4-hydroxy-1-methylquinolin-2(1H)-one (66.4 mg, 0.30 mmol). Purification by MPLC (cyclohexane/EtOAc 1:0 to 0:1) afforded the product which was directly dissolved in DCM (3 mL) and cooled to 0 °C. TFA (0.23 mL, 10 equiv) was added slowly and the mixture was stirred at 0 °C for 1 hour and at room temperature for 30 min. The reaction was quenched by slow addition of saturated $Na_2CO_3$ solution at 0 °C and dilution with EtOAc (10 mL). The layers were separated and the aqueous phase was extracted with EtOAc (3 x 10 mL). The combined organic layers were washed with brine (20 mL), dried over $MgSO_4$ and concentrated in vacuo. The crude was purified by MPLC (cyclohexane/EtOAc 1:0 to 0:1) and repurified by prep. HPLC to afford the product (22.1 mg, 17% over two steps). **$^{1}$H-NMR** (600 MHz, DMSO): δ 8.78 (s, 1H), 8.48 (s, 1H), 7.74-7.68 (m, 2H), 7.59 (d, *J* = 8.6 Hz, 1H), 7.33 (t, *J* = 7.5 Hz, 1H), 6.71 (d, *J* = 8.5 Hz, 1H), 4.32 (ddd, *J* = 8.5, 7.1, 2.8 Hz, 1H), 3.59 (s, 3H), 3.27-3.11 (m, 5H), 2.99 (dd, *J* = 14.6, 2.8 Hz, 1H), 2.04 (dq, *J* = 14.3, 3.1 Hz, 1H), 1.85 (ddd, *J* = 14.6, 12.1, 4.5 Hz, 1H), 1.78 (ddd, *J* = 14.2, 12.3, 4.4 Hz, 1H), 1.44 (dq, *J* = 14.7, 3.2 Hz, 1H). **$^{13}$C-NMR** (151 MHz, DMSO): δ 210.5, 159.6, 159.0, 140.5, 132.2, 122.5, 122.3, 115.6, 110.6, 108.0, 104.7, 78.4, 40.1, 38.9, 38.6, 34.6, 30.8, 28.7, 28.5. **HRMS**-ESI (m/z): $[M + H]^+$ calculated for $C_{19}H_{21}N_2O_4^+$, 341.1496; found, 341.1494.

**(±) *Tert*-butyl-6',8'-dioxo-6b',7',8',10a'-tetrahydro-6'H-spiro[piperidine-4,9'-pyrano[3',2':4,5]furo[3,2-c]chromene]-1-carboxylate (14dn)**

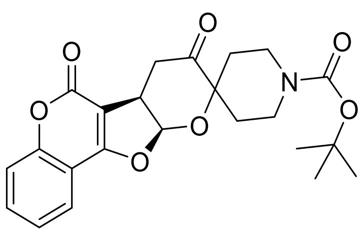

According to GP7, **8d** (116.9 mg, 0.36 mmol) was reacted with 4-hydroxy-2H-chromen-2-one (1H)-one (58.3 mg, 0.36 mmol). Purification by MPLC (cyclohexane/EtOAc 1:0 to 0:1) afforded the product (134.3 mg, 87%). **$^{1}$H-NMR** (500 MHz, $CD_2Cl_2$): δ 7.66-7.63 (m, 1H), 7.63-7.61 (m, 1H), 7.38 (d, *J* = 8.4 Hz, 1H), 7.33 (td, *J* = 7.6, 1.0 Hz, 1H), 6.65 (d, *J* = 8.7 Hz, 1H), 4.20 (ddd, *J* = 8.7, 6.6, 2.7 Hz, 1H), 3.95-3.76 (m, 2H), 3.30-3.14 (m, 2H), 3.08-2.94 (m, 2H), 1.77-1.61 (m, 3H), 1.41 (s, 9H), 1.39-1.36 (m, 1H). **$^{13}$C-NMR** (126 MHz, $CD_2Cl_2$): δ 210.0, 165.3, 159.1, 155.6, 154.7, 133.7, 124.8, 123.2, 117.5, 112.0, 106.5, 103.1, 82.6, 79.7, 39.9, 38.6, 34.9, 32.1, 28.5. **HRMS**-ESI (m/z): $[M + H]^+$ calculated for $C_{23}H_{26}NO_7^+$, 428.1704; found, 428.1703.

**(±) 6b',10a'-dihydro-6'H-spiro[piperidine-4,9'-pyrano[3',2':4,5]furo[3,2-c]chromene]-6',8'(7'H)-dione (14en)**

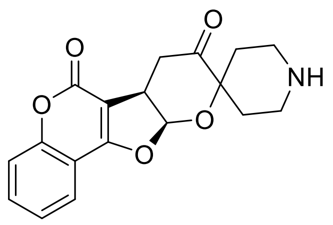

**14dn** (130.0 mg, 0.30 mmol) was dissolved in DCM (3 mL) and cooled to 0 °C. TFA (0.23 mL, 10 equiv) was added slowly and the mixture was stirred at 0 °C for 1 hour and at room temperature for 30 min. The reaction was quenched by slow addition of saturated $Na_2CO_3$ solution at 0 °C and dilution with EtOAc (10 mL). The layers were separated and the aqueous phase was extracted with EtOAc (3 x 10 mL). The combined organic layers were washed with brine (20 mL), dried over $MgSO_4$ and concentrated in vacuo to afford the product (74.5 mg, 75%). **$^1$H-NMR** (700 MHz, DMSO): δ 7.72 (ddd, *J* = 8.6, 7.5, 1.7 Hz, 1H), 7.67 (dd, *J* = 7.5, 1.7 Hz, 1H), 7.48 (dd, *J* = 8.6, 1.0 Hz, 1H), 7.41 (td, *J* = 7.5, 1.0 Hz, 1H), 6.78 (d, *J* = 8.4 Hz, 1H), 4.26 (ddd, *J* = 8.4, 6.9, 2.9 Hz, 1H), 3.14 (dd, *J* = 14.5, 6.9 Hz, 1H), 2.85 (tt, *J* = 11.9, 3.4 Hz, 2H), 2.80-2.75 (m, 2H), 2.72 (dt, *J* = 12.6, 4.0 Hz, 1H), 1.74 (dq, *J* = 13.3, 2.9 Hz, 1H), 1.60 (ddd, *J* = 13.7, 11.5, 4.3 Hz, 1H), 1.51 (ddd, *J* = 13.2, 11.4, 4.5 Hz, 1H), 1.23 (dd, *J* = 13.8, 2.8 Hz, 1H). **$^{13}$C-NMR** (176 MHz, DMSO): δ 210.6, 164.2, 158.2, 154.4, 133.6, 124.8, 122.7, 116.9, 111.2, 106.2, 102.8, 82.1, 40.5, 40.2, 38.6, 34.4, 34.2, 31.8. **HRMS**-ESI (m/z): $[M + H]^+$ calculated for $C_{18}H_{18}NO_5^+$, 328.1180; found, 328.1180.

**(±) *Tert*-butyl 7-methyl-3,5-dioxo-3,4,4a,9a-tetrahydro-5H-spiro[furo[2,3-b:4,5-c']dipyran-2,4'-piperidine]-1'-carboxylate (14do)**

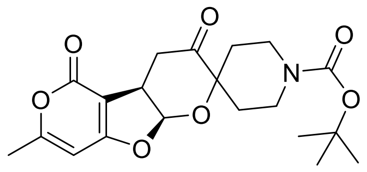

According to GP7, **8d** (116.9 mg, 0.36 mmol) was reacted with 4-hydroxy-6-methyl-2H-pyran-2-one (1H)-one (45.3 mg, 0.36 mmol). Purification by MPLC (cyclohexane/EtOAc 1:0 to 0:1) afforded the product (118.9 mg, 85%). **$^1$H-NMR** (500 MHz, $CD_2Cl_2$): δ δ 6.44 (d, *J* = 8.5 Hz, 1H), 5.92 (s, 1H), 4.04-4.00 (m, 1H), 3.93-3.80 (m, 2H), 3.23-3.08 (m, 2H), 2.96-2.88 (m, 2H), 2.24 (s, 3H), 1.76 (ddd, *J* = 14.2, 11.9, 4.5 Hz, 1H), 1.72-1.66 (m, 1H), 1.62 (ddd, *J* = 13.5, 11.6, 4.7 Hz, 1H), 1.46-1.43 (m, 1H), 1.42 (s, 9H). **$^{13}$C-NMR** (126 MHz, $CD_2Cl_2$): δ 210.3, 169.9, 167.8, 160.6, 154.7, 106.0, 99.9, 95.3, 82.3, 79.7, 39.5, 38.8, 35.0, 34.8, 31.9, 28.5, 20.8. **HRMS**-ESI (m/z): $[M + H]^+$ calculated for $C_{20}H_{26}NO_7^+$, 392.1704; found, 392.1704.

**(±) 7-methyl-4a,9a-dihydro-5H-spiro[furo[2,3-b:4,5-c']dipyran-2,4'-piperidine]-3,5(4H)-dione (14eo)**

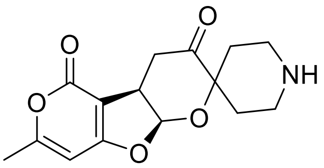

**14do** (110.0 mg, 0.28 mmol) was dissolved in DCM (3 mL) and cooled to 0 °C. TFA (0.21 mL, 10 equiv) was added slowly and the mixture was stirred at 0 °C for 1 hour and at room temperature for 30 min. The reaction was quenched by slow addition of saturated $Na_2CO_3$ solution at 0 °C and dilution with EtOAc (10 mL). The layers were separated and the aqueous phase was extracted with EtOAc (3 x 10 mL). The combined organic layers were washed with brine (20 mL), dried over $MgSO_4$ and concentrated in vacuo to afford the product (62.3 mg, 76%). **$^1$H-NMR** (700 MHz, DMSO): δ 6.56 (d, *J* = 8.4 Hz, 1H), 6.34 (s, 1H), 4.08-4.05 (m, 1H), 3.05 (dd, *J* = 14.4, 6.8 Hz, 1H), 2.85-2.75 (m, 4H), 2.66 (dd, *J* = 14.4, 2.7 Hz, 1H), 2.22 (s, 3H), 1.71 (dq, *J* = 13.5, 3.0 Hz, 1H), 1.66 (ddd, *J* = 13.8, 8.8, 7.1 Hz, 1H), 1.49 (ddd, *J* = 13.3, 11.2, 4.8 Hz, 1H), 1.30 (dq, *J* = 13.7, 3.0 Hz, 1H). **$^{13}$C-NMR** (176 MHz, DMSO): δ 210.7, 169.2, 167.2, 159.7, 105.5, 99.3, 95.0, 81.6, 40.5, 40.2, 37.5, 34.3, 34.2, 31.4, 19.9. **HRMS**-ESI (m/z): $[M + H]^+$ calculated for $C_{15}H_{18}NO_5{}^+$, 292.1180; found, 292.1178.

**(±) 2,6,7-trimethyl-4a,9a-dihydro-2H-pyrano[3',2':4,5]furo[3,2-c]pyridin-5(6H)-one (15a)**

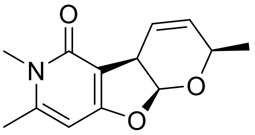

According to GP2, **7b**-*trans* (50.8 mg, 0.17 mmol) was reacted with **5b** (19.5 mg, 0.14 mmol) at 100 °C for 1 hour. After filtration over celite the crude was purified by FC (cyclohexane/EtOAc 1:0 to 0:1) to afford the product (19.4 mg, 59%). **$^1$H-NMR** (700 MHz, DMSO): δ δ 6.14 (d, *J* = 6.6 Hz, 1H), 6.10 (ddd, *J* = 10.3, 3.9, 2.0 Hz, 1H), 6.03 (s, 1H), 5.87 (ddd, *J* = 10.3, 2.8, 1.8 Hz, 1H), 4.34-4.30 (m, 1H), 3.66-3.63 (m, 1H), 2.32 (s, 3H), 1.16 (d, *J* = 6.8 Hz, 3H). **$^{13}$C-NMR** (176 MHz, DMSO): δ 163.9, 160.3, 149.8, 130.1, 121.6, 107.2, 104.5, 94.2, 66.9, 37.1, 29.9, 21.8, 20.9. **HRMS**-ESI (m/z): $[M + H]^+$ calculated for $C_{13}H_{16}NO_3{}^+$, 234.1125; found, 234.1124.

**(±) 2,6,7-trimethyl-3,4,4a,9a-tetrahydro-2H-pyrano[3',2':4,5]furo[3,2-c]pyridin-5(6H)-one (16a)**

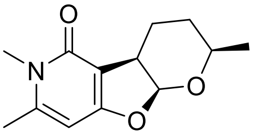

**15a** (19.4 mg, 0.08 mmol) was suspended with Pd/C (5 mg, 10 wt%) in toluene (2 mL) and was hydrogenated at 20 °C for 18 hours using a $H_2$-balloon. The catalyst was filtered off and the solvent removed in vacuo. The crude was purified by MPLC (EtOAc/MeOH 1:0 to 9:1 + 0.1% DIPEA) to afford the

product (11.7 mg, 62%). $^{1}$**H-NMR** (700 MHz, $CD_2Cl_2$): δ 5.92 (d, *J* = 6.5 Hz, 1H), 5.32 (s, 1H), 3.82-3.77 (m, 1H), 3.43 (s, 3H), 3.34 (ddd, *J* = 8.0, 6.5, 2.3 Hz, 1H), 2.39 (dddd, *J* = 13.7, 4.5, 3.5, 2.3 Hz, 1H), 2.32 (s, 3H), 1.78 (dddd, *J* = 13.7, 13.0, 6.3, 3.7 Hz, 1H), 1.63-1.58 (m, 1H), 1.24-1.21 (m, 1H), 1.17 (d, *J* = 6.3 Hz, 3H). $^{13}$**C-NMR** (176 MHz, $CD_2Cl_2$): δ 165.9, 161.9, 149.1, 106.9, 106.5, 95.2, 70.8, 38.7, 30.6, 27.7, 23.2, 21.9, 20.3. **HRMS**-ESI (m/z): $[M + H]^+$ calculated for $C_{13}H_{18}NO_3^+$, 236.1281; found, 236.1284.

**(±) 2,7-dimethyl-3,4,4a,9a-tetrahydro-2H-pyrano[3',2':4,5]furo[3,2-c]pyridin-5(6H)-one (16b)**

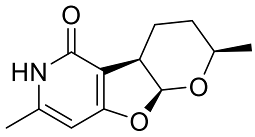


According to GP2, **7b**-*trans* (50.8 mg, 0.17 mmol) was reacted with **5a** (19.5 mg, 0.14 mmol) at 100 °C for 1 hour. After filtration over celite the crude was purified by FC (EtOAc/MeOH 1:0 to 9:1) and the product was directly suspended with Pd/C (5 mg, 10 wt%) in toluene (2 mL) and was hydrogenated at 20 °C for 18 hours using a $H_2$-balloon. The catalyst was filtered off and the solvent removed in vacuo. The crude was purified by MPLC (EtOAc/MeOH 1:0 to 9:1 + 0.1% DIPEA) to afford the product (8.00 mg, 18%). $^{1}$**H-NMR** (600 MHz, $CD_2Cl_2$): δ 11.52 (s, 1H), 5.95 (d, *J* = 6.5 Hz, 1H), 5.84 (s, 1H), 3.81 (ttd, *J* = 10.5, 6.7, 6.2, 3.5 Hz, 1H), 3.35 (td, *J* = 6.5, 2.9 Hz, 1H), 2.39 (ddd, *J* = 13.6, 6.3, 2.9 Hz, 1H), 2.27 (s, 3H), 1.80 (tdd, *J* = 13.6, 6.3, 3.7 Hz, 1H), 1.65-1.60 (m, 1H), 1.28-1.24 (m, 1H), 1.17 (d, *J* = 6.3 Hz, 3H). $^{13}$**C-NMR** (151 MHz, $CD_2Cl_2$): δ 168.8, 163.3, 147.9, 107.2, 106.6, 94.7, 70.8, 37.9, 27.6, 23.2, 20.5, 19.6. **HRMS**-ESI (m/z): $[M + H]^+$ calculated for $C_{12}H_{16}NO_3^+$, 222.1125; found, 222.1130.

**(±) 6-cyclobutyl-2,7-dimethyl-4a,9a-dihydro-2H-pyrano[3',2':4,5]furo[3,2-c]pyridin-5(6H)-one (15c)**

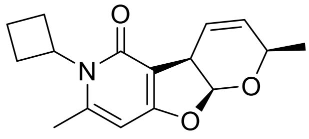


According to GP2, **7b**-*trans* (33.4 mg, 0.11 mmol) was reacted with **5e** (18.0 mg, 0.10 mmol) at 110 °C for 1 hour. The catalyst was filtered off and the filtrate was diluted with DCM (10 mL) and washed with saturated $NaHCO_3$ solution (15 mL). The aqueous phase was extracted with DCM (3 x 10 mL) and the combined organic layers were washed with brine (50 mL), dried over $MgSO_4$ and concentrated in vacuo. The crude was purified by MPLC (cyclohexane/EtOAc 1:0 to 1:1) to afford the product (3.4 mg, 12%). $^{1}$**H-NMR** (600 MHz, $CD_2Cl_2$): δ 6.26 (ddd, *J* = 10.3, 4.0, 2.0 Hz, 1H), 6.04 (d, *J* = 6.5 Hz, 1H), 5.84 (dt, *J* = 10.3, 2.4 Hz, 1H), 5.77 (s, 1H), 4.71 (p, *J* = 8.8 Hz, 1H), 4.31 (qq, *J* = 6.9, 2.4 Hz, 1H), 3.70-3.63 (m, 1H), 3.28-3.19 (m, 2H), 2.30 (s, 3H), 2.24-2.16 (m, 2H), 1.97-1.88 (m, 1H), 1.72 (dddd, *J* = 18.0, 11.0, 9.6, 8.5 Hz, 1H),

1.25 (d, *J* = 6.9 Hz, 3H). **$^{13}$C-NMR** (151 MHz, $CD_2Cl_2$): δ 164.6, 163.1, 148.9, 130.4, 122.5, 110.0, 105.4, 95.9, 68.0, 53.2, 38.8, 28.1, 22.5, 22.1, 15.1. **HRMS**-ESI (m/z): $[M + H]^+$ calculated for $C_{16}H_{10}NO_3^+$, 274.1438; found, 274.1439.

**(±) 6-benzyl-2,7-dimethyl-3,4,4a,9a-tetrahydro-2H-pyrano[3',2':4,5]furo[3,2-c]pyridin-5(6H)-one (16d)**

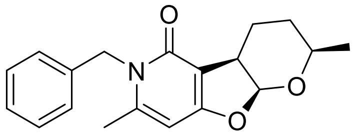


According to GP2, **7b**-*trans* (69.4 mg, 0.23 mmol) was reacted with **25c** (38.0 mg, 0.18 mmol) at 100 °C for 1 hour. After filtration over celite the crude was purified by FC (Hep/EtOAc 1:0 to 1:1) and the product was directly suspended with Pd/C (5 mg, 10 wt%) in toluene (2 mL) and was hydrogenated at 20 °C for 18 hours using a $H_2$-balloon. The catalyst was filtered off and the solvent removed in vacuo. The crude was purified by MPLC (EtOAc/MeOH 1:0 to 9:1 + 0.1% DIPEA) to afford the product (18.9 mg, 34%). **$^1$H-NMR** (500 MHz, $CDCl_3$): δ 7.30 (t, *J* = 7.4 Hz, 2H), 7.23 (t, *J* = 7.4 Hz, 1H), 7.13 (d, *J* = 7.4 Hz, 2H), 6.00 (d, *J* = 6.7 Hz, 1H), 5.93 (s, 1H), 5.46 (d, *J* = 15.9 Hz, 1H), 5.17 (d, *J* = 15.9 Hz, 1H), 3.87-3.79 (m, 1H), 3.47 (td, *J* = 6.7, 2.4 Hz, 1H), 2.54 (ddd, *J* = 13.6, 7.8, 2.4 Hz, 1H), 2.26 (s, 3H), 1.84 (tdd, *J* = 13.6, 6.2, 3.7 Hz, 1H), 1.64 (dq, *J* = 13.8, 3.7 Hz, 1H), 1.35 (tdd, *J* = 13.8, 10.4, 3.6 Hz, 1H), 1.23 (d, *J* = 6.3 Hz, 3H). **$^{13}$C-NMR** (126 MHz, $CDCl_3$): δ 166.2, 162.0, 148.8, 137.0, 128.9, 127.4, 126.5, 107.2, 106.2, 96.3, 70.6, 46.6, 38.5, 27.6, 23.1, 21.5, 20.1. **HRMS**-ESI (m/z): $[M + H]^+$ calculated for $C_{19}H_{22}NO_3^+$, 312.1594; found, 312.1592.

**(±) 6-(3,5-dimethylbenzyl)-2,7-dimethyl-4a,9a-dihydro-2H-pyrano[3',2':4,5]furo[3,2-c]pyridin-5(6H)-one (15e)**

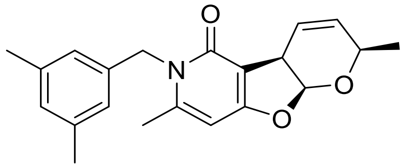


According to GP2, **7b**-*trans* (61.1 mg, 0.22 mmol) was reacted with **5n** (27.0 mg, 0.11 mmol) at 110 °C for 1 hour. The catalyst was filtered off and the filtrate was diluted with DCM (10 mL) and washed with saturated $NaHCO_3$ solution (15 mL). The aqueous phase was extracted with DCM (3 x 10 mL) and the combined organic layers were washed with brine (50 mL), dried over $MgSO_4$ and concentrated in vacuo. The crude was purified by prep. HPLC to afford the product (1.1 mg, 3%). **$^1$H-NMR** (600 MHz, $CD_2Cl_2$): δ 6.88 (s, 1H), 6.70 (s, 2H), 6.28 (ddd, *J* = 10.3, 3.9, 2.0 Hz, 1H), 6.12 (d, *J* = 6.5 Hz, 1H), 5.89 (s, 1H), 5.87 (dt, *J* = 10.3, 2.3 Hz, 1H), 5.36 (d, *J* = 15.9 Hz, 1H), 5.00 (d, *J* = 15.9 Hz, 1H), 4.35 (qq, *J* = 6.9, 2.3 Hz, 1H), 3.77-3.73 (bm, 1H), 2.26 (s, 6H), 2.24 (s, 3H), 1.28 (d, *J* = 6.9 Hz, 1H). **$^{13}$C-NMR** (151 MHz, $CD_2Cl_2$): δ 164.8, 161.2, 149.1, 138.3, 137.2, 130.0,

128.7, 123.8, 121.9, 108.4, 105.2, 95.5, 67.7, 46.3, 38.2, 21.7, 21.1. **HRMS**-ESI (m/z): [M + H]$^+$ calculated for $C_{21}H_{24}NO_3^+$, 338.1751; found, 338.1753.

**(±) 6-(4-methoxybenzyl)-2,7-dimethyl-4a,9a-dihydro-2H-pyrano[3',2':4,5]furo[3,2-c]pyridin-5(6H)-one (15f)**

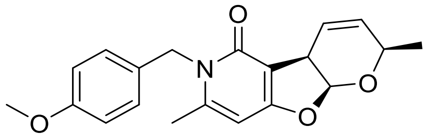

According to GP2, **7b**-*trans* (74.7 mg, 0.25 mmol) was reacted with **5d** (46.6 mg, 0.19 mmol) at 110 °C for 1 hour. After filtration over celite the crude was purified by FC (cyclohexane/EtOAc 1:0 to 1:1) to afford the product (23.2 mg, 36%). $^{1}$**H-NMR** (700 MHz, $CD_2Cl_2$): δ 7.07 (d, *J* = 8.7 Hz, 2H), 6.84 (d, *J* = 8.7 Hz, 2H), 6.26 (ddd, *J* = 10.3, 3.9, 2.0 Hz, 1H), 6.13 (d, *J* = 6.6 Hz, 1H), 5.92 (s, 1H), 5.88 (ddd, *J* = 10.3, 2.6, 1.9 Hz, 1H), 5.33 (m, 1H), 5.08 (d, *J* = 15.9 Hz, 1H), 4.36 (qq, *J* = 6.9, 2.6 Hz, 1H), 3.77-3.75 (m, 4H), 2.27 (s, 3H), 1.28 (d, *J* = 6.9 Hz, 1H). $^{13}$**C-NMR** (176 MHz, $CD_2Cl_2$): δ 165.5, 161.8, 159.3, 149.6, 130.5, 129.3, 128.1, 122.1, 114.4, 109.0, 105.8, 96.4, 68.2, 55.6, 46.5, 38.4, 22.2, 21.5. **HRMS**-ESI (m/z): [M + H]$^+$ calculated for $C_{20}H_{22}NO_4^+$, 340.1543; found, 340.1545.

**(±) 6-(4-methoxybenzyl)-2,7-dimethyl-3,4,4a,9a-tetrahydro-2H-pyrano[3',2':4,5]furo[3,2-c]pyridin-5(6H)-one (16f); (±) 4-hydroxy-1-(4-methoxybenzyl)-6-methyl-3-(2-methyltetrahydro-2H-pyran-3-yl)pyridin-2(1H)-one (17bg)**

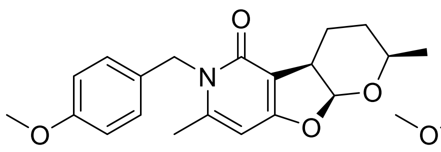

16f

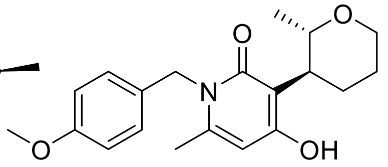

17bg

According to GP3, **7b**-*trans* (69.6 mg, 0.23 mmol) was reacted with **5d** (49.1 mg, 0.20 mmol). After purification by MPLC (cyclohexane/EtOAc 1:0 to 0:1), the product was directly suspended with Pd/C (5 mg, 10 wt%) in toluene (2 mL) and was hydrogenated at 20 °C for 48 hours using a $H_2$-balloon. The catalyst was filtered off and the solvent removed in vacuo. The crude was purified by prep. HPLC to afford product **16f** (5.9 mg, 9%) and **17bg** (9.5 mg, 14%) in separated fractions. $^{1}$**H-NMR** product **16f** (600 MHz, $CD_2Cl_2$): δ 7.07 (d, *J* = 8.7 Hz, 2H), 6.84 (d, *J* = 8.7 Hz, 2H), 5.82 (s, 1H), 5.42 (d, *J* = 5.1 Hz, 1H), 5.26 (d, *J* = 15.5 Hz, 1H), 5.17 (d, *J* = 15.5 Hz, 1H), 4.46 (q, *J* = 6.5 Hz, 1H), 3.76 (s, 3H), 3.21 (bs, 1H), 2.24 (s, 3H), 2.21-2.10 (m, 3H), 1.99-1.94 (m, 1H), 1.35 (d, *J* = 6.5 Hz, 3H). $^{13}$**C-NMR** product **16f** (151 MHz, $CD_2Cl_2$): δ 164.1, 163.8, 159.3, 145.3, 129.2, 128.0, 114.5, 114.4, 103.0, 98.3, 77.4, 55.6, 47.5, 34.4, 28.3, 21.3, 20.7, 20.6. **HRMS**-ESI (m/z) product **16f**: [M + H]$^+$ calculated for $C_{20}H_{24}NO_4^+$, 342.1670; found, 342.1707. $^{1}$**H-NMR** product **17bg** (400 MHz, MeOH-d$_4$): δ 7.01 (d, *J* = 8.6 Hz, 2H), 6.86 (d, *J*

= 8.6 Hz, 2H), 5.86 (s, 1H), 5.31-5.21 (m, 2H), 4.36-4.18 (m, 1H), 3.93 (dd, *J* = 11.3, 4.3 Hz, 1H), 3.76 (bs, 4H), 3.56 (t, *J* = 11.3 Hz, 1H), 2.16 (s, 3H), 1.79-1.52 (m, 4H), 1.02 (d, *J* = 6.2 Hz, 3H). **$^{13}$C-NMR** product **17bg** (126 MHz, MeOH-$d_4$): δ 164.8, 160.4, 160.3, 146.7, 130.3, 128.4, 115.2, 111.4, 101.0, 76.5 69.4, 55.7, 47.3, 40.1, 28.2, 28.1, 20.4. **HRMS**-ESI (m/z) product **17bg**: $[M + H]^+$ calculated for $C_{20}H_{26}NO_4^+$, 344.1856; found, 344.1865.

**(±) 6-(4-fluorobenzyl)-2,7-dimethyl-4a,9a-dihydro-2H-pyrano[3',2':4,5]furo[3,2-c]pyridin-5(6H)-one (15g)**

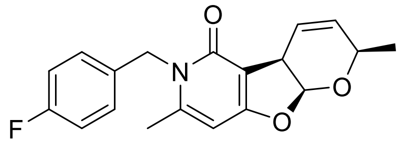


According to GP2, **7b**-*trans* (75.9 mg, 0.25 mmol) was reacted with **5k** (45.0 mg, 0.19 mmol) at 100 °C for 1 hour. After filtration over celite the crude was purified by FC (cyclohexane/EtOAc 1:0 to 1:1) to afford the product (15.9 mg, 25%). **$^1$H-NMR** (700 MHz, $CD_2Cl_2$): δ 7.13 (dd, *J* = 8.6, 5.4 Hz, 2H), 7.02 (t, *J* = 8.6 Hz, 2H), 6.23 (ddd, *J* = 10.4, 3.9, 2.3 Hz, 1H), 6.16 (d, *J* = 6.2 Hz, 1H), 5.99 (s, 1H), 5.89 (ddd, *J* = 10.4, 2.7, 1.8 Hz, 1H), 5.37 (d, *J* = 16.1 Hz, 1H), 5.15 (d, *J* = 16.1 Hz, 1H), 4.37 (qq, *J* = 6.9, 2.7 Hz, 1H), 3.77 (dq, *J* = 6.2, 2.3 Hz, 1H), 2.30 (s, 3H), 1.29 (d, *J* = 6.9 Hz, 3H). **$^{13}$C-NMR** (176 MHz, $CD_2Cl_2$): δ 166.0, 163.1, 161.7, 161.9, 149.6, 133.0, 130.1, 128.6, 121.7, 115.9, 109.4, 106.1, 97.1, 68.2, 46.7, 38.1, 22.2, 21.5. **HRMS**-ESI (m/z): $[M + H]^+$ calculated for $C_{19}H_{19}FNO_3^+$, 328.1344; found, 328.1343.

**(±) 8-methyl-1,2,3,4,5b,9a-hexahydro-5H,8H-cyclopenta[b]pyrano[3',2':4,5]furo[2,3-d]pyridin-5-one (15h)**

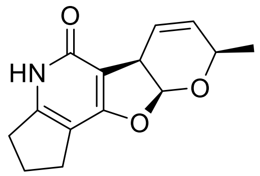


According to GP2, **7b**-*trans* (50.0 mg, 0.17 mmol) was reacted with 4-hydroxy-1,5,6,7-tetrahydro-2H-cyclopenta[b]pyridin-2-one (25.0 mg, 0.17 mmol) at 110 °C for 1 hour. The catalyst was filtered off and the filtrate was diluted with EtOAc (10 mL) and washed with water (15 mL). The aqueous phase was extracted with EtOAc (3 x 10 mL) and the combined organic layers were washed with brine (50 mL), dried over $MgSO_4$ and concentrated in vacuo. The crude was purified by prep. HPLC to afford the product (0.80 mg, 2%). **$^1$H-NMR** (700 MHz, $CD_2Cl_2$): δ 6.22-6.17 (m, 2H), 5.88 (dt, *J* = 10.4, 2.4 Hz, 1H), 4.37 (dq, *J* = 6.9, 2.4 Hz, 1H), 3.75-3.72 (m, 1H), 2.86 (t, *J* = 7.5 Hz, 2H), 2.80-2.70 (m, 2H), 2.16 (p, *J* = 7.3 Hz, 2H), 1.27 (d, *J* = 6.9 Hz, 1H). **HRMS**-ESI (m/z): $[M + H]^+$ calculated for $C_{14}H_{16}NO_3^+$, 246.1125; found, 246.1127.

**(±) 6-(2-chlorobenzyl)-2,7-dimethyl-4a,9a-dihydro-2H-pyrano[3',2':4,5]furo[3,2-c]pyridin-5(6H)-one (15i); (±) 8-(2-chlorobenzyl)-1,7-dimethyl-8,9b-dihydro-1H-pyrano[3',4':4,5]furo[3,2-c]pyridin-9(4aH)-one (9bi)**

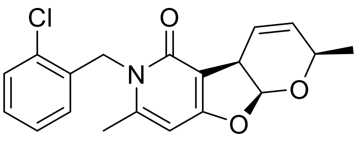

15i

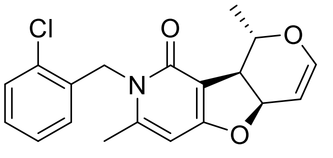

9bi

According to GP2, **7b**-*trans* (67.8 mg, 0.22 mmol) was reacted with **5l** (28.0 mg, 0.11 mmol) at 110 °C for 1 hour. The catalyst was filtered off and the filtrate was diluted with DCM (10 mL) and washed with saturated $NaHCO_3$ solution (15 mL). The aqueous phase was extracted with DCM (3 x 10 mL) and the combined organic layers were washed with brine (50 mL), dried over $MgSO_4$ and concentrated in vacuo. The crude was purified by prep. HPLC to afford product **15i** (7.7 mg, 20%) and **9bi** (1.0 mg, 3%) in separated fractions. **$^1$H-NMR** product **15i** (600 MHz, $CD_2Cl_2$): δ 7.41 (dd, *J* = 7.7, 1.5 Hz, 1H), 7.23 (td, *J* = 7.7, 1.8 Hz, 1H), 7.19 (td, *J* = 7.9, 1.5 Hz, 1H), 6.72 (d, *J* = 7.9 Hz, 1H), 6.26 (ddd, *J* = 10.3, 3.9, 2.0 Hz, 1H), 6.14 (d, *J* = 6.5 Hz, 1H), 5.95 (s, 1H), 5.88 (ddd, *J* = 10.3, 2.6, 1.8 Hz, 1H), 5.41 (d, *J* = 16.9 Hz, 1H), 5.23 (d, *J* = 16.9 Hz, 1H), 4.36 (qq, *J* = 6.9, 2.6 Hz, 1H), 3.80-3.73 (m, 1H), 2.19 (s, 3H), 1.29 (d, *J* = 6.9 Hz, 1H). **$^{13}$C-NMR** product **15i** (151 MHz, $CD_2Cl_2$): δ 165.5, 161.5, 149.3, 134.8, 132.7, 130.5, 129.9, 128.8, 128.7, 126.7, 122.1, 108.8, 105.7, 96.3, 68.1, 44.6, 38.5, 22.1, 21.2. **HRMS**-ESI (m/z) product **15i**: $[M + H]^+$ calculated for $C_{19}H_{19}ClNO_3^+$, 344.1048; found, 344.1050. **$^1$H-NMR** product **9bi** (600 MHz, $CD_2Cl_2$): δ 7.41 (dd, *J* = 7.7, 1.5 Hz, 1H), 7.23 (td, *J* = 7.7, 1.9 Hz, 1H), 7.19 (td, *J* = 7.5, 1.5 Hz, 1H), 6.79 (d, *J* = 6.2 Hz, 1H), 6.71 (dd, *J* = 7.5, 1.9 Hz, 1H), 5.92 (s, 1H), 5.40 (d, *J* = 16.9 Hz, 1H), 5.28 (d, *J* = 16.9 Hz, 1H), 5.26 (dd, *J* = 6.2, 4.7 Hz, 1H), 4.98 (ddd, *J* = 7.0, 4.7, 1.1 Hz, 1H), 3.53 (dq, *J* = 10.7, 6.3 Hz, 1H), 3.03 (dd, *J* = 10.7, 7.0 Hz, 1H), 2.19 (s, 3H), 1.46 (d, *J* = 6.3 Hz, 3H). **$^{13}$C-NMR** product **9bi** (151 MHz, $CD_2Cl_2$): δ 168.4, 161.9, 150.0, 149.5, 134.9, 132.7, 129.9, 128.8, 127.7. 126.5, 107.6, 98.5, 96.3, 79.4, 74.0, 44.8, 44.0, 21.2, 19.8. **HRMS**-ESI (m/z) product **9bi**: $[M + H]^+$ calculated for $C_{19}H_{19}ClNO_3^+$, 344.1048; found, 344.1045.

**(±) 2,7-dimethyl-6-(thiophen-2-ylmethyl)-4a,9a-dihydro-2H-pyrano[3',2':4,5]furo[3,2-c]pyridin-5(6H)-one (15j); (±) 1,7-dimethyl-8-(thiophen-2-ylmethyl)-8,9b-dihydro-1H-pyrano[3',4':4,5]furo[3,2-c]pyridin-9(4aH)-one (9bj)**

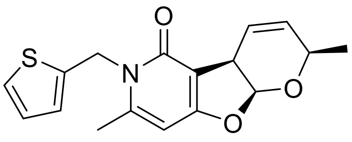

**15j**

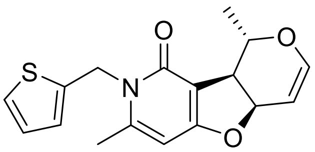

**9bj**

According to GP2, **7b**-*trans* (66.5 mg, 0.22 mmol) was reacted **5j** (44.3 mg, 0.20 mmol) at 110 °C for 1 hour. After filtration over celite the crude was purified by MPLC (cyclohexane/EtOAc 1:0 to 0:1) to afford a mixture of products a and b. The product mixture was subjected for separation by prep. HPLC to afford pure product **15j** (9.8 mg, 16%) and **9bj** (3.4 mg, 5%). **$^{1}$H-NMR** product **15j** (700 MHz, $CD_2Cl_2$): δ 7.24 (dd, *J* = 5.1, 1.2 Hz, 1H), 7.00 (dd, *J* = 3.5, 1.2 Hz, 1H), 6.94 (dd, *J* = 5.1, 3.5 Hz, 1H), 6.23 (ddd, *J* = 10.4, 3.8, 2.0 Hz, 1H), 6.15 (d, *J* = 6.6 Hz, 1H), 5.89 (ddd, *J* = 10.4, 2.7, 1.8 Hz, 1H), 5.97 (s, 1H), 5.47 (d, *J* = 15.5 Hz, 1H), 5.28 (d, *J* = 15.5 Hz, 1H), 4.37 (qq, *J* = 6.9, 2.7 Hz, 1H), 3.79-3.75 (m, 1H), 2.44 (s, 1H), 1.27 (d, *J* = 6.9 Hz, 3H). **$^{13}$C-NMR** product **15j** (176 MHz, $CD_2Cl_2$): δ 166.1, 161.5, 149.2, 139.4, 130.7, 127.0, 126.9, 126.1, 121.6, 109.4, 106.2, 97.3, 68.2, 43.0, 37.9, 22.3, 21.4. **HRMS**-ESI (m/z) product **15j**: $[M + H]^+$ calculated for $C_{17}H_{18}NO_3S^+$, 316.1002; found, 316.1016. **$^{1}$H-NMR** product **9bj** (700 MHz, $CD_2Cl_2$): δ 7.24 (dd, *J* = 5.1, 1.2 Hz, 1H), 7.02 (dd, *J* = 3.5, 1.2 Hz, 1H), 6.95 (dd, *J* = 5.1, 3.5 Hz, 1H), 6.78 (d, *J* = 6.2 Hz, 1H), 5.94 (s, 1H), 5.42-5.37 (bs, 2H), 5.24 (dd, *J* = 6.2, 4.7 Hz, 1H), 4.96 (ddd, *J* = 6.8, 4.7, 1.1 Hz, 1H), 3.47 (dq, *J* = 10.8, 6.3 Hz, 1H), 3.05 (dd, *J* = 10.8, 6.8 Hz, 1H), 2.45 (s, 3H), 1.48 (d, *J* = 6.3 Hz, 3H). **$^{13}$C-NMR** product **9bj** (176 MHz, $CD_2Cl_2$): δ 168.9, 161.9, 150.2, 149.5, 139.6, 127.0, 126.8, 126.2, 108.2, 98.3, 97.2, 79.7, 73.9, 43.7, 43.1, 21.5, 19.6. **HRMS**-ESI (m/z) product **9bj**: $[M + H]^+$ calculated for $C_{17}H_{18}NO_3S^+$, 316.1002; found, 316.1004.

## 2. Substructure Search in DNP

Substructure search for combinations of pyridones, THPs and DHPs was performed on the open source website:
http://dnp.chemnetbase.com/faces/chemical/ChemicalSearch.xhtml.

All permutations of the combinations of *N*-methyl-2-pyridone with dihydropyrane (DHP) or tetrahydropyrane (THP) in a monopodal, bipodal or edge-on connection type were analysed. In

the following only combinations for which natural product examples in the DNP were found are listed in Figure S1. Altogether 121 natural products containing examples for combinations between *N*-methyl-2-pyridone with DHPs and *N*-methyl-2-pyridone with THPs were found. The DNP Version 27.2 has approximately a total of 40 000 entries, giving a coverage of 0.3% of the reported NP chemical space by the shown combinations.

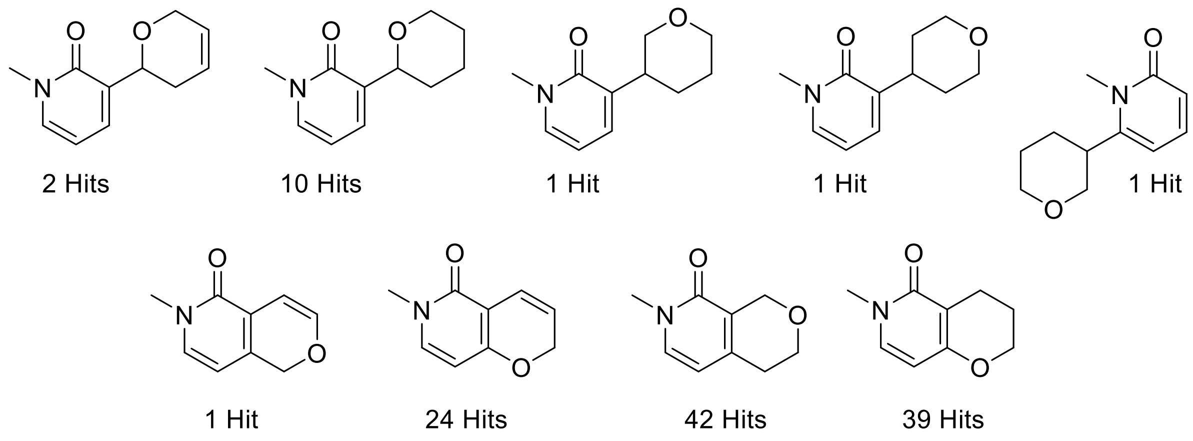


**Figure S1.** Combinations of *N*-methyl-2-pyridone with dihydropyrane (DHP) or tetrahydropyrane (THP), in a monopodal, bipodal or edge-on connection type for which examples were found in the DNP (Version 27.2).

## 3. Biology

### 3.1. Cell-Painting Assay

The described assay follows closely the method described by Bray et al.[18] and was performed by the Compound Management and Screening Center (COMAS). Initially, 5 µl U2OS medium were added to each well of a 384-well plate (PerkinElmer CellCarrier-384 Ultra). Subsequently, U2OS cells were seeded with a density of 1600 cells per well in 20 µl medium. The plate was incubated for 5 min at the ambient temperature, followed by an additional 4 h incubation (37 °C, 5% $CO_2$). Compound treatment was performed with the Echo 520 acoustic dispenser (Labcyte) at final concentrations of 50, 30, 10, 3 or 1 µM for 20 h (37 °C, 5% $CO_2$). Subsequently, mitochondria were stained with Mito Tracker Deep Red (Thermo Fisher Scientific, Cat. No. M22426). The MitoTracker Deep Red stock solution (1 mM) was diluted to a final concentration of 100 nM in prewarmed medium. The medium was removed from the plate leaving 10 µl residual volume and 25 µl of the Mito Tracker solution were added to each well. The plate was incubated for 30 min in the dark (37 °C, 5% $CO_2$). To fix the cells 7 µl of 18.5 % formaldehyde in PBS were added, resulting in a final formaldehyde concentration of

3.7 %. Subsequently, the plate was incubated for another 20 min in the dark (37 °C, 5% $CO_2$) and washed three times with 70 µl of PBS using the Biotek Washer Elx405. Cells were permeabilized by addition of 25 µl 0.1% Triton X-100 to each well, followed by 15 min incubation (37 °C, 5% CO2) in the dark. The cells were washed three times with PBS leaving a final volume of 70 µl. To each well 25 µl of a staining solution were added, which contains 1% BSA, 50 µl phalloidin (Thermo Fisher Scientific, A12381), 25 µg/ml concanavalin A (Thermo Fisher Scientific, Cat. No. C11252), 50 µl/ml Hoechst 33342 (Sigma, Cat. No. B2261-25mg), 15 µl/ml WGA-Alexa594 conjugate (Thermo Fisher Scientific, Cat. No. W11262) and 0.3 µl/ml SYTO 14 solution (Thermo Fisher Scientific, Cat. No. S7576). The plate was incubated for 30 min (37 °C, 5% $CO_2$) in the dark and washed three times with 70 µl PBS. After the final washing step, the PBS was not aspirated. The plates were sealed and centrifuged for 1 min at 500 rpm.

The plates were prepared in triplicates with shifted layouts to reduce plate effects and imaged using a Micro XL High-Content Screening System (Molecular Devices, 5 channels, 9 sites per well, 20x magnification, binning 2).

The generated images were processed with the CellProfiler package (https://cellprofiler.org/) on a computing cluster of the Max Planck Society to extract 1716 cell features (parameters).

Further analysis was performed with custom Python (https://www.python.org/) scripts using the Pandas (https://pandas.pydata.org/) and Dask (https://dask.org/) data processing libraries (separate publication to follow).

In a first step, the data was aggregated as overall medians per well.

A subset of highly reproducible parameters was determined using the procedure described by Woehrmann et al.[19] in the following way:

Two biological repeats of one plate containing reference compounds were analyzed. For every parameter, its full profile over each whole plate was calculated. If the profiles from the two repeats showed a similarity >= 0.8 (see below), the parameter was added to the set.

This was carried out once and resulted in a set of 579 parameters that was used for all further analyses.

Z-scores were then calculated for each parameter as how many times the MAD of the controls the measured value deviates from the median of the controls:

$$z - score = \frac{value_{meas.} - Median_{Controls}}{MAD_{Controls}}$$

The phenotypic compound profile is then the list of z-scores of all parameters for one compound.

In addition to the phenotypic profile, an induction value was determined for each compound as the fraction of significantly changed parameters, in percent:

$$Induction\ [\%] = \frac{number\ of\ parameters\ with\ abs.\ \ values\ >\ 3}{total\ number\ of\ parameters}$$

Similarities of phenotypic profiles were calculated from the correlation distances between two profiles (https://docs.scipy.org/doc/scipy/reference/generated/scipy.spatial.distance.correlation.html; Similarity = 1 - Correlation Distance) and the compounds with the most similar profiles were determined from a set of 3000 reference compounds that was also measured in the assay.

### 3.1.1. Clustering

The compounds were sorted by descending induction and therefore the highest inducing compound was put into the first cluster. Compounds exhibiting a fingerprint similarity above 80% to that first compound would be added to this cluster. When no more compounds could be added, a new analysis of the remaining compounds was started. The procedure was repeated until all compounds were distributed into clusters.

**Table S1.** Induction cut-off filter < 10%, > 80% for compounds measured at 10 µM.

| Compound | Induction [%] at 10µM | Cluster | BioSim [%] | ChemSim |
|---|---|---|---|---|
| **14dk** | 29 | 1 | - | - |
| **14ek** | 23 | 1 | 84 | 0.72 |
| **8d** | 27 | 2 | - | - |
| **14cf** | 17 | 3 | - | - |
| **8b** | 14 | 4 | - | - |

**Table S2.** Induction cut-off filter < 10%, > 80% for compounds measured at 10, 30 and 50 µM. Only compounds belonging to cluster 1 are listed.

| **Compound** | **Induction** [%] | **Cluster** | **BioSim** [%] | **ChemSim** |
|---|---|---|---|---|
| **14ek** | 80 (at 50 µM) | 1 | - | - |
| **14ek** | 69 (at 30 µM) | 1 | 98 | 1.00 |
| **14dd** | 28 (at 50 µM) | 1 | 93 | 0.44 |
| **14df** | 40 (at 50 µM) | 1 | 93 | 0.49 |
| **14de** | 34 (at 50 µM) | 1 | 92 | 0.45 |
| **14dg** | 47 (at 50 µM) | 1 | 90 | 0.43 |
| **14df** | 27 (at 30 µM) | 1 | 89 | 0.49 |
| **14de** | 17 (at 30 µM) | 1 | 89 | 0.45 |
| **14dk** | 36 (at 30 µM) | 1 | 88 | 0.72 |
| **14dc** | 39 (at 50 µM) | 1 | 87 | 0.42 |
| **14dk** | 35 (at 50 µM) | 1 | 86 | 0.72 |
| **14dl** | 25 (at 50 µM) | 1 | 85 | 0.36 |
| **14dg** | 34 (at 30 µM) | 1 | 85 | 0.43 |
| **14dd** | 16 (at 30 µM) | 1 | 85 | 0.44 |
| **14dk** | 29 (at 10 µM) | 1 | 84 | 0.72 |
| **14cf** | 29 (at 50 µM) | 1 | 82 | 0.48 |

### 3.1.2. Cross-Correlation Matrix

**Table S3.** Fingerprints of entries in the y-axis were individually compared to fingerprints of annotated reference compounds on the x-axis. References targeting GPCRs were excluded for the analysis as they were found to occur ubiquitous in the whole data set for yet unresolved reasons.

| BioSim ↘ | **Aumitin**[20] (10 µM) | **Chromo-pynone 1**[21] (50 µM) | **Lipoxygenin**[22] (50 µM) | **Pipinib**[23] (30 µM) | **GW2974**[24] (10 µM) |
|---|---|---|---|---|---|
| **14dk** (10 µM) | 78% | 81% | 85% | 81% | 87% |
| **14dk** (30 µM) | 88% | 89% | 94% | 91% | 87% |
| **14dk** (50 µM) | 87% | 88% | 94% | 89% | 84% |
| **14ek** (10 µM) | 63% | 61% | 63% | 65% | 75% |
| **14ek** (30 µM) | 90% | 85% | 84% | 94% | 85% |
| **14ek** (50 µM) | 92% | 89% | 86% | 93% | 88% |
| Annotated bioactivity | Autophagy inhibition | Glucose uptake inhibition | Wnt inhibition | Hedgehog (Hh) inhibition | erbB inhibitor |
| Target | Mitochondrial complex I | GLUT-1/3 | 5-LO | (PI4KIIIβ) | erbB 1/2 |
| In-house observed pathway bioactivity | ROS, GLUT, Wnt, Hh | Autophagy, Wnt, Hh | Autophagy, Hh | Autophagy, Wnt | ROS |

## 3.1.3. Statistical Analysis of Structure-Induction-Relationship

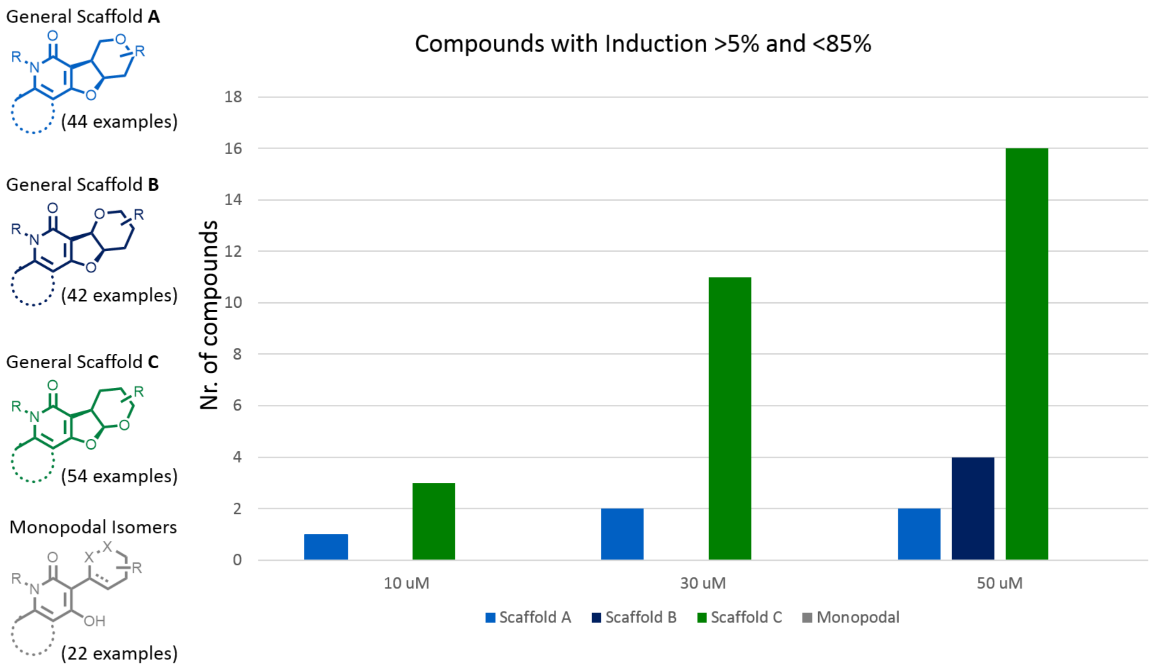


**Figure S2.** Number of active compounds distributed by their substructure classes.

## 3.1.4. Representative Cell-Painting Microscopy Images

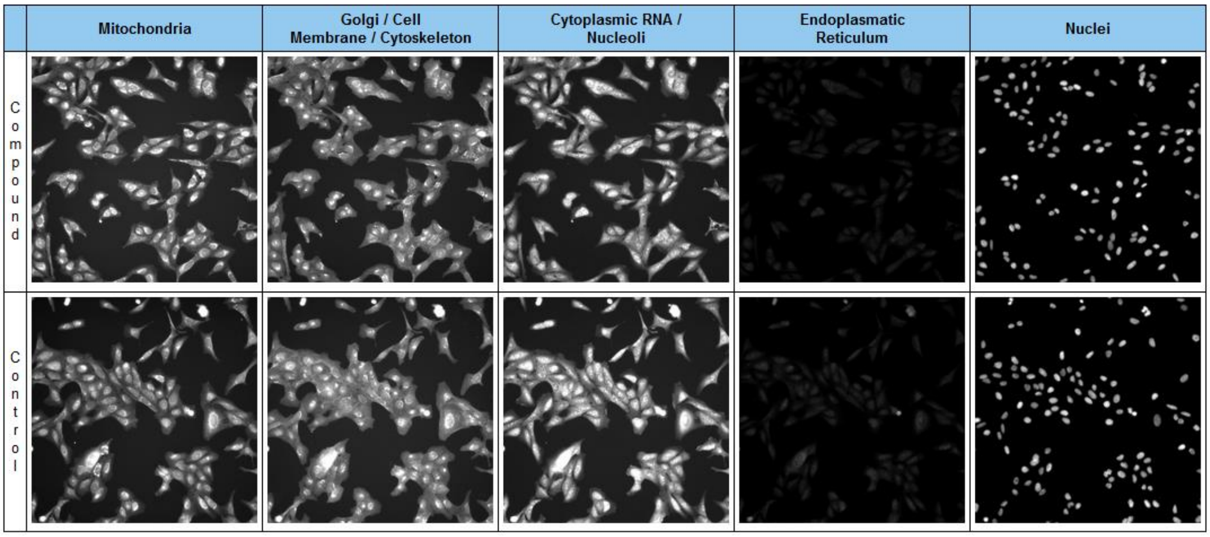


**Figure S3.** Microscopy images recorded for **14dk** at 10 µM; cell-count = 90%

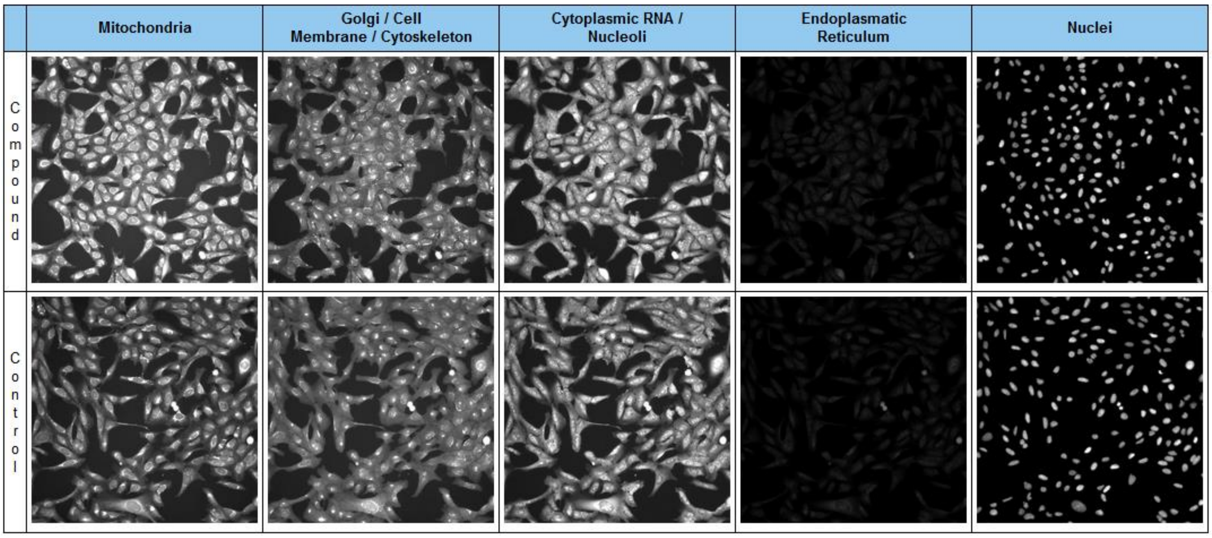


**Figure S4.** Microscopy images recorded for **14de** at 30 µM; cell-count = 96%

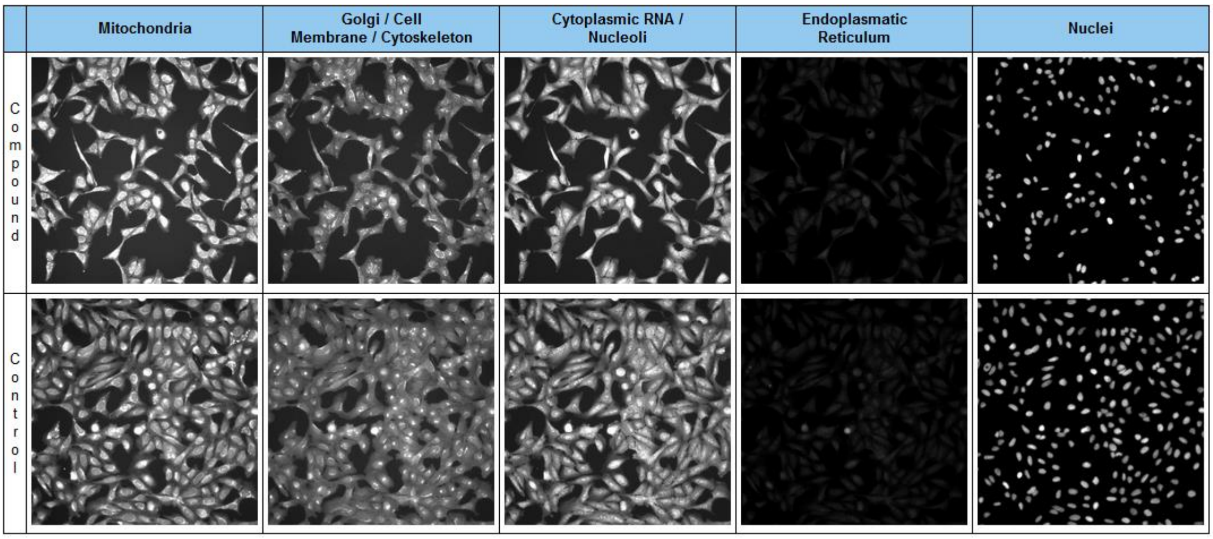


**Figure S5.** Microscopy images recorded for **14dk** at 50 µM; cell-count = 93%

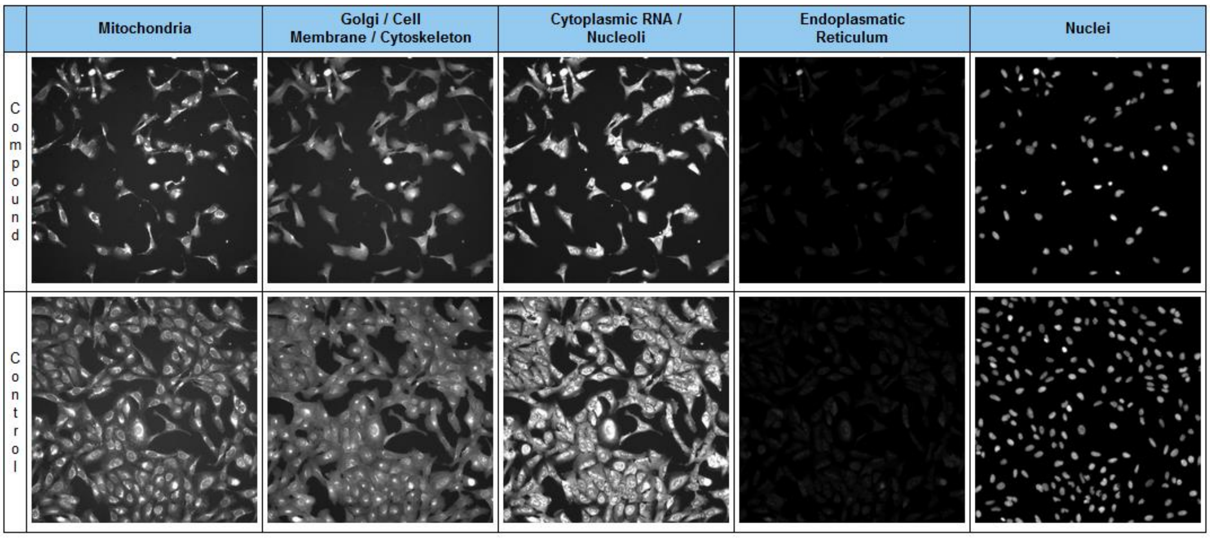


**Figure S6.** Microscopy images recorded for **8d** at 10 µM; cell-count = 50%

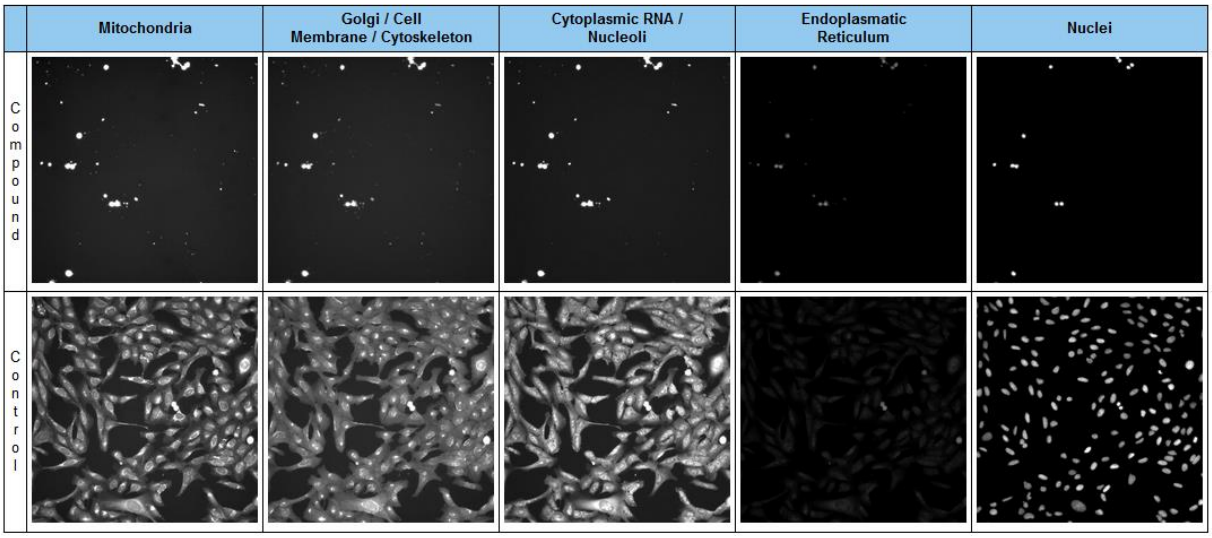


**Figure S7.** Microscopy images recorded for **8d** at 30 µM; cell-count = 34%

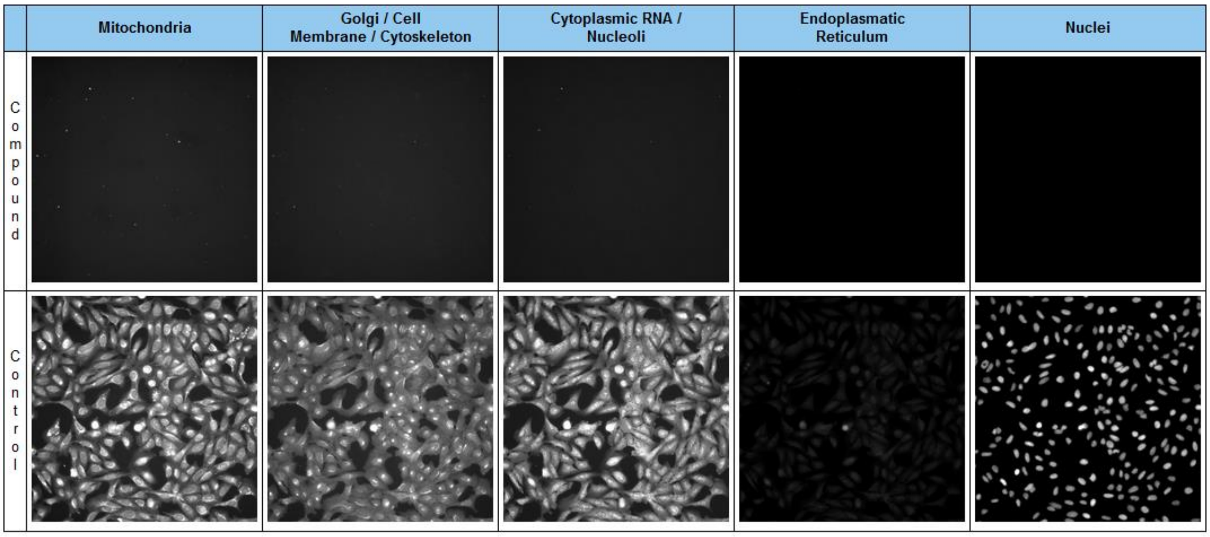


**Figure S8.** Microscopy images recorded for **8d** at 50 µM; cell-count = 7%

### 3.1.5. Additivity of Profiles

The mathematical addition of the fingerprints of fragments **5r** and **8d** generates an artificial fingerprint **29dj-art.** representing the combination of both fragments. This has a profile similarity of 4% when compared to the experimentally derived fingerprint of **29dj** corresponding to the synthesized combination of fragments **5r** and **8d**.

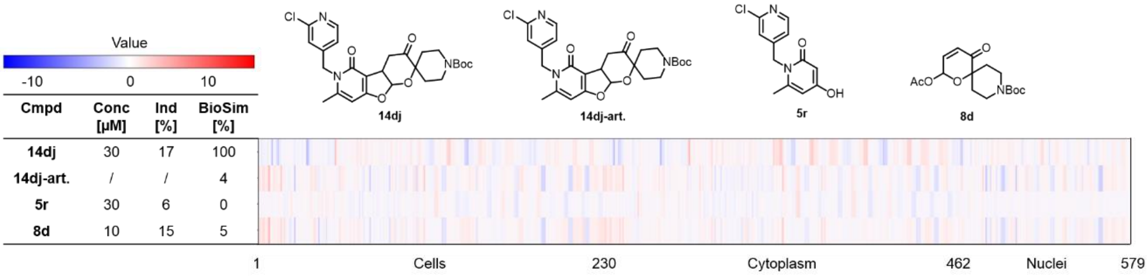


| Cmpd | Conc [µM] | Ind [%] | BioSim [%] |
|---|---|---|---|
| 14dj | 30 | 17 | 100 |
| 14dj-art. | / | / | 4 |
| 5r | 30 | 6 | 0 |
| 8d | 10 | 15 | 5 |

**Figure S9.** Evaluation of additivity of profiles. The top line is set as reference fingerprint (--- % BioSim) to which subjacent fingerprints are compared, respectively; blue indicates a decrease of a specific parameter compared to DMSO control; red indicates an increase of a specific parameter compared to DMSO control.

### 3.2. Cell Culture

HeLa (ACC 57) cells were purchased from DSMZ GmbH (Germany) and cultured in DMEM with 10% FBS, sodium pyruvate, non-essential amino acids, penicillin and streptomycin. Cells were incubated at 37°C, 5% $CO_2$ in a humidified atmosphere. During regular testing for mycoplasma infections, cells were found negative.

### 3.3. Cell Mito Stress Test

The influence of test compounds on mitochondrial respiration was assessed using the Seahorse XFp analyzer (Agilent, USA) in combination with the Cell Mito Stress Test kit (Agilent, USA) according to the manufacturer's protocol. 20,000 HeLa cells per well were seeded into XFp cell culture plates (Agilent, USA) and incubated at 37°C, 5% $CO_2$ overnight. XFp cartridges were hydrated using XF Calibrant and incubated overnight at 37°C. Seeding medium was exchanged for pH 7.4 DMEM-based assay medium (Agilent, USA) containing 2 mM GlutaMAX (ThermoFisher), 1 mM sodium pyruvate (PAN Biotech, Germany) and 25 mM glucose (Sigma-Aldrich, Germany). Oxygen consumption rate (OCR) and extracellular acidification rate (ECAR) were measured in intervals of 6 min. After five measurement intervals of baseline recording, the test compounds were injected, followed by ten measurement intervals. Subsequently, Oligomycin, FCCP and Rotenone/Antimycin A were injected, followed by three measurement intervals after each injection. Data was background-subtracted and normalized to the last baseline measurement (=100%), using the Wave software (Agilent, USA).

### 3.4. Semi-Intact Assay for Mitochondrial Respiration

Inhibition of mitochondrial complexes I-IV was tested using the Seahorse XFp analyzer. Seeding of 10,000 HeLa cells per well and hydration of XFp cartridges were performed as described for the Cell Mito Stress Test. The assay was performed using MAS buffer (220 mM mannitol, 70 mM sucrose, 10 mM $KH_2PO_4$, 5 mM $MgCl_2$, 2 mM HEPES, 1 mM EGTA, pH 7.4). The buffer was supplemented with 0.5% (w/v) fatty acid free BSA for complex I-III. Oxygen consumption rate (OCR) was measured in intervals of 8 min. After three baseline measurement intervals, the test compound, 1 nM of Seahorse XF Plasma membrane permeabilizer (Agilent) and 1 mM ADP were injected together with 10 mM pyruvate / 1 mM malate for complex I, 10 mM succinate / 1 µM rotenone for complex II, 0.2 mM duroquinol for complex III or 0.5 mM tetramethylphenylenediamine (TMPD) / 2 mM ascorbate for complex IV, followed by three measurement intervals. Subsequently, first 1 µM Oligomycin, then 1 µM antimycin A (complex I and II) or 20 mM sodium azide (complex III and IV) were injected, followed by three measurement intervals each. Data analysis was performed as described for the Cell Mito Stress Test.

### 3.5. MitoSOX Red Assay

Mitochondrial superoxide levels were determined using the indicator dye MitoSOX Red (ThermoFisher, USA). 15,000 Hela cells were seeded per well into black 96 well plates with clear flat bottom and incubated at 37°C, 5% $CO_2$ overnight. Seeding medium was exchanged for staining medium comprising DMEM without additives containing 5 µM MitoSOX Red and 5 µg/µL Hoechst-33342 (ThermoFisher, USA). Cells were incubated for 30 min at 37°C, 5% $CO_2$. Subsequently, the medium was exchanged for DMEM with additives containing test compounds, followed by 60 min of incubation at 37°C, 5% $CO_2$. Cells were fixed in PBS containing 0.5% paraformaldehyde for 10 min at room temperature and washed three times with PBS. Cells were imaged using an Axiovert 200M automated microscope (Carl Zeiss, Germany) at 10x magnification. MetaMorph 7.7.8.0 (Visitron, Germany) was used to quantify the integrated fluorescence intensity of MitoSOX Red per cell. The data was normalized to control cells treated with either DMSO (=0%) or 10 µM CDNB (=100%). Non-linear regression via four-parameter fit was performed using Prism 7 (GraphPad Software, USA) and $EC_{50}$ values were obtained by interpolating X values for 50% staining intensity.

## 3.6. Structure-Phenotype and Structure-Activity Relationship Studies

**Table S4.** Establishment of a structure-phenotype relationship (SPR) for the PFPs by means of the induction parameter delineated from the cell painting assay, and comparison with activity in the MitoSOX Red assay; $EC_{50}$ determined in HeLa cells (n = 3); Biosimilarity was compared to **14dk** if not indicated differently; n.c. means that biosimilarity was not calculated because induction was out of 20-40% comparison window; 1) Biosimilarity was compared to **14df**.

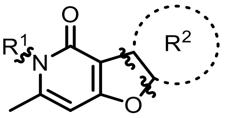


| Entry | Nr. | R¹ = | R² = | Induction [%] | BioSim [%] | $EC_{50}$ [µM] |
|---|---|---|---|---|---|---|
| 1 | **14db** | 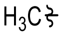 | 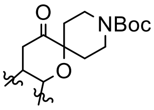 | 2 (50 µM) | n.c. | >30 |
| 2 | **14dc** | 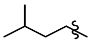 | 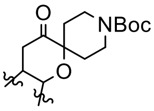 | 39 (50 µM) | 92 | >30 |
| 3 | **14dk** | 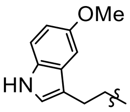 | 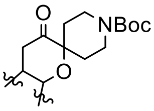 | 29 (30 µM) | 100 | 3.7 ± 0.9 |
| 4 | **14dd** | 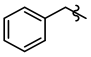 | 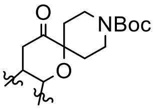 | 28 (50 µM) | 87 | 9.2 ± 2.4 |
| 5 | **14df** | 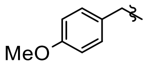 | 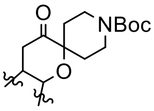 | 27 (30 µM) | 85 | 6.8 ± 0.7 |
| 6 | **14de** | 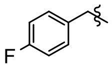 | 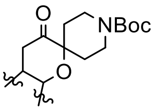 | 34 (50 µM) | 87 | 15.7 ± 6.6 |
| 7 | **14dg** | 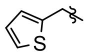 | 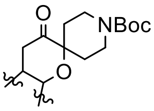 | 34 (30 µM) | 90 | 13.4 ± 2.6 |
| 8 | **14di** | 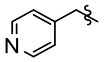 | 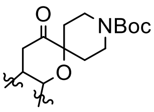 | 3 (50 µM) | n.c. | >30 |

| | | | | | | |
|---|---|---|---|---|---|---|
| 9 | **14dh** | 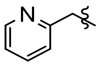 | 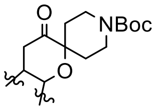 | 10 (50 µM) | n.c. | 25.3 ± 6.2 |
| 10 | **14dj** | 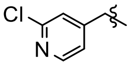 | 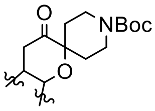 | 24 (50 µM) | 90 | 9.7 ± 1.8 |
| 11 | **13ah** | 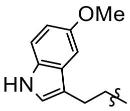 | 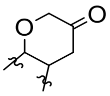 | 15 (50 µM) | 37 | >30 |
| 12 | **18k** | 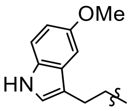 | 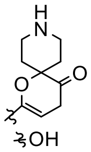 | 3 (50 µM) | n.c. | >30 |
| 13 | **14ek** | 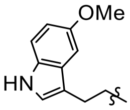 | 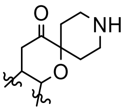 | 23 (10 µM) | 84 | 10.7 ± 3.6 |
| 14 | **14cf** | 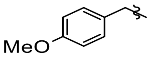 | 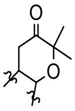 | 28 (30 µM) | 62[1] | >30 |
| 15 | **14ad** | 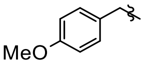 | 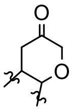 | 6 (50 µM) | n.c. | >30 |
| 16 | **13ce** | 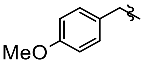 | 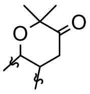 | 3 (50 µM) | n.c. | >30 |
| 17 | **14aa** | $H_3C$ | 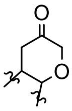 | 1 (50 µM) | n.c. | >30 |
| 18 | **14ef** | 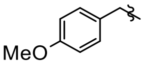 | 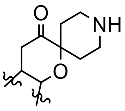 | 0 (10 µM) | n.c. | >30 |
| 19 | **14dn** | 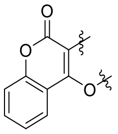 | 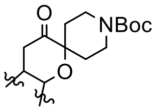 | 3 (50 µM) | n.c. | >30 |
| 20 | **14dl** | 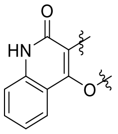 | 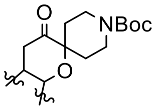 | 25 (50 µM) | 90 | 13.5 ± 1.2 |

**Table S5.** Biological evaluation of fragments of PFP **14dk** by means of the induction parameter delineated from the cell painting assay, and comparison with activity in the MitoSOX Red assay; $EC_{50}$ determined in HeLa cells (n = 3); n.c. means that biosimilarity was not calculated because induction was out of 20-40% comparison window.

| **Entry** | **Nr.** | **Fragment** | **Induction [%]** | **BioSim [%]** | **$EC_{50}$ [µM]** |
|---|---|---|---|---|---|
| 1 | **5p** | 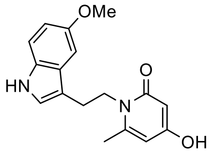 | 2 (50 µM) | n.c. | >30 |
| 2 | **8d** | 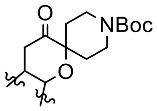 | 27 (10 µM) | 9 | 3.4 ± 0.1 |
| 3 | **8e** | 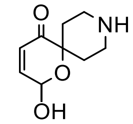 | 1 (50 µM) | n.c. | >30 |
| 4 | **5r** | 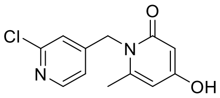 | 2 (50 µM) | n.c. | >30 |

## 4. Representative NMR Spectra

$^{1}$H-NMR of 5l

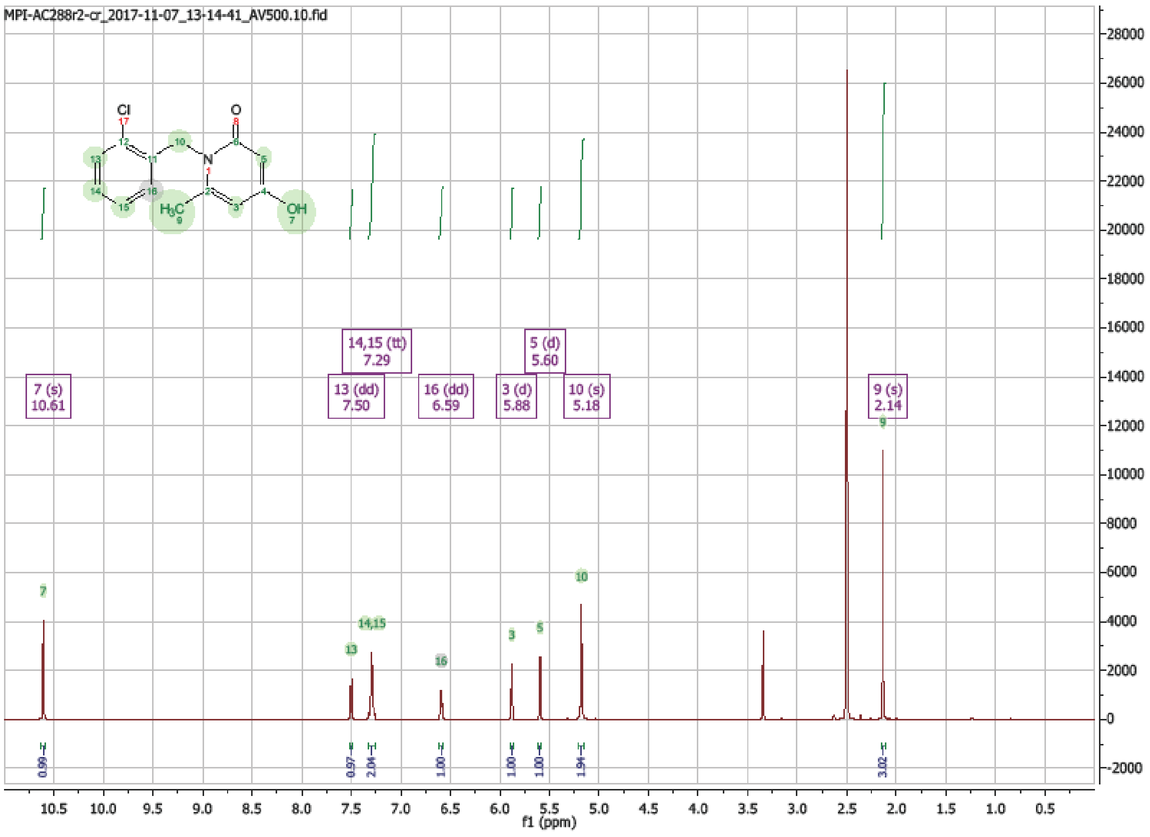


$^{13}$C-NMR of 5l

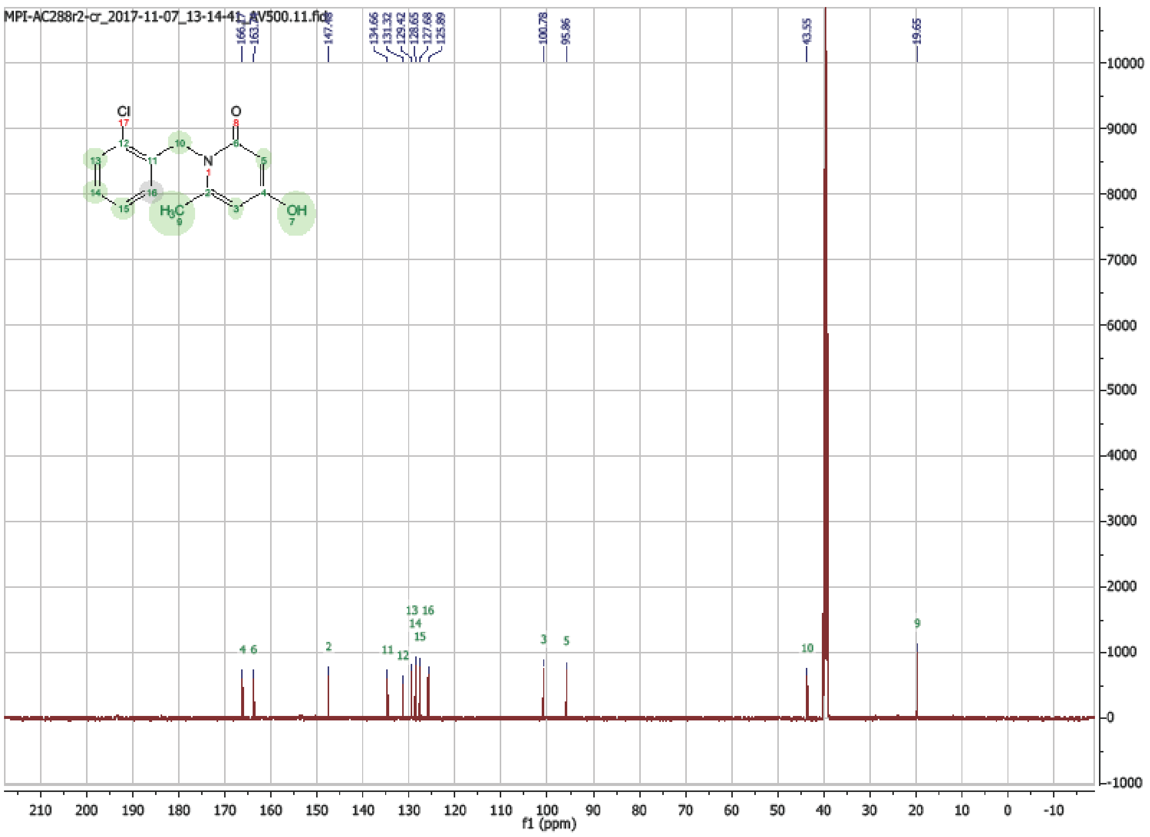

$^{1}$H-NMR of 7a

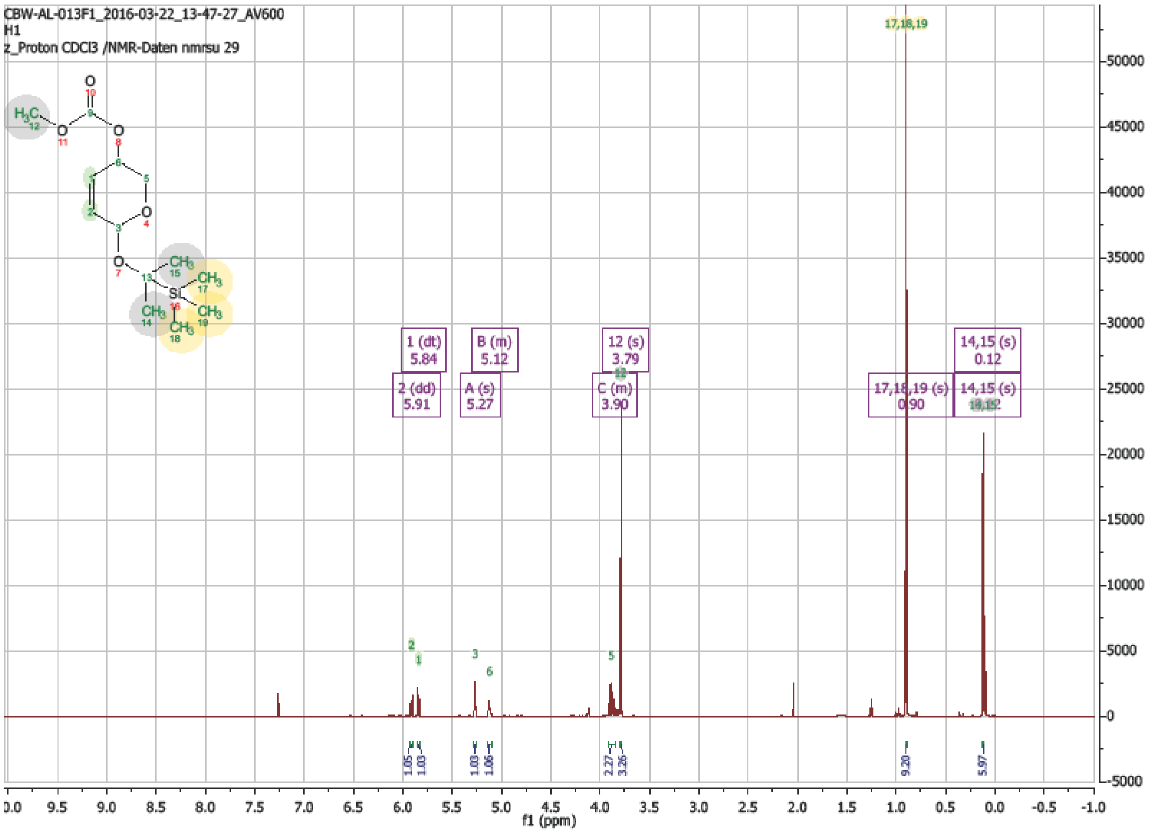


$^{13}$C-NMR of 7a

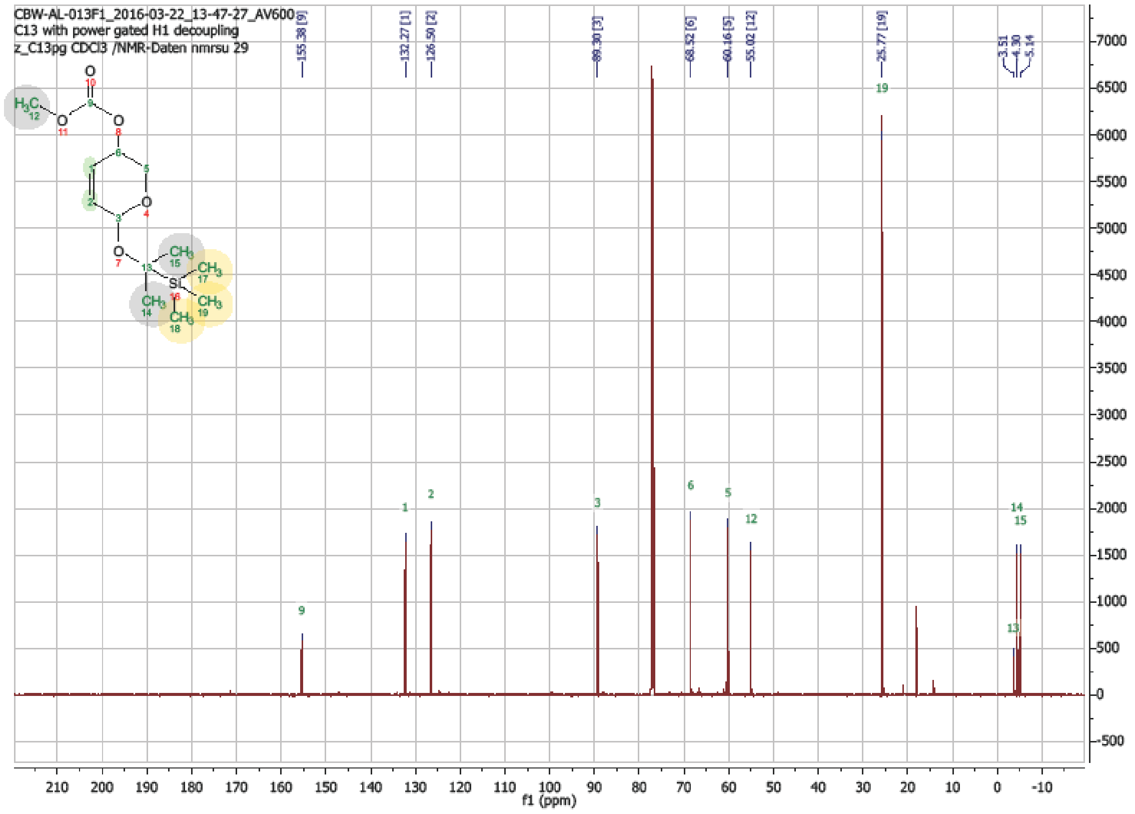

$^{1}$H-NMR of 8c

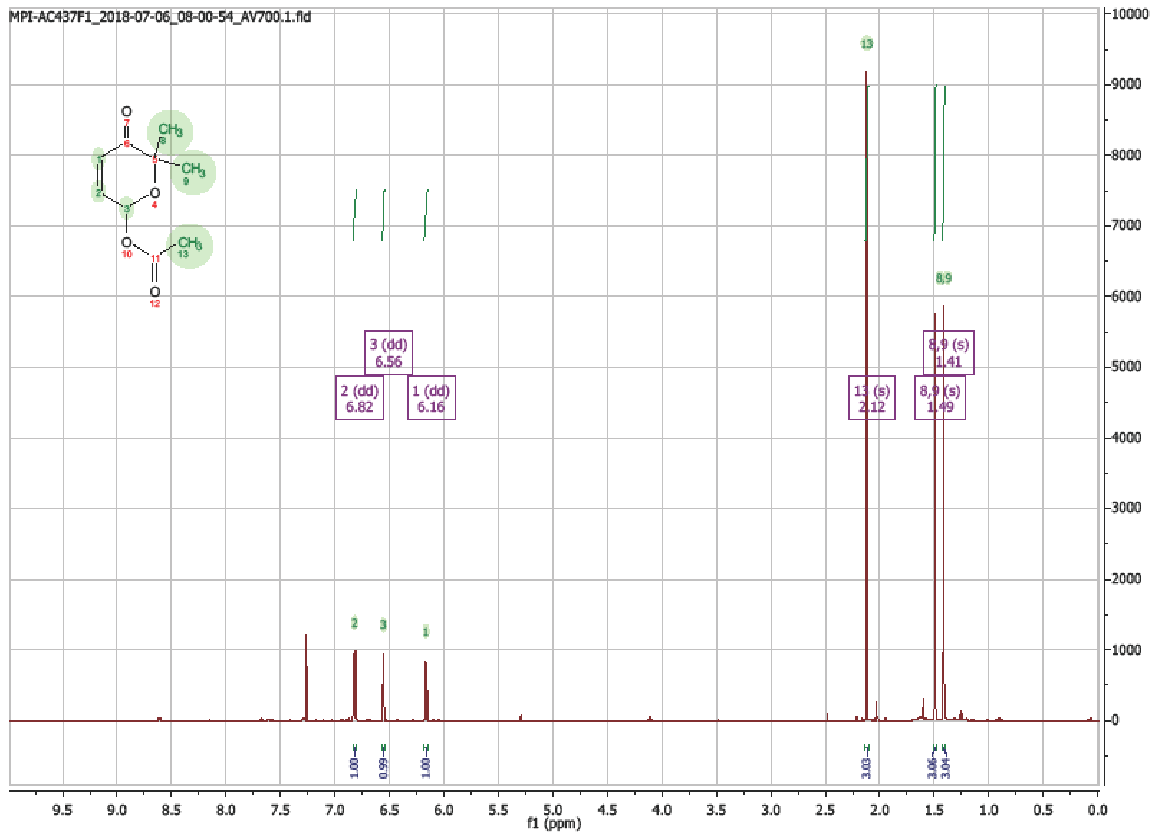


$^{13}$C-NMR of 8c

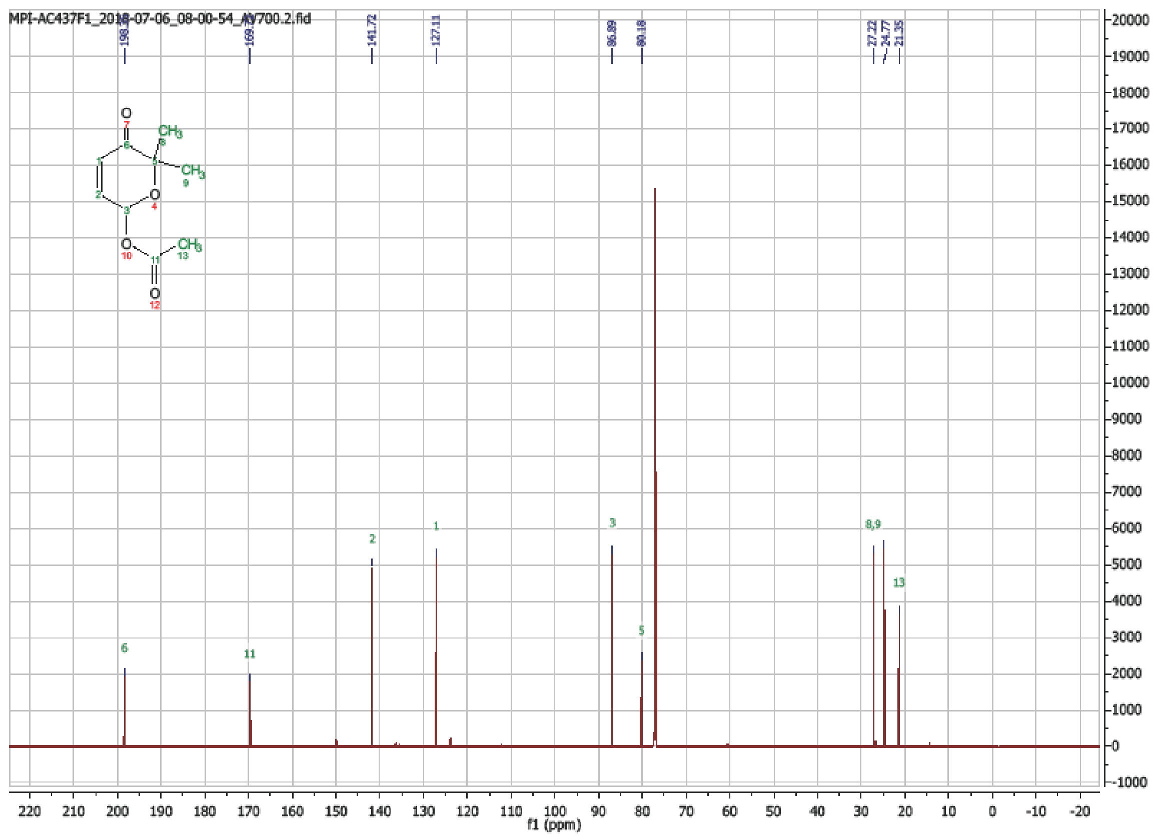

$^{1}$H-NMR of **9ai**

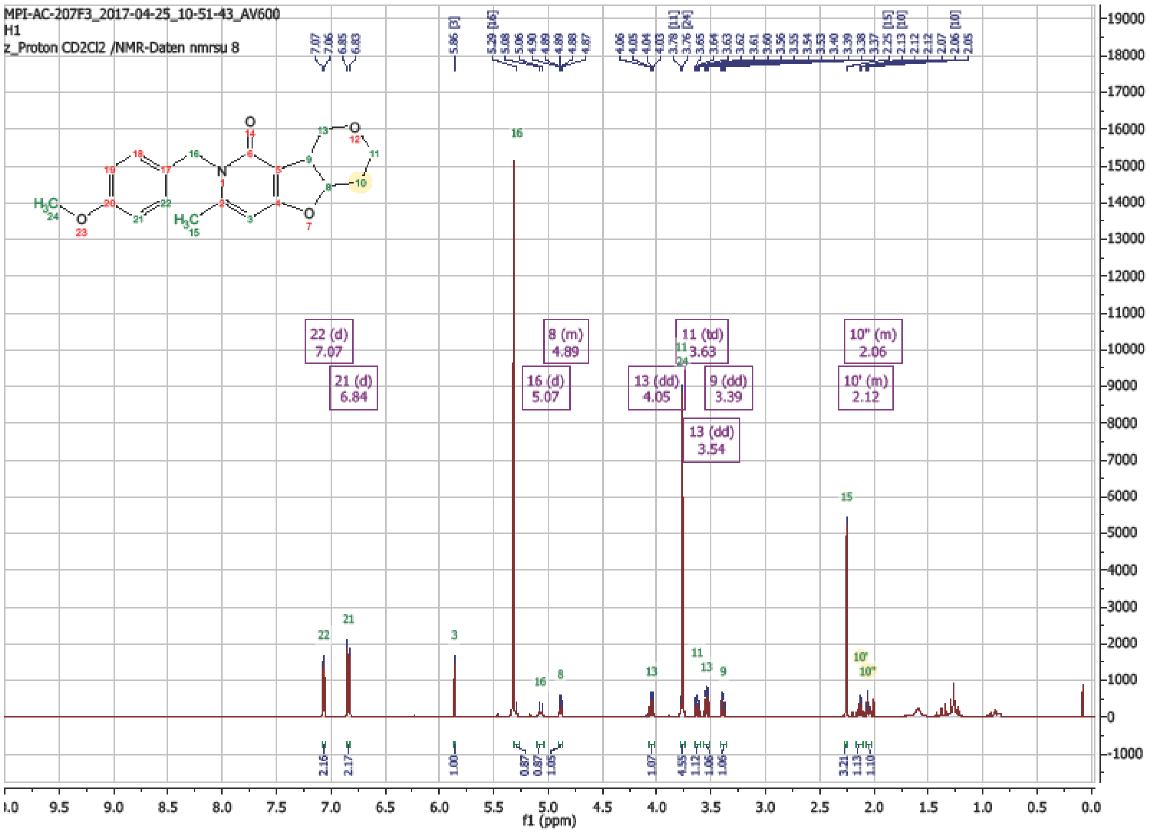

$^{13}$C-NMR of **9ai**

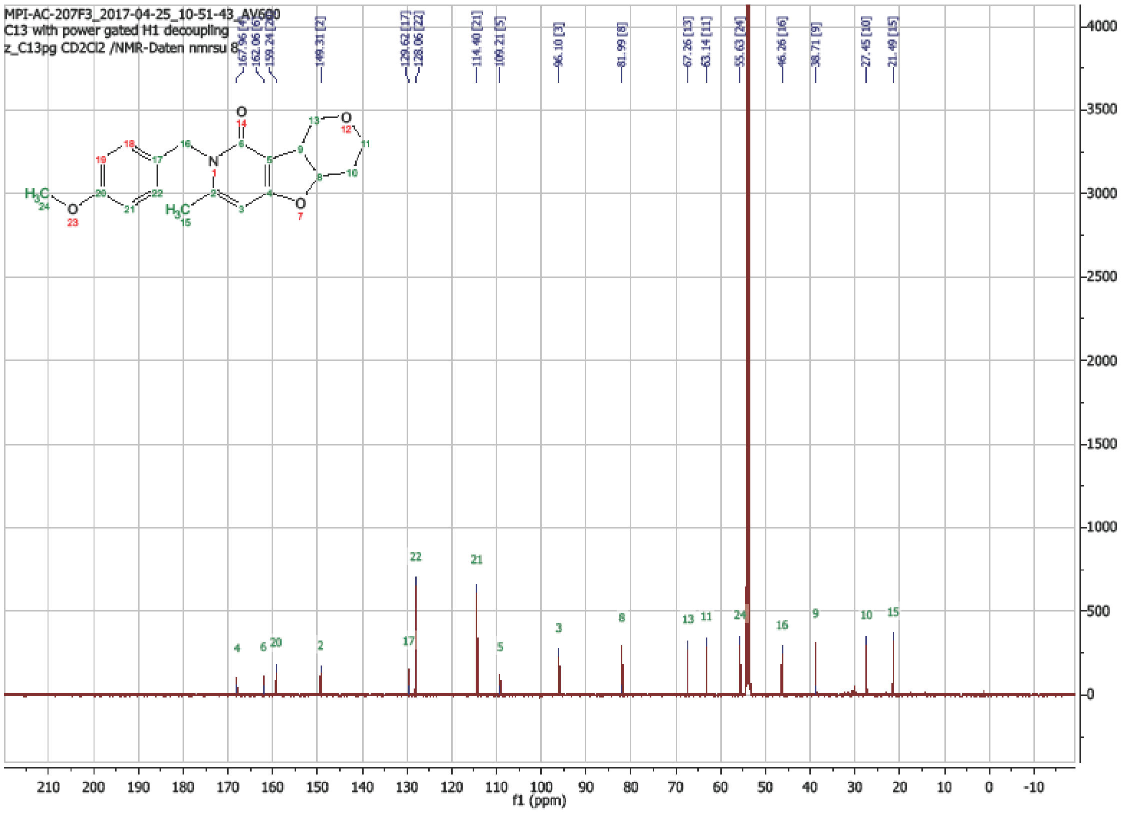

[1]**H-NMR** of **10bb**

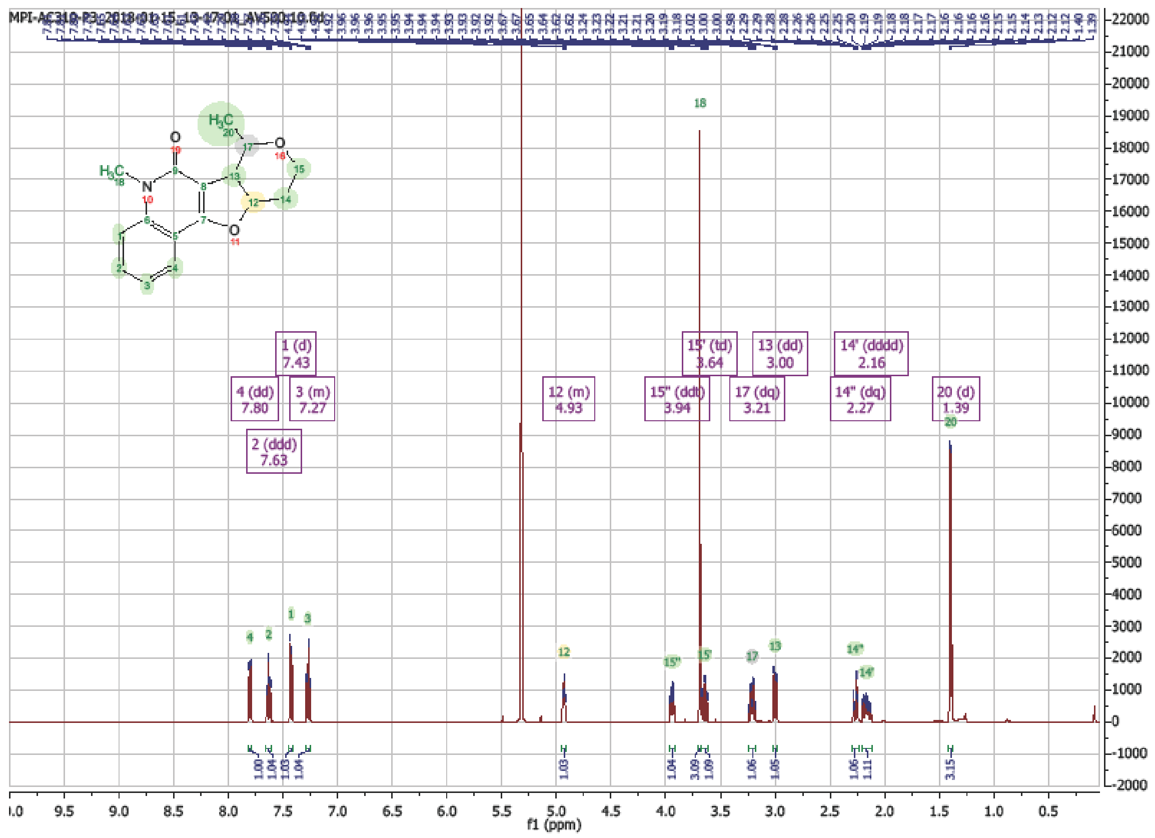

[13]**C-NMR** of **10bb**

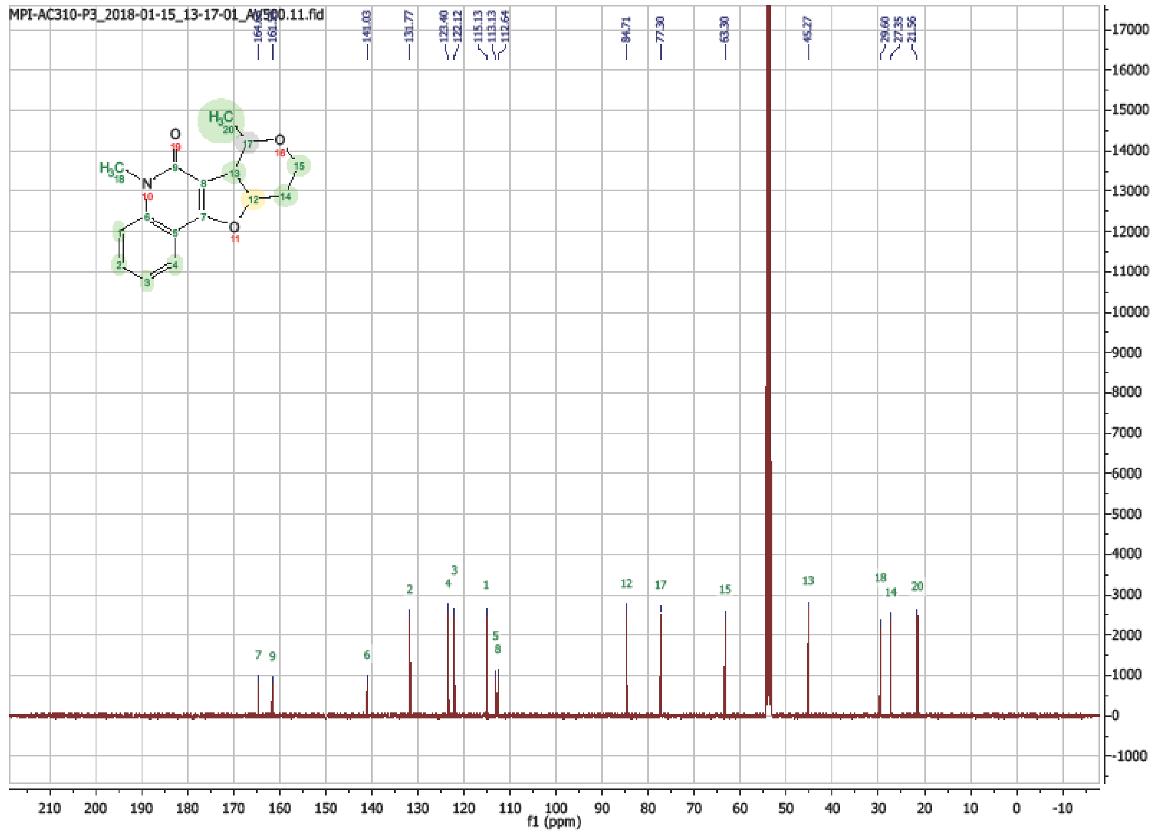

$^{1}$H-NMR of 15a

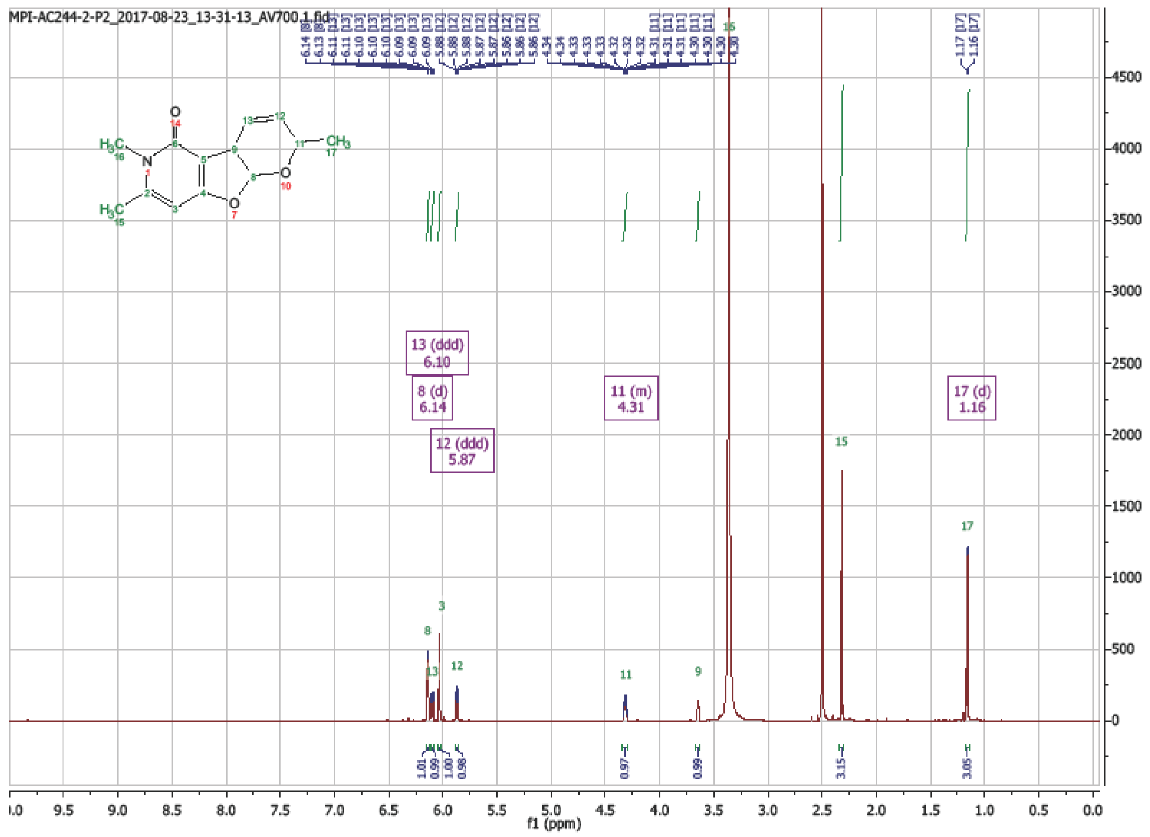

$^{13}$C-NMR of 15a

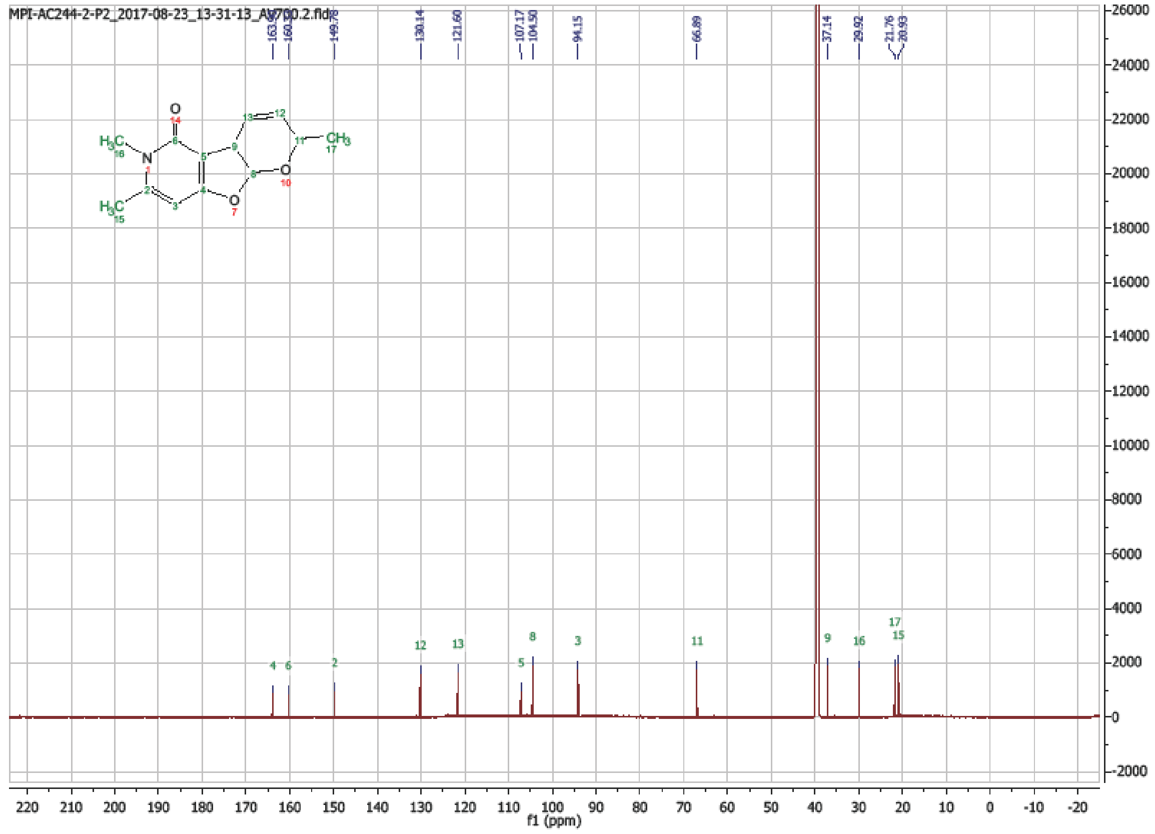

$^{1}$H-NMR of **13ab**

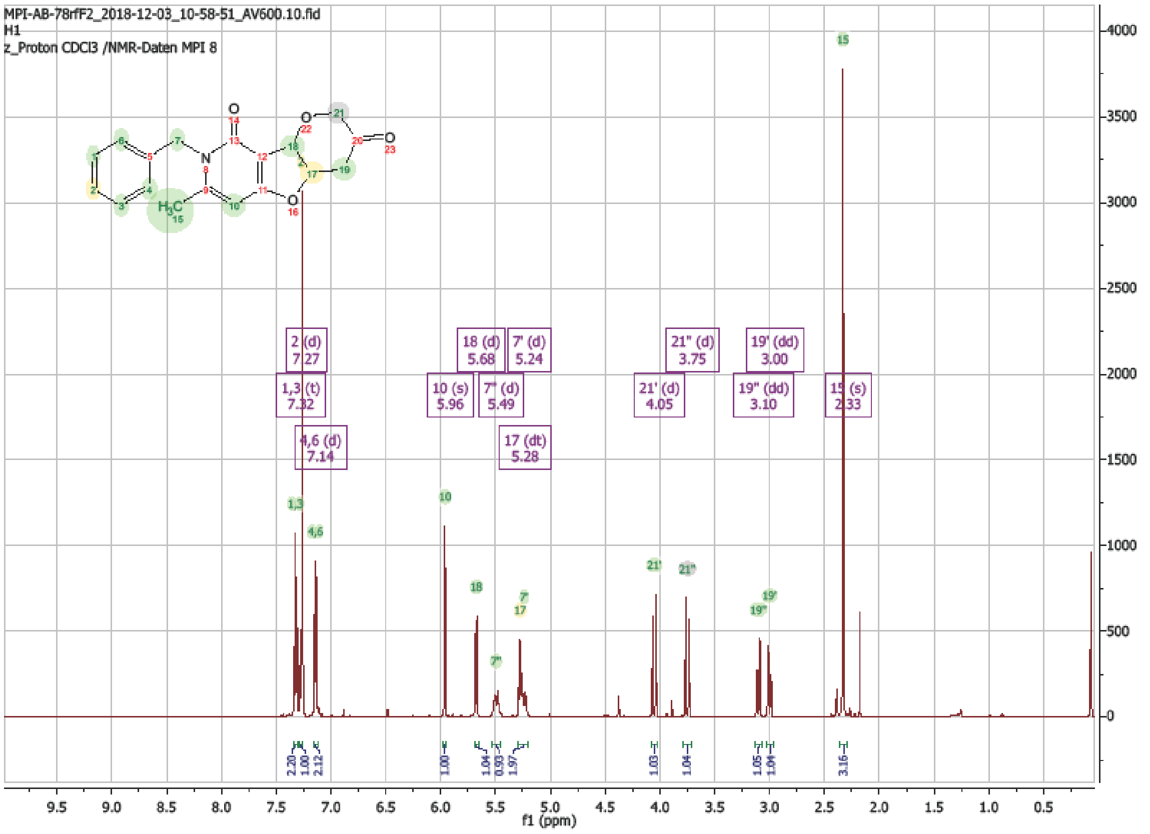


$^{13}$C-NMR of **13ab**

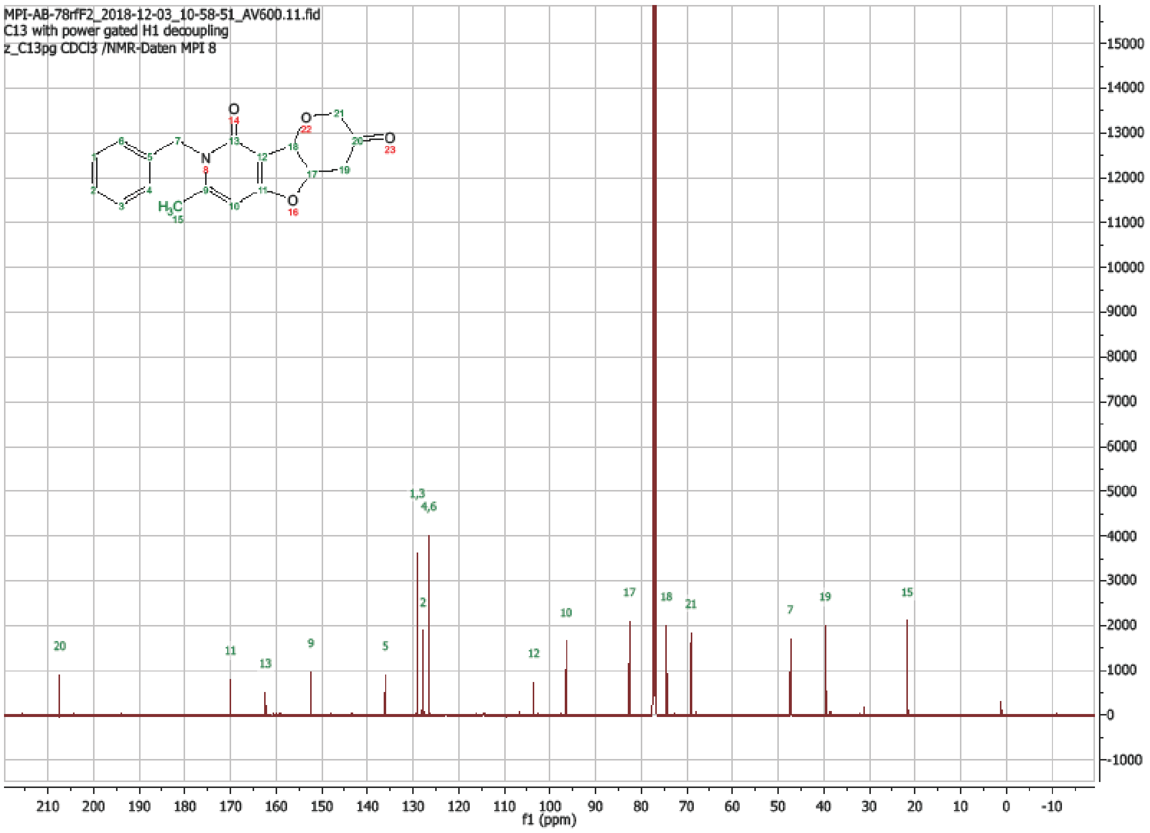

$^{1}$H-NMR of 13bi

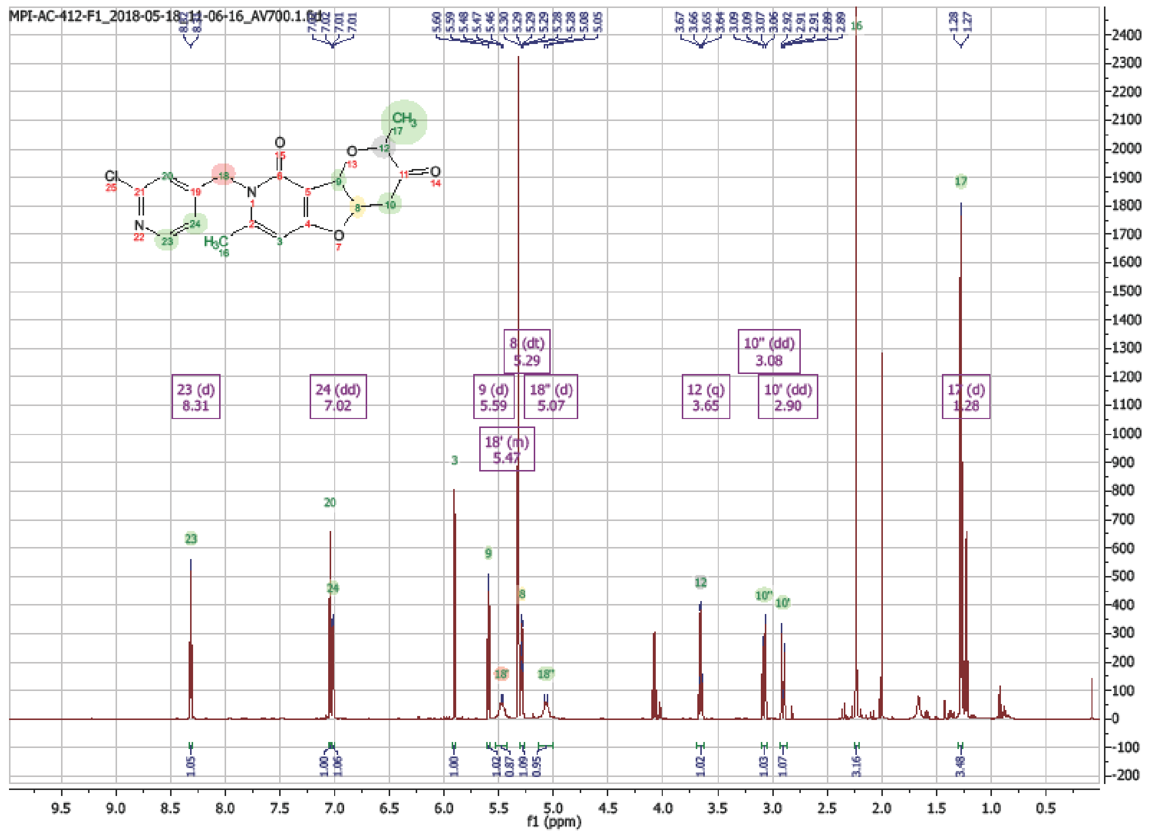

$^{13}$C-NMR of 13bi

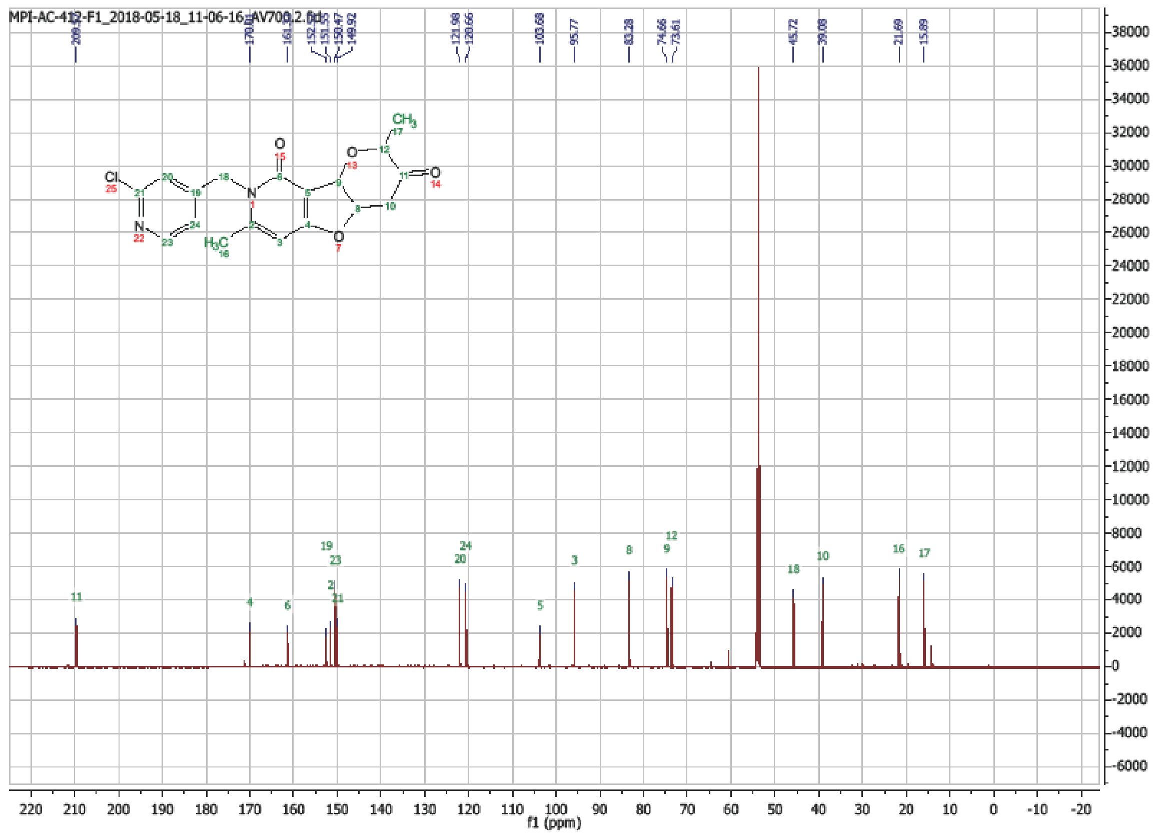

$^{1}$**H-NMR** of **13dd**

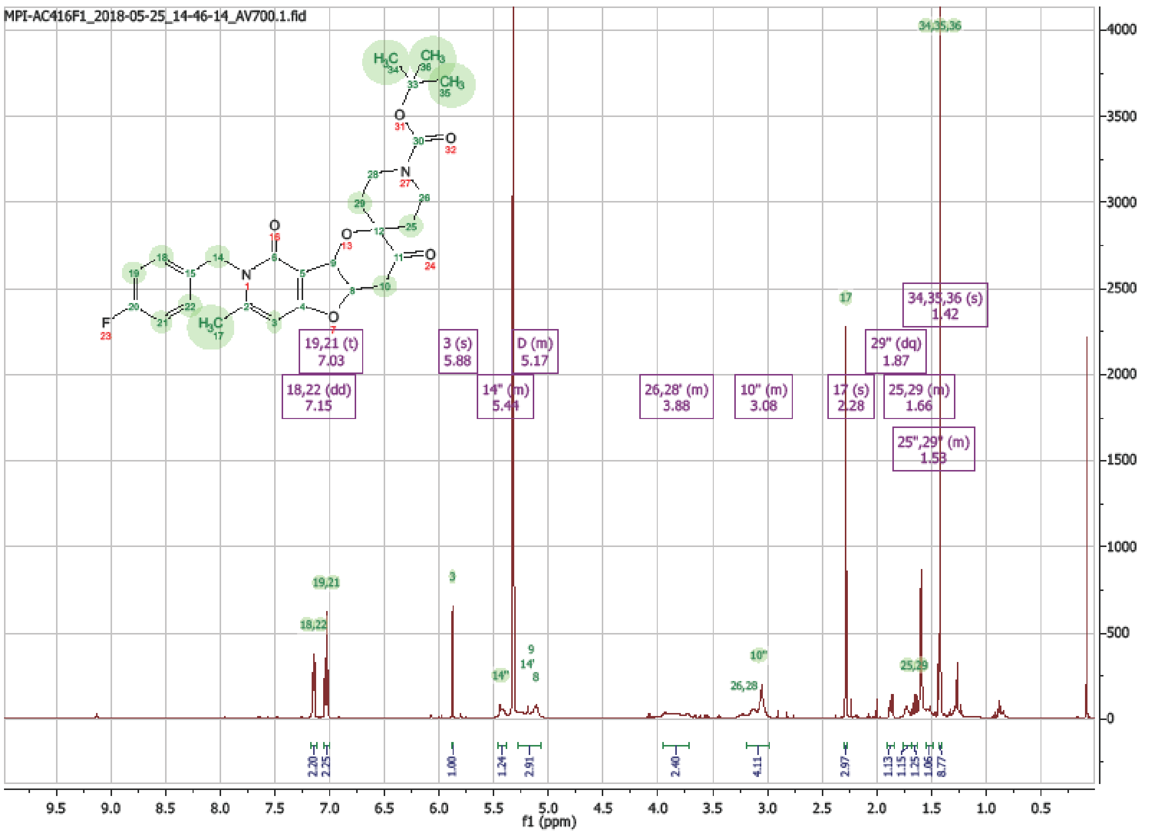


$^{13}$**C-NMR** of **13dd**

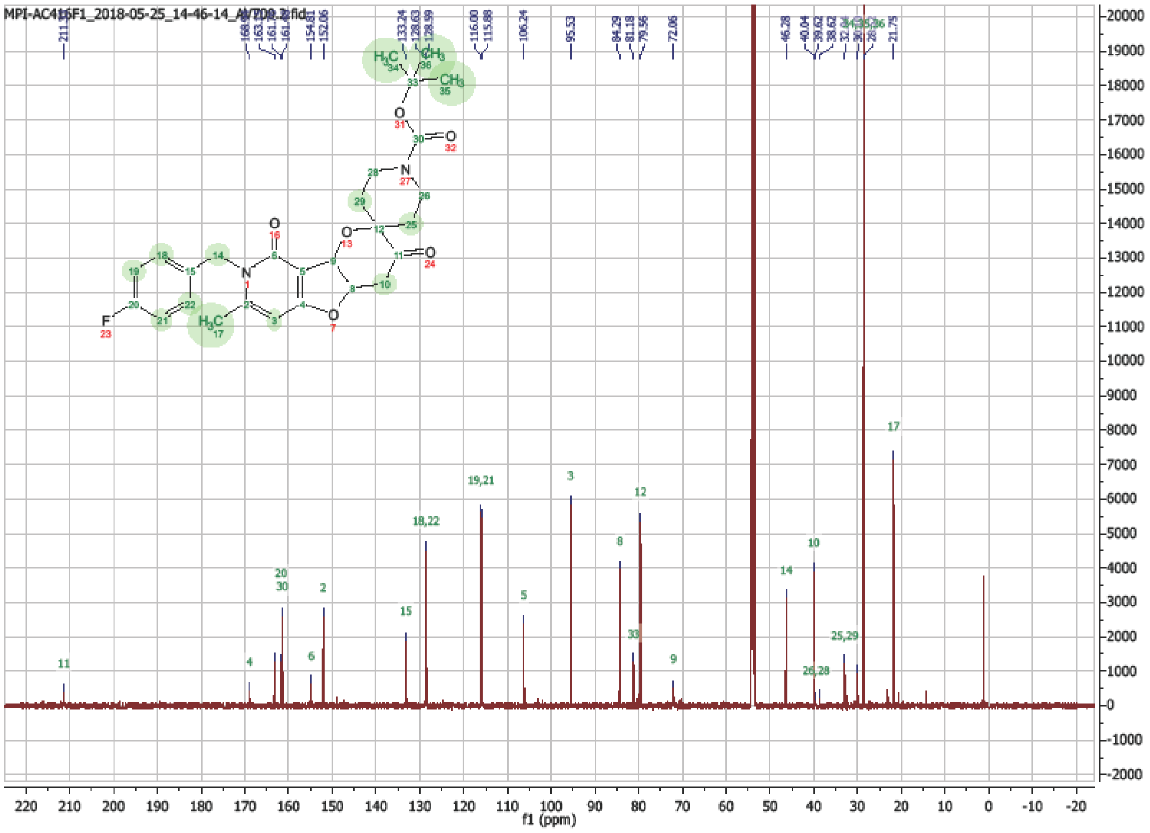

$^{1}$H-NMR of **18d**

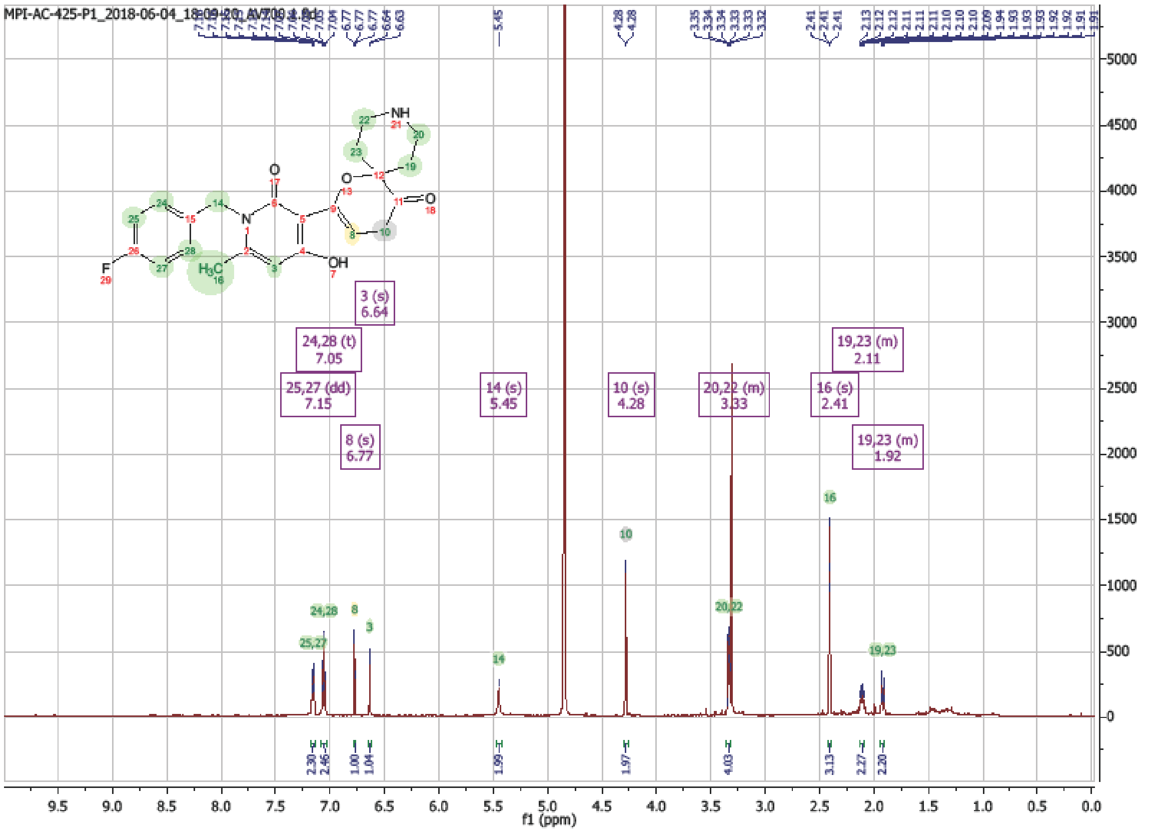


$^{13}$C-NMR of **18d**

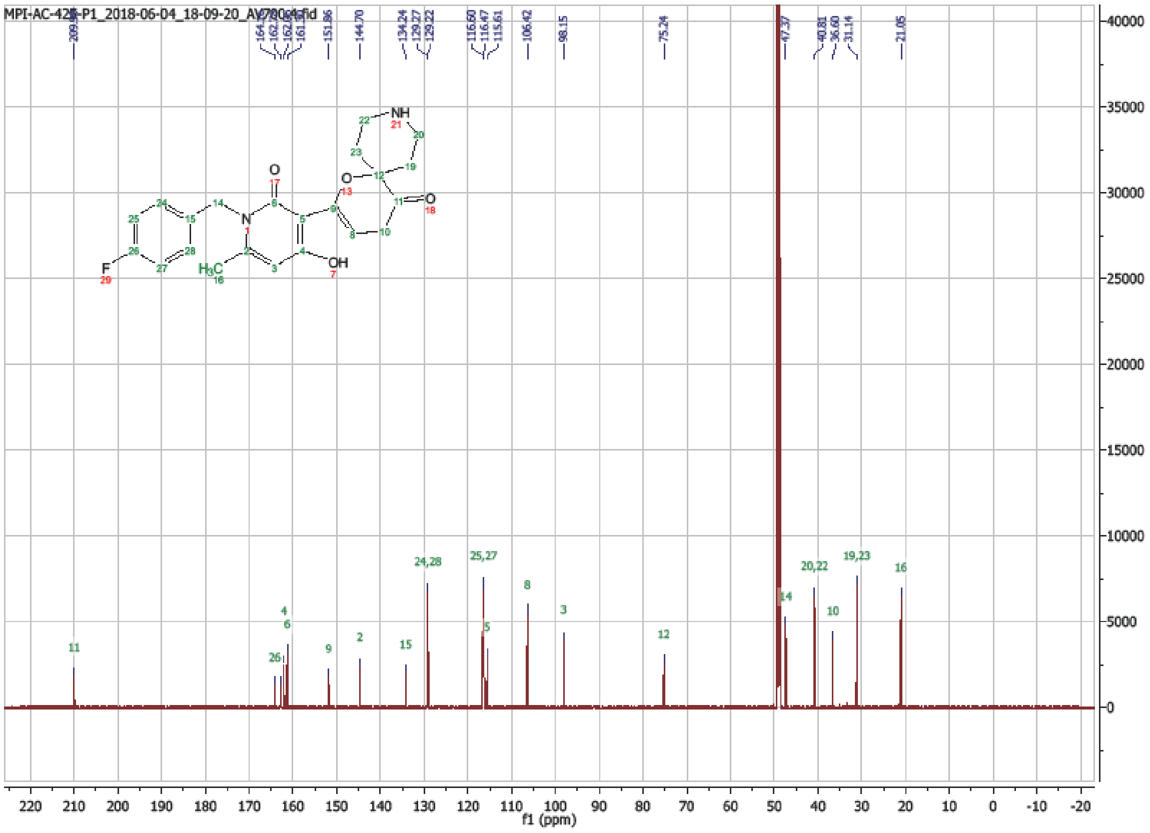

$^{1}$H-NMR of **13ej**

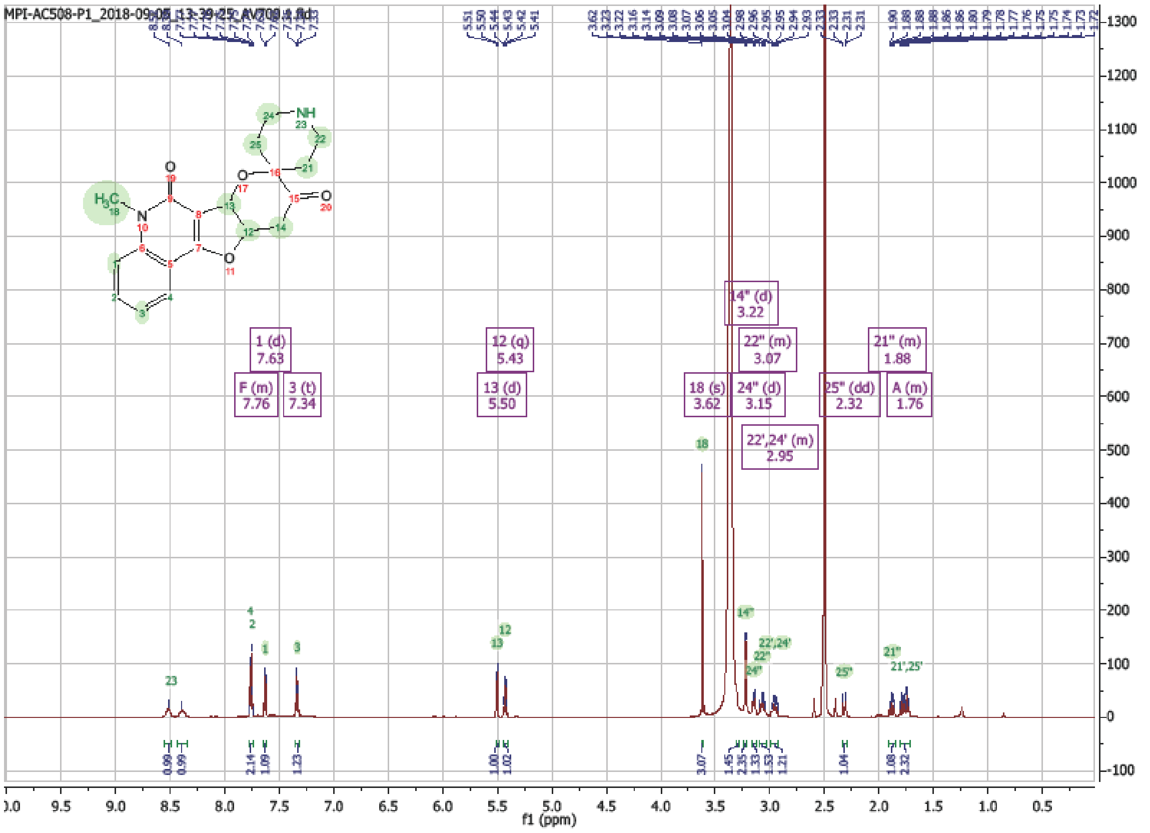

$^{13}$C-NMR of **13ej**

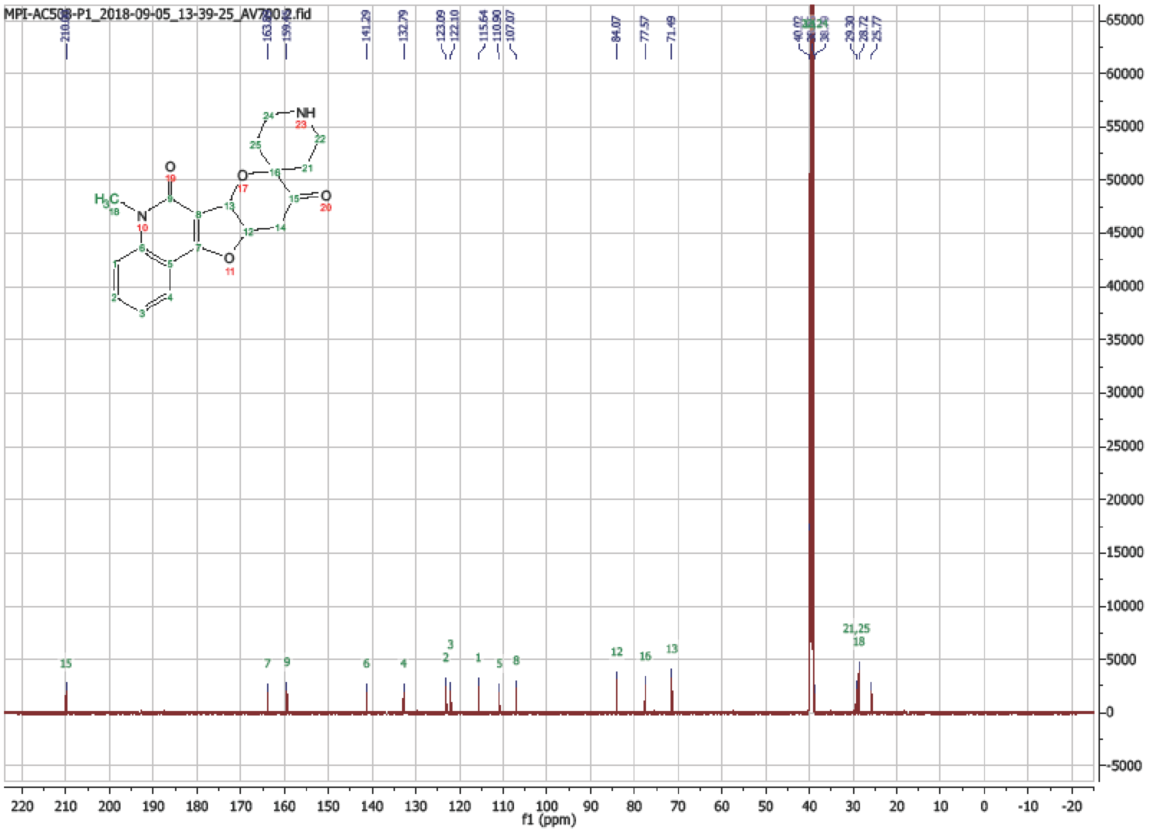

$^{1}$**H-NMR** of **13cj**

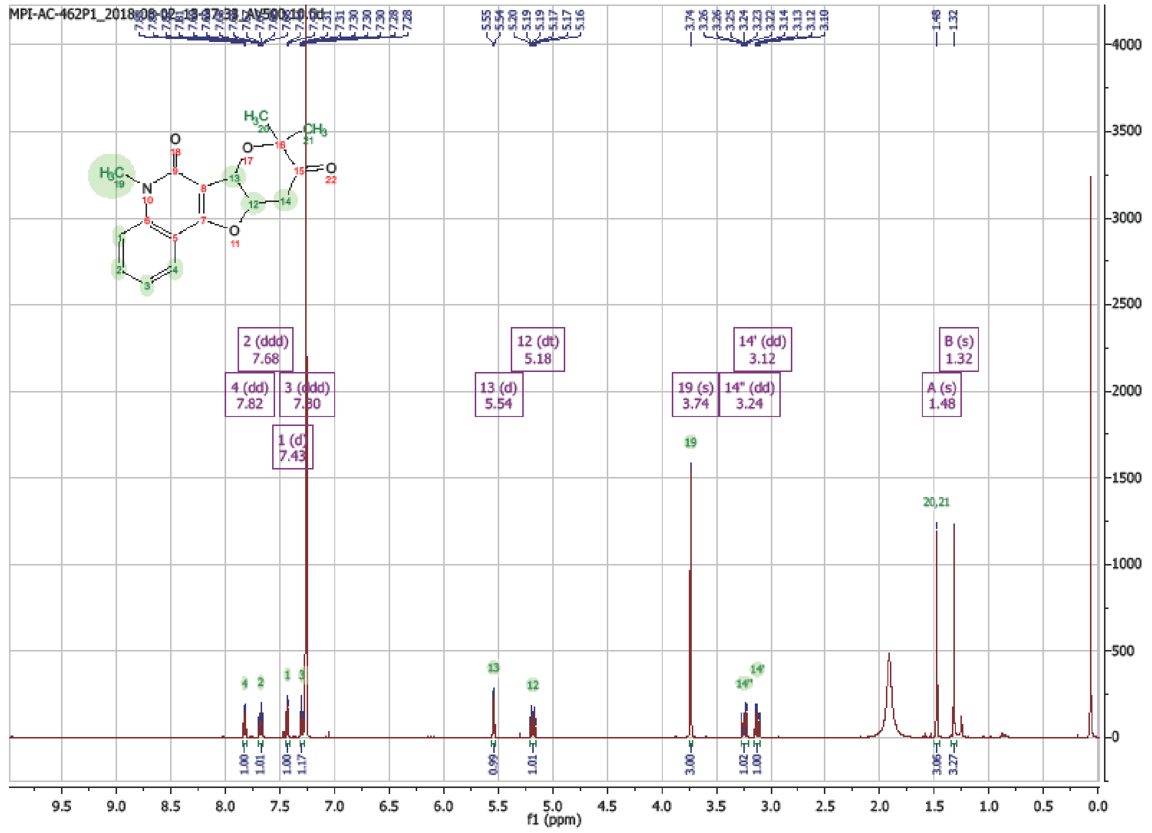


$^{13}$**C-NMR** of **13cj**

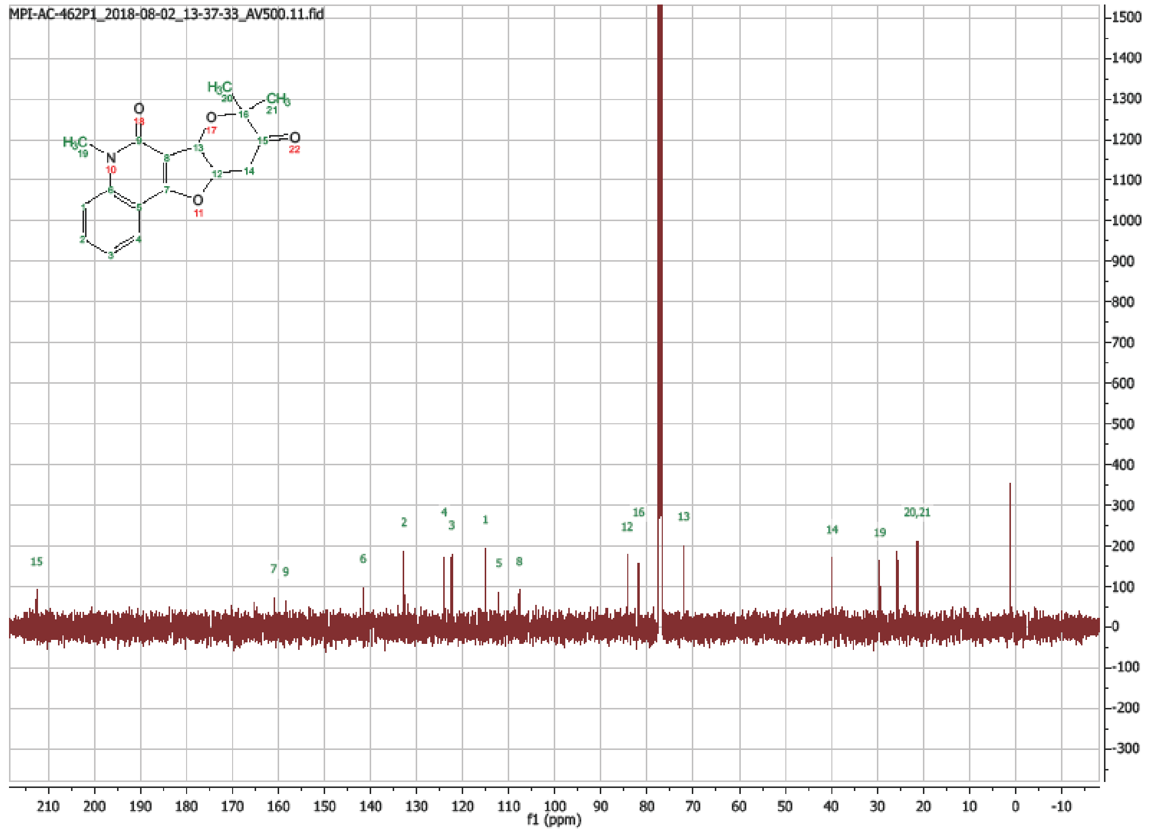

$^{1}$H-NMR of **14ae**

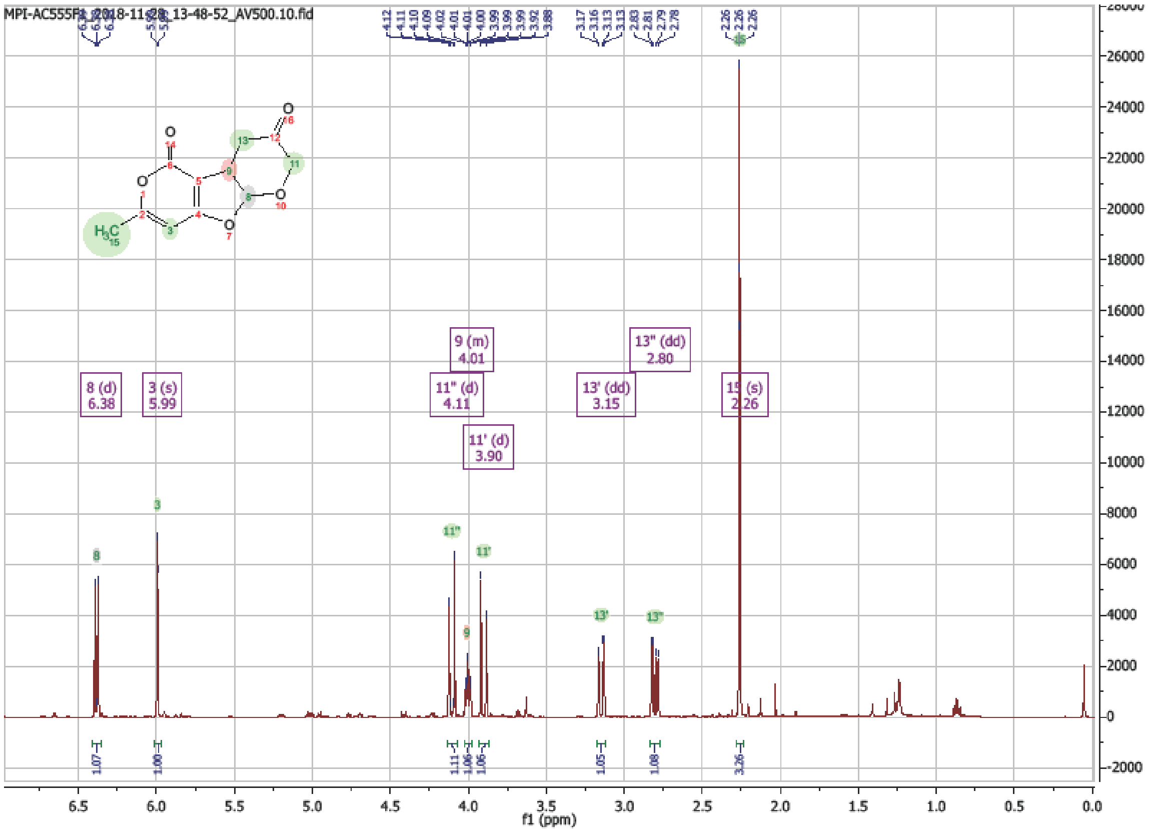


$^{13}$C-NMR of **14ae**

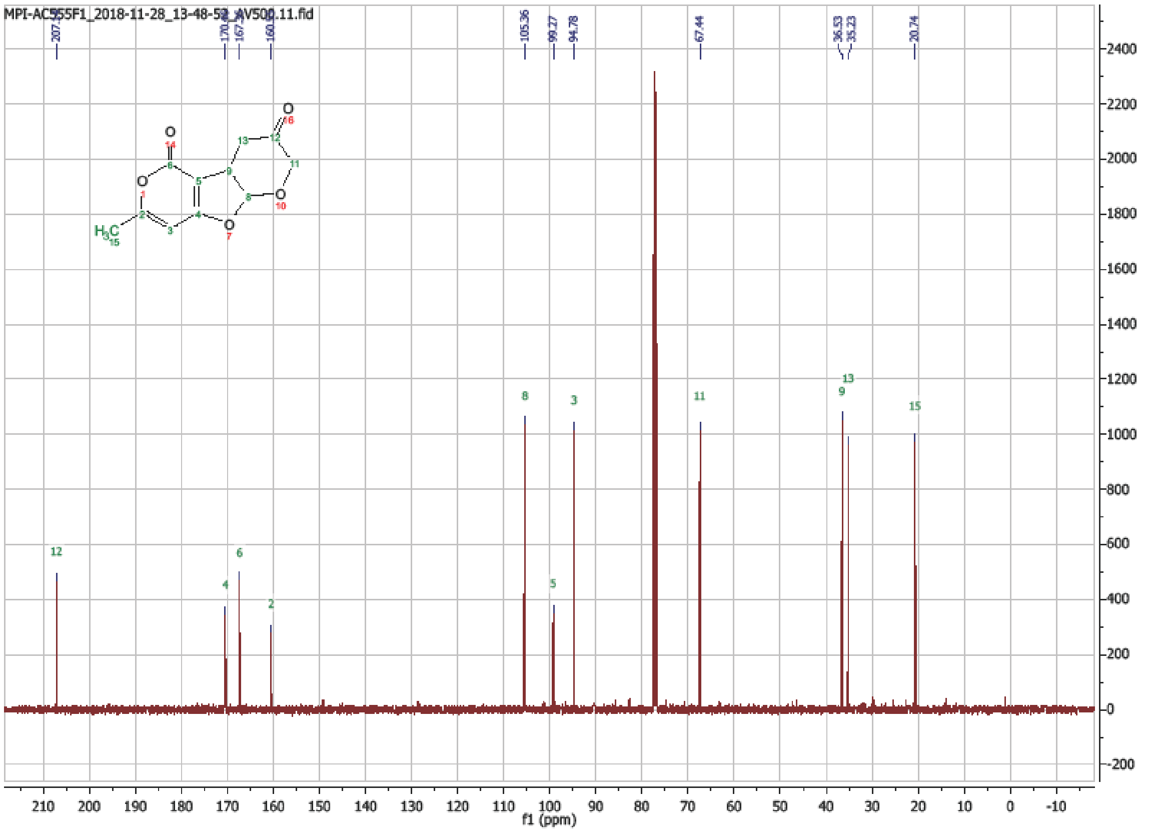

**$^{1}$H-NMR** of **14cc**

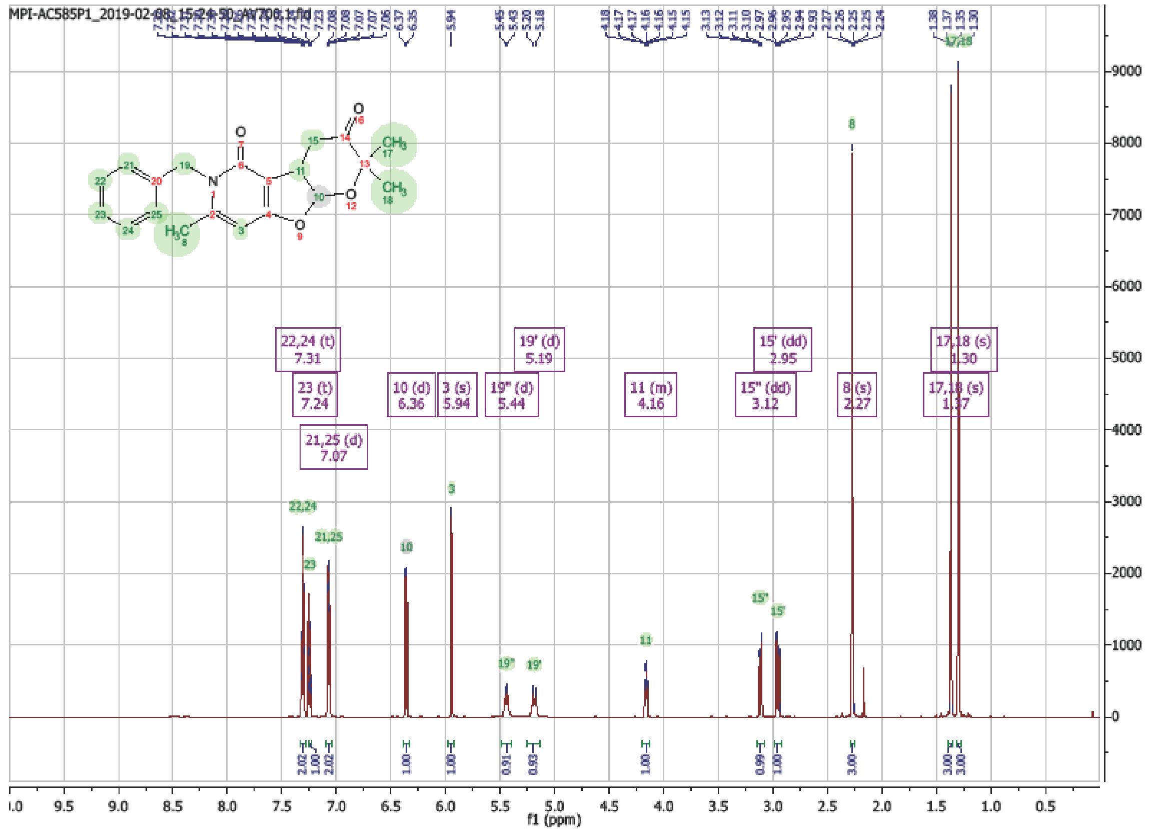


**$^{13}$C-NMR** of **14cc**

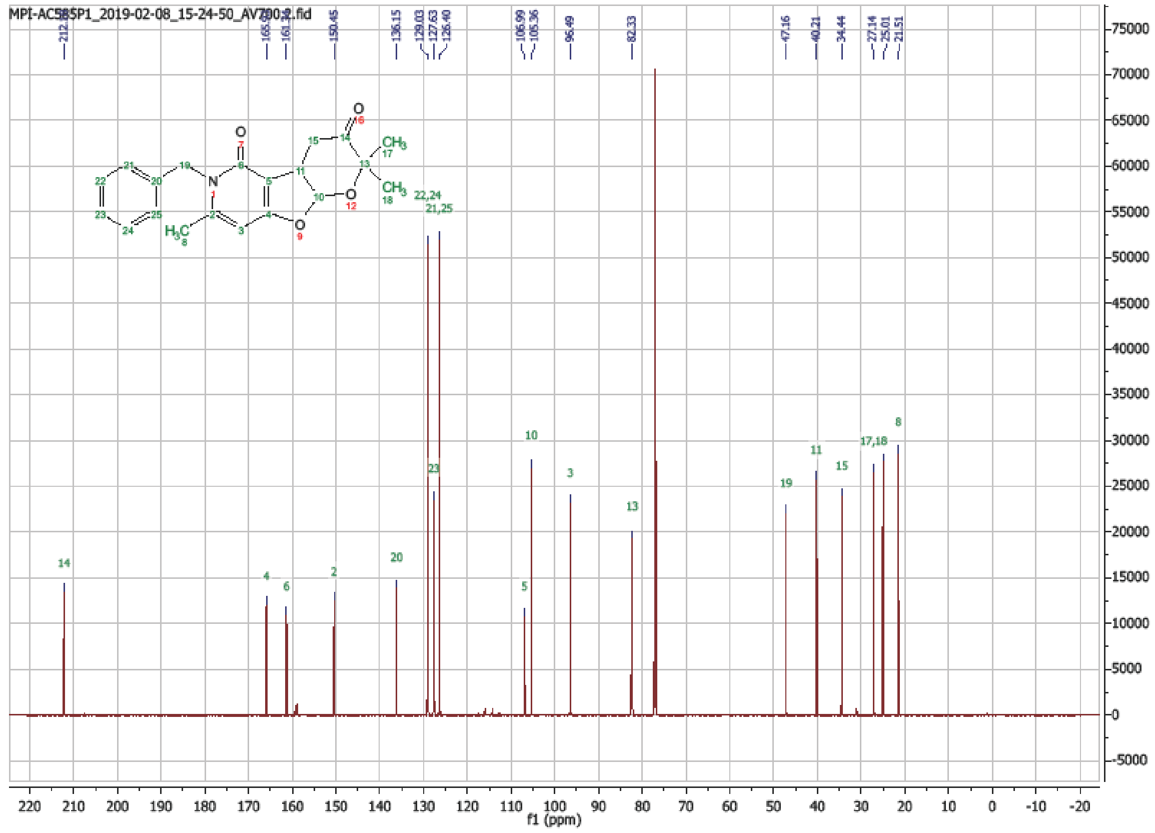

$^{1}$H-NMR of **14ed**

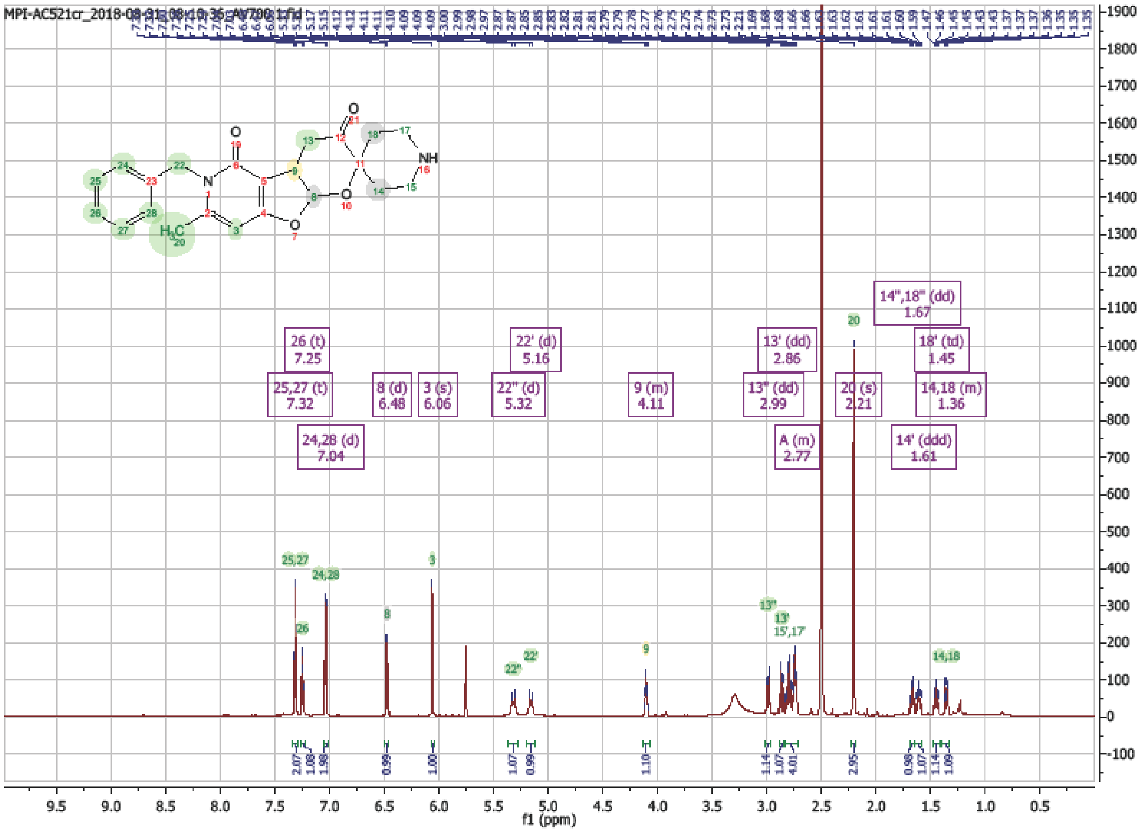


$^{13}$C-NMR of **14ed**

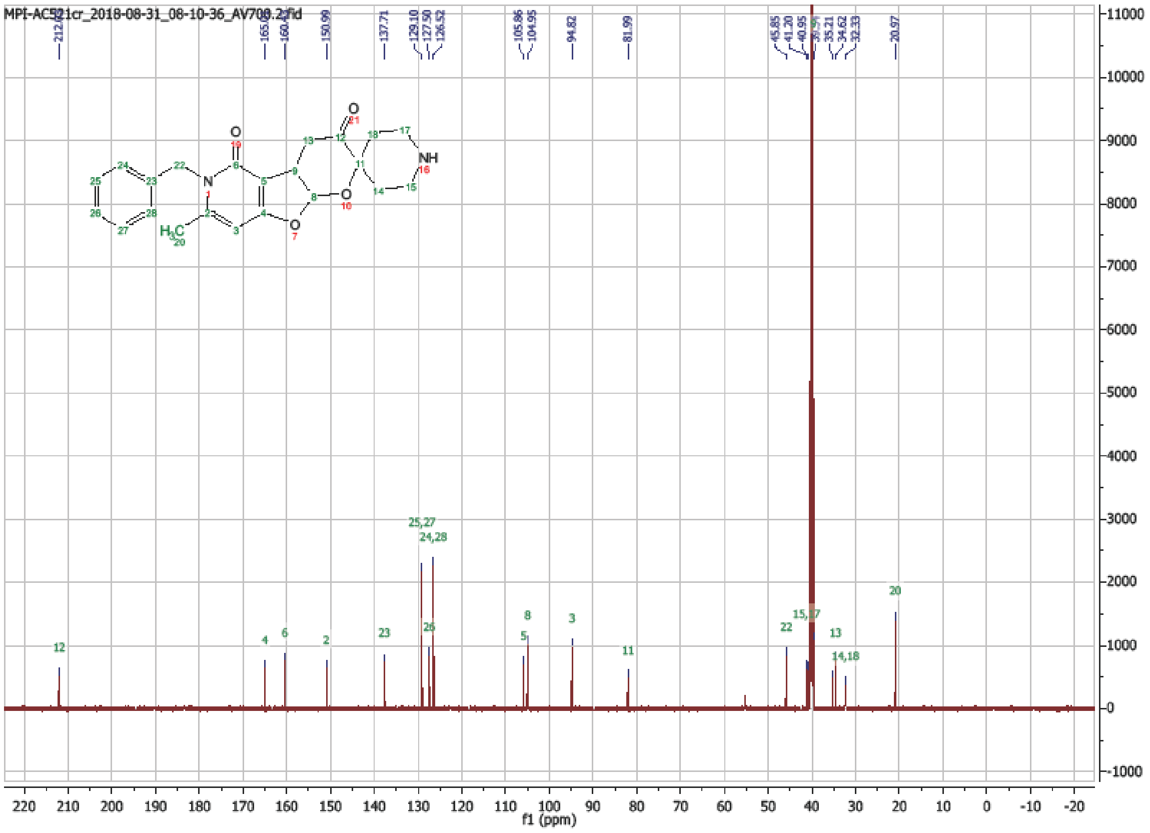

$^{1}$H-NMR of **14dk**

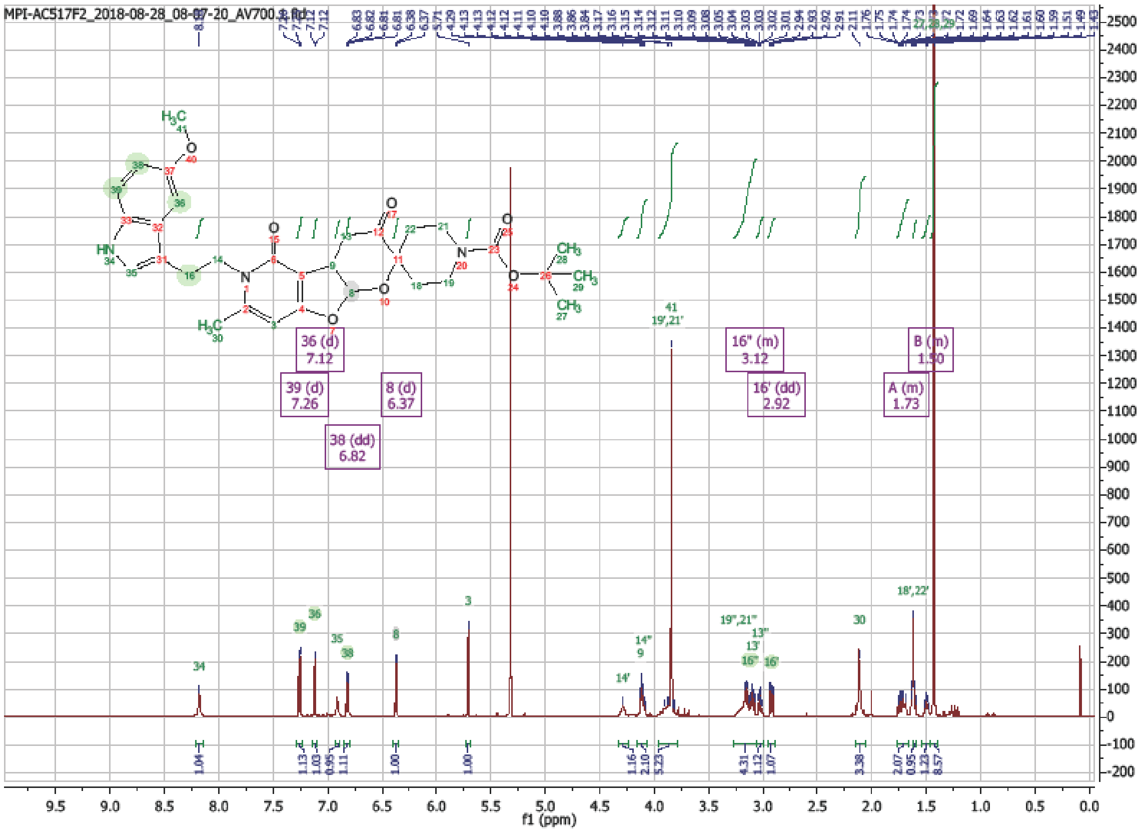


$^{13}$C-NMR of **14dk**

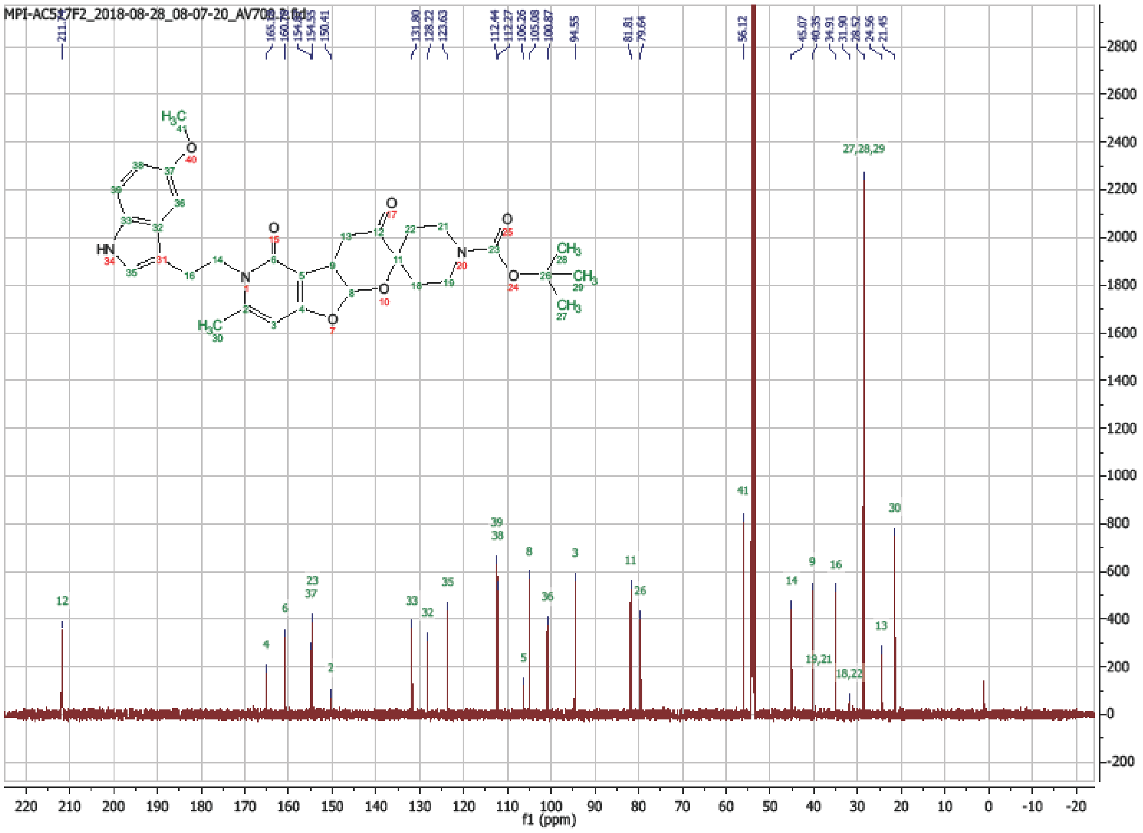

$^{1}$H-NMR of 12d

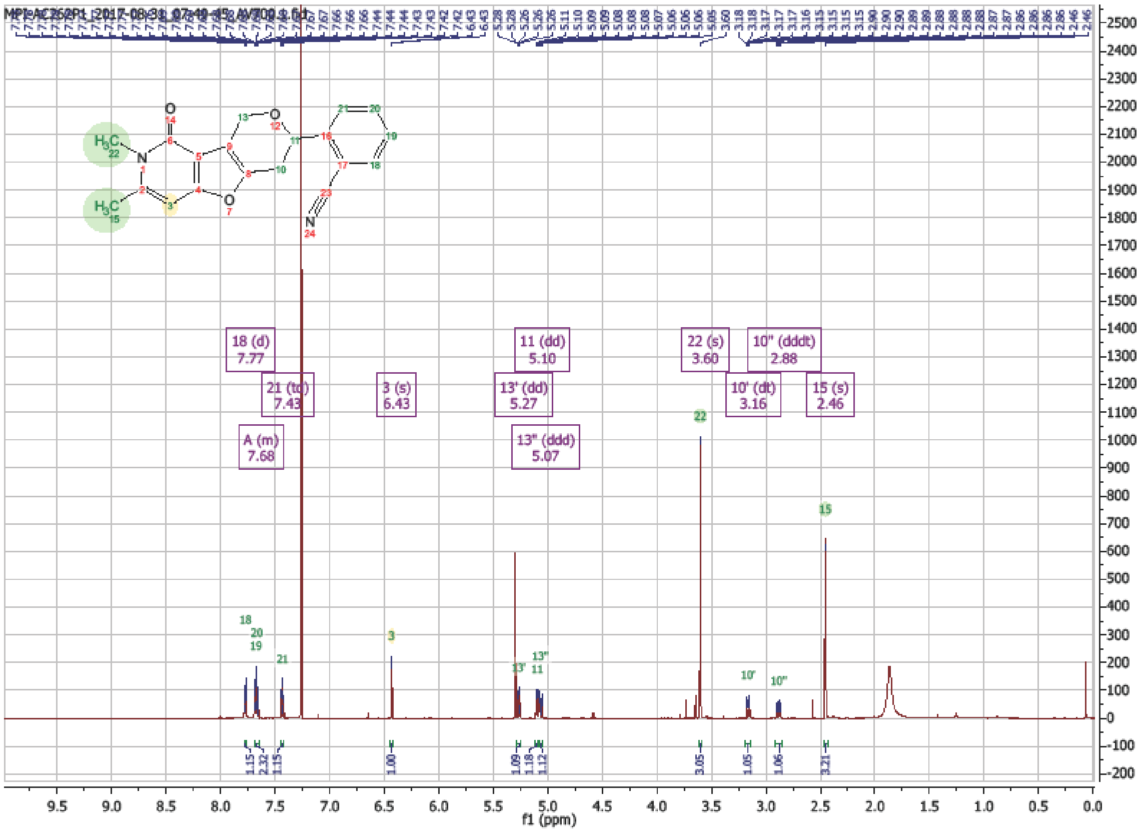

$^{13}$C-NMR of 12d

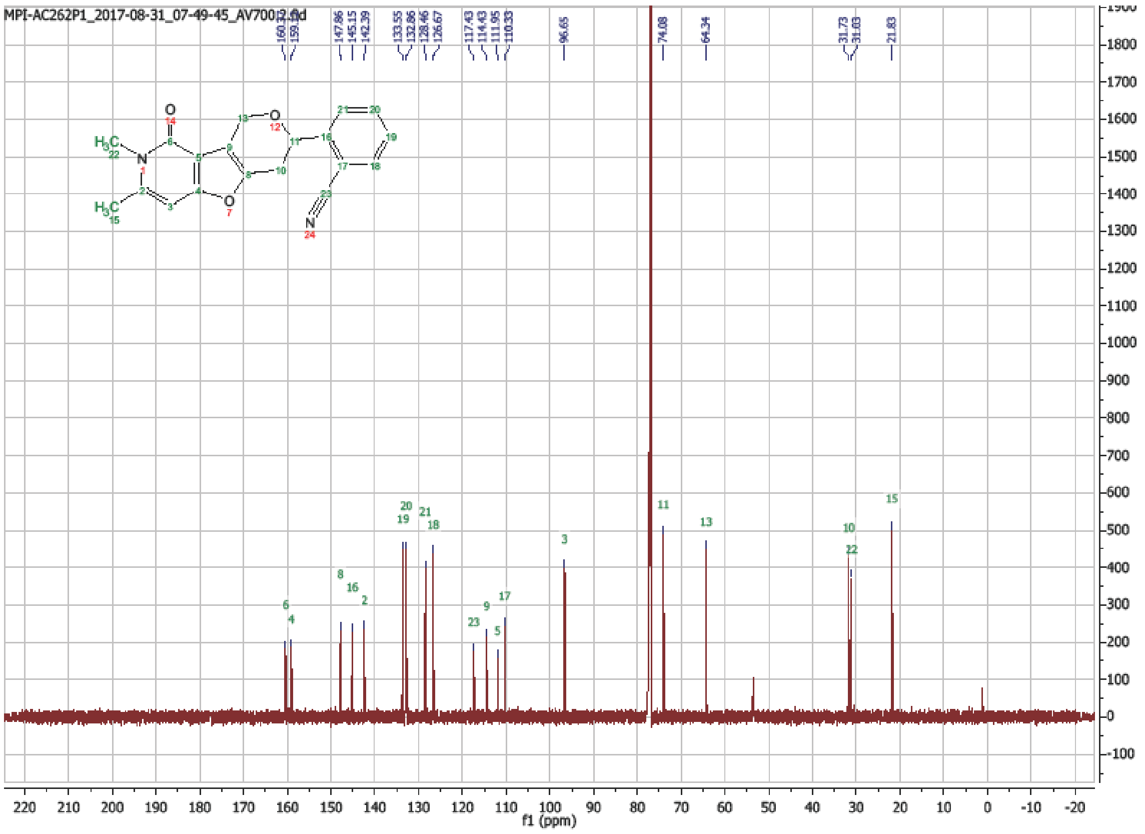

$^{1}$H-NMR of **17aa**

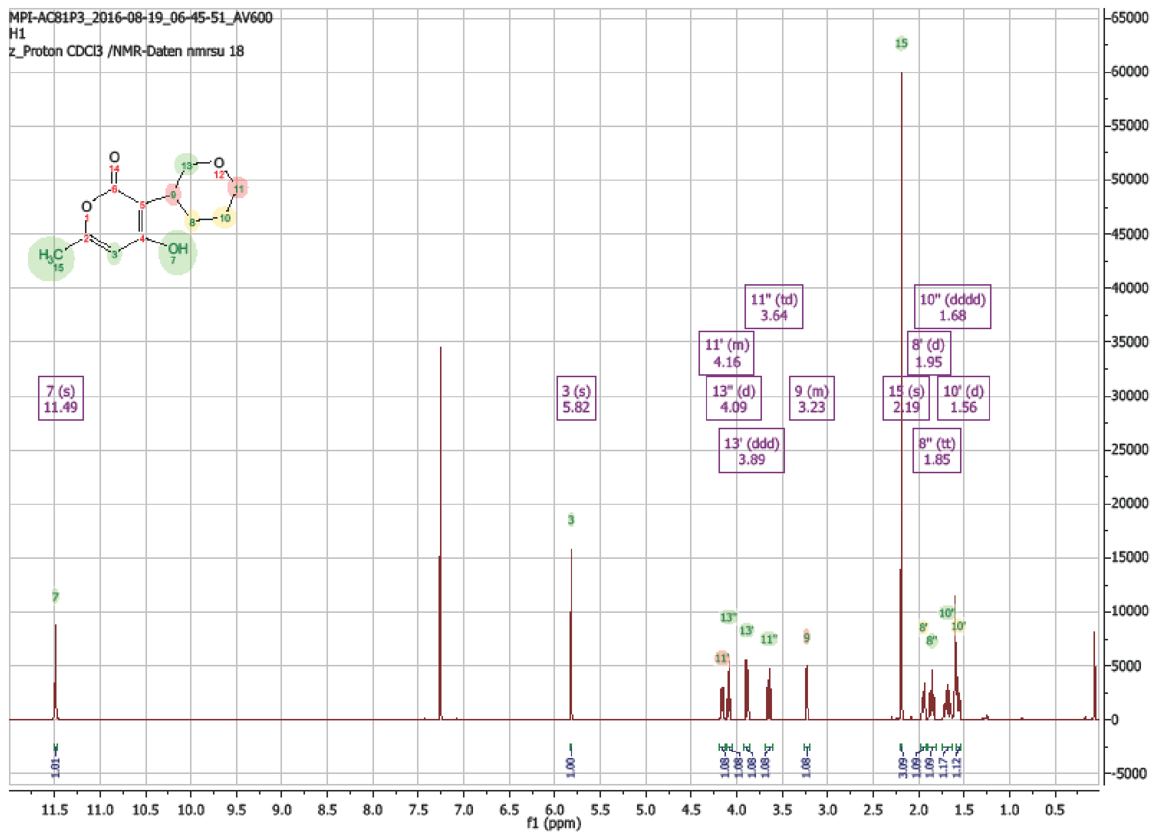


$^{13}$C-NMR of **17aa**

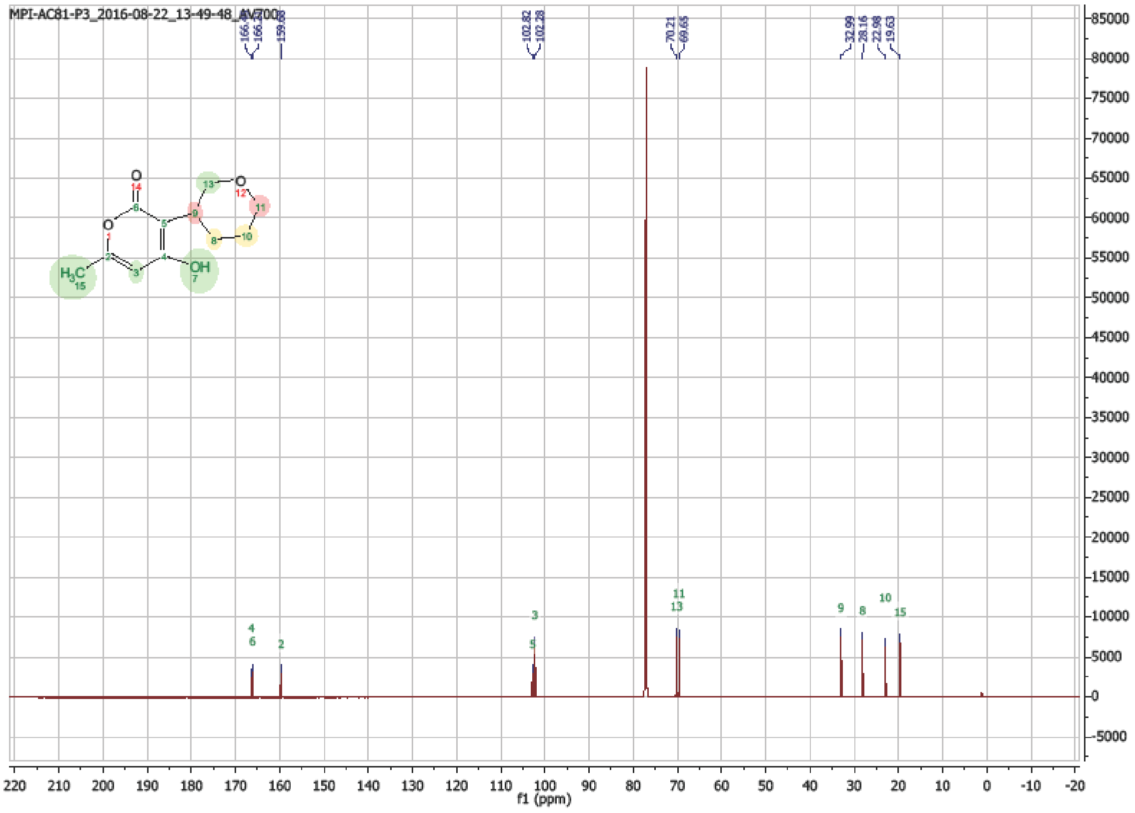

## 5. References


[1] G. R. Fulmer, A. J. M. Miller, H. N. Sherden, H. E. Gottlieb, A. Nudelman, B. M. Stoltz, J. E. Bercaw, K. I. Goldberg, *Organometallics* **2010**, *29*, 2176-2179.

[2] M. B. Plutschack, P. H. Seeberger, K. Gilmore, *Org. Lett.* **2017**, *19*, 30-33.

[3] P. Vincetti, F. Caporuscio, S. Kaptein, A. Gioiello, V. Mancino, Y. Suzuki, N. Yamamoto, E. Crespan, A. Lossani, G. Maga, G. Rastelli, D. Castagnolo, J. Neyts, P. Leyssen, G. Costantino, M. Radi, *J. Med. Chem.* **2015**, *58*, 4964-4975.

[4] B. H. Patel, A. M. Mason, A. G. M. Barrett, *Org. Lett.* **2011**, *13*, 5156-5159.

[5] M.-T. Nolan, J. T. W. Bray, K. Eccles, M. S. Cheung, Z. Lin, S. E. Lawrence, A. C. Whitwood, J. S. Fairlamb, G. P. McGlacken, *Tetrahedron* **2014**, *70*, 7120-7127.

[6] S. L. Clarke, G. P. McGlacken, *Tetrahedron* **2015**, *71*, 2906-2913.

[7] S. R. Selness, R. V. Devraj, J. B. Monahan, T. L. Boehm, J. K. Walker, B. Devadas, R. C. Durley, R. Kurumbail, H. Shieh, L. Xing, M. Hepperle, P. V. Rucker, K. D. Jerome, A. G. Benson, L. D. Marrufo, H. M. Madsen, J. Hitchcock, T. J. Owen, L. Christie, M. A. Promo, B. S. Hickory, E. Alvira, W. Naing, R. Blevis-Bal, *Bioorg. Med. Chem. Lett.* **2009**, *19*, 5851-5856.

[8] N. M. Przheval'skii, E. N. Rozhkova, *Doklady TSKhA* **2009**, *281*, 114-118.

[9] G. P. Tokmakov, N. M. Przheval'skii, E. N. Rozhkova, N. L. Nam, *Izvestiya Timiryazevskoi Sel'skokhozyaistvennoi Akademii* **2009**, *2*, 169-175.

[10] J. Vucicevic, T. Srdic-Rajic, M. Pieroni, J. M. M. Laurila, V. Perovic, S. Tassini, E. Azzali, G. Costantino, S. Glisic, D. Agbaba, M. Scheinin, K. Nikolic, M. Radi, N. Veljkovic, *Bioorg. Med. Chem.* **2016**, *24*, 3174-3183.

[11] G. Dannhardt, W. Meindl, S. Gussmann, S. Ajili, T. Kappe, *J. Med. Chem.* **1987**, *22*, 505-510.

[12] M. J. Bartlett, C. A. Turner, J. E. Harvey, *Org. Lett.* **2013**, *15*, 2430-2433.

[13] H. Takayama, Z.-J. Jia, L. Kremer, J. Bauer, C. Strohmann, S. Ziegler, A. P. Antonchick, H. Waldmann, *Angew. Chem. Int. Ed.* **2013**, *52*, 12404-12408.

[14] O. Achmatowicz, P. Bukowski, B. Szechner, Z. Zwierzchowska, A. Zamojski, *Tetrahedron* **1971**, *21*, 1973-1996.

[15] C. Zhao, D. A. Glazier, D. Yang, D. Yin, I. A. Guzei, M. M. Aristov, P. Liu, W. Tang, *Angew. Chem. Int. Ed.* **2019**, *58*, 887-891.

[16] W.-Z. Song, N. Zheng, M. Li, K. Ullah, J.-H. Li, K. Dong, Y.-B. Zheng, *Heterocycles* **2018**, *96*, 1779-1785.

[17] J. Yu, H. Ma, H. Yao, G. Cheng, R. Tong, *Org. Chem. Front.* **2016**, *3*, 714-719.

[18] M.-A. Bray, S. Singh, H. Han, C. T. Davis, B. Borgeson, C. Hartland, M. Kost-Alimova, S. M. Gustafsdottir, C. C. Gibson, A. E. Carpenter, *Nat. Protoc.* **2016**, *11*, 1757-1774.

[19] M. H. Woehrmann, W. M. Bray, J. K. Durbin, S. C. Nisam, A. K. Michael, E. Glassey, J. M. Stuart, R. S. Lokey, *Mol. BioSyst.* **2013**, *9*, 2604-2617.

[20] L. Robke, Y. Futamura, G. Konstantinidis, J. Wilke, H. Aono, M. Zhwan, N. Watanabe, Y.-W. Wu, H. Osada, L. Laraia, H. Waldmann, *Chem. Sci.* **2018**, *9*, 3014-3022.

[21] G. Karageorgis, E. S. Reckzeh, J. Ceballos, M. Schwalfenberg, S. Sievers, C. Ostermann, A. Pahl, S. Ziegler, H. Waldmann, *Nat. Chem.* **2018**, *10*, 1103-1111.

[22] S. Brand, S. Roy, P. Schröder, B. Rathmer, J. Roos, S. Kapoor, S. Patil, C. Pommerenke, T. Maier, P. Janning, S. Eberth, D. Steinhilber, D. Schade, G. Schneider, K. Kumar, S. Ziegler, H. Waldmann, *Cell Chem. Biol.* **2018**, *25*, 1095-1106.

[23] L. Kremer, E. Hennes, A. Brause, A. Ursu, L. Robke, H. T. Matsubayashi, Y. Nihongaki, I. Mejdrová, J. Eickhoff, M. Baumann, R. Nencka, P. Janning, S. Kordes, H. R. Schöler, J. Sterneckert, T. Inoue, S. Ziegler, H. Waldmann, *unpublished results*.
[24] K. Kiquchi, L. Ruffino, T. Kawamoto, T. Ajiki, J. Digiovanni, *Clin. Cancer Res.* **2005**, *11*, 5572-5580.
